# Identification of the HAK/KUP/KT Potassium Transporter Gene Family in Sweet Potato and Functional Characterization of IbHAK5A

**DOI:** 10.3390/plants15162451

**Published:** 2026-08-12

**Authors:** Fang Wang, Zhongmei Xie, Songtao Yang, Shuai Qiao, Changfeng Yang, Cuiping Li, Wei Song, Wenfang Tan

**Affiliations:** 1 Crop Germplasm Innovation and Genetic Improvement Key Laboratory of Sichuan Province, Crop Research Institute, Sichuan Academy of Agricultural Sciences, Chengdu 610066, China; wangfangsaas@126.com (F.W.); yost60@126.com (S.Y.); qiaoshuai321@126.com (S.Q.); cfyang0318@163.com (C.Y.); lcp132103007@126.com (C.L.); 17828001240@163.com (W.S.); 2 College of Biological Sciences, China Agricultural University, Beijing 100193, China; 118600683013@163.com

**Keywords:** sweet potato, potassium transporter, LK stress, IbHAK5, transcription factor

## Abstract

Potassium (K^+^) is an essential mineral element for plant growth and development. Members of the *HAK/KUP/KT* (*HAK*) gene family serve pivotal roles in K^+^ uptake, translocation and homeostasis. Although numerous *HAK* genes have been extensively identified across diverse plant species, a comprehensive genomic and functional analysis of this family in sweet potato (*Ipomoea batatas* L.) remains lacking. In this study, 22 putative *IbHAK* genes were identified and classified into four distinct clades (I–IV). A systematic characterization was performed for each *IbHAK* gene, including protein physicochemical properties, chromosome distribution, gene structure, synteny, and promoter *cis*-elements. Notably, five *IbHAK5* genes (*IbHAK5A*–*IbHAK5E*) clustered on the HAK gene tree with *AtHAK5*, *OsHAK5*, and *ZmHAK5*. This suggests that small-scale duplication events likely drove the expansion of HAK5 in sweet potato. Among them, *IbHAK5A*, a gene with broad expression across tissues and strong transcriptional induction under low-K^+^ (LK) stress, was cloned. The function was then characterized in a K transporter-deficient yeast mutant and an Arabidopsis *hak5* mutant. Transcription factor IbPTL1 directly binds the *IbHAK5A* promoter, upregulates its expression, and integrates into the K^+^ signaling. In this work, we provide foundational insights into the underlying molecular mechanisms governing K^+^ acquisition in sweet potato.

## 1. Introduction

Potassium (K^+^) is the most abundant cation in plants, typically constituting 2–10% of their dry weight [1]. It is essential for enzyme activity, osmotic regulation, expansion-driven movement, membrane polarization control, protein biosynthesis, and assimilates transport [2,3]. The K^+^ concentration in plant cells is relatively stable at 80–200 mM [4,5]. Vacuoles are the largest K^+^ storage pool, where K^+^ concentration ranges from 10 mM to 200 mM [1,6,7]; however, the concentration of potassium ions in the surfaces of roots is extremely low, ranging from 0.1 to 1 mM [4,8]. Therefore, plants must absorb K^+^ from the soil against the concentration gradient. To adapt to this low-K^+^ (LK) environment, plants have evolved intricate and complex regulatory mechanisms [9,10,11]. These mechanisms are mediated by plant K^+^ transporters and channels.

K^+^ transporters are important transporter proteins responsible for potassium absorption and transport in plant root systems. Among K^+^ transporters, the HAK family members are responsible for high-affinity potassium ion absorption under LK conditions [12,13,14].These potassium transporters in plants were first discovered in Arabidopsis and barley [15,16,17]. Thirty members have been reported in Arabidopsis, among which AtHAK5 is the most important high-affinity potassium uptake transporter. It primarily functions under LK conditions, with the *hak5* mutant essentially ceasing growth at potassium levels below 10 μM [5]. Twenty-seven members have been reported in rice, where OsHAK5 has a function similar to that of Arabidopsis AtHAK5 and participates in potassium ion homeostasis under LK conditions [18]. OsHAK1 has dual affinity and functions under both high and low potassium conditions, with both mediating K^+^ uptake in the roots and K^+^ transport from roots to shoots in rice [19]. Additionally, 27 members have been reported in maize, where ZmHAK5 and ZmHAK1 have both been functionally characterized, with ZmHAK5 showing the highest similarity to AtHAK5, followed by ZmHAK1. Both are important high-affinity potassium ion transporter proteins responsible for potassium uptake in maize [20]. The *HAK* family genes are all highly conserved. In recent years, homology-based and bioinformatics analyses have led to the identification of numerous family members in diverse plant species, such as wheat [21], tea [22], rapeseed [23], and sugarcane [24]. *HAK* gene functions are well characterized in Arabidopsis and rice, but largely unknown in other crops.

Sweet potato (*Ipomoea batatas* (L.) Lam.), a member of the *Convolvulaceae* family, is extensively cultivated in tropical and subtropical regions worldwide [25]. Sweet potato exhibits the highest requirement for potassium among major nutrients, followed by nitrogen and phosphorus [26]. K^+^ plays a crucial role in promoting the formation and transport of carbohydrates in sweet potato and enhances the activity of the root cambium [27]. K^+^ deficiency impacts root growth, the distribution of photosynthetic products, carbon metabolism, enzyme activity, photosynthetic characteristics, and yield in sweet potato. Conversely, applying potassium fertilizer can enhance sucrose synthesis in sweet potato leaves and promote the conversion of sucrose to starch in storage roots. This process enhances starch accumulation and provides a material foundation for root development and tuber enlargement [28].

The absorption of K^+^ by plant roots from LK soil mainly relies on high-affinity HAK family potassium transporters located on root cell plasma membranes [29]. In sweet potato, a K^+^ preferring crop, it is important to investigate its K^+^ absorption and utilization mechanism. However, research into HAK family members in sweet potato (IbHAKs) remains limited and insufficiently systematic. The key high-affinity K^+^ transporters and their regulatory mechanism remain poorly understood. Comprehensive understanding of the structural characteristics, evolutionary relationships, and regulatory mechanisms of the *HAK* genes remains lacking in sweet potato. To address this gap, we systematically identified and characterized the *HAK* genes in sweet potato using bioinformatics methods. Furthermore, this study performed an evolutionary analysis of the *IbHAK* gene family, and uncovered a lineage-specific gene duplication event in the HAK5 clade of sweet potato. Interestingly, the sweet potato genome harbors five *IbHAK5* paralogs, whereas Arabidopsis, rice, and maize each contain only a single *HAK5* ortholog. Among them, *IbHAK5A* was notable for its pronounced transcriptional induction under LK stress and its consistently high expression across multiple tissues. Consequently, *IbHAK5A* was selected for functional validation in both transgenic Arabidopsis and a potassium transporter-deficient yeast strain. Notably, a candidate transcription factor, IbPTL1, was identified that interacts with the *IbHAK5A* promoter and functions within the K^+^ signaling pathway. The findings presented here significantly advance our understanding of the *IbHAK* gene family in sweet potato. They also provide a theoretical foundation and valuable reference for identifying key genes involved in K^+^ uptake in polyploid crops.

## 2. Results

### 2.1. Genome-Wide Identification and Evolutionary Analysis of HAK Family Members in Sweet Potato

Through blast comparison analysis of the sweet potato genome database, a total of 22 potassium transporter genes of the *IbHAK* family were identified, and their physicochemical properties were systematically analyzed. The results revealed that the sizes of the IbHAK proteins ranged from 437 amino acids (IbHAK11) to 1252 amino acids (IbHAK10). Among them, three proteins were acidic (IbHAK2, IbHAK5A, IbHAK14), and the remaining 19 were basic. The minimum number of transmembrane domains was six (IbHAK13), and the maximum was 13 (IbHAK8). Subcellular localization prediction analysis results revealed that all members of the IbHAK family are localized to the plasma membrane (Table S1).

**Figure 1:**
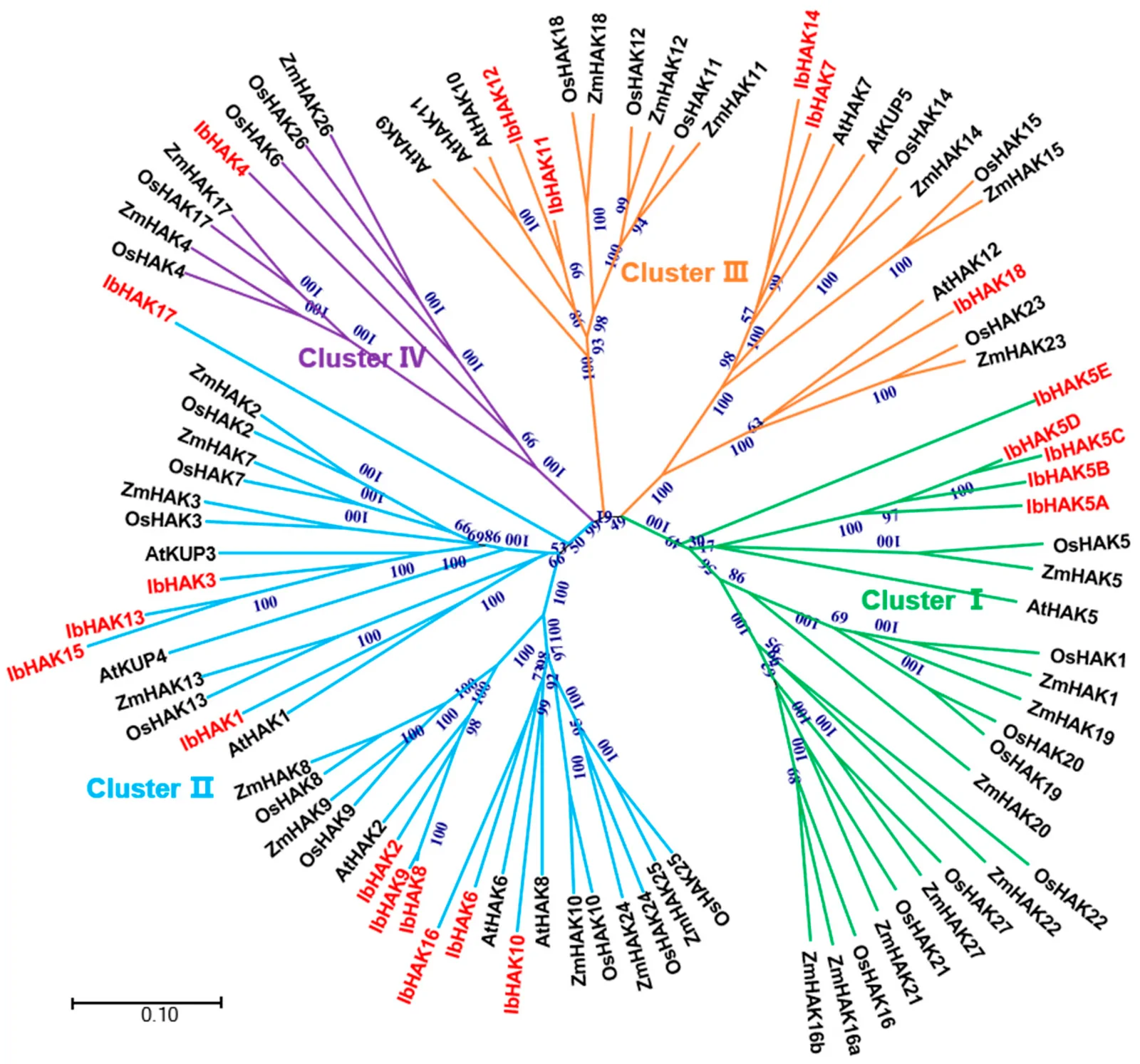
HAK protein family tree. The full-length protein sequences of 13 AtKUPs from Arabidopsis, 27 HAKs from rice, 27 HAKs from maize, and 22 HAKs from sweet potato were aligned using ClustalW. The phylogenetic tree was constructed by the neighbor-joining method in MEGA 11 software with 1000 bootstrap replicates. In total, 22 HAK family members were divided into four groups with different colors (Groups I, II, III and IV filled with green, blue, orange and purple, respectively) consistent with the classification criteria for HAK transporters in maize, rice, and Arabidopsis. All the IbHAKs were marked with red and named largely according to their evolutionary relationships with Arabidopsis. Ib: *Ipomoea batatas*, At: *Arabidopsis thaliana*, Zm: *Zea mays*, Os: *Oryza sativa*.

The evolutionary tree was constructed for 22 IbHAK family members, combined with their homologous proteins from Arabidopsis, rice, and maize (Figure 1). The results revealed that the 22 IbHAK proteins were unevenly distributed across four major clusters (I–IV). The first cluster comprised five *IbHAK* genes (designated *IbHAK5A*–*IbHAK5E*), which are closely related to the well-characterized *HAK5* orthologs from rice, Arabidopsis, and maize. This finding suggests a close evolutionary relationship, implying that these genes likely perform similar functions in potassium uptake and transport. Cluster II encompassed the largest number of *HAK* genes, comprising 11 *IbHAK*s. Cluster III contained five *IbHAK* genes, and Cluster IV comprised the fewest *IbHAK* genes, with only one member. Nearly all *HAK* genes in sweet potato share a closer evolutionary relationship with those in Arabidopsis, reflecting their shared ancestry within the eudicot clade. In contrast, all *HAK* genes from rice and maize cluster more closely with each other, consistent with their classification within the monocot lineage.

### 2.2. Chromosome Distribution, Conserved Protein Motifs and Domains, Gene Structure, Collinearity Analysis, and Cis-Acting Elements of the HAK Gene Family

**Figure 2:**
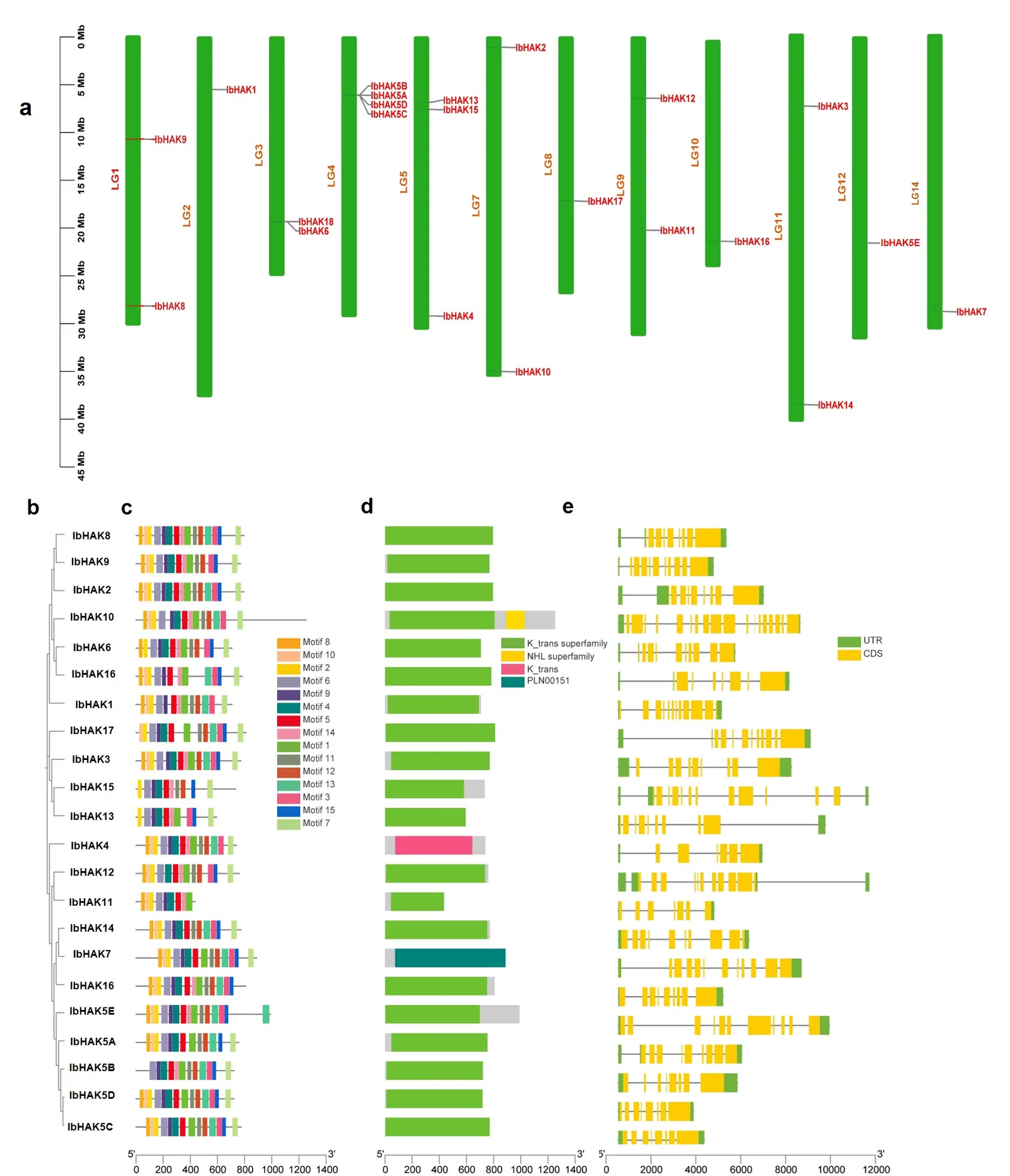
Chromosomal locations, evolutionary relationships, conserved motifs, conserved protein domains, and gene structure of IbHAK family members. (**a**) Distribution of *IbHAKs* on the sweet potato chromosomes. The legend on the left indicates the size of the chromosomes. The genes were mapped using the MG2C. LG1–15 represents sweet potato chromosome 1–15; (**b**) The evolutionary tree was constructed according to the full-length sequences of 22 IbHAK proteins. (**c**) The conserved motifs of IbHAK proteins. The different colors on the sequences denote various motif types. (**d**) The domain of IbHAK proteins. The protein motifs were predicted using MEME program (Version 5.5.9); the conserved protein domains were searched in NCBI-CDD database. The gray regions indicate areas without specific domains, while the colored sections mark the locations of the K_trans domain. (**e**) The gene structure of *IbHAK* genes. UTR, untranslated region. The gray horizontal lines represent intron regions, green corresponds to UTRs, and yellow corresponds to CDS regions.

An analysis of the chromosomal distribution of the 22 *IbHAK* genes revealed their uneven distribution across 12 chromosomes (Figure 2a). Among them, *IbHAK5A*–*IbHAK5D* are clustered on the same chromosome (LG4) and reside in close physical proximity, suggesting that these four paralogous genes likely arose from tandem or segmental duplication events during evolution. The analysis of the 15 conserved protein motifs revealed that motifs 2, 5, and 6 are present in all IbHAK proteins, indicating their high evolutionary conservation and potential functional importance (Figure 2b,c). In all 22 genes, IbHAK2 and IbHAK8 each contain all 15 conserved motifs, whereas IbHAK5C and IbHAK5D share an identical set of 14 conserved motifs. This suggests that IbHAK2 and IbHAK8 may have more similar functional roles and regulatory mechanisms, as do IbHAK5C and IbHAK5D. Nearly all conserved motifs are located within the functional domains, suggesting that these conserved elements are essential for the function of the sweet potato potassium transporter. Furthermore, conserved domains revealed that all IbHAK family members contain a conserved potassium transporter domain (Figure 2d). Significant differences exist in gene sequence length and the number of exons among different IbHAK family members (Figure 2e). Lastly, collinearity analyses among sweet potato (*Ipomoea batatas*), diploid sweet potato *Ipomoea trifida*, *Arabidopsis thaliana*, and *Oryza sativa* were performed (Figure S1). The results demonstrated that *IbHAK* genes share the greatest number of homologs with the diploid sweet potato, followed by Arabidopsis (dicotyledonous species) and rice (monocotyledonous species), consistent with expected evolutionary relationships.

**Figure 3:**
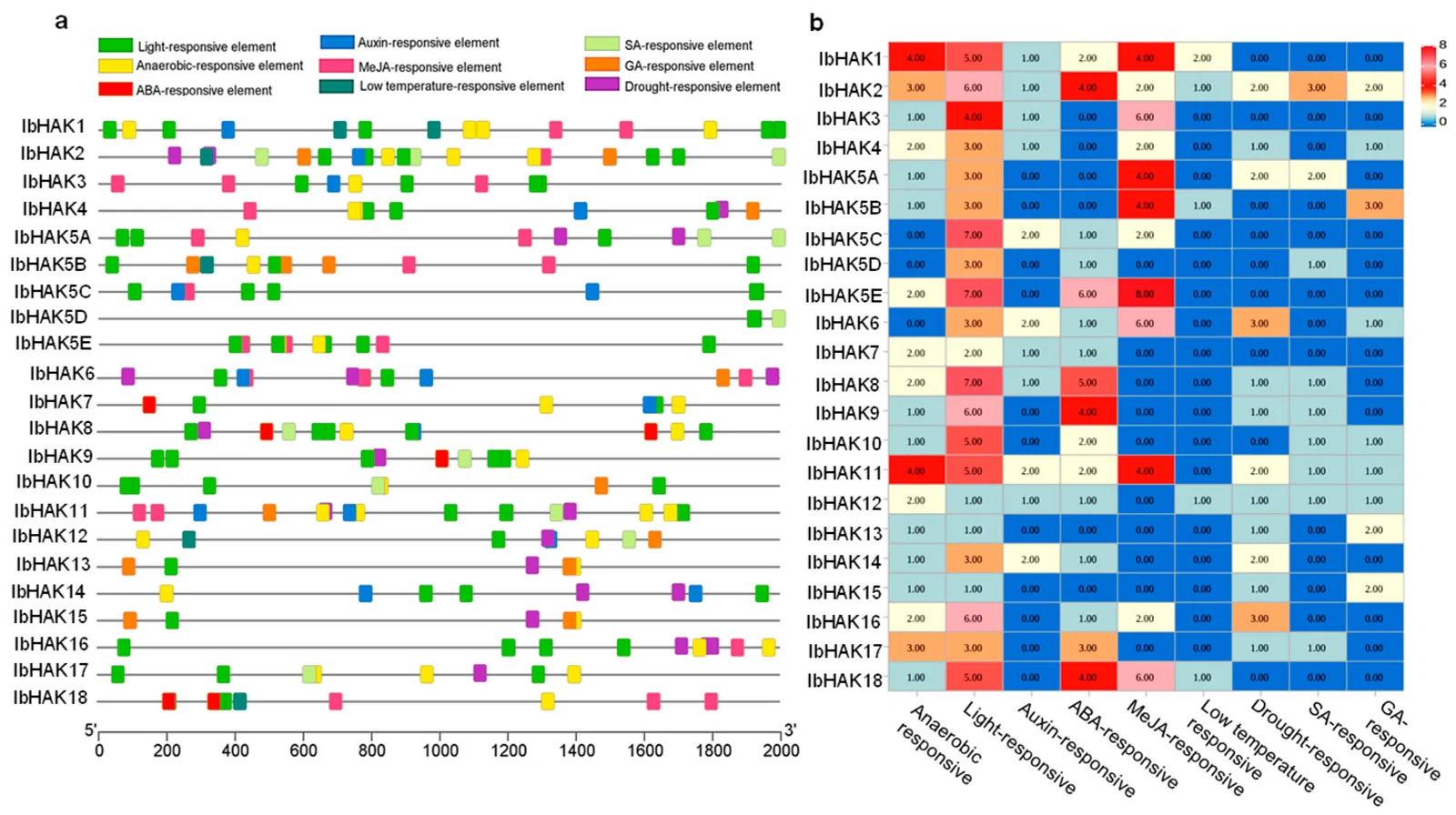
Promoter *cis*-acting element analysis of *IbHAK* genes. (**a**) The *cis*-elements in the 2 kb promoter region of *IbHAK* genes were identified using the database PlantCARE, and the map was generated by TBtools (v2.486). The promoter regions of the *IbHAKs* genes were primarily divided into two major categories of elements: hormone response-related elements and abiotic stress-related elements. (**b**) The numbers of hormone-related *cis*-elements and stress-related *cis*-elements were calculated in *IbHAK* genes, with a gradient from blue to red signifying an increasing number of elements. The numbers on the color key refer to the number of *cis*-elements.

To obtain deeper insights into the transcriptional regulatory mechanisms and potential biological functions of the *IbHAKs*, a *cis*-acting element analysis was performed on the 2 kb promoter regions upstream of the translation start codon (ATG) for each *IbHAK* gene (Figure 3a). These results indicate that the promoter regions of the *IbHAK* genes contain numerous abiotic stress-responsive and phytohormone signaling elements. Specifically, they contain numerous elements responsive to light (89), anaerobic signals (35), low temperatures (6), and drought stress (21). Hormone-responsive elements encompass auxin (15), abscisic acid (ABA) (39), methyl jasmonate (MeJA) (50), salicylic acid (SA) (12), and gibberellin (GA) (14). Among abiotic stress-response elements, the light signal response elements were the most abundant, followed by those responsive to anaerobic conditions and drought stress. In the hormone response category, the MeJA response elements were the most prevalent, followed by ABA and auxin response elements (Figure 3b). These cis-acting elements are closely associated with the biological functions of *HAK* genes, especially in mediating responses to light and drought stress [30,31], as well as to key phytohormones, including auxin, ABA, GA, and MeJA [32]. Collectively, these findings suggest that *IbHAK* gene expression is likely regulated by a complex interplay of multiple *cis*-acting elements in response to diverse abiotic stresses and hormonal signals.

### 2.3. Transcriptional Analysis of HAK Family Genes and the Subcellular Localization of IbHAK5A

**Figure 4:**
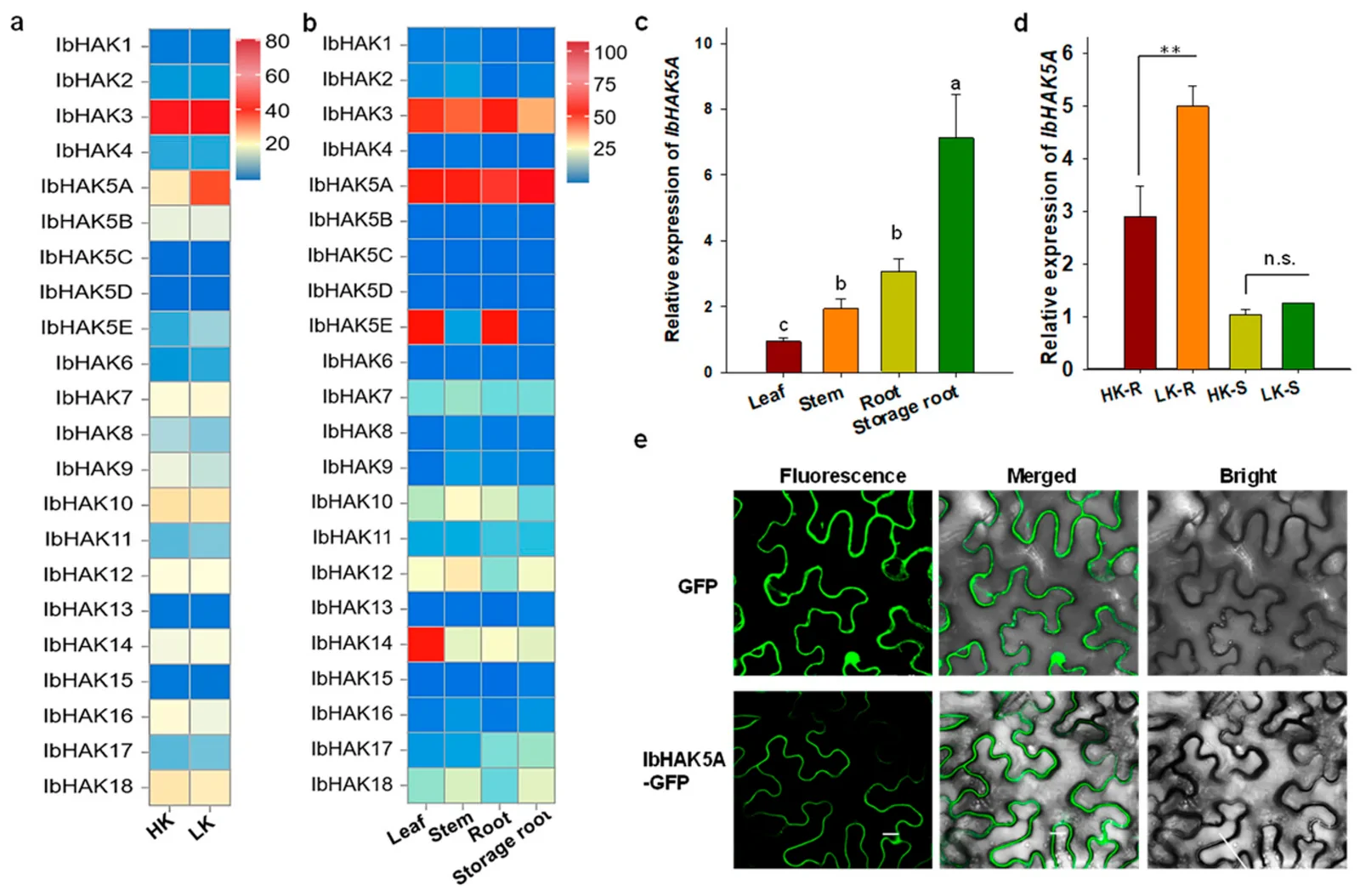
Expression profiles of *IbHAKs* in different tissues and *IbHAK5A* expression analysis. (**a**) The expression patterns of *HAK* genes under HK and LK condition. (**b**) The expression patterns of *IbHAK* genes in leaf, stem, root and storage roots were analyzed. (**c**) qPCR analysis of the expression of *IbHAK5A* in different tissue. Different lowercase letters indicate significant differences among different treatments at the *p* < 0.05 level. Data represent mean ± SE (*n* = 3). (**d**) qPCR analysis of *IbHAK5A* expression levels in response to K^+^ starvation patterns in roots and shoots of seedlings under high (1 mM) and low potassium (0 mM) conditions for 5 days. Data represent mean ± SE (*n* = 3). Student’s *t*-test (** *p* < 0.01) was used to analyze statistical significance. n.s. indicates no significance, HK-R: sufficient K^+^ condition root, LK-R: low K^+^ condition root, HK-S: sufficient K^+^ condition shoot, LK-S: low K^+^ condition shoot. (**e**) Subcellular localization of IbHAK5A in *Nicotiana benthamiana*. The expression data were obtained from RNA-seq data and shown as log2(FPKM) values. The low and high expression levels are colored with blue and red.

In this study, we evaluated eight different sweet potato cultivars, with the results demonstrating that the responses of sweet potato to LK stress vary significantly among different genotypes (Figure S2). However, the LK tolerance phenotype is primarily regulated by potassium-efficient genes. To investigate the potential biological function of the key *IbHAK* gene, we analyzed the expression profiles of 22 *IbHAK* genes according to transcriptome data under LK stress and across multiple tissues (Figure 4a,b). The results indicated that *IbHAK5A* was significantly upregulated under LK stress (log2 (fold change) = 0.52) and exhibited preferential expression in storage and uptake roots, suggesting *IbHAK5A* may play a key role in potassium acquisition and homeostasis. In addition, *IbHAK3* exhibited consistently high expression across all examined tissues, implying that this gene is responsible for a wider array of functions in sweet potato. To verify the transcriptome data, we performed RT-qPCR analysis. As expected, *IbHAK5A* displayed strong expression in storage roots and was markedly induced under LK stress (Figure 4c,d), corroborating the RNA-seq data. Furthermore, to gain insight into its functional context, the subcellular localization of the IbHAK5A protein was determined (Figure 4e). Confocal imaging revealed that IbHAK5A is localized to the plasma membrane, consistent with its putative role as a plasma membrane-localized K^+^ transporter.

### 2.4. Functional Characterization of IbHAK5A in Yeast and Arabidopsis

**Figure 5:**
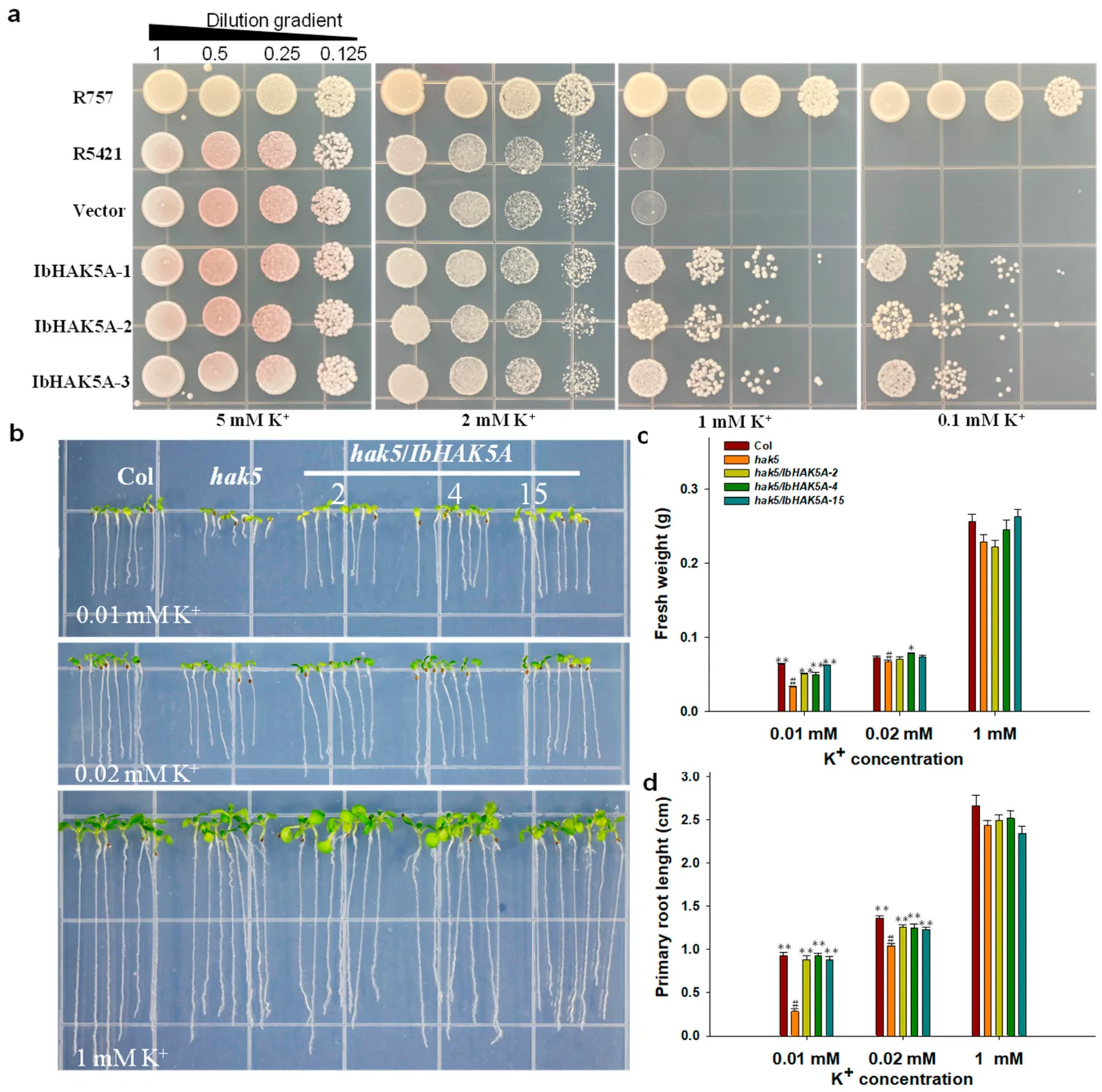
Potassium transport activity of IbHAK5A in yeast mutant and low-potassium phenotype of transgenic Arabidopsis. (**a**) Complementation of *IbHAK5A* in yeast mutant R5421 (*trk1D*, *trk2D*) under different K^+^ concentrations. Yeast cells harboring either an empty vector or *IbHAK5A* construct were grown in AP medium to an OD_600_ of 1. Equal volumes of 2-fold serial dilutions were applied to AP medium (pH 5.5) and then incubated at 30 °C for 5 days. Three biological replicates were performed for each sample. Different K^+^ concentrations phenotype comparison (**b**), fresh weight (**c**) and primary root length of various materials (**d**). Seeds were germinated and grown on low K^+^ (0.01 and 0.02 mmol/L) or K^+^ sufficient (1 mmol/L) medium for 7 days. Data represent mean ± SE (*n* = 7). Student’s *t*-test (** *p* < 0.01) was used to analyze statistical significance. # indicates control (wildtype plants, WT), 0.01 < * *p* < 0.05, ** *p* < 0.01.

To analyze the function of IbHAK5A, we constructed the CDS of *IbHAK5A* into the yeast expression vector pRS426 and transformed it into the potassium transporter-deficient yeast strain R5421. The yeast growth assays were performed on AP medium containing K^+^ concentrations ranging from 0.5 to 5 mmol/L. The results showed that IbHAK5A restores potassium transport function in the potassium transporter-deficient yeast strain, especially under LK conditions (0.1 and 1 mM K^+^ concentrations). The results confirmed IbHAK5A’s role in mediating potassium uptake (Figure 5a). The results of a previous study demonstrated that the Arabidopsis *hak5* mutant is hypersensitive to LK conditions, displaying a significantly shorter primary root phenotype compared with the wild type (Col-0) [5]. Notably, as the K^+^ concentration increases, the short-root phenotype weakens. In the present study, we generated the *hak5*/*IbHAK5A* transgenic Arabidopsis overexpression materials and comprehensively evaluated their phenotype, including primary root length and fresh weight under LK (0.01 mM and 0.02 mM K^+^) and HK (1 mM K^+^) conditions (Figure 5b–d). Our results demonstrated that the *hak5* mutant exhibited markedly reduced primary root growth relative to Col-0 under 0.01 mM K^+^ conditions (short by 1/3), whereas the *hak5*/*IbHAK5A* transgenic lines fully rescued this defect. These results demonstrate that IbHAK5A functions as a potassium transporter.

### 2.5. IbPTL1 Can Interact with IbHAK5A and Regulate the K^+^ Signaling Pathway

**Figure 6:**
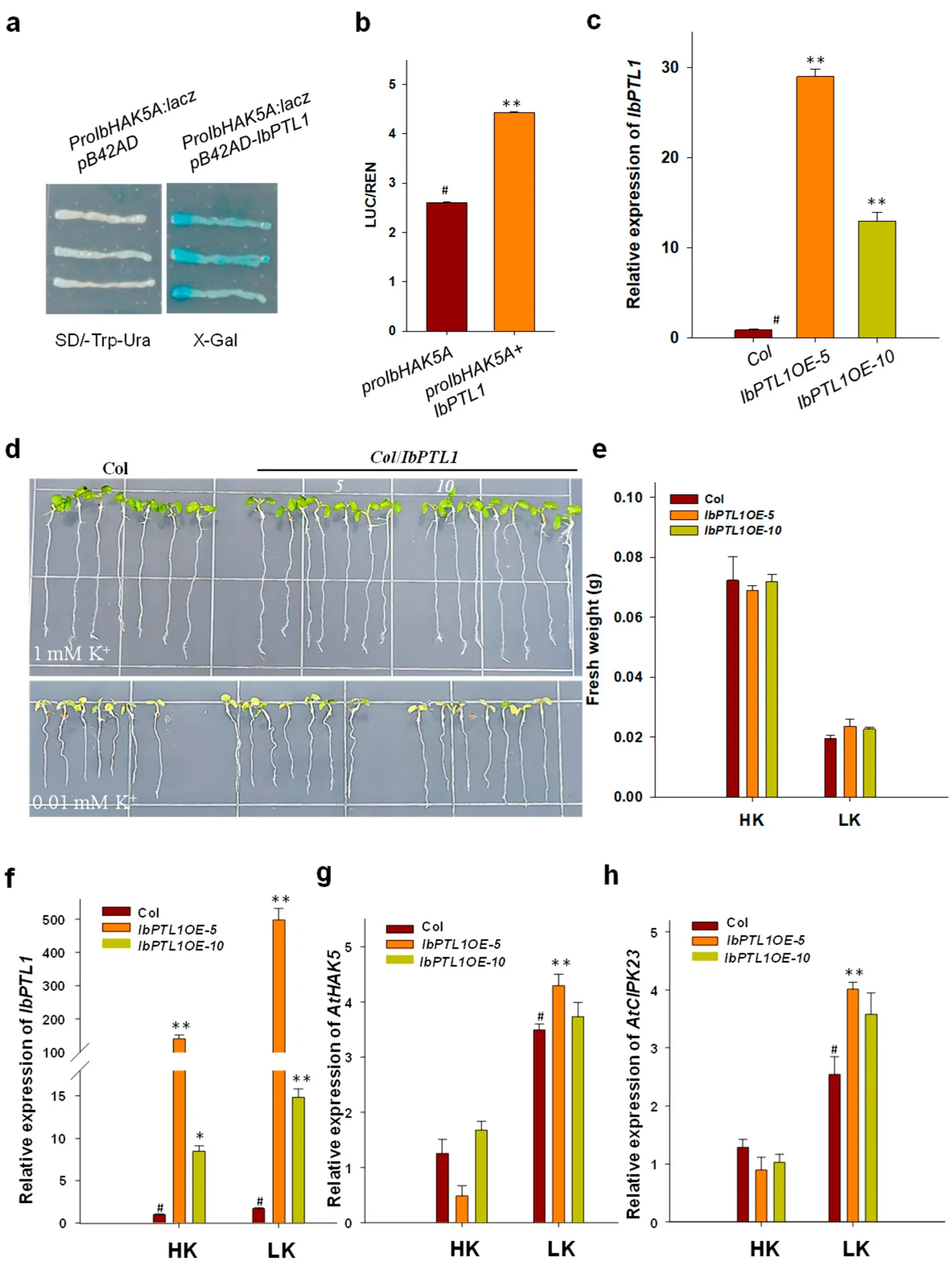
IbPTL1 binds the *IbHAK5A* promoter. (**a**) Yeast-one-hybrid assays showing that IbPTL1 can bind *IbHAK5A* promoter. *pB42AD* refers to the empty vector. *LacZ* was used as a reporter gene, driven by *IbHAK5A* promoter in yeast. (**b**) Transient expression assay showing that IbPTL1 can activate the expression of *IbHAK5A*. *IbHAK5A* promoter with the empty effector (*pGreenII 62-SK*) as the control and the *IbHAK5A* promoter with IbPTL1 as the assay. (**c**) Relative expression level analysis of *IbPTL1* in transgenic Arabidopsis. (**d**,**e**) Low-potassium phenotype of *IbPTL1* transgenic Arabidopsis and fresh weight. (**f**–**h**) Transcript levels of *IbPTL1*, *AtHAK5*, *AtCIPK23* in WT (Col) and *IbPTL1* overexpression lines under LK (0.01 mM K^+^) and HK (1 mM K^+^) conditions. Seedlings were germinated and grown on HK and LK medium for 7 days. Transcript levels were normalized to *Atactin2/8*. Data represent mean ± SE. Asterisks indicate significant differences compared with control, # indicates control, 0.01 < * *p* < 0.05, ** *p* < 0.01.

*IbHAK5A* was evidently induced under LK conditions, suggesting the involvement of one or more transcription factors that positively regulate its expression in sweet potato. Based on previous transcriptome analysis data [33] and the yeast-one-hybrid (Y1H) assay, we screened an AP2/EREBP family transcription factor, IbPTL1, as a candidate regulator of *IbHAK5A* (Figure 6a). To determine whether IbPTL1 directly regulates *IbHAK5A* transcription, we performed a dual-luciferase assay (Dual-LUC) in *N. benthamiana* leaves. In this assay, the firefly luciferase (*LUC*) reporter gene was placed under the control of the IbHAK5A promoter. The LUC activity analysis revealed that IbPTL1 could activate *IbHAK5A* expression, suggesting that IbPTL1 directly binds the *IbHAK5A* promoter (Figure 6b). Moreover, we generated the *IbPTL1* transgenic overexpression lines in Arabidopsis, obtaining two independent overexpressing lines (Figure 6c). Although no obvious phenotype was observed in *IbPTL1*-overexpressing Arabidopsis under LK stress, the expression of *AtHAK5* and *AtCIPK23* was significantly induced in these overexpressing lines (Figure 6d–h). Together, these findings indicate that IbPTL1 could bind to the *IbHAK5A* promoter and modulate the potassium signaling pathway.

## 3. Discussion

Although *HAK* family genes play an important role in K^+^ uptake, analyses of the sweet potato genome remain incomplete, and a comprehensive genome-wide analysis of the HAK family has yet to be performed [34,35]. Sweet potato contains 22 *KUP/HAK/KT* genes (Table S1), a number similar to that reported in most other plants and crops, such as tea (21) [22], saccharum (30) [24], barley (27) [36], potato (24) [37], mung bean (19) [38], and cassava (21) [39]. This finding suggests that the *HAK* gene family is highly conserved across different plant species. Notably, wheat possesses 56 *HAK* genes, significantly more than most other species, indicating that a large-scale gene duplication event occurred during its evolutionary history [21].

In this study, we identified five *IbHAK5* genes (*IbHAK5A*–*IbHAK5E*) in cultivated sweet potato. On the evolutionary tree, all five *IbHAK5* genes cluster together with *HAK5* orthologs from Arabidopsis, rice and maize (Figure 1). Among the five *IbHAK5* genes, four genes (*IbHAK5A*–*IbHAK5D*) exhibited closer similarity and were arrayed in tight clusters of tandemly duplicated genes (Figure 1 and Figure 2a and Figure S3). In contrast, *IbHAK5E* is located on a different chromosome, suggesting that *IbHAK5A*–*IbHAK5D* underwent lineage-specific tandem duplication during sweet potato evolution, whereas *IbHAK5E* was retained as a singleton copy. To trace the evolutionary origin of this expansion, we identified and analyzed the *HAK* family genes in wild diploid sweet potato (Table S2). Strikingly, we also identified five *HAK5* genes organized in a similarly compact, tandem array, suggesting that the *HAK5* gene expansion predates the whole-genome duplication events associated with the transition from diploid to hexaploid sweet potato (Figure S4). The duplicated genes, *IbHAK5A*–*IbHAK5D*, likely contribute to functional redundancy, potentially enhancing the sweet potato’s ability to acquire more K^+^ for growth and tuber expansion.

The HAK family potassium transporters have been identified as candidate high-affinity potassium (K^+^) uptake transporters, with HAK5 playing a critical role in plant growth and development under LK conditions [5]. Overexpression of *AtHAK5*, *OsHAK5*, and *ZmHAK5* can increase the K^+^ accumulation of the plant and improve potassium use efficiency (KUE) [5,18,20]. In this study, *IbHAK5A*, which was evidently induced by LK conditions and highly expressed across multiple tissues (Figure 4), was cloned and functionally characterized. Heterologous expression of IbHAK5A successfully complemented K^+^ uptake defects in both the Arabidopsis *hak5* mutant and the yeast mutant strain R5421 (Figure 5). These findings establish IbHAK5A as a critical K^+^ transporter in sweet potato. Enhancing the expression of *IbHAK5A* would be beneficial for KUE and boosting crop yield and economic value under LK conditions in future breeding and biotechnological applications. However, the role of *IbHAK5A* in K^+^ uptake and transport still needs experimental validation. This requires key genetic evidence, such as phenotypic analysis of overexpression or knockout lines in sweet potato. Moreover, as a polyploid crop, sweet potato often possesses multiple homologous copies of a given gene. Consequently, silencing or knocking down one gene may fail to produce a discernible phenotype under LK conditions due to functional redundancy among paralogs. Therefore, simultaneous knockout of major *IbHAK5* homologs will likely be necessary to unambiguously elucidate the specific physiological function of *IbHAK5A*.

Promoting K^+^ uptake under high-salt conditions is a key strategy for enhancing plant salt tolerance [19]. To gain a deeper understanding of the *IbHAK* gene family, we obtained salt stress transcriptome data for sweet potato from the NCBI database [40]. FPKM values were used to quantify gene expression levels and generate heatmap visualizations. The results revealed that *IbHAK3*, *IbHAK5B*, *IbHAK10*, *IbHAK16*, and *IbHAK18* were evidently unregulated under salt stress, suggesting their potential involvement in the salt stress response and signaling pathway (Figure S5). Moreover, the strong expression of *IbHAK3* across multiple tissues (Figure 4b) and significantly induction under salt stress, suggest that *IbHAK3* plays a pivotal role within the *IbHAK* gene family.

Transcriptional regulation is a key mechanism underlying plant responses to LK stress. The results of previous studies have demonstrated that the CBLs–CIPK23 and CBLs–CIPK1/9 complexes regulate the high-affinity K^+^ uptake mediated by HAK5 [41,42]. The AP2/ERF transcription factor RAP2.11 and the R2R3-type MYB transcription factor MYB77 directly bind to the *HAK5* promoter and activate its K^+^ uptake function [43,44]. In addition, an auxin response factor, ARF2, functions as a transcriptional repressor to negatively regulate *AtHAK5* expression in response to LK stress [45]. However, the authors of such studies have primarily focused on Arabidopsis species; thus, the regulatory mechanisms governing *HAK5* genes in crop species remain largely unknown. In this study, we screened an AP2/EREBP (ethylene-responsive element-binding protein) family transcription factor, IbPTL1. The promoter region of *IbHAK5A* contains two ethylene-responsive elements (EREs), each featuring the canonical ATTTTAAA motif, a well-characterized *cis*-acting element preferentially bound by transcription factors of the AP2/EREBP family (Figure S6, Supplementary Materials). The yeast one hybrid and dual-luciferase assays revealed that IbPTL1 can bind to the *IbHAK5A* promoter and activate its expression (Figure 6a,b). Furthermore, results of RT-qPCR analysis revealed that *IbPTL1* expression was significantly upregulated under LK conditions, and the key LK response marker genes (*AtHAK5* and *AtCIPK23*) were also upregulated in transgenic Arabidopsis overexpression lines of *IbPTL1* (Figure 6f–h). These findings indicate that IbPTL1 functions within the potassium signaling pathway and acts as a positive regulator of *IbHAK5A* expression. Nevertheless, functional validation of IbPTL1 in sweet potato is required to conclusively establish this regulatory relationship in its native context.

Genotypes tolerant to LK conditions may be used to breed sweet potato varieties with improved potassium use efficiency and to elucidate the genetic basis underlying sweet potato responses to LK stress [46]. Tolerance to LK stress is a complex quantitative trait, strongly influenced by genotype–environment interaction [47]. In the present study, we screened eight sweet potato cultivars with diverse genetic backgrounds for LK tolerance (Figure S2a). Vine length and fresh weight, two key growth indicators under LK stress, were quantified (Figure S2b,c). Our results showed that ‘Chuanzishu6’, Chuanshu218’, ‘chuanshu294’, and ‘Chuanshu217’ are relatively LK-sensitive phenotypes, whereas ‘chuanshu228’, ‘chuanshu20’, ‘chuanshu225’, and ‘Nanshu88’ exhibited clear LK tolerance. These LK-tolerant cultivars represent valuable genetic resources for future breeding programs aimed at improving potassium use efficiency in sweet potato. However, LK-responsive molecular marker genes remain to be developed.

## 4. Materials and Methods

### 4.1. Plant Materials and Growth Conditions

The Arabidopsis wild-type (Col-0) and *hak5* mutant were generously provided by Professor Yi Wang from China Agricultural University. The overexpression constructs of *IbHAK5A* and *IbPTL1* in Arabidopsis were independently generated in this study, with the CDS of *IbHAK5A* and *IbPTL1* genes cloned into the vector pSuper1300. Thereafter, the constructed vectors were introduced into *Agrobacterium tumefaciens* (GV3101) and used to transform the Arabidopsis *hak5* mutant and Col plants by means of the mature floral dip method. Transgenic *Arabidopsis thaliana* seeds were surface-sterilized and germinated under 1/2MS conditions with hygromycin B resistance, until T4 homozygous transgenic seeds of *hak5*/*IbHAK5A* and *Col/IbPTL1* were obtained. Lastly, the qRT-PCR method was used to screen the transgene lines used in this study. The plants were grown in growth chambers under controlled conditions: 22 °C, a 16 h light/8 h dark photoperiod, and 70% relative humidity.

For the LK germination phenotype experiment, seeds that had undergone vernalization for 3 days were placed on solid media with sufficient potassium (HK, 1 mM K^+^) and LK (LK, 0.01 mM and 0.02 mM K^+^) conditions for light cultivation (22 °C, 16 h light/8 h dark). After 7 days, phenotypic photographs were taken, and the primary root lengths of each material were measured using ImageJ software (v1.51) [45]. Seven biological replicates were used for Arabidopsis primary root lengths, and three biological replicates were applied for fresh weight count. Student’s t-test was used to assess statistical significance. All graphical analyses and statistical tests were performed using SigmaPlot v10.0 (https://systatsoftware.com/).

The experimental materials ‘TaiZhong6’ and ‘NanShu88’ were provided by the National SweetPotato Industry Technology System Resource Library. ‘ChuanShu294’, ‘ChuanZiShu6’, ‘ChuanShu228’, ‘ChuanShu20’, ‘ChuanShu225’, ‘ChuanShu218’, and ‘ChuanShu227’ are sweet potato varieties independently bred by the Crop Research Institute of Sichuan Academy of Agricultural Sciences and were used in this study. For the LK phenotype experiment, uniform sweet potato stem segments (approximately 15 cm) were collected in the field and grown in vermiculite to the three-leaf stage and then transferred to Hoagland hydroponic solution for HK (1 mM K^+^) and LK (0.01 mM K^+^) phenotype experiments, with fresh solution supplied every 3 days. After 20 days, phenotypic and physiological data were recorded. Three biological replicates were performed for each sample. The conditions in the artificial climate chamber were set to 28 °C, with a light/dark cycle of 16 h/8 h.

### 4.2. Identification of HAK Family Members

The sweet potato whole-genome sequence and annotation files were downloaded from the Sweetpotato Genome Database (https://sweetpotao.com/download_genome.html, accessed on 20 March 2024). Blast analysis in TBtools was then performed using 13 HAK family members of Arabidopsis to initially obtain proteins homologous to the Arabidopsis HAK family. Further analysis of the domain integrity and reliability of the HAK family members was conducted using the CD search tool (https://www.ncbi.nlm.nih.gov/Structure/cdd/wrpsb.cgi, accessed on 20 March 2024) and SMART (https://smart.embl.de/, accessed on 20 March 2024), with an E-value threshold of <0.001. Removal of incomplete or low-confidence sequences resulted in a final set of 22 high-confidence HAK family members. Next, TBtools software was used to calculate physicochemical properties, including amino acid count, relative molecular weight, isoelectric point, and number of transmembrane domains. Lastly, the subcellular localization predictions were performed using the online website Cell-PLoc 2.0 (http://www.csbio.sjtu.edu.cn/bioinf/Cell-PLoc-2/, accessed on 10 May 2024), providing insights into the potential cellular compartments in which these transporters function.

### 4.3. Evolutionary Analysis of HAK Family Members

The protein sequences of 22 sweet potato, 13 Arabidopsis, 27 rice, and 27 maize HAK family members were analyzed in MEGA 11 software using the neighbor-joining method [48]. An evolutionary tree was subsequently constructed, with 1000 bootstrap replicates; all other parameters were set to their default values. Clades were classified according to the established subgrouping criteria for the Arabidopsis *HAK* gene family.

### 4.4. Gene Locations, Conserved Protein Motifs, Conserved K^+^ Transporter Domains and Gene Structure Analysis of HAK Family Members

The 22 identified *IbHAK* gene IDs, together with the downloaded sweet potato genome GFF annotation file, were submitted to TBtools [49]. Gene chromosomal positions were determined and visualized using the Gene Location Visualize from the GTF/GFF function. Subsequently, the corresponding HAK protein sequences were then analyzed separately in MEGA 11 software for multi-sequence alignment. An evolutionary tree was constructed using the neighbor-joining method. MEME (http://meme-suite.org/tools/meme, accessed on 25 May 2024) was subsequently used to identify the conserved motifs using default parameters, comprising a maximum of 15 motifs. The results were saved in XML format. Lastly, the conserved domains of sweet potato HAK proteins were obtained from the NCBI’s CDD database and combined with the sweet potato genome GFF annotation file, evolutionary tree, conserved motif analysis file, and sweet potato genome file, and a final visualization analysis was performed in the Gene Structure View (Advanced) in TBtools (v2.486).

### 4.5. Collinear Relationships Between HAK Family Members

MCScan X [50] was employed to identify tandem and segmental duplication events within the *HAK* gene family using default parameters. Additionally, it was used to analyze collinearity among the genomes of sweet potato (*I. batatas*), wild diploid sweet potato (*I. trifida*), Arabidopsis (*A. thaliana*), and rice (*O. sativa*). The collinear relationship between genome pairs was visualized using TBtools.

### 4.6. Cis-Regulatory Elements of the HAK Gene Family

The 2000 bp promoter sequences of each *HAK* gene were extracted using TBtools. These sequences were then submitted to the PlantCARE database (http://bioinformatics.psb.ugent.be/webtools/plantcare/html/, accessed on 25 May 2024) to predict the putative *cis*-acting regulatory elements in the promoters. Excel software (v2021) was used for statistical analysis of various *cis*-acting elements, and heatmaps and TBtools software were employed for visual analysis. All promoter sequences of *IbHAKs* have been provided in the Supplementary Materials.

### 4.7. Subcellular Localization of IbHAK5A and Yeast Complementation Assay

The CDS sequence of *IbHAK5A* was constructed into the pSuper1300:GFP vector, and then the fused vector and the empty pSuper1300:GFP vector were transformed into *Agrobacterium* strain GV3101 via electroporation. Subsequently, the transformed agrobacteria were infiltrated into the lower epidermis of tobacco leaves. After 3 days, subcellular localization was examined by confocal laser-scanning microscopy (LSM800; Carl Zeiss, Oberkochen, Germany).

For the potassium transporter-deficient yeast complementation experiment, the CDS of *IbHAK5A* was constructed into the yeast expression vector pRS426. The empty pRS426 vector and the IbHAK5A-pRS426 construct vector were co-transformed into the potassium transporter-deficient yeast strain R5421 (ura3–52his3*Δ*200 leu2 *Δ*1 trp1 *Δ*1ade2 trk1 *Δ*::HIS3 trk2 *Δ*::HIS3), a K^+^ uptake-deficient mutant in which both endogenous high-affinity potassium transporter genes (TRK1 and TRK2) have been deleted [51]. The mutant yeast strain R5421 grows normally under HK conditions, but grows slowly in LK conditions. The WT yeast strain R757 was used as the control. After 3 days, positive clones were selected and cultured in YPDA medium supplemented with 0.1 M KCl until reaching an OD_600_ of 0.8. The cells were then harvested and washed three times with sterile water. After resuspension, the initial OD_600_ was adjusted to 1.0, followed by a two-fold serial dilution (2:1 dilution ratio). Equal volumes of each dilution were spotted onto AP medium containing different concentrations of K^+^ [20].

### 4.8. RNA-Seq Analysis and Real-Time PCR Analysis

Based on transcriptome raw data from sweet potato (NCBI SRA accession: PRJNA760652; PRJNA1041734; PRJNA1065644) obtained across multiple tissues and under LK and salt-stress conditions [33,40], gene expression levels were quantified as fragments per kilobase of exon per million fragments mapped (FPKMs). These FPKM values were subsequently used for heatmap visualization.

For the expression assay, different tissues (stem, leaves, root, storage root) were collected at 80 days (the sweet potato expansion stage). For the LK stress-induced assay, 15 cm stem segments were collected from plants grown under field condition. These segments were cultured in vermiculite until reaching the three-leaf stage and then transferred to Hoagland’s nutrient solution containing HK (1 mM K^+^) and LK (0 mM K^+^) for 5 days. At the end of treatment, roots and shoots were harvested, immediately frozen in liquid nitrogen, and stored at −80 °C for subsequent analysis.

Total RNA was extracted from the samples using a Polysaccharide Polyphenol Plant Total RNA Extraction Kit (TIANGEN; DP441; Beijing, China), following the manufacturer’s instructions. RNA concentration and purity were assessed using a NanoDrop 2000 spectrophotometer (Thermo Fisher Scientific, Wilmington, DE, USA). The sweet potato cultivar ‘Taizhong6’, whose genome has been fully sequenced and assembled [52], was selected as the reference genotype for transcriptome profiling and RT-qPCR analysis. The qRT-PCR experiment for different tissues and LK stress was performed with three biological replicates. The qRT-PCR cycling program consisted of an initial denaturation at 95 °C for 10 s, followed by 40 cycles of 95 °C for 15 s and 60 °C for 1 min. *IbACTIN* served as the reference gene for normalization, and SigmaPlot 10 software was used for graphical analysis. All primer sequences employed in this study are listed in Table S3.

### 4.9. Yeast One-Hybrid (Y1H) Assay

The 2000 bp promoter region of *IbHAK5A* was cloned into the pLacZi vector, whereas the full-length CDS sequences of *IbPTL1* were cloned into the pB42AD vector. The resulting reporter plasmids were co-transformed into the yeast strain EGY48 following the protocol outlined in the Yeast Protocols Handbook (Coolaber, YH3010, Beijing, China). Transformants were first selected on SD/-Trp/−Ura at 30 °C for 3 days. Positive colonies were then patched onto fresh SD/-Trp/-Ura plates supplemented with X-gal (5-bromo-4-chloro-3-indolyl-β-D-galactopyranoside), 2% galactose, and 1% raffinose for blue color development. All assays were repeated in three independent biological replicates. The respective pairs of primers are shown in Table S3, and the promoter sequence of *IbHAK5A* is shown in the Supplementary Materials.

### 4.10. Dual-Luciferase Assay

The pGreenII0800 vector containing the *IbHAK5A* promoter and the pGreenII-SK-62 vector containing the CDS of *IbPTL1* were respectively transformed into *Agrobacterium tumefaciens* GV3101 and were transiently expressed in *Nicotiana benthamiana* leaves by means of infiltration. Three days post-infiltration, firefly luciferase was detected using a Promega GloMax96 instrument, and Renilla luciferase was measured using the Dual-Luciferase Reporter Assay System (Promega).

## 5. Conclusions

In conclusion, we identified 22 *IbHAK* genes in sweet potato. Notably, five *IbHAK5* genes exhibited high similarity to *HAK5* orthologs from Arabidopsis, rice and maize, suggesting that these *HAK5* genes underwent gene duplication events during sweet potato evolution. One of the highly expressed genes, *IbHAK5A,* was cloned and functionally characterized in both potassium-deficient yeast and Arabidopsis *hak5* mutants. Functional assays confirmed that IbHAK5A mediates K^+^ uptake and transport. Moreover, the transcription factor IbPTL1 was found to be able to directly bind the *IbHAK5A* promoter and modulate its expression. Taken together, our findings provide valuable insights into the evolutionary diversification and functional roles of *IbHAK* genes in sweet potato. Nevertheless, further studies are necessary to elucidate the specific physiological contributions of individual IbHAK transporters. Future efforts should include the integration of targeted genome editing with comparative genomics and transcriptomics analyses across sweet potato germplasm exhibiting natural variation in K^+^ uptake efficiency, thereby enabling precise dissection of each *IbHAK* gene’s role in potassium nutrition and stress adaptation.

## Data Availability

The datasets presented in this study can be found in online repositories. The transcriptome expression data are available in the National Center for Biotechnology Information SRA database, accession numbers PRJNA1041734, PRJNA760652 and PRJNA1065644.

## Supplementary Materials

The following supporting information can be downloaded at https://www.mdpi.com/article/10.3390/plants15162451/s1, all supplementary figures, the CDS, promoter and protein sequences of *IbHAKs* genes have been provided in Supplementary Materials.

## Abbreviations

The following abbreviations are used in this manuscript:

LK	low-K^+^
HK	high-K^+^
CDS	The code sequence
KUE	potassium use efficiency
FPKM	the fragments per kilobase of exon per million fragments mapped
SA	salicylic acid
GA	gibberellin
Dual-LUC	dual-luciferase assay
