# Supplementary material for: Identification of the HAK/KUP/KT Potassium Transporter Gene Family in Sweet Potato and Functional Characterization of IbHAK5A
Source: Plants (Basel). 2026 Aug 12;15(16):2451. doi: 10.3390/plants15162451 (PMC13516474; doi:10.3390/plants15162451)
Supplement: Supplementary file 1 [file plants-15-02451-s001.zip › supplementary file 1.pdf]

Protein sequences

>g5020.t1/IbHAK1

MSPTSSAMNPTPSPECRGQGMASKRNLNKVPCSTVLTLAYQSLGVVYGDLSTSPLYVYKT

IFSGKLNLKEDDEEIYGVLSFIFWTFTIIALFKYIFIVLSADDNGEGGTFALYSLLCRHA

RLSMLPNQQDTDQKLSAYAIEGSEDTRQTAALKSFFERYPSFRNGLLIFVLLGTCMAIGD

GIFTPTISVLSAVSGVQVKLKGLHENYIVVISCIILVALFSIQHHGTHRALSPFYMLRFL

KVTGWEGFVSLGGIVLSITGVETMFADLGHFSTLSVKIAFTSLVYPCLMLAYLGEAAFLS

RHHEDIQRSFYKAIPEPIFWPVFLVATLAAVVGSQAVISATFSIISQCSALHCFPRVKIV

HTSSKIYGQIYIPEINWILMCLCLAVTIGLRDTNKIGAAYGLTVTTVMFVTTCLMAMVMV

VVWKKKILTAAAFVAFFGSVELLYLSAAIYKIREGAWIPLLLSIIFMGIMFIWNYGTLKK

HQFDLENKVSMERILALGPGLGIVRVPGIGLIYTNLVTGIPAIFGHFVTNLPAFHEVLVF

VCVKSVQVPYISEEERFLICRVGPKEFKETKPTQTLASGNSDRLVLEAPEYRLSNLSYTA

QSTHDIRVIISGERNAETCPGIQDESRDIMRARESGVAYIFGHSYAKAKKSSSIIKKFAI

NIVYAFLSKNSRGPDVVLNVPHTSLLEVGMLVLIYLLRFYSLISRIH*

>g25454.t1/IbHAK2

MDLENGKCWDTAKKSSWKTTLILAYQSLGVVYGDLSISPIYVYKSTFAEDIHHSETNEEI

FGVLSFVFWTLTLVPLFKYVFIVLRADDNGEGGTFALYSLICRHAKVSLLPNRQISDEAL

STYKLEPPPEAKNSSKVKMVLEKHRSLHTALLILVLLGTCMVIGDGLLTPAISVFSAVSG

LELSMSKDHHQYAVIPITCVILVCLFALQHYGTHRIGFAFAPVVLTWLLCISALGLYNIF

HWNPHIYKALSPYYMVKFLKKTRQRGWMSLGGILLCITGSEAMFADLGHFSYAAIQIAFT

FLVYPALILAYMGQAAFLSRHHHTIHQIGFYVSVPESVRWPVLVVAILASVVGSQAIISG

TFSIINQSQSLGCFPRVKVVHTSDEIHGQVYIPEINWILMILCVAVTIGFRDTKHMGNAS

GLAVMAVMLVTTCLTSLVIILCWHKPPILALCFLLLFGSIELLYFAASLIKFLEGAWLPI

LLSLFLLTVMFVWHYATIKKYEFDLQNKVSLEWLLALGPSLGISRVPGIGLVFTDLTSGI

PANFSRFVTNLPAFHGILVFVCIKSVPVPFVPPAERYLVGRVGPASHRSYRCIVRYGYRD

VHQDVDSFESELVSILADFIRYDWYKSHGVIDDVDGDAPAGASPGEYRLAVIGTVALSRS

PAFDADDESVQPASVSAGFPTVESVTEMLPPPPAAAAGKRVRFAVDEESASEVDSRSEME

IQMQSELEELYAAQQAGIAFILGHSHVRTKQGSSLFKRLAINFGYNFLRRNCRGTDVSLK

VPPASLLEVGMVYVV*

>g42476.t1/IbHAK3

MYQVDINRQEDGAELPPPLAASASEVSSHVGGREGSAVHRKKQSKSFGVVYGDLSTSPLY

VYKSIFVDKLQKHQTPDVIFGAFSLVFWTITLLPLLKYVVVVLSADDNGEGGSFALYSLL

CRHAKFSLLPNQQAADEELSAYKYGSTGHSASSLSLKRFLERHKKARTVLLIIVLLGACM

LIGDGVLTPPISVLSAISGIQDTAESLPNGRFNLPSLFVMALSAVVLITCVILVGLFALQ

HVGTHRVAFLFAPIVIIWLISIFAIGLYNTIVWNPNILSALSPYYIVKFFRETGKDGWIS

LGGVLLSVTGTEAMFADLGHFNSFSMRLAFVFVVYPCLVVQYMGQAAFLSKNIPSISNSF

YDSVPGGAHWPVFVIATLASIVGSQSIITATFSIVKQCNALGCFPRVKIVHTSRHIYGQI

YIPEINWILMILTLAVAIGFRDTTLIGNAYEGLYLSASVIKVPQGGWVSLVLSLVFLFVM

FVWHYGTRKKYSFDLHNKVPLRWLLGLGPSLGIVRVPGIGLIYSELATGVPSIFSHFVTN

LPAFHSVLVFVCVKSVPVPYVAPEERFLIGRICPRPYRMYRCIIRYGYKDIQRNDGDFEE

QLIQSIAEFIQMEAVEPQLSSSESLSFDGRMAVISTKSIQSSSSLIVRDDDLGSSYSIQS

SKSPTLQSLRSAYDDENMQVRRRRVRFQLADTPGMDPAVREELTDLIQAKEAGAAYILGH

SYVKARRSSSFLKKLVIDIAYSFLRKNCRGPSVALNIPHISLIEVGMIYYV*

>g20629.t1/IbHAK4

MEVSKPFSPVSDKNDPENGRRFSGRRCFSDSQLVFPAATPTGDTMMSFYQPYRSHQLKAG

ISYSYPPMETLLLAYQSLGVVYGDLGTSPVNVFSATQLINLSEEDLLGTLSLIFWALTIL

VLIKYTFIVIHADDHGEGGTFALYSQLCQHINIRSKLAIQSCMKGEPDDGPVYYGGSGGQ

LRAKTKKFIENSPRTQNFLTFIVLLGTCMVIGDGTLTPATCVISALQGIQSVSPKITQDH

VVFMSAVLLIVLFAFQHCGTSKVSFAFSPIMLLWFATNTSIGIYNIIKYNPSILRAISPH

YMVKFFIRNHKTAWDLLGAVFLGITGAEAMFADLGHFNKRAIQLGFCFVVYPSLVITYAG

ETAYLVRYPEKITNAYYSSIPKAVYWPMFGISTLAAIVASQSMISACFSIVKQSLSLECF

PRVKIVHTSSKHEGQVYCPEINYTLMVLCVALVIGFKGGVELANAYGVVVIWVMIITTFL

TALVMLLIWNTNILLILGFFLPYILIEGFFMTSLLRKIPQGGWVPFAISVILFAVMISWT

YGSSKKKEYEAERKMALTELDEMLSVSYIYRTPGTCFFFTDLVNGIPPIVRHYIQHTGSI

QEVMVIVTVRTVPIKTVPPEERFSVQKLGVKGAYRCLVQFGYKDSQNVGGEECVSSMIAR

LQEAAESSGEKQKISSATQRGVIFVVGRTILKANGQNGRFSRFTINYLYRFLQKNCRGAI

STAEFPLDKTLQVGMLYEI*

>g13675.t1/IbHAK5A

MADETTIHGHTDDHHETSVDGGETLAAGNNTTTRLTDRKLSWSKLCRVDSLSLEAGRVSF

APSHHRASSRDNWQRTLHLAFQCIGVVFGDIGTSPLYVYSSTFTKIDNKNDILGVLSLVI

YTIILLPLLKYVCIILWANDNGDGGTFVLYSLMCRHMSVSLRPNQEPEDIELSDYRLEAP

SNELKWAQKIKEKLESSMLSKTVLFLVTMLATSMVIGDGVLTPCVSVLSAVSGVKSLGED

AVMWISIAILVLLFNVQRFGTDKVGYVFAPALCLWFSFICGIGLYNLCKYDITVLRAFNP

MYIVDYFRRNGKEGWASLGGVVLCITGTEAMFADLGHFNVRAVQISFSGVVLPAVLIAYC

GQAAYLTKFHVAAPLYWPMFAIAVMAAIIASQALISGAFAIISQSLSLGCFPRVKVIHTS

SKYEGQVYIPEVNWILMILCIVVTYSFKTTTQINNAYGIAVVSVMLITTCMASLIMLVIW

KTQIWLVALFFMAFISIEGIYLSAVLYKFTQGGYLPLLFALVLMMVMVVWHYVHKERYSF

ELNKVSSEYVRDLAENPEVKRVPGIMLLYSELVEGIPLIFPHYVSNAGWAKEYRMFRCVV

RYGYNDKIEEPKEFEGQLVDHLMEFIRQEHFIAGSPLALENIHEQPLPTEQVDESLENVS

ARLSSAEIQTVPSTRTNEAVAGWAEDVQFVQHAKEQGGVFYLLGEATVQAKHDSCFFKKL

VVNYVYSFLRKNFRQEENVFAVPQDQHLKVGMVYEI*

>g13673.t1/IbHAK5B

MADDTTIHGRTDDHHETSVDDDETLAAPGNNTTTKLKDRKVSWAKLRRVDSLNLEAGRIS

FTPSHHHASSRDNWKITLHLAFHCIGVIYGDIGTSLCAETGAKIKQKLESSMAAKTVLFI

VTILATSMVIGDGVLTPCISVLSAVSGIKSLGQDAVMWVSIAILVLLFSVQRFGTDKVGY

AFAPAVCLWFTFISGIGLYNLFKHDVTVLRAFNPKYIVDYFRRNGKEGWVSLGGIVLCIT

GTEAMFADLGHFNVRAVQISFSGVVCPALLTAYCGQAAYLSKYPDHVANTFYDSIPDPLY

WPMFVVAVAAAIIASQALISGAFAIISQSLSLGCFPRVKVIHTSAKYEGQVYIPEVNWIL

MILCVVVTFGFKTTTQIGHAYGIAVMNVMVITTCMVTLIMLVIWKTNIWLVASFFVVFIA

IEGIYLSAVLYKFTEGGYLPLLFALVLMIVMGIWHYVYKERYAFELNNKVSSEYVRDLAK

NPDVKRVPGIMLLYSELVQGIPPIFPHFVSNIRSLHSVVVLVSIKNLPISKVVPAERFLF
RQVEPREYRVFRCVVRYGYNDKISEPKEFEGQLVDHLTEFIRHEHFIVGGPPHPEQPLPH
RKSTSSSTVHVEETLENVSARLSSASIQSMNAIRSAHSSTRANVAAAGWEEEVKFVQRAK
EQGGVFYLLGEAAVEAKQDSWFFKKFVVNYVYSFLRKNFRQGEKVFAIPQNRLLRVGMTY
EI*

>g13677.t1/IbHAK5C

MADDTTIHDHHETSVDDDETTLGAGNNVTTARLKDRKISWAKLRRVDSLNLEAGRVSFAP
TNHHASSRENWRRTLHLAFQCIGVIYGDIGTSPLYVYASTFTKIDNKNDILGVLSLIIYT
IILVPMLKYVFIVLWANDNGDGGTFALYSLMCRHTRVSLRPNEEAEDRELSNYRLETPSN
ELRRAQMIKQKLESSMVAKTVLFLVTILGTSMVIGDGVLTPCISVLSAVSGIKALGEETV
MWVSIAILVLLFSVQRFGTDKVGYAFAPGLCLWFSFIIGTGLYNVFKHDITVLRAFNPKY
IVDYFRRNGKEAWVSLGGTILCITGSEAMFADLGHFNVRAIQHIVAKLHTSPSTLTKSPI
RFMPQSLANPLYWPMFVVAVAAAIIASQALISGAFAIISQSLSLGCFPRVKVIHTSAKYE
GQVYIPEVNWILMILCVVVTYCFKNTIQIGNAYGIAVMNVQVITTCMVTLIMLVIWKTNI
WLVALFFLVFIAIEGLYLSAALYKFMQGGYLPLLLSLVLMIVMGIWHYVYKERYAFELKN
KVSSRVREGFSQESRREKSARNHASLLRACPRNSADFPHFVSNIQSLHSVVVLVSIKNLP
ISKVVPEERFLFRQFEPREYRVFRCVVRYGYNDKIGEHKEFEGQLVSHLMEFIRHEHFVA
PEQPSPHMNSASFDETLQNVSGRLSSTSIRSINAAGAGWEEEVQFVQHAMEQGGVFYLLG
EAAVEAKQDSWFFKKLVVNYVYNFLRKNFRQGEKVFAIPQNRLLRVGMTYEI*

>g13676.t1/IbHAK5D

MAQNTAIHGRVNSLNLEAGRVSFAPSNHHGSSRCIGVIYGDIGTSPLYVFASTFTKIDNK
NDILGVLSLIIYTIILVPMLKYVFIVLWANDNGDGGTFALYSVMCRHTRVSLRPNEEAED
RELSNYRLETPSNELRRAQMIKQKLESSMVAKTVLFLVTILGTSMVIGDGVLTPCISVLS
AVSGIKALGEETVMWVSIAILVLLFSVQRFGTDKVGYAFAPALCVWFSFIIGTGLYNVFK
HDITVLRAFNPKYIVDYFRRNGKEAWVSLGGTILCITGSEAMFADLGHFNIRAVQLHTSP
STLTKSPIRFMPQSLANPLYWPMFVVAVAAAIIASQALISGAFAIISQSLSLGCFPRVKV
IHTSAKYEGQVYIPEVNWILMILCVVVTYCFKNTIQIGNAYGIAVMNVQVITTCMVTLIM

LVIWKTNIWLVALFFLVFIAIEGLYLSAALYKFMQGGYLPLLLSLVLMIVMGIWHYVYKE

RYAFELNNKVSIEYVRDLAKNPDVKRVPGIMLLYTELVQGIPPIFLHFVSNIQSLHSVVV

LVSIKNLPISKVVPEERFLFRQFEPREYRVFRCVVRYGYNDKIGEHKEFEGQLVSHLMEF

IRHEHFVAPEQPLPHMKSASFDETLQNVSGRLSSTSIRSISAAGAGWEDEVQFVQHAMEH

GGVFYLLGEAAVEAKQDSWFFKKLVVNYFYSFLRKNFRQGEKVFAIPHNRLLRVGMTYEI*

>g49793.t1/IbHAK5E

MSSVVEPTLHDESESSGIEPTTDEISSQVSSTPLQGRNNSWYKLKRLDSLDLESNKLKGK

HAHHHASKGFGWSVVLQLAFQSIGVVYGDIGTSPLYVYSSTFTNPIKNDDDILGVLSLIY

YTITLIPLIKYVFIVLRANDNGDGGTFAMYSLICRYAKVGLIPSQEAEDGDVSTFKLELP

NHRVRLASKLKSNLENSTVAKFILLFATMLGTSMVIGDGVLTPCISVLSAVGGLKEATSK

LTDDTVVWISVAILVFLFMIQRFGTDKVGYSFAPIICLWFFSIAVIGVYNLAKHDHTVLK

AVNPKYIVDYFRRNKKDAWISLGGVVLAITGTEALFADVGHFSVRSIQISMCSVTYPALI

LAYTGQAAFLRNHKDLVSDTFYKSIPKSVYWPMFGVAVAAAIIASQAMISGTFSIIQQSL

SLGCFPRVKIVHTSAKYEGQVYIPEVNYLLMVACVAVTLGFKTTARIGNAYGIAVVFVMT

LTSTLLVLIMIMIWKTNIFLVILYVLVIGSVELLYLSSVLYKFDQGGYLPLAFALFLMFI

MFTWNMVYRKKYYFELENKVSSDKVMEILTKTNSCRLPGLAVFYSELVHGIPPIFKHYVE

NVPALHSVLVFVSIKSLPISKVPVEERFLFRRVEPREMCVFRCVVRYGYTDVRNEEEPFE

RLLVERIKEFVRDDIVFRALTNDKEGDERSGEISSNSKGEDVERLSQRDIEVVDRAWRSG

VRNLRQSYKVFDIPQKRMLKSYGASKNGSGWLEKQGGKGSKSSWTWKWPGGQSGDRDFGC

GFGSWGMSQVDPEKRVERGLSRNGHGGQSSDKDFGCGFGSVVITLGLQRLLRQTKFKSKG

IFEDLFGVEMGGESLHRGLDASRISFDASRTSSVQQLEPNSRLDASRASSEPSCFEEPRL

DASNPCLDASRMLCSTEIQICRISYFPLFWSPLLKGSFPKTLDLSCVLEAKRIECKGVVS

SRKSNRDPQLLGRKLSPKGAIKALGGRREA*

>g12041.t1/IbHAK6

MDLESGSCQNRAKRESWRQVLILAYQSLGVVYGDLSTSPLYVYKSTFAEDIEHSRSNDEI

YGGGTFALYSLLCRHAKVNSLPSCQLADEELSSFKKDIINPAPATLGGRLKSSLERYRVL

QRFLLVLALIGACMVIGDGILSPALSVFSAVSGIELAMAKEHHKYIEVPVACVILIVLFS

LQHYGTHRVGFLFAPVVITWLLCISAIGLYNIFRWNPFVYQALSPYYMYKFLKKTQRGGW
MSLGGILLCITGSEAMFADLGHFTQMSIQIAFTFMVYPSLVLAYMGQAAYLTKHHDFESG
YHISFYISVPEKLRWPVLVIAVLAAVVGSQAIITGTFSIIKQCSSLGCFPRVKILHTSSR
MHGQIYIPEINWTLMLLCLAVTIGFRDTKRLGNASGLAVITVMLVTTCLMSLVIVLCWHR
SVVLAIGFVLFFGTMEAFYFSASLIKFCEGAWVPIALSFLFMIIMCVWHYGTIKKYEFDV
QNKVSIDWLLSVGPSLGIVLVFLCVKYVQVPHVRPEERFLVGRVGPREYRMYRCIVRYGY
RDAHKDDLEFENDLICSIAEYIRAGDPTKDDEEMAVVGSPSTHLNRVQVFQEDGHHEGDP
SSSSTGILELREIKSPRKRVRFVVPESPNINANARGEMRDLMEARESGIAYILGHSYMKA
KNGSGLTKKMAINLGYEFLRRNSRAPTYAFSVPHASTLEVGMVYNV*
>g59493.t1/IbHAK7
MDNNNKLGPVYTVPNGYIYSNGAVAKVTVYDTGKTVQTDLFIHSCKAKRFEVSDEEKEVK
FQRRWRRRDRRGRMVSGWRRWIRSSRVGCFRTRRGSEMDNDGTDTGESVDESMQLNGIDS
DDEDNAEQRLIRTGPRIDSFDVEALEVPGVHKNYYEDATLGRRIILIIQTLGVVFGDVGT
SPLYTFSVMFSKAPVNGNEDVLGALSLVIYTLILIPLVKYVLIVLWANDGGEGGTFALYS
LICRYANVSLLPNQLPSDTRITSFRLKVPSPELERSLKIKERLETSLTLKKLLLMLVLAG
TSMVIANGVVTPAMSVVSAVAGLKVGVSIEQDQVVMISVAFLVILFSVQRYMTSKVGLVV
GPALFVWFCSLGGIGIYNIVKYDNRVWRAFNPVHIYYYFKRNSTKAWYSLGGCLLCATGS
EAMFADLCYFSVHSVQLTFLFLVLPCLLLGYLGQAAYLMDNHSDTTHAFFSSVPTLIASR
AMTTATFSCIKQSTALGCFPRLKIVHPSRKFMGQIYIPVMNWFLLALSLVLICSISNIYE
IGNAYGIAELGMMMITTILVTVVMLLIWQINIIIVLSFVTIFLGVELTFFSSVLWSVGDG
SWIILVFAVVLFLVMYIWNYGSKLKYETEVMKKMSMDLLRELGPNLGTVRAPGIGLLYNE
LAKGVPEIFGHFLTTLPAVHSMIIFVCIKYVPVPVVPQNERFLFRRVCPKSYHMFRCIAR
YGYKDARKENHQTFEQLLIESLEKFICREAQERSLESDGDSDSEEESTFSRVLVAPNGSV
YSLAVPLLADFEDKGKLVVEESTSSDPNISDAEQSLEKELSFLRTAKESGVVYLLGHADI
RARKDSWFVKKLIINYLYAFLKKNSRRGICNLSVPHSHVIQVGMTYMV*
>g3900.t1/IbHAK8
MDLEHGKCWDTTKKNSWKTTLILAYQSLGVVYGDLSISPLYVFKSTFAEDIQHSDTNEEI

FGVMSFVFWTLTLVPLFKYVFIVLRADDNGEGGTFALYSLICRHAKVSLLPNRQVSDEAL
STYKREEPPERNNKVSRVKLVLEKYKFLHTALLILVLLGTCMVIGDGLLTPAISVFSAVS
GLEFYMSKDHHQYAVVPITCVILVCLFALQHYGTHRIGFCFAPIVLSWLICISGLGLYNI
LTWNPHVYKALSPYYMFKFLKKTRKDGWMSLGGILLCITGSEAMFADLGHFSYAAIQTAF
TFLVYPALILAYMGQAAFLSRHHHNMHEISFYLSVPGSVRWPVLIIAILASVVGSQAIIS
GTFSIINQSQSLGCFPRVKVVHTSAKIHGQIYIPEINWILMILCVAVTIGFRDTKHLGNA
SGLALMAVMLVTTCLTSLVIVLCWHKPPILALVFLVFFGSIELLYFSASVIKFLEGAWLP
ILLALILVTVMFVWHYATIKKYEYDLHNKVSLDWLLALGPSLGIARVPGIGIVFSDLTSG
IPANFSRFVTNLPAFHRVLVFVCVKAVPVPFIPPAERYLVGRVGPPSHRSYRCIVRYGYR
DVHQDVDFFETELVSKLADFIRYDWYKTHGIITSPNDDDGSRSGASSSEYRLAVIGTMAF
RHTAFEIEETAQPESVSFGFPTAESLTDIIEMQPTTTAAEKRVRFAVDDSEQDSHSHQEM
DIQLQEELRSPRSPRSGIAFILGHSHVRTKQGSSIFKRLAINFGYNLLKRNCRGPDVSLK
VPPVSLLEVGMVYVV*

>g1704.t1/IbHAK9

MVSAIYNSVHRNIGIDMDLEHRKCWDTTKKNSWKTTLILAYQSLGVVYGDLSISPLYVFK
STFAEDIQHSDTNEEIFGVMSFVFWTLTLVPLFKYVFIVLRADDNGEGGTFALYSLICRH
AKVSLLPNRQVSDEALSTYKREEPPERNNRVSRVKLVLEKYKFLHTALLILVLLGTCMVI
GDGLLTPAISVFSAVSGLEFYMSKDHHQYAVVPITCVILVCLFALQHYGTHRIGFCFAPI
VLSWLICISGLGLYNILTWNPHVYKALSPYYMFKFLKKTRKDGWMSLGGILLCITGSEAM
FADLGHFSYAAIQTAFTFLVYPALILAYMGQAAFLSRHHHNMHEISFYLSVPGSVRWPVL
IIAILASVVGSQAIISGTFSIINQSQSLGCFPRVKVVHTSAKVHGQIYIPEINWILMILC
VAVTIGFRDTKHLGNASGLALMAVMLVTTCLTSLVIVLCWHKPPILALVFLVFFGSIELL
YFSASVIKFLEGAWLPILLALILVTVIDLTSGIPANFSRFVTNLPAFHRVLVFVCVKAVP
VPFIPPAERYLVGPVGPPSHRSYRCIVRYGYRDVHQDVDFFETELVSKLADFIRYDWYKT
HGIITSPNDDDGSRSGASSSEYRLAVIGTMAFPGTPAFEIEETAQPESVSFGFPTAESLT
DIIEMQPTSTAAEKRVRFAVDDSEQDSHSHQEMDIQLQEELRDLHEAREAGIAFILGHSH
VRTKQGSSIFKRLAINFGYNLLKRNCRGPDVSLKVPPVSLLEVGMVYVV*

>g30149.t1/IbHAK10

MTAQLPHWASPHGSPLQIGVRNAFLLMILRRPVMDIEGWGRGNPVKKESWRTVLALAYQS

LGVVYGDLATSPLYVYKSTFAEDIHHSETNEEIFGVLSFVFWTLTLIPLLKYVFIVLRAD

DNGEGGTFALYSLLCRHARVSTLPNGQVADEDLFEYKKDGIHSANKGLGLSLRSTLEKHQ

WLRRILLVLALIGTCMVIGDGVLTPAISVFSAVSGLELSMSKHHHESNLLAIYSFLSQVC

LNAMEVVPGLDEYVEVPVACVILVFIFSIQHYGTHRIGFLFAPIVVTWLLCISAIGLYNI

FRWNPHVYQALSPYYMYKFLKKTQRGGWMSLGGDFVMYYSILSFCLRNCIKHRLSTEIAF

TFAVYPSLILAYMGQAAYLSKHHTLETDYRIGFYVSVPEKLRWPVLGIAILAAVVGSQAI

ITGTFSIIKQCSALGCFPRVKITHTSSKFHGQIYIPEINWTLMVLCLAVTIGFEDTKHIS

NASGLAVITVMLVTTCLMSLVIVLCWHKSVFLAIFFIFFFGTIEALYFSASLIKFLDGAW

VPVALSFIFLLIMYIWHYGTLKKYEFDVQNRVPINWLLQLSPNLGIARVRGIGLIHTELV

SGIPAIFSHFVTNLPAFHQVLIFLCIKSVPVPHVRPEESSRRIYFAVSNNSSDRKPPPEA

PERGDLIDFDDDERMTVIGTTSTYLDGKTMSEDGENMSATATSTTELREISSPEMMTVTG

RRVRFLVPESPQMDPSVRAELRDLMEAREAGMAFILGRCYVKAKSGSSFMKKVVIDLGYD

FLRRNSRGPTYALSFPRASTLEVGMIYLQASMRKLNKTQGRRKECGDLYSLTTSATPTAK

MVCGVVTNVVAAVFKWLVSLKSEMEPAFSRIKFEKGYNIETIFDGNKLGVEPYSVEVSPA

GEVFILDSENSNIYKITTPHSLYSQPILAVGSPEGCSGHVDGKLREARMNHPKGLAMDDS

GNMYVADAMNMAIRKITDTGVVTIAGGKRFQGDRPREDDAKLSNDFDVVYVRSSCSLLVI

DRGNRAIKQVQLHNHDCYDQYDSNLHLGAALLFASGFFGYTLALLQRKVAAFFSSIHRHT

NPIRPGLVPTEDEHKKPAENMFVSIGRLLVNIASSVTEMFGRTLSAFRKKPLQPHFQQNL

HYSHKHSQYYTNTIGYEDDLPFSDQRYPPPRTSSYSSMAKDMERTQHFRQHHHQKKQPSS

PQTCTEQRCERNEVVFGAVQEQDGCGEAMVIKKIDYSDPAYNHYNIRSRYGN*

>g36794.t1/IbHAK11

MASGMEIDEDENETKGGMWVLDQKLDQPMDEEAGRIRNMYREKSLGVVYGDLGTSPLYVF

YNTFPRGIKDTEDVIGALSLIIYSLTLVPLLKYVFLVCKANDNGQGGTFALYSLLCRHAK

IKTIPNQHRTDEDLTTYSRNTFQEHSCAAKTKRWLEAYAFRKNALLILVLVGTCMVIGDG

ILTPAISVLSASGGIKVDHPKMSNDVVVLVAVFILVGLFSMQHYGTDKVGWMFAPVVLLW

FLIIGGIGIFNIWKYDTSVLKAFSPLYIYHYLRRGRKEAWISLGGIMLSITGTEALFADL

AHFPLSAIQLAFTVIVFPCLLLAYIGQAAYLMQHKDHVAEAFYRSIPESIYWPVFVIATL

AAIVASQATISATFSIIKQALALGCFPRVKVVHTSKKFLGQIYIPDINWILMILCIAVTA

GFRNQSQIGNAYGEFTK*

>g34971.t1/IbHAK12

MEFDDDSSETKGGMWVLDQKLDQPMEEEAGRLKTMYREKKFSSILLLQLAFQSLGVVYGD

LGTSPLYVFYNTFPHGIEDTEDIIGALSLIIYSLTLIPLLKYVFIVCRASDNGQGGTFAL

YSLLCRHAKIKTIPNQHRTDEELTTYSRNTFHEQSFAAKTKRWLEAYAFRKNALLILVLV

GTCMVIGDGILTPAISVLSASGGIQVEHPKMSTDIVVLVAVLILVGGIGIFNIWKYDSSV

LKAFSPVYIYRYFRRGRKDAWTSLGGIMLSITGTEALFADLSHFPLAAIQLAFTVIVFPC

LLLAYCGQASYLTQHRDHVSDAFYRSIPDSIYWPVFIVATLAAIVASQATISATFSIIKQ

ALALGCFPRVKVVHTSKKFLGQIYIPDINWILMVLCIAVTAGFRDKSQIGNAYGTAVVIV

MLVTTLLMILIMLLVWHCHWIIVVIFTGLSLVVECTYFSAVLFKVDQGGWVPLVIAAAFL

IIMYVWHYGTVKRYEFELHSKVSMAWILGLGPSLGLVRVPGIATHSVVVFVCVKYLPVYT

VPEDERFLVKRIGPKNFHMFRCVARYGYKDLHKKDDDFEKKLFDNLFTFVRLESMMEGCS

DSDEYSVYGQQTQQSRDFLLAKNNGNTCTSTDLTVSSVDSIVPVQSPRHGCNMTMSSGRD

STQTEVDELEFLNACRDAGVVHILGNTVVRAGRDSRFYRKIAIDYVYAFLRKICRENSAI

FNVPHESLLNVGQIIYLSIASGCAVLLCSANTLMTDHKIN*

>g17621.t1/IbHAK13

MPLLKYVFVVLSADDNGEGGPFALYSLLCRHAKFSLLPNQQAADEELSAYKYGLSRQSAS

SLPFKRFLEKHKKSRTVLLLVVLLGACMVIGDGVLTPPMAVLSSISGIQAAAEKMSNGEV

LLITCIILVGLFALQHSGTHRVAFLFAPIIIIWLVSMFSLGLYNTIFWNPKVVSALSPHY

IVKFFPVTGRDGWISLGGVLLSVAGTEAMFANLGHFTAFSMRLAFVFGVYPCLVVQYMGQ

AAFLSKNIASIPNSFYGTVPGSVYWPVFVIATLAAIVGSQSIITATFSIVNQCNALGLLP

RSRLSTPQTIFMGRCLPVSIINEIPTRRMGLRCAALLYSCLSCLFGTTDPERGVPSVFSH

FVTNLPAFHSVLVFACVKSVPVPHVPPEERFLIGRICPRPYRMYRCIVRYGYKDIQRDNG

NFEDLLIQSIAEFIQMEAEEPQLSSSDSFYGRMAVVSTRTIQSSSSLVIPTEEDLGKNHS

FQNSKSPSLQNLRSFYADENKKIRRRGVRFRVAERLAMDTAVREELEDLIEAKEAGVAYI

MGHSYVKARRSSSFLKKIAIDIAYLFLRRNCRAPSVALNIPHISLIEVGMIYYV*

>g46647.t1/IbHAK14

MADEGSEREYGGLGSLDSIESRWVFQDEEDESEIYNDGTDAGESGDDSLPRDGMDSDDED

NAEQRLIRTGPRIDSFDVEALEVPGAQRNDFEDASVMRKIALAFQTLGVVFGDVGTSPLY

TFSVMFSKAPVTGDEDVLGSLSMVLYTLFLFPLVKYVFIVLWANDDGEGGTFALYSLLCR

HANVSLLPNQLPSDSRISSFRLKVPSPELERSLKIKERLESSVTLKKLLLMLVLAGTSMV

IAVGVVTPAVSVMSAMGGLKVGVAAIQQDEVKMISVAFLVILFSLQKYGTSKMGIVVGPA

LFIWFCSLGGIGLYNLTKYDNRVWRAFNPFHIYNYFKRDSTKAWRALGGCLLCATGSEAM

FADLCYFSVRSIQLTFLLLVLPCLLLGYLGQAAYLMENHADTKEVFFSTLPSIAELGVMM

MSTALVTIVMLLIWQINIIIVLSFVFIFLGLELTFFSSVLWCVGDGSWIILVFAVVLFLI

MYIWNYGSKLKYETEVMNKMSMDLLRELGPNLGTIRAPGIGLLYNELAKGVPAIFGHFLT

TLPAVHSMVIFVCIKYVPVPVVAQKERFLFRRVCLKSYHIFRCIARYGYKDARKENHQVF

EQLLIESLEKFIRREAQERQLESDGDDDSNSGDECTFSRVVVAPNGSMFTLGVPLLADIR

DTGKTVAKESTSYGHGPGPSANSNLSDAEQSLEMELSFLHKAKESGVVYLLGHGNIRARK

DSWFIKKLIINYFYAFLRKNIKSAGRLMPRSIPTQVTFILNPSRSEARFAGGF*

>g17727.t1/IbHAK15

MPLLKYVFVVLSADDNGEGGPFALYSLLCRHAKFSLLPNQQAADEELSAYKYGLSRQSAS

SLPFKRFLEKHKKSRTVLLLVVLLGACMVIGDGVLTPPMAVLSSISGIQAAAEKMSNGEV

LLITCIILVGLFALQHSGTHRVAFLFAPIIIIWLVSMFSLGLYNTIFWNPKVVSALSPHY

IVKFFRVTGRDGWISLGGVLLSVAGTEAMFANLGHFTAFSMRLAFVFGVYPCLVVQYMGQ

AAFLSKNIASIPNSFYGTVPGSVYWPVFVIATLAAIVGSQSIITATFSIVNQCLACMTVM

FITTFLMSLIMVFVWQRSIALASIFLLFFWVIEGVYLSASLMKFPQGGWVSVALSLVFLF

VMFVWHYGSRKSPFLFPMFHPRSVSLLAVFAQDPIACTGASSDMGTRIYRDNGNFEDLLI

QSIAEFIQMEAEEPQLSSSDSFYGRMAVVSTRTIQSSSSLVIPTEEDLGKNHSFQNSKSP

SLQNLRSFYADENKKIRRRGVRFRVAERLAMDTAVREELEDLIEAKEAGVAYIMGHSYVK

ARRSSSFLKKIAIDIAYLFLRRNCRAPSVALNIPHISLIEVGQGNGVGLSVRLLLTFKVS

NWVDLRKQPELRKCKWERGREGKSAIIRNFSLRRLVGVASAVSSDLYCNNQIIDGGRDGD

YFPFPVTEMGTRAGSILPAPTLLPSLVSPTLNMAKAASQGAYMKYGMEYSLTSSFRFHGL

HAMQCLEEAACKGI*

>g40997.t1/IbHAK16

MDLETGFRHNSSKRQSWRTVLTLAYQSLGVVYGDLSTSPLYVYKSTFAEDIKHSETNEEI

YGVLSFIFWTLTLVPLLKYVFIVLKADDNGEGGTFALYSLLCRHARVNSLPSCQLEDEEL

SFYKKDIVSPAPTTVGARLKSTLERHRVLQRFLLVLALIGACMVIGDGILSPAISVFSAV

SGLELAVSKEHNKYIEVPVACIILIALFALQHYGTHRVGFLFAPVVITWLVCSEAMFADL

GHFSQLSIQLAFTFLVYPSLILAYMGQAAYLSQHHVIESDYHIGFYVSVPGRYPKPVHFL

LGRFGKFGLLKLSKTLRPEKVRWPVLVIAILAAVVGSQAIITGTFSIIKQCSSLGCFPGV

KIVHTSSKIHGQIYIPEINWTLMLLCLAVTIGFRDTKRLGNASGGCWTWPFSPPPNNLQA

IGFGCYNSYAGHNVFDVTGYGSVLAPERLPCDWLCALLRDHRSLIFLCISHQIFGRSMGA

HCNGFHLHDSHVCLALWIPKKYEFDVQNKVSVDWLLSLGPSLGIVRVNGIGLIHTELVSG

IPAIFSHFVTNLPAFHQVLVFLCIKSVPIPHVKHEERFLVGHIGPREYRIYRCIVRYGYR

DVHKDDSGFENDLVCSIAEYIRTGKAGSNGAATATAAAAAEENSAQEREEMAVMGTPSTY

LGGVYVYEDGEGHSSSAGTSELREIKSPPVLKPRKKVRFVVPESPKIDQSARTELRELME

AREAGIAYILGHSYMQAKQGSSFVKKMVINFGYEFLRRNSRPPTYALSVPHASTLEVGMV

YNI*

>g32726.t1/IbHAK17

MFFLTSTAPLYVFGSIDVKDVKSKEEIYELFSFAFWTLTIIPLLKYALIVLKADDQGEGG

TFALYSLLCRHAKVGLLPSDKTAAETMHHEEIPSKIKMRTRARWAIENHKSCHYFLLLLA

LVGSCLIICDGVLTPSISAASSEEADSIDKYLTKYIPVPTACAILVCLFTLQKYGTHKIG

FLFAPVVIIWIVFISTMGIYNISRYPSILRSVSPVYIVRFIRNADITSWKLLGRIVLCIA

GSEAMFADLGHFSKKSIQGTRLGLLLASTQQSPSHMVLDLALTGFRPTIVVTFALIIYPV

LIICYAGQAAFISNHLGVTPYVTHLSESLPISRDLHHVFTVLSLIASLVGSQATITASFS

IINQCQALGCFPRVKVIHTSDKIYGQVYIPDVTWILMVLSLGVTFGLGDITTIANATEFE

SLPPWFEPVNYRKPRLVYLLVVLCWLGHKAGFTHTQKGCLTVICGMSVTSCLMSLVIALY

WDKSLFFSVCFVLAFGSVEAMYLFSSLMNFSKGTWCVLVLTSLFLMTMLSWHYGTVKKYE

FDVENKVAVDWLTDLSPGLGVNRVQGIGFIYTEIVTGIPSFFSHFVTNLPAFHELLVFVS

FKPLPVPYIPQSKRYLIGRVGHKEYKIYRCIVRYGYCDHMRDTHDFEEHIISSIGEFIAR

EEQEDEDQEAMAEGRMIALGGPSTSAALIPVTEIEENEVSQTSADIESQRSPLIDSYPPV

KRKKVRFFLPPTSPKVNPAVRRELQELVDARERGTAYFLGKPHLSVRKGSNFFKRFLIMN

YIFLDKNCREPPVALNIPHAALLEVGMAYIV*

>g12038.t1/IbHAK18

MERGEIEESITVGLLRRTGTGGSGESRWVDGSEVDSESPPWSIHGDEGLREGYGSVRRRL

VKKPKRVNSFDVEAMEIVGDHAHHKKDLSLAGTLALAFQTLGVVYGDVGTSPLYVFADVF

SKVTITSEIDVLGALSIVIYTIALIPLMKYVFIVLKANDNGEGGTFALYSLICRYANVNL

LPNRQQADEGISSFKLKLPTPELERALNIKDALERKSFLKTLLLLLVLTGTSMIIGDGIL

TPAMSVVSAVSGLQGKIPGFGTNALVITSIVILGALFSIQRFGSSKVGFTFAPALAIWFF

SLGSIGIYNLFKYDVSVVRALNPAYIFLFFSKNSSKAWSALGGCVLCITAIDIQCRDAGA

EAMFADLGHFSVRSIQIAFTGVVFPCLLLAYLGQAAYLMKHPNSASTVFYSSVPGKNASF

RTDGFFWPIFVIATVAAIIASQAMISASFSCVKQAMALGCFPRLKVVHTSKRHMGQIYIP

VINWFLMLMCMAVVAAFRSTTDIANAYGIAEVGVMLVSTTMVTLVMVLIWQTNLFLALCF

PLVFGSIELLYLSAVLSKILEGGWLPFVFASFFLSVMYVWNYGSVLKYQVEVKQKISMDF

VYDLGSTLGTVRVPGIGLLYNELVQGIPSVFMQFLLDLPAIHLIIVFVCIKYVPVPVVPQ

EERFLFRRVGPKDYHMFRCVARYGYKDVRKEDHQAFEQLLVDSLEKFLRKEAQDLALESV

SSVTRQTDDVASTRSSDAGDGGGIEGLKVPLIQQDEGRKKAWRMMSQHCYCQLAPCQRTK

IPAWSTSCQPSAKPPNRVSLTCWGTLT*

CDS sequences

>g13675.t1/IbHAK5A

ATGGCGGATGAAACGACAATCCATGGCCATACAGATGATCATCATGAGACAAGTGTTGAT

GGCGGCGAGACATTGGCTGCAGGAAACAATACCACTACCAGACTCACAGATAGAAAGCTT

TCCTGGTCGAAACTGTGTCGTGTTGACTCCCTGAGTTTGGAAGCTGGAAGAGTTTCTTTC

GCTCCCAGCCATCACCGTGCTTCCTCTAGGGATAACTGGCAGAGAACGCTGCATTTAGCG

TTTCAGTGCATCGGGGTGGTATTCGGCGACATCGGGACTTCCCCGTTGTATGTGTACTCC

AGCACATTCACAAAGATCGACAACAAAAATGACATCCTTGGGGTTCTGTCCCTCGTCATC

TACACCATAATCCTCTTACCTCTGCTCAAGTATGTCTGCATCATCTTGTGGGCAAATGAC

AACGGCGACGGTGGAACGTTTGTGCTGTACTCGCTGATGTGTCGGCATATGAGTGTGAGT

CTGAGGCCGAACCAGGAGCCCGAGGATATAGAGCTGTCAGACTACAGGCTGGAGGCGCC
TTCGAATGAGCTGAAATGGGCACAGAAGATAAAGGAGAAGCTGGAGTCCAGTATGCTTTCT

AAAACTGTGTTGTTCCTAGTCACCATGCTTGCGACTTCCATGGTTATCGGCGATGGAGTT

CTCACTCCTTGCGTATCTGTTCTTTCTGCTGTGAGTGGGGTCAAAAGCCTTGGTGAAGAT

GCTGTGATGTGGATTTCCATTGCAATACTGGTGCTGCTCTTCAATGTGCAGCGGTTTGGG

ACAGACAAAGTAGGATATGTGTTTGCGCCAGCGCTGTGTCTTTGGTTTTCCTTCATATGT

GGGATCGGTCTGTACAACCTGTGCAAGTATGACATCACGGTCCTGCGTGCCTTCAATCCC

ATGTACATTGTGGACTACTTCCGCAGAAATGGTAAAGAAGGGTGGGCTTCACTCGGCGGA

GTTGTTCTGTGCATTACTGGGACTGAAGCAATGTTTGCGGATTTGGGCCACTTCAATGTT

AGAGCTGTTCAGATTAGTTTCTCGGGAGTGGTTCTTCCTGCCGTGCTGATTGCATATTGT

GGCCAAGCTGCATACCTCACCAAATTTCATGTTGCAGCTCCGTTGTATTGGCCAATGTTT

GCTATTGCTGTGATGGCTGCCATCATAGCCAGCCAAGCTTTGATATCTGGAGCATTTGCG

ATCATCAGCCAATCTCTAAGCTTGGGATGTTTCCCAAGAGTGAAGGTGATTCATACTTCT

AGTAAGTATGAAGGTCAAGTCTACATTCCAGAGGTGAATTGGATCCTCATGATCCTATGC

ATCGTCGTCACTTACAGCTTCAAAACTACAACACAGATCAACAATGCTTATGGGATTGCT

GTGGTGAGTGTGATGTTGATCACCACTTGCATGGCGAGTCTAATAATGCTCGTGATATGG

AAGACACAGATCTGGCTGGTTGCATTGTTTTTCATGGCCTTCATCTCCATAGAAGGCATA

TATCTTTCGGCTGTTCTGTACAAATTCACGCAGGGCGGATACTTGCCGTTGCTGTTTGCG

TTGGTCTTGATGATGGTCATGGTGGTATGGCACTACGTGCACAAAGAACGCTACTCGTTC

GAGCTTAACAAGGTTTCCAGCGAGTATGTGAGAGATTTAGCAGAGAATCCCGAGGTGAAA

AGAGTGCCAGGAATCATGCTTCTTTACTCGGAGCTGGTTGAAGGAATTCCGCTGATTTTC

CCTCACTATGTCTCCAATGCAGGTTGGGCCAAGGAGTACCGCATGTTTCGCTGCGTGGTC

CGCTATGGCTACAATGACAAAATCGAAGAGCCGAAGGAGTTCGAGGGGCAGCTGGTGGAT

CACCTCATGGAATTCATCCGGCAAGAGCATTTCATCGCTGGCAGCCCGCTGGCTCTGGAA

AACATTCACGAACAGCCTTTGCCCACTGAACAGGTCGATGAAAGCCTAGAAAACGTGTCA

GCTCGTTTATCCTCGGCCGAAATTCAGACAGTTCCCTCCACCCGGACCAACGAGGCGGTT

GCAGGTTGGGCAGAAGACGTGCAGTTTGTGCAGCATGCCAAGGAGCAAGGCGGCGTGTTC

TACCTGCTGGGAGAAGCAACAGTGCAGGCAAAACACGACTCTTGTTTTTCAAGAAATTG

GTGGTAAACTATGTCTACAGTTTCCTGCGCAAGAATTTCAGGCAGGAGGAGAATGTTTTT

GCAGTTCCTCAGGATCAGCATTTGAAAGTAGGTATGGTTTATGAAATATGA

>g13677.t1/IbHAK5C

ATGGCGGACGACACGACAATCCATGATCACCACGAGACCAGTGTTGATGACGATGAGACG

ACGTTGGGCGCAGGAAACAATGTTACTACGGCTAGACTCAAAGATCGAAAGATTTCCTGG

GCGAAACTGCGTCGTGTTGACTCCCTGAATTTGGAAGCTGGAAGAGTTTCTTTCGCCCCC

ACCAATCACCATGCCTCCTCTAGGGAGAACTGGCGGAGAACGCTGCATTTGGCGTTTCAG

TGCATCGGAGTGATATACGGCGACATCGGGACTTCCCCGTTGTACGTGTACGCCAGCACG

TTCACAAAGATCGACAACAAAAACGACATCCTCGGAGTTTTGTCCCTCATCATTTACACC

ATAATCCTCGTACCTATGCTCAAGTACGTCTTCATCGTCTTGTGGGCAAATGACAACGGC

GACGGTGGAACGTTTGCGCTGTATTCGTTGATGTGTCGGCATACTCGGGTGAGCCTGCGG

CCGAACGAGGAGGCGGAGGATAGAGAGCTGTCAAATTATCGGCTGGAGACGCCTTCGAAT

GAGCTGAGACGGGCGCAGATGATAAAGCAGAAATTGGAGTCGAGTATGGTTGCCAAAACT

GTGTTGTTTCTAGTCACAATTCTCGGAACTTCCATGGTTATCGGTGATGGAGTTCTCACT

CCTTGCATCTCAGTTCTTTCGGCAGTGAGCGGGATCAAAGCCTTGGGTGAAGAGACTGTG

ATGTGGGTTTCGATTGCGATATTGGTGCTGCTCTTTAGCGTGCAACGATTTGGGACAGAC

AAAGTGGGATATGCGTTTGCGCCGGGGTTGTGTCTGTGGTTTTCCTTCATAATTGGGACC

GGTTTGTACAACGTTTTCAAGCACGACATCACCGTCCTGCGTGCTTTCAATCCCAAGTAC

ATAGTTGATTATTTCCGCAGAAATGGTAAAGAAGCGTGGGTTTCACTTGGCGGAACTATT

TTGTGCATCACTGGGTCGGAAGCCATGTTTGCTGATTTGGGCCACTTTAATGTTCGAGCT

ATTCAGCATATTGTGGCCAAGCTGCATACCTCACCAAGCACCCTGACCAAGTCGCCAATT

CGTTTTATGCCTCAATCCCTGGCAAATCCGCTGTATTGGCCGATGTTTGTTGTTGCTGTC

GCGGCTGCCATTATAGCCAGTCAAGCTCTAATATCTGGAGCATTTGCCATCATCTCTCAA

TCTTTAAGCTTAGGATGTTTTCCAAGAGTGAAGGTGATTCACACTTCTGCTAAGTATGAA

GGTCAAGTCTACATCCCAGAGGTCAATTGGATCCTCATGATCTTATGCGTCGTCGTCACT

TACTGCTTCAAAAATACAATACAGATAGGAAATGCTTATGGGATTGCTGTGATGAATGTG

CAGGTGATCACCACTTGTATGGTGACACTGATAATGTTAGTGATATGGAAGACAAACATA

TGGTTGGTGGCATTGTTCTTCTTGGTGTTCATAGCCATTGAAGGCCTATATCTTTCGGCG

GCTTTGTACAAATTCATGCAAGGCGGGTACCTGCCGCTGTTATTATCCTTGGTTCTGATG

ATCGTGATGGGGATATGGCACTACGTGTACAAAGAACGCTACGCGTTCGAGCTTAAAAAC

AAGGTTTCAAGCCGAGTACGTGAGGGATTTAGTCAAGAATCGCGACGTGAAAAGAGTGCC

AGGAATCATGCTTCTTTACTCCGAGCTTGTCCAAGGAATTCCGCCGATTTTCCCCATTTC

GTCTCCAATATCCAGTCGCTCCATTCCGTGGTGGTTTTGGTTTCAATAAAGAATCTTCCG

ATTAGTAAAGTGGTCCCGGAGGAACGGTTTCTGTTCAGACAGTTTGAACCCAGGGAGTAC

CGTGTTTTCCGTTGTGTGGTCCGTTACGGCTACAACGACAAAATCGGAGAGCACAAGGAG

TTCGAGGGGCAATTAGTGAGTCACCTCATGGAATTTATCCGGCACGAGCATTTCGTCGCT

CCTGAACAACCTTCGCCGCATATGAATTCGGCCTCGTTCGACGAAACTCTACAAAACGTG

TCGGGCCGGTTATCCTCGACCTCAATTCGGTCAATAAACGCGGCCGGTGCGGGTTGGGAA

GAAGAAGTGCAGTTTGTGCAGCACGCGATGGAGCAAGGCGGCGTGTTCTACCTGCTGGGA

GAAGCGGCGGTGGAGGCGAAACAGGACTCGTGGTTTTTCAAGAAGCTTGTGGTTAACTAC

GTCTACAATTTCCTGCGCAAGAATTTCAGGCAGGGAGAGAAAGTTTTTGCGATTCCTCAA

AATCGGCTTTTGAGAGTCGGCATGACTTACGAAATATGA

>g13676.t1/IbHAK5D

ATGGCGCAAAACACGGCAATCCATGGTCGTGTAAACTCCCTGAATTTGGAAGCTGGAAGG

GTTTCTTTCGCCCCAAGCAATCACCATGGCTCCTCTAGGTGCATCGGAGTGATATACGGC

GACATCGGGACTTCCCCGTTGTACGTGTTCGCCAGCACGTTCACAAAGATCGACAACAAA

AACGACATCCTCGGAGTTCTGTCCCTCATCATCTACACCATCATCCTCGTACCTATGCTC

AAGTACGTCTTCATCGTCTTGTGGGCAAATGACAACGGCGACGGTGGAACGTTTGCGCTG

TATTCGGTGATGTGTCGGCATACTCGGGTGAGCCTGCGGCCGAACGAGGAGGCGGAGGAT

AGAGAGCTGTCAAACTATCGGCTGGAGACGCCTTCGAATGAGCTGAGACGGGCGCAGATG

ATAAAGCAGAAACTGGAGTCGAGTATGGTTGCCAAAACTGTGTTGTTCCTAGTCACCATC

CTCGGAACTTCCATGGTTATCGGTGATGGAGTTCTCACTCCTTGCATCTCAGTTCTTTCG

GCAGTGAGCGGGATCAAAGCCTTGGGTGAAGAGACTGTGATGTGGGTTTCGATTGCGATA

TTGGTGCTGCTATTTAGTGTGCAGCGATTTGGGACAGACAAAGTGGGATATGCGTTTGCG

CCGGCGTTGTGTGTGTGGTTTTCCTTCATAATTGGGACCGGTCTGTACAACGTTTTCAAG

CACGACATCACCGTCCTGCGTGCTTTCAATCCCAAGTACATAGTTGATTATTTCCGCAGA

AATGGTAAAGAAGCGTGGGTTTCACTTGGCGGAACTATTTTGTGCATCACTGGGTCGGAA

GCCATGTTTGCTGATTTGGGCCACTTTAATATTCGAGCTGTTCAGCTGCATACCTCACCA

AGTACCCTGACCAAGTCGCCAATACGTTTTATGCCTCAATCCCTGGCAAATCCTCTGTAT

TGGCCGATGTTTGTTGTTGCTGTCGCGGCTGCCATTATAGCCAGTCAAGCTCTGATATCT

GGAGCATTTGCCATCATCTCTCAGTCTTTAAGCTTAGGGTGTTTCCCAAGAGTGAAGGTG

ATTCACACTTCTGCTAAGTATGAAGGTCAAGTCTACATCCCAGAGGTGAATTGGATCCTC

ATGATCTTATGCGTCGTCGTCACTTACTGCTTCAAAAATACAATACAGATAGGAAATGCT

TATGGGATTGCTGTGATGAATGTGCAGGTGATCACCACTTGTATGGTGACTCTGATAATG

TTAGTGATATGGAAGACAAACATATGGTTGGTGGCATTGTTCTTCTTGGTGTTCATAGCC

ATTGAAGGCCTATATCTTTCGGCGGCTTTGTACAAATTCATGCAAGGCGGGTACCTGCCG

CTGTTATTATCCTTGGTTCTGATGATCGTGATGGGGATATGGCACTACGTGTACAAAGAA

CGCTACGCGTTCGAGCTTAACAACAAGGTTTCAATCGAGTACGTGAGGGATTTAGCCAAG

AATCCCGACGTGAAAAGAGTGCCTGGAATCATGCTTCTTTACACCGAGCTTGTCCAAGGA

ATTCCACCGATTTTCCTCCATTTCGTCTCCAACATCCAGTCGCTCCATTCCGTGGTGGTT

TTGGTTTCAATAAAGAATCTCCCTATTAGTAAAGTGGTCCCGGAGGAACGATTTCTGTTT

AGGCAGTTTGAACCTAGGGAGTACCGTGTTTTCCGTTGCGTGGTCCGGTACGGTTACAAT

GACAAAATCGGAGAGCACAAGGAGTTCGAGGGGCAGCTGGTGAGTCACCTCATGGAATTC

ATCCGGCACGAGCATTTCGTCGCTCCCGAACAACCTTTGCCGCATATGAAATCGGCCTCG

TTCGACGAAACTCTACAAAATGTGTCGGGCCGGTTATCCTCGACCTCAATTCGGTCAATA

AGCGCGGCCGGTGCGGGTTGGGAAGACGAAGTGCAGTTTGTGCAGCACGCGATGGAGCA
CGGTGGCGTTTTCTACCTGTTGGGAGAAGCGGCGGTGGAGGCGAAGCAAGACTCGTGGTT
TTTCAAGAAGTTGGTGGTTAACTACTTCTACAGTTTCCTGCGCAAGAATTTCAGGCAGGGA

GAGAAAGTTTTTGCGATTCCTCATAATCGGCTTTTGAGAGTCGGCATGACTTACGAAATA

TGA

>g13673.t1/IbHAK5B

ATGGCGGACGACACGACAATCCATGGCCGTACAGATGATCATCACGAGACAAGTGTTGAC

GACGACGAGACGTTGGCCGCGCCGGGAAACAATACTACTACCAAACTGAAAGATCGGAA
GGTTTCATGGGCGAAACTCCGTCGTGTTGACTCCCTGAATTTGGAAGCCGGAAGAATTTCT

TTCACTCCCAGCCATCACCATGCTTCTTCTAGGGATAACTGGAAGATAACGCTGCATTTA

GCGTTTCACTGCATCGGAGTGATATACGGCGACATCGGGACTTCCTTATGCGCTGAGACG

GGCGCAAAGATAAAGCAGAAGCTGGAGTCGAGTATGGCTGCCAAAACTGTATTGTTCATA

GTCACCATCCTCGCAACTTCCATGGTTATCGGCGATGGAGTTCTCACTCCTTGCATCTCA

GTTCTTTCTGCGGTGAGTGGGATCAAATCCCTCGGTCAAGACGCTGTGATGTGGGTTTCG

ATTGCGATATTGGTGCTGCTCTTCAGCGTGCAGCGATTCGGGACCGACAAAGTAGGATAT

GCGTTTGCGCCGGCAGTGTGTCTTTGGTTTACCTTCATAAGTGGGATAGGTTTGTACAAC

CTATTCAAGCATGACGTCACGGTCCTGCGTGCCTTCAATCCCAAGTACATAGTTGACTAC

TTCCGCAGAAATGGTAAAGAAGGGTGGGTTTCACTTGGCGGAATTGTTCTGTGCATTACT

GGGACCGAAGCCATGTTTGCTGATTTAGGCCACTTTAATGTTCGAGCTGTTCAGATTAGT

TTCTCGGGAGTTGTTTGTCCTGCACTGCTGACTGCATATTGTGGCCAAGCTGCATACCTC

AGCAAGTACCCTGACCACGTTGCCAATACATTTTATGACTCGATCCCTGATCCGTTGTAT

TGGCCAATGTTTGTTGTTGCTGTCGCGGCTGCCATTATAGCTAGTCAAGCTCTGATATCT

GGAGCGTTTGCCATCATTTCTCAATCTTTAAGTTTAGGATGTTTCCCAAGGGTGAAGGTG

ATTCACACTTCTGCTAAGTATGAAGGTCAAGTCTACATCCCAGAGGTGAATTGGATCCTC

ATGATCTTATGTGTCGTAGTCACTTTTGGCTTCAAAACTACAACACAGATCGGCCATGCT

TATGGGATTGCTGTGATGAATGTGATGGTGATCACCACCTGCATGGTGACTCTGATAATG

CTTGTGATATGGAAGACAAACATCTGGCTGGTGGCATCGTTCTTCGTGGTGTTCATCGCC

ATTGAAGGCATATATCTATCGGCGGTTTTGTACAAATTCACGGAGGGCGGGTACCTGCCG

CTGCTGTTCGCCTTGGTTCTGATGATCGTGATGGGGATATGGCACTACGTGTACAAAGAA

CGCTACGCATTCGAGCTTAACAACAAGGTTTCAAGCGAGTACGTGAGAGATTTAGCCAAG

AATCCCGACGTGAAAAGAGTGCCCGGAATTATGCTTCTTTACTCCGAGCTTGTCCAAGGA

ATTCCGCCGATTTTCCCCCACTTCGTCTCCAACATTCGATCACTCCATTCCGTGGTGGTT

TTGGTTTCAATAAAGAATCTCCCCATTAGTAAAGTGGTGCCGGCGGAACGGTTTCTGTTT

AGACAGGTTGAACCTAGGGAGTACCGTGTTTTTCGCTGCGTGGTCCGTTACGGCTACAAT

GACAAAATTAGTGAGCCTAAGGAGTTCGAGGGGCAGTTGGTGGATCACCTCACGGAATTC

ATCCGACACGAACATTTCATCGTCGGCGGCCCGCCCCATCCCGAACAACCTTTGCCACAC

AGGAAATCAACTTCCTCGTCCACCGTACACGTCGAGGAAACTCTAGAAAATGTGTCGGCG

CGGTTATCCTCGGCCTCGATTCAGTCAATGAACGCGATCAGGTCGGCCCATTCCTCCACC

CGGGCCAACGTGGCGGCCGCAGGTTGGGAAGAAGAAGTGAAGTTTGTGCAGCGTGCGAA
GGAGCAGGGTGGCGTGTTCTACCTGCTGGGAGAAGCGGCGGTGGAGGCGAAACAAGACT
CGTGGTTTTTCAAGAAGTTTGTGGTTAACTACGTCTACAGTTTCTTGCGCAAGAATTTCAGG

CAGGGGGAGAAAGTTTTTGCGATTCCTCAGAATCGACTTTTGAGAGTCGGCATGACTTAC

GAGATATGA

>g49793.t1/IbHAK5E

ATGTCTAGTGTTGTTGAGCCAACCCTCCATGATGAGAGTGAGAGCAGTGGCATAGAGCCA

ACCACGGACGAAATTTCTTCCCAAGTTTCATCCACCCCGCTTCAGGGCAGGAATAATTCT

TGGTACAAGTTGAAACGATTGGACTCGCTGGACCTCGAGTCCAACAAGCTTAAAGGCAAA

CACGCTCACCATCACGCCTCTAAGGGGTTTGGGTGGTCAGTGGTGTTGCAACTAGCTTTC

CAGAGCATAGGAGTGGTGTATGGAGACATCGGAACCTCACCTTTGTATGTGTACTCCAGC

ACCTTCACTAATCCGATCAAAAACGATGATGACATCCTAGGTGTCCTGTCTTTGATCTAC

TACACCATCACTCTCATCCCACTCATCAAATACGTCTTCATTGTTCTTCGTGCCAACGAC

AATGGAGATGGTGGGACGTTTGCAATGTATTCTTTGATATGCCGATATGCGAAAGTGGGG

TTAATCCCAAGCCAAGAGGCAGAAGATGGAGATGTGTCCACGTTCAAACTTGAGTTGCCT

AATCATCGTGTTCGTTTAGCTTCTAAGCTCAAGTCCAATCTTGAAAACAGCACTGTAGCC

AAATTCATCTTGCTCTTTGCCACCATGCTTGGCACTTCTATGGTAATCGGTGACGGTGTC

CTTACTCCTTGTATTTCAGTTCTGTCAGCGGTGGGAGGCTTGAAGGAAGCAACTTCAAAA

CTTACCGATGATACCGTGGTCTGGATTTCAGTTGCAATCTTAGTGTTTCTGTTCATGATT

CAAAGGTTTGGAACTGACAAAGTTGGCTACAGCTTCGCCCCCATAATTTGCCTATGGTTC

TTTTCCATAGCCGTGATCGGAGTGTACAACTTAGCAAAGCATGACCACACCGTCCTTAAA

GCCGTTAATCCTAAGTACATTGTTGACTACTTCCGGAGAAACAAAAAAGATGCCTGGATT

TCTTTGGGTGGCGTCGTTCTTGCTATAACAGGAACTGAGGCGCTGTTTGCGGATGTTGGT

CATTTTAGTGTCCGGTCCATACAAATCAGCATGTGTAGTGTTACCTATCCTGCCCTTATC

TTAGCCTACACTGGACAGGCTGCTTTCCTTCGGAATCACAAGGACTTAGTTTCTGACACC

TTTTACAAATCAATACCAAAATCAGTATACTGGCCAATGTTTGGGGTAGCTGTAGCGGCG

GCAATTATTGCGAGTCAGGCCATGATTTCTGGCACATTTTCTATCATCCAGCAATCCCTC

TCTCTAGGATGCTTTCCTCGAGTCAAGATCGTCCATACCTCCGCCAAATACGAGGGACAA

GTTTACATCCCCGAGGTCAATTACCTTCTCATGGTAGCCTGCGTTGCCGTCACTCTCGGC

TTCAAAACCACCGCCCGAATCGGCAACGCCTACGGCATCGCCGTGGTTTTTGTGATGACA

CTAACATCCACACTGCTAGTCTTGATAATGATCATGATTTGGAAAACCAATATCTTCCTG

GTAATCCTCTACGTCCTGGTGATCGGCTCAGTCGAGCTGCTTTACCTAAGCTCAGTCCTA

TACAAGTTCGATCAAGGCGGGTATCTTCCCCTCGCATTTGCCCTGTTTCTCATGTTCATC

ATGTTCACTTGGAACATGGTGTACCGAAAGAAATACTATTTCGAGCTGGAAAACAAAGTT

TCCTCAGACAAGGTTATGGAGATCCTCACTAAAACAAATTCTTGCCGTCTCCCGGGTCTA

GCGGTTTTTTACTCGGAATTGGTTCATGGAATTCCCCCAATTTTCAAACACTATGTGGAA

AACGTGCCGGCCCTTCATTCCGTTCTGGTTTTCGTCTCCATAAAGTCCCTCCCAATCAGC

AAAGTCCCCGTGGAAGAGCGGTTTCTGTTCCGCCGCGTGGAGCCGCGCGAAATGTGCGTT

TTCCGGTGCGTGGTCAGGTACGGGTACACGGACGTGCGGAACGAGGAGGAGCCGTTCGA
GCGCCTGTTGGTGGAGAGGATAAAGGAGTTCGTGAGAGATGATATCGTGTTTCGGGCACTA

ACTAACGACAAAGAAGGCGATGAGAGGTCCGGAGAGATCAGCAGCAACAGCAAGGGAGA
AGATGTGGAGAGATTGTCACAGAGAGACATTGAGGTGGTGGATAGGGCGTGGCGCAGTGG
GGTGAGGAATTTGAGGCAGAGTTACAAAGTGTTTGATATTCCGCAGAAGAGAATGCTCAAG

AGCTATGGGGCCTCAAAGAATGGTTCAGGTTGGTTAGAGAAACAGGGTGGAAAGGGGTCT

AAGTCGAGTTGGACATGGAAGTGGCCTGGTGGTCAAAGCGGTGACAGGGACTTCGGATGT

GGATTTGGTAGCTGGGGAATGAGTCAAGTTGATCCAGAAAAAAGGGTGGAAAGGGGTCTA

AGTCGAAATGGACATGGGGGTCAAAGCAGTGACAAGGACTTCGGATGTGGATTTGGTAGT

GTGGTCATTACTCTTGGATTGCAGAGGCTGCTTAGACAGACCAAATTCAAAAGCAAGGGA

ATATTTGAAGACTTGTTTGGAGTCGAAATGGGTGGAGAAAGTTTACATCGAGGCCTCGAC

GCGTCTAGGATATCATTCGACGCGTCCAGAACATCCAGTGTGCAGCAGTTGGAGCCGAAT

TCAAGGCTCGACGCGTCGAGAGCATCCAGTGAGCCATCCTGTTTTGAAGAGCCAAGGCTC

GACGCGTCGAACCCCTGTCTCGACGCGTCGAGAATGCTGTGCAGTACAGAAATCCAGATC

TGCAGAATTTCCTATTTTCCCCTGTTTTGGTCTCCACTATTAAAAGGTTCATTTCCCAAA

ACCCTAGACCTAAGCTGCGTTTTGGAGGCAAAAAGGATTGAGTGTAAGGGTGTTGTCTCT

TCAAGGAAGTCAAACAGGGATCCACAACTTTTGGGGAGAAAATTGTCACCAAAAGGAGCT

ATAAAGGCTTTGGGAGGAAGAAGGGAAGCTTGA

>g3900.t1/IbHAK8

ATGGATCTTGAACATGGGAAGTGTTGGGACACCACAAAGAAGAATTCTTGGAAGACGACA

CTGATCTTAGCTTATCAAAGTCTTGGTGTCGTTTACGGGGATCTCAGCATTTCCCCGCTC

TATGTCTTCAAGAGCACATTTGCGGAAGACATTCAGCACTCGGATACTAATGAAGAAATT

TTCGGGGTTATGTCGTTTGTTTTCTGGACTTTAACTCTTGTCCCTTTATTCAAGTATGTC

TTTATAGTCCTCCGAGCTGATGACAATGGAGAGGGTGGTACTTTCGCTCTTTATTCCCTG

ATATGCAGGCATGCCAAAGTAAGCCTTCTTCCAAACAGACAAGTTTCCGATGAAGCCCTT

TCCACGTATAAACGCGAGGAACCTCCCGAGAGAAACAATAAGGTTTCGAGGGTGAAGTTG

GTGCTCGAGAAGTACAAATTCTTGCACACGGCATTGCTAATCTTGGTGCTCCTTGGCACT

TGTATGGTCATCGGAGATGGACTGCTTACTCCCGCCATATCCGTTTTCTCTGCGGTTTCT

GGGCTTGAGTTCTACATGTCCAAGGATCACCACCAATATGCGGTGGTTCCTATAACTTGC

GTTATATTAGTCTGCCTATTCGCGTTACAGCATTATGGCACGCACCGCATTGGCTTTTGT

TTTGCGCCAATCGTGCTATCTTGGTTAATCTGCATAAGTGGGCTCGGATTGTACAATATC

CTCACTTGGAATCCACACGTTTACAAGGCGCTTTCCCCGTATTACATGTTCAAGTTCTTG

AAGAAGACTAGAAAAGATGGATGGATGTCGTTGGGAGGGATTCTCTTGTGCATAACAGGA

TCTGAAGCAATGTTTGCTGACCTCGGCCACTTCTCTTATGCTGCAATTCAGACCGCATTC

ACCTTTCTGGTTTATCCAGCACTTATATTGGCATATATGGGCCAAGCAGCTTTTTTGTCG

AGGCATCATCACAACATGCACGAGATTAGTTTCTATTTGTCTGTTCCAGGAAGTGTAAGG

TGGCCGGTTCTTATTATAGCCATTCTTGCTTCTGTCGTTGGCAGTCAGGCAATCATAAGT

GGAACATTCTCGATTATCAACCAGAGTCAATCACTAGGTTGTTTTCCAAGAGTTAAGGTT

GTTCACACCTCTGCTAAAATACATGGGCAGATCTACATCCCGGAGATAAATTGGATTCTC

ATGATCCTTTGTGTTGCTGTGACTATCGGGTTCCGGGACACGAAACATTTGGGAAATGCA

TCAGGGTTAGCGTTGATGGCAGTCATGCTGGTGACGACCTGTCTAACCTCGTTGGTTATC

GTCCTGTGTTGGCACAAGCCTCCAATTCTTGCGCTTGTCTTTCTCGTTTTCTTTGGATCC

ATTGAATTGCTTTACTTCTCTGCATCTGTGATCAAGTTCCTCGAGGGCGCTTGGCTCCCG

ATCCTCCTCGCCCTCATCTTGGTAACTGTCATGTTTGTTTGGCACTACGCCACGATCAAG

AAATATGAATATGATCTGCACAACAAGGTTTCGCTGGATTGGCTGCTAGCCTTAGGCCCG

AGCCTTGGCATTGCTCGAGTCCCTGGCATTGGCATTGTGTTCAGCGATCTGACCTCGGGG

ATCCCTGCGAACTTCTCGCGTTTCGTCACCAACCTCCCTGCCTTCCACCGTGTGCTGGTT

TTCGTGTGTGTGAAAGCTGTGCCCGTCCCTTTCATCCCCCCGGCAGAGCGCTACCTCGTG

GGGCGCGTTGGACCTCCCTCCCACCGCTCCTACAGGTGCATCGTGCGATATGGCTACCGA

GATGTTCACCAGGATGTAGACTTCTTCGAGACCGAACTTGTCAGCAAGCTCGCTGATTTC

ATCCGCTACGACTGGTACAAGACGCACGGGATCATCACCTCACCAAACGACGACGATGGC

TCTCGTTCTGGCGCATCATCAAGCGAATACCGGTTAGCAGTGATAGGAACCATGGCTTTC

CGGCACACCGCCTTTGAAATCGAGGAAACCGCCCAACCCGAGAGCGTATCCTTCGGTTTC

CCCACCGCTGAGAGCCTGACCGACATCATCGAGATGCAACCAACCACTACAGCAGCAGAG

AAAAGGGTGAGATTTGCTGTTGATGACTCAGAACAAGACTCACACTCACACCAAGAAATG

GACATTCAGCTTCAAGAAGAGCTGAGATCTCCACGAAGCCCGAGAAGCGGGATCGCCTTC

ATTCTCGGGCACTCACATGTAAGAACAAAGCAAGGCTCATCCATATTCAAGAGATTAGCC

ATCAACTTTGGGTACAATCTCCTCAAGAGAAACTGCAGAGGACCAGATGTATCACTTAAA

GTCCCCCCAGTGTCCCTTCTTGAGGTTGGCATGGTATATGTTGTCTGA

>g25454.t1/IbHAK2

ATGGATCTTGAGAATGGCAAGTGTTGGGACACTGCAAAGAAGAGTTCGTGGAAGACGACG

TTGATTCTAGCATACCAAAGCCTCGGGGTGGTGTATGGGGATCTCAGTATCTCCCCTATC

TATGTCTACAAGAGCACATTTGCAGAAGACATTCATCACTCCGAGACGAATGAAGAAATC

TTTGGTGTTCTGTCGTTCGTTTTCTGGACTTTAACTCTTGTCCCTCTATTCAAGTATGTG

TTTATAGTGCTTCGAGCTGACGATAATGGAGAGGGTGGTACTTTTGCACTGTATTCTCTG

ATATGCAGGCATGCCAAAGTTAGCCTTCTCCCAAACCGACAGATTTCTGATGAAGCTCTT

TCCACTTATAAACTCGAGCCCCCACCCGAGGCCAAGAACAGCTCGAAGGTGAAAATGGTG

CTCGAAAAGCACAGATCACTGCACACTGCACTGCTAATCTTGGTTCTGCTTGGCACTTGT

ATGGTCATCGGAGATGGACTGCTTACTCCAGCCATATCGGTTTTCTCTGCAGTTTCAGGC

CTCGAGTTATCCATGTCTAAAGATCACCACCAGTATGCTGTGATTCCTATTACCTGTGTC

ATCCTAGTATGCCTGTTCGCATTACAACATTATGGGACACATCGAATTGGTTTTGCTTTC

GCCCCGGTTGTGCTTACTTGGCTGCTCTGCATCAGTGCTCTTGGATTGTATAACATCTTC

CACTGGAATCCCCATATATACAAGGCTCTTTCCCCTTATTACATGGTTAAGTTCTTGAAG

AAGACTAGACAAAGAGGGTGGATGTCATTGGGAGGAATTCTTCTCTGCATTACTGGATCT

GAAGCAATGTTTGCTGATCTTGGTCACTTCTCATATGCTGCAATTCAGATTGCTTTCACC

TTTCTGGTTTATCCAGCACTGATCTTAGCATACATGGGCCAAGCAGCTTTTCTATCAAGG

CATCATCACACCATTCACCAGATTGGTTTCTATGTATCAGTTCCAGAAAGCGTGAGGTGG

CCGGTGCTCGTGGTCGCCATTCTGGCGTCGGTCGTCGGCAGTCAGGCAATCATCAGTGGA

ACATTCTCAATCATCAACCAGAGCCAATCCCTGGGTTGTTTCCCAAGAGTCAAGGTGGTT

CACACCTCTGATGAAATACATGGGCAGGTTTACATCCCAGAGATCAACTGGATACTAATG

ATCCTTTGTGTTGCTGTGACCATTGGATTCAGAGACACAAAGCACATGGGGAATGCATCA

GGATTAGCAGTGATGGCAGTGATGCTGGTGACTACATGCCTCACTTCACTAGTCATCATC

CTCTGCTGGCACAAACCTCCCATCCTCGCTCTCTGCTTCCTCCTCCTCTTCGGCTCCATC

GAGCTTCTCTACTTCGCCGCCTCCCTCATCAAGTTCCTCGAAGGCGCCTGGCTCCCCATC

CTCCTCTCCCTCTTCCTCCTCACCGTCATGTTCGTCTGGCACTACGCCACCATCAAAAAA

TACGAATTCGATCTCCAGAACAAAGTCTCTCTCGAATGGCTCCTCGCCCTAGGCCCCAGC

CTCGGCATTTCTCGAGTCCCCGGAATCGGCCTCGTTTTCACCGACCTCACCTCCGGAATT

CCCGCTAACTTCTCCCGGTTCGTAACTAACCTCCCCGCTTTTCACGGAATTCTGGTATTC

GTCTGTATTAAATCGGTTCCGGTCCCGTTTGTCCCGCCCGCGGAACGGTATCTTGTTGGA

CGGGTGGGCCCCGCGTCTCACCGCTCGTATCGGTGTATTGTTCGGTACGGTTACCGGGAT

GTTCACCAGGACGTGGATTCATTCGAATCGGAGCTCGTGAGTATTCTGGCGGATTTTATC

CGCTACGATTGGTACAAATCTCACGGCGTTATCGACGACGTCGACGGCGACGCGCCGGCC

GGCGCGTCGCCGGGAGAATACCGGCTGGCCGTGATCGGAACCGTCGCGCTCTCCAGATCG

CCGGCGTTCGACGCCGACGACGAAAGCGTGCAGCCGGCGAGCGTATCCGCGGGGTTCCC
CACGGTGGAGAGCGTGACGGAGATGTTGCCGCCGCCGCCTGCGGCGGCGGCGGGGAAAA
GAGTGAGGTTTGCGGTGGACGAGGAATCTGCTTCGGAGGTGGATTCGCGGTCGGAAATGG
AGATTCAGATGCAATCGGAGCTGGAAGAGCTTTACGCGGCGCAACAGGCGGGGATAGCGT
TCATCCTCGGACATTCGCACGTGAGAACGAAACAGGGATCGTCTCTTTTCAAACGATTGGC
CATTAATTTCGGGTATAATTTTCTGAGGAGGAATTGCAGAGGCACGGATGTGTCGTTGAAG

GTCCCGCCGGCGTCTCTTCTTGAAGTTGGGATGGTTTATGTTGTTTGA

>g12038.t1/IbHAK18

ATGGAGAGGGGGGAGATAGAAGAGAGTATTACAGTGGGATTGTTGAGGAGGACTGGTACC

GGCGGGAGTGGGGAGTCGAGGTGGGTGGATGGGAGTGAGGTGGACTCAGAGTCACCTCC
ATGGTCAATACATGGGGACGAGGGGCTCAGAGAAGGGTACGGATCTGTTAGGAGAAGGCT
TGTGAAGAAGCCCAAGAGAGTCAATTCTTTCGATGTTGAAGCCATGGAGATTGTTGGTGAT

CATGCCCACCATAAGAAGGATCTTTCACTTGCGGGAACTCTTGCTCTAGCATTTCAAACG

CTTGGTGTGGTATATGGTGATGTGGGAACAAGCCCTTTGTATGTTTTTGCTGATGTGTTT

AGTAAGGTAACCATAACTTCAGAAATTGATGTCTTGGGGGCATTATCGATAGTGATATAT

ACAATTGCTCTCATTCCCCTCATGAAGTATGTATTTATAGTACTGAAGGCCAATGACAAT

GGAGAAGGAGGAACGTTTGCCCTGTATTCATTGATCTGCAGGTATGCAAATGTTAATCTT

CTCCCAAATCGCCAGCAAGCCGATGAAGGTATCTCCAGTTTTAAGCTCAAGCTGCCCACA

CCGGAACTGGAAAGGGCACTAAATATAAAGGATGCACTGGAACGTAAATCCTTTTTGAAG

ACCCTTCTCTTGCTCTTGGTCCTCACAGGAACATCAATGATTATAGGGGATGGTATCTTG

ACACCAGCAATGTCAGTGGTGTCTGCTGTGAGTGGTCTGCAGGGTAAGATACCCGGTTTT

GGAACAAATGCTCTTGTTATCACCTCGATAGTCATCCTTGGTGCATTGTTCAGCATACAG

CGATTTGGGTCAAGTAAAGTTGGCTTCACATTTGCTCCAGCCCTTGCTATCTGGTTCTTT

AGTTTGGGGTCCATTGGAATTTACAACCTGTTTAAGTATGATGTTTCTGTGGTAAGGGCA

TTGAATCCAGCTTATATCTTCCTTTTCTTCTCGAAGAATTCCAGTAAGGCATGGTCAGCT

CTCGGTGGCTGTGTTTTATGCATCACAGCTATTGATATCCAGTGCCGGGATGCAGGAGCC

GAAGCAATGTTTGCTGACTTAGGTCATTTCTCAGTGCGATCTATACAGATTGCGTTCACT

GGCGTGGTTTTCCCCTGCCTTCTCTTAGCCTACTTGGGCCAAGCTGCATATCTCATGAAA

CATCCAAATTCAGCCAGCACAGTATTTTATTCCTCTGTACCTGGTAAAAATGCTAGTTTT

CGTACAGATGGGTTTTTCTGGCCAATATTCGTGATAGCTACAGTTGCTGCTATAATTGCC

AGCCAGGCCATGATATCTGCTTCGTTTTCTTGCGTCAAGCAAGCCATGGCTCTCGGATGC

TTCCCACGGCTAAAGGTAGTTCACACCTCAAAAAGGCACATGGGTCAGATTTACATTCCC

GTAATCAATTGGTTTTTGATGCTCATGTGCATGGCTGTAGTTGCTGCTTTCAGAAGCACA

ACAGACATAGCAAATGCATACGGCATAGCTGAAGTTGGTGTGATGTTGGTGAGCACCACT

ATGGTTACGCTAGTGATGGTTTTGATATGGCAAACGAATCTGTTTCTGGCGTTGTGCTTC

CCACTTGTATTCGGGTCGATAGAGCTTCTGTACTTGTCTGCTGTACTGTCGAAAATCCTG

GAGGGCGGTTGGCTTCCGTTCGTCTTTGCTTCGTTCTTCCTGTCCGTGATGTACGTGTGG

AACTATGGGAGCGTGTTGAAGTACCAGGTCGAGGTGAAGCAGAAGATCTCGATGGATTTC

GTGTATGACCTCGGGTCTACCCTTGGAACTGTGCGAGTGCCGGGCATTGGACTGCTTTAC

AACGAGCTCGTCCAAGGTATCCCCTCGGTTTTCATGCAGTTCCTGCTCGACCTTCCAGCA

ATCCACTTGATCATCGTGTTTGTTTGCATCAAGTATGTCCCAGTGCCCGTCGTCCCTCAG

GAGGAACGGTTCTTGTTCCGAAGGGTCGGCCCGAAGGACTACCACATGTTCCGGTGTGTT

GCTCGATACGGTTACAAAGATGTGAGGAAGGAAGATCACCAAGCATTTGAGCAGCTCCTG

GTGGACAGCCTTGAGAAGTTTTTAAGGAAGGAGGCTCAGGATCTTGCCCTGGAGAGTGTC

AGCAGCGTGACACGTCAGACTGATGACGTGGCATCCACGAGGTCATCTGACGCGGGAGAT

GGCGGTGGGATAGAGGGTCTCAAGGTCCCACTGATTCAGCAGGACGAGGGTCGGAAGAA
GGCGTGGCGGATGATGAGTCAGCATTGCTATTGCCAGCTAGCGCCATGTCAGCGGACGAA
GATCCCAGCTTGGAGTACGAGCTGTCAGCCCTCCGCGAAGCCACCGAATCGGGTTTCACTT

ACTTGCTGGGGCACGCTGACGTGA

>g59493.t1/IbHAK7

ATGGACAATAACAACAAATTGGGTCCGGTGTACACTGTTCCAAATGGTTACATTTATTCC

AACGGCGCAGTTGCTAAAGTGACAGTTTACGACACCGGAAAAACAGTTCAGACTGATCTA

TTCATTCACAGCTGCAAAGCAAAACGTTTTGAGGTAAGTGACGAAGAGAAGGAAGTGAAG

TTTCAGAGGAGATGGCGGAGGAGGGATCGGAGAGGGAGAATGGTGAGCGGTTGGCGTCG
ATGGATTCGATCGAGTCGCGTTGGGTGTTTCAGGACGAGGAGGGGGTCGGAGATGGATAA
TGACGGCACTGATACCGGCGAAAGCGTCGATGAGTCGATGCAGCTGAACGGTATTGACTC
CGACGACGAGGACAACGCCGAGCAGCGGCTGATTCGGACTGGTCCTCGGATTGATTCTTT
CGACGTCGAAGCTCTCGAAGTTCCCGGTGTTCATAAGAATTACTATGAGGATGCTACTTTA

GGCAGGAGAATCATATTGATCATTCAGACACTTGGAGTAGTCTTTGGTGATGTGGGGACA

AGTCCATTATATACATTTAGTGTGATGTTTAGTAAAGCCCCAGTGAATGGAAATGAAGAT

GTTCTTGGTGCATTATCATTGGTTATATACACCTTAATCCTGATTCCATTGGTCAAATAT

GTCCTTATTGTTCTTTGGGCCAATGATGGTGGTGAAGGTGGTACATTTGCTCTATACTCA

TTAATTTGTAGGTATGCTAATGTTAGTCTTCTCCCTAATCAATTGCCATCTGATACTCGC

ATAACAAGCTTCAGACTCAAGGTGCCATCTCCAGAACTTGAGAGGTCTTTGAAAATAAAG

GAAAGACTTGAAACATCACTCACATTGAAAAAACTTCTTCTGATGTTAGTTCTTGCTGGC

ACCTCTATGGTGATAGCTAATGGTGTTGTTACACCAGCTATGTCAGTGGTATCTGCTGTT

GCGGGCTTAAAAGTTGGAGTTTCAATTGAGCAAGATCAAGTGGTCATGATTTCAGTGGCA

TTTCTTGTAATTTTATTCAGTGTACAGAGGTACATGACAAGTAAAGTGGGACTTGTAGTT

GGTCCTGCCTTATTTGTTTGGTTCTGTTCCCTTGGTGGCATTGGAATTTATAACATTGTG

AAGTATGATAACCGTGTGTGGAGAGCATTTAATCCAGTTCACATCTACTACTATTTCAAG

CGTAATTCTACTAAGGCCTGGTACTCACTTGGGGGCTGTCTTTTGTGTGCAACAGGTTCT

GAAGCAATGTTTGCTGATCTTTGCTACTTCTCTGTGCACTCAGTTCAGCTCACTTTTTTG

TTTCTTGTACTTCCATGCCTTCTACTGGGTTACCTCGGTCAAGCTGCTTACCTGATGGAT

AATCATTCTGATACAACACATGCTTTCTTTTCATCCGTGCCAACACTGATTGCCAGCAGG

GCAATGACGACAGCAACTTTTTCTTGTATTAAACAATCAACAGCTCTAGGTTGCTTCCCT

CGCCTTAAAATTGTTCACCCATCTAGGAAATTCATGGGTCAAATTTATATACCAGTCATG

AATTGGTTTCTGCTGGCATTGTCATTGGTGTTGATCTGCTCTATATCAAACATTTATGAG

ATTGGAAATGCTTATGGCATTGCTGAGCTTGGAATGATGATGATCACAACGATATTAGTA

ACCGTTGTTATGCTTCTAATATGGCAGATCAACATAATTATTGTGCTGAGCTTTGTTACC

ATTTTCTTGGGAGTGGAGTTAACATTCTTTTCATCTGTATTATGGAGTGTGGGAGATGGT

AGTTGGATCATATTAGTATTTGCAGTTGTCTTGTTTCTAGTAATGTACATATGGAATTAT

GGGAGCAAGCTGAAGTATGAAACTGAAGTAATGAAGAAGATGTCTATGGATTTATTGCGG

GAATTGGGTCCAAACCTAGGAACAGTAAGAGCTCCAGGAATTGGATTGCTTTACAATGAA

CTGGCAAAGGGTGTACCAGAGATATTTGGACATTTCCTTACCACTCTTCCTGCAGTGCAT

TCAATGATAATTTTTGTATGTATCAAATACGTTCCTGTCCCTGTTGTGCCTCAGAATGAA

AGGTTCTTATTCCGGCGAGTCTGCCCCAAAAGCTACCACATGTTCCGATGTATTGCTAGA

TACGGCTACAAGGATGCTCGAAAAGAAAACCACCAAACTTTTGAGCAGCTACTGATTGAG

AGTCTTGAGAAATTCATTTGCCGAGAAGCTCAGGAACGGTCGCTTGAAAGTGATGGTGAT

TCTGATTCTGAAGAAGAGAGCACCTTTTCAAGGGTCCTTGTAGCCCCCAATGGCAGTGTA

TATTCACTTGCAGTGCCTCTCTTGGCTGATTTTGAAGACAAGGGGAAACTGGTTGTTGAA

GAAAGCACCTCAAGTGATCCTAACATCTCTGATGCTGAGCAGAGTCTTGAAAAGGAACTC

TCCTTCCTACGCACAGCCAAAGAATCCGGTGTAGTTTACCTTCTTGGTCATGCTGACATT

CGGGCAAGGAAGGATTCCTGGTTCGTCAAGAAGCTTATCATAAATTATCTATACGCCTTC

CTGAAAAAGAACTCGAGGAGGGGAATATGCAATTTGAGCGTGCCCCATTCGCACGTAATC

CAGGTTGGTATGACTTACATGGTGTAA

>g1704.t1/IbHAK9

ATGGTATCTGCAATCTACAATTCGGTACATAGGAATATCGGGATAGATATGGATCTTGAA

CATCGGAAGTGTTGGGACACCACAAAGAAGAATTCTTGGAAGACGACACTGATCCTAGCT

TATCAAAGTCTTGGTGTCGTTTACGGGGATCTCAGCATTTCCCCGCTCTATGTCTTCAAG

AGCACATTTGCGGAAGACATTCAGCACTCGGATACTAATGAAGAAATTTTCGGGGTTATG

TCGTTTGTTTTCTGGACTTTAACTCTTGTCCCGTTATTCAAGTATGTGTTTATAGTCCTC

CGAGCGGATGACAATGGAGAGGGTGGTACCTTCGCTCTTTATTCCCTGATATGCAGGCAT

GCCAAAGTAAGCCTTCTTCCAAACAGACAAGTTTCCGATGAAGCCCTTTCCACGTATAAA

CGCGAGGAACCTCCCGAGAGAAACAATAGGGTTTCGAGGGTGAAGTTGGTGCTCGAGAAG

TACAAATTCCTGCACACGGCATTGCTAATCTTGGTGCTCCTTGGCACTTGTATGGTCATC

GGAGATGGACTGCTTACTCCCGCCATATCCGTTTTCTCTGCGGTTTCTGGGCTCGAGTTC

TACATGTCCAAGGATCACCACCAATATGCGGTGGTTCCTATAACTTGCGTCATATTAGTC

TGCCTATTCGCGTTACAGCATTATGGCACACACCGCATTGGCTTTTGTTTTGCGCCAATC

GTGTTATCTTGGTTAATCTGCATAAGTGGGCTCGGATTGTACAATATCCTCACTTGGAAT

CCACATGTTTACAAGGCGCTTTCCCCGTATTACATGTTCAAGTTCTTGAAGAAGACTAGA

AAAGATGGATGGATGTCGTTGGGAGGAATTCTCTTGTGCATAACAGGATCTGAAGCAATG

TTTGCTGACCTCGGCCACTTCTCGTATGCTGCAATTCAGACCGCATTCACCTTTCTGGTT

TATCCAGCGCTTATATTGGCATATATGGGCCAAGCAGCTTTTTTGTCGAGGCATCATCAC

AACATGCACGAGATTAGTTTCTATTTGTCTGTTCCAGGAAGTGTAAGGTGGCCGGTTCTT

ATTATAGCCATTCTTGCTTCTGTCGTTGGCAGTCAGGCAATCATCAGCGGAACATTCTCG

ATTATCAACCAGAGTCAATCACTAGGTTGTTTTCCAAGAGTTAAGGTTGTTCACACCTCT

GCTAAAGTACATGGGCAGATCTACATCCCGGAGATAAATTGGATTCTCATGATCCTTTGT

GTTGCTGTGACTATCGGGTTCCGGGACACGAAACATTTGGGAAATGCATCAGGGTTAGCG

TTGATGGCAGTCATGCTGGTGACTACCTGTCTAACCTCGTTGGTTATCGTCCTGTGTTGG

CACAAGCCTCCAATTCTTGCGCTTGTCTTTCTCGTTTTCTTTGGATCCATTGAATTGCTT

TACTTCTCTGCATCTGTGATCAAGTTCCTCGAGGGCGCTTGGCTCCCGATCCTCCTCGCC

CTCATCTTGGTAACTGTCATTGATTTGACCTCGGGGATCCCTGCTAACTTCTCGCGTTTC

GTCACCAACCTCCCTGCCTTCCACCGTGTGCTGGTTTTCGTGTGTGTGAAAGCTGTGCCC

GTCCCTTTCATCCCCCCGGCAGAGCGGTACCTCGTGGGGCCCGTTGGACCTCCCTCCCAC

CGCTCCTACAGGTGCATCGTGCGATATGGCTACCGGGACGTTCACCAGGATGTAGACTTC

TTTGAGACCGAACTTGTCAGCAAGCTTGCTGATTTCATCCGCTACGACTGGTACAAAACG

CACGGGATCATCACCTCACCAAACGACGACGATGGCTCTCGTTCCGGTGCATCATCAAGC

GAATACCGGTTAGCAGTGATAGGAACCATGGCTTTCCCGGGCACACCCGCCTTTGAAATC

GAGGAAACCGCCCAACCCGAGAGCGTATCCTTCGGTTTCCCCACCGCAGAGAGCCTAACC

GACATCATCGAGATGCAACCAACCTCTACAGCAGCAGAGAAAAGGGTGAGATTTGCTGTT

GATGACTCAGAACAAGACTCACACTCACACCAAGAAATGGACATTCAGCTTCAAGAAGAG

CTGAGAGATCTCCACGAAGCCCGAGAAGCCGGGATCGCCTTCATTCTCGGGCACTCACAT

GTAAGAACAAAGCAAGGCTCATCCATATTCAAGAGATTAGCCATCAACTTTGGGTACAAT

CTCCTGAAGAGAAACTGCAGAGGACCAGATGTATCACTTAAAGTCCCCCCAGTGTCCCTT

CTTGAGGTTGGCATGGTATATGTTGTCTGA

>g34971.t1/IbHAK12

ATGGAATTTGATGATGACTCCAGTGAAACTAAGGGAGGGATGTGGGTTTTGGACCAAAAA

CTTGATCAACCTATGGAAGAGGAGGCTGGAAGGTTAAAAACCATGTACAGAGAAAAGAAG

TTCTCCTCGATTTTGCTATTGCAGCTTGCGTTTCAGAGCCTGGGAGTGGTGTATGGGGAC

TTGGGGACATCACCGTTGTATGTTTTCTACAATACATTTCCCCATGGGATTGAAGACACG

GAGGATATCATCGGAGCACTTTCATTGATCATCTACTCGCTCACCCTTATACCGCTCCTC

AAATATGTTTTCATAGTGTGTAGAGCGAGTGACAATGGCCAAGGTGGGACCTTTGCTCTT

TATTCGTTACTCTGTCGACATGCTAAGATAAAAACCATTCCCAATCAACACCGGACAGAT

GAGGAGCTAACGACTTATAGCCGTAACACATTTCATGAGCAATCATTTGCTGCAAAAACA

AAGAGATGGCTGGAAGCATATGCATTCAGAAAAAATGCGCTGCTTATTCTCGTCCTTGTT

GGGACTTGCATGGTAATTGGGGACGGAATTCTTACTCCTGCTATATCAGTTCTTTCGGCT

TCGGGTGGAATCCAGGTGGAGCATCCAAAGATGAGCACTGATATAGTTGTGCTTGTTGCG

GTCCTTATACTAGTTGGAGGTATAGGCATTTTCAACATCTGGAAATACGATAGCTCGGTT

TTGAAGGCCTTTTCGCCTGTATACATATATCGGTATTTCAGGAGGGGAAGGAAAGATGCC

TGGACATCACTCGGAGGAATCATGTTAAGCATTACAGGTACCGAGGCACTTTTTGCTGAT

CTTTCTCATTTTCCTCTGGCTGCAATTCAGCTTGCTTTCACAGTTATCGTTTTCCCCTGC

CTTCTTTTGGCCTACTGTGGCCAAGCATCCTACCTCACTCAACATCGAGATCACGTGTCC

GATGCATTCTACCGTTCTATTCCAGATAGCATATATTGGCCTGTCTTTATTGTTGCTACT

CTAGCTGCTATTGTCGCGAGTCAAGCCACCATATCTGCTACGTTTTCGATAATCAAGCAG

GCTCTAGCATTAGGCTGTTTTCCGAGAGTTAAGGTCGTGCATACATCGAAGAAGTTCCTT

GGACAGATATATATTCCAGATATCAATTGGATCCTCATGGTTCTTTGCATTGCTGTGACG

GCTGGATTCAGAGATAAGAGTCAAATTGGCAACGCTTACGGCACAGCGGTCGTGATAGTC

ATGCTGGTCACCACGCTTCTCATGATCTTGATAATGTTGCTAGTCTGGCACTGTCACTGG

ATCATCGTCGTTATCTTCACGGGATTATCTCTTGTGGTGGAATGCACTTACTTCTCGGCA

GTTCTTTTCAAGGTTGACCAGGGCGGTTGGGTCCCGCTTGTGATTGCTGCAGCTTTTCTG

ATAATCATGTATGTTTGGCACTATGGCACGGTGAAGCGCTATGAATTCGAGTTGCACAGC

AAGGTCTCGATGGCTTGGATTCTAGGGCTCGGCCCGAGCTTAGGCCTAGTTCGTGTTCCG

GGGATAGCTACACATTCAGTAGTGGTATTTGTGTGTGTGAAGTATCTGCCGGTCTATACG

GTCCCGGAAGACGAGAGATTCCTCGTGAAGAGGATAGGACCGAAGAACTTTCACATGTTC

CGCTGTGTTGCAAGGTATGGTTACAAAGACCTCCACAAGAAAGACGATGATTTCGAGAAG

AAACTGTTTGACAACCTCTTCACATTTGTTCGCCTCGAGTCTATGATGGAAGGCTGCTCG

GATTCTGACGAGTACAGCGTGTATGGGCAGCAAACGCAGCAATCGAGGGATTTCCTTCTG

GCGAAGAACAATGGCAACACGTGCACGAGCACAGACCTCACGGTGTCCTCGGTGGACTCC

ATCGTGCCCGTGCAGTCTCCTAGGCACGGGTGCAACATGACCATGTCGTCGGGGCGTGAC

AGCACGCAGACAGAAGTCGACGAGCTGGAGTTCTTGAACGCCTGTAGAGATGCCGGGGTT

GTACACATTCTTGGGAACACAGTAGTGAGAGCAGGGAGAGATTCTCGGTTTTATAGGAAA

ATCGCCATCGACTATGTATATGCATTTCTTAGGAAAATTTGCAGGGAAAATAGTGCAATT

TTCAATGTGCCTCATGAGAGCCTCTTGAACGTTGGACAGATTATCTATTTGTCAATTGCA

TCTGGTTGTGCTGTTCTTCTGTGCTCTGCTAATACTCTCATGACAGATCACAAAATCAAC

TAG

>g5020.t1/IbHAK1

ATGTCACCAACAAGCTCAGCCATGAACCCAACACCTTCACCAGAATGTAGAGGACAAGGG

ATGGCTTCCAAACGGAATCTGAACAAGGTACCTTGTTCAACCGTTCTCACCTTGGCTTAT

CAGAGTCTCGGAGTAGTTTATGGCGACCTGAGTACTTCTCCTCTTTATGTATATAAGACC

ATCTTTTCTGGAAAGCTAAACCTTAAGGAGGATGATGAAGAAATATACGGGGTGCTTTCC

TTCATCTTCTGGACATTTACAATAATCGCGCTATTCAAATATATTTTCATCGTATTGTCA

GCTGATGATAACGGGGAAGGTGGCACTTTTGCGCTGTACTCCCTACTGTGCAGGCATGCG

AGACTCAGCATGCTTCCCAATCAACAAGACACGGATCAGAAGTTGTCTGCATATGCCATA

GAAGGATCGGAGGACACTAGGCAAACTGCGGCTTTGAAGTCTTTCTTTGAAAGATACCCG

AGCTTTCGTAATGGACTTCTGATCTTTGTTTTGCTGGGAACCTGCATGGCTATAGGCGAT

GGTATATTTACCCCAACCATTTCAGTTCTTTCAGCTGTTTCGGGCGTGCAAGTTAAGCTA

AAAGGGCTGCATGAGAATTATATTGTTGTGATTTCATGTATCATTCTTGTCGCACTCTTC

TCCATTCAGCATCACGGAACACATAGAGCGCTTTCTCCATTCTACATGTTGAGGTTTCTT

AAAGTAACGGGTTGGGAAGGTTTTGTCTCCTTAGGTGGAATAGTGCTCTCAATAACAGGC

GTGGAGACGATGTTTGCTGATTTGGGCCATTTTTCCACATTGTCGGTCAAGATTGCCTTC

ACGTCTTTGGTGTATCCCTGTTTGATGCTCGCATATTTGGGCGAGGCTGCATTTCTCTCT

AGACACCACGAGGATATCCAAAGAAGTTTCTACAAAGCCATACCGGAGCCGATATTCTGG

CCAGTGTTTTTAGTGGCCACTTTGGCAGCTGTTGTAGGAAGTCAGGCTGTAATATCGGCT

ACTTTCTCAATCATAAGCCAGTGTTCTGCACTGCATTGCTTTCCCCGTGTGAAGATAGTA

CATACTTCCAGCAAGATATACGGGCAAATTTACATCCCGGAAATCAACTGGATATTGATG

TGCCTCTGTTTAGCTGTTACTATCGGATTAAGGGACACGAACAAGATTGGAGCTGCTTAT

GGCTTGACGGTCACAACTGTTATGTTCGTGACAACTTGCTTGATGGCAATGGTGATGGTC

GTCGTGTGGAAGAAGAAGATACTAACAGCCGCTGCTTTTGTTGCATTCTTTGGATCAGTG

GAACTCCTCTATCTGTCAGCAGCGATCTATAAGATTCGAGAAGGAGCTTGGATACCCCTT

CTTCTGTCGATTATCTTTATGGGCATAATGTTTATATGGAACTATGGAACTCTGAAGAAA

CATCAATTCGATCTGGAGAACAAGGTCTCGATGGAAAGAATTTTAGCGCTGGGGCCTGGC

CTTGGAATAGTTCGAGTCCCTGGAATCGGACTTATTTACACCAATCTGGTCACAGGCATT

CCAGCCATTTTTGGGCACTTTGTTACCAACTTGCCTGCTTTTCATGAAGTCCTTGTTTTT

GTCTGTGTGAAATCTGTTCAGGTGCCATACATTAGCGAAGAAGAGAGATTCTTGATATGC

AGAGTTGGTCCAAAGGAGTTCAAAGAAACAAAGCCAACACAGACTTTGGCTTCTGGGAAC

TCAGATCGTCTGGTTCTTGAAGCCCCAGAGTACAGACTCTCCAACCTTAGTTACACAGCA

CAGTCTACACATGACATCCGCGTGATAATATCAGGGGAGAGGAATGCAGAAACCTGCCCT

GGTATTCAAGATGAATCCCGGGATATAATGAGGGCGAGGGAGTCAGGAGTTGCGTATATC

TTTGGGCATTCTTATGCCAAGGCAAAGAAATCATCTTCCATCATCAAGAAATTCGCCATC

AACATAGTGTATGCTTTCCTGAGCAAGAACTCTCGAGGACCTGATGTGGTTCTGAATGTC

CCTCACACTTCTTTACTGGAAGTAGGCATGTTGGTTCTCATATATCTTTTGAGATTTTAT

AGTTTGATAAGTAGAATCCACTGA

>g20629.t1/IbHAK4

ATGGAAGTCTCCAAACCCTTTTCTCCTGTGTCTGATAAGAATGACCCTGAGAATGGCCGC

CGGTTCAGTGGTCGGCGTTGCTTCTCCGACAGTCAGTTGGTCTTTCCGGCGGCGACCCCG

ACCGGAGATACTATGATGAGTTTCTATCAACCGTACAGGTCTCATCAGCTCAAGGCAGGA

ATATCCTATTCCTATCCACCCATGGAGACGCTACTCCTGGCCTACCAATCCCTCGGAGTC

GTGTACGGCGACCTCGGCACATCCCCGGTGAACGTTTTCTCGGCCACCCAACTCATCAAT

CTTAGCGAGGAAGACCTGCTAGGCACACTAAGCCTCATCTTCTGGGCCCTAACAATTCTG

GTCTTGATTAAATACACCTTCATCGTTATCCATGCAGACGATCATGGCGAGGGCGGCACC

TTCGCCCTGTATTCGCAGCTGTGTCAGCACATAAACATTCGGAGCAAGCTCGCCATACAG

AGCTGCATGAAGGGCGAACCAGACGACGGCCCAGTGTATTATGGCGGATCAGGAGGGCA

GCTGCGGGCTAAGACTAAGAAGTTTATAGAGAACAGCCCGAGGACTCAGAATTTTCTTACG

TTTATTGTGCTGCTGGGGACTTGCATGGTGATTGGGGATGGAACTCTCACACCAGCCACT

TGTGTTATTTCAGCCCTTCAGGGAATTCAATCTGTCTCCCCAAAGATAACTCAAGATCAT

GTTGTGTTCATGTCTGCGGTTCTTCTAATCGTCCTCTTTGCTTTCCAGCATTGCGGAACC

AGTAAAGTTAGCTTCGCCTTCTCCCCGATAATGCTATTGTGGTTTGCCACTAATACATCT

ATTGGCATCTACAATATCATAAAGTACAATCCATCCATTCTCAGAGCAATCTCTCCTCAC

TATATGGTGAAATTCTTTATAAGAAACCACAAGACCGCGTGGGATCTTCTTGGCGCTGTG

TTCCTGGGCATAACAGGAGCAGAGGCCATGTTTGCAGACTTAGGTCATTTCAACAAAAGA

GCAATTCAGTTGGGTTTTTGTTTCGTTGTCTACCCATCGCTCGTTATCACCTATGCTGGG

GAAACGGCTTACTTAGTCAGGTACCCGGAGAAGATCACAAACGCCTATTACAGCTCTATC

CCTAAGGCTGTATACTGGCCAATGTTTGGAATCTCTACCCTAGCTGCCATAGTTGCAAGC

CAATCTATGATATCGGCTTGTTTTTCCATCGTGAAACAGTCCCTGTCTCTCGAGTGTTTC

CCGAGGGTGAAGATTGTCCACACTTCTTCCAAGCATGAAGGGCAGGTCTACTGTCCAGAA

ATAAACTACACCCTAATGGTTCTCTGTGTTGCTCTAGTTATCGGCTTCAAGGGCGGTGTT

GAACTGGCAAATGCTTATGGTGTGGTTGTGATCTGGGTTATGATTATAACTACTTTCTTG

ACAGCCCTAGTCATGTTGCTCATCTGGAACACAAATATTCTTCTAATTCTAGGGTTTTTC

TTGCCCTACATACTGATTGAAGGCTTCTTTATGACCTCCCTGCTACGTAAAATACCACAA

GGGGGATGGGTACCATTTGCAATCTCTGTTATACTATTTGCAGTCATGATCTCTTGGACC

TATGGAAGCAGTAAGAAGAAAGAGTATGAAGCTGAAAGGAAGATGGCCCTAACAGAATTA

GATGAAATGTTATCAGTCAGTTATATCTATAGAACACCGGGAACTTGTTTCTTCTTTACT

GATCTTGTCAATGGGATTCCGCCCATTGTACGACACTACATTCAGCACACAGGATCTATT

CAAGAAGTCATGGTGATTGTCACGGTTAGGACTGTTCCAATCAAAACCGTGCCCCCTGAG

GAGCGTTTCAGTGTACAGAAGCTAGGAGTGAAGGGGGCTTACAGATGTTTGGTTCAGTTT

GGGTACAAGGATTCTCAGAATGTTGGGGGAGAAGAATGTGTTAGTTCAATGATTGCAAGG

CTCCAGGAGGCAGCTGAGTCGAGTGGCGAAAAACAGAAAATAAGTTCAGCCACACAGAG
AGGGGTCATTTTTGTGGTAGGCAGAACCATTCTAAAAGCAAATGGGCAGAATGGCAGGTTT

TCTAGGTTTACAATAAACTATTTGTACAGGTTTCTGCAAAAGAACTGCAGAGGGGCCATT

TCAACAGCTGAATTCCCTCTCGACAAAACATTGCAGGTAGGAATGCTTTATGAAATCTGA

>g12041.t1/IbHAK6

ATGGATCTAGAGTCTGGGTCTTGCCAGAATCGTGCAAAGAGGGAGTCTTGGAGGCAAGTT

TTGATATTGGCATATCAAAGCTTAGGTGTTGTCTATGGTGATTTAAGCACATCACCATTG

TATGTGTACAAGAGTACTTTTGCTGAGGACATTGAGCACTCAAGAAGCAATGATGAAATC

TATGGGGGGGGCACATTTGCTCTTTATTCACTCCTCTGTAGGCATGCTAAGGTGAACTCC

TTGCCCAGTTGCCAGTTAGCAGACGAGGAATTATCCTCGTTTAAGAAGGACATAATCAAC

CCTGCCCCTGCAACTCTTGGGGGGAGGCTGAAATCTAGTCTGGAGAGGTATAGAGTATTG

CAGAGATTTCTCCTTGTGTTGGCTTTAATTGGAGCGTGTATGGTCATAGGCGATGGTATT

CTTTCACCGGCCCTCTCGGTTTTTTCAGCAGTTTCTGGTATTGAACTTGCAATGGCTAAA

GAGCACCACAAATATATAGAGGTTCCCGTTGCTTGTGTCATCCTGATCGTCTTGTTTTCC

CTTCAACATTATGGCACTCACAGGGTTGGATTTTTGTTCGCTCCTGTGGTCATAACATGG

CTTCTATGTATCAGTGCTATTGGTTTATATAATATTTTTCGTTGGAATCCTTTTGTGTAT

CAAGCACTATCACCATATTACATGTACAAATTCCTGAAGAAAACTCAAAGAGGAGGCTGG

ATGTCGTTGGGAGGGATTCTCTTATGCATAACGGGTTCAGAGGCGATGTTTGCTGATCTC

GGGCACTTCACTCAAATGTCTATACAGATTGCTTTCACATTCATGGTTTATCCATCGCTT

GTCCTTGCATATATGGGGCAAGCTGCTTATCTGACCAAGCATCATGATTTTGAGAGTGGC

TACCATATTAGCTTCTACATTTCAGTTCCCGAGAAATTAAGATGGCCTGTTTTGGTGATT

GCTGTACTAGCTGCAGTTGTGGGGAGCCAAGCTATAATCACGGGAACCTTTTCAATTATT

AAACAATGCTCTTCTCTGGGATGCTTTCCGAGAGTGAAAATATTGCACACATCGTCAAGA

ATGCATGGTCAGATTTACATTCCAGAGATCAACTGGACACTGATGCTGCTATGTTTGGCC

GTTACTATTGGTTTCCGAGACACCAAGCGCCTGGGTAATGCATCAGGTTTGGCTGTGATA

ACTGTTATGCTGGTTACAACTTGTTTGATGTCATTGGTGATTGTGCTGTGCTGGCACCGG

AGTGTCGTCCTTGCAATTGGCTTCGTGCTCTTCTTTGGTACAATGGAAGCATTTTATTTC

TCGGCTTCTCTAATTAAATTTTGCGAAGGAGCGTGGGTGCCCATTGCCCTGTCCTTCCTT

TTCATGATAATCATGTGTGTTTGGCACTACGGAACCATCAAAAAGTACGAGTTTGATGTC

CAAAACAAGGTCTCCATCGACTGGCTACTTAGCGTGGGTCCTAGCCTGGGGATTGTCCTG

GTTTTCCTTTGTGTCAAGTATGTCCAAGTTCCTCATGTTAGGCCCGAAGAGCGGTTCCTC

GTGGGGCGTGTTGGCCCCCGAGAGTACAGAATGTACCGCTGCATTGTCAGGTACGGGTAC

AGAGACGCCCACAAGGATGACCTAGAGTTCGAAAATGACCTCATCTGCAGCATAGCCGAG

TACATCCGGGCAGGAGACCCCACGAAGGACGACGAGGAAATGGCGGTGGTAGGCTCCCC
CTCGACTCACTTAAACCGAGTCCAAGTCTTCCAGGAGGACGGTCATCACGAAGGCGACCC
CTCGTCATCATCCACCGGGATATTAGAGCTCCGAGAAATAAAGTCTCCAAGAAAACGGGTG

AGGTTCGTCGTCCCCGAGTCTCCAAATATCAACGCAAACGCGCGGGGAGAGATGCGGGAC

CTCATGGAAGCGAGGGAGTCAGGTATAGCTTACATACTAGGGCATTCATATATGAAAGCA

AAGAATGGATCAGGACTTACAAAGAAGATGGCTATAAATCTTGGATATGAGTTCTTGAGA

AGGAACAGTAGAGCACCAACATATGCATTCAGTGTTCCTCATGCTTCAACTCTAGAAGTA

GGAATGGTGTACAATGTTTGA

>g42476.t1/IbHAK3

ATGTATCAAGTTGACATTAATCGTCAAGAAGATGGTGCAGAACTGCCTCCTCCACTTGCA

GCAAGTGCGAGTGAGGTTTCATCCCATGTAGGTGGACGAGAAGGCAGTGCAGTTCATAGA

AAAAAGCAATCAAAGAGTTTTGGGGTCGTATATGGGGATTTGAGCACATCACCTCTTTAT

GTCTATAAAAGTATATTTGTGGACAAGTTGCAGAAGCATCAAACACCAGATGTAATATTT

GGTGCCTTCTCATTGGTCTTTTGGACAATAACATTGCTGCCTTTGCTCAAGTATGTAGTT

GTGGTGCTGAGTGCAGATGACAATGGTGAAGGTGGCAGCTTTGCTTTATACTCTCTGCTC

TGTAGACATGCAAAGTTTAGTCTCCTTCCCAATCAACAAGCTGCTGATGAGGAACTATCT

GCTTACAAATATGGCTCCACTGGGCATTCTGCATCATCTTTATCATTGAAGAGATTTCTA

GAGAGGCATAAAAAGGCACGGACAGTTCTACTCATCATTGTATTACTAGGGGCTTGCATG

CTCATTGGTGATGGTGTGCTTACTCCTCCTATATCAGTTTTATCAGCGATTTCTGGGATT

CAAGATACTGCTGAAAGCCTGCCTAATGGTAGATTCAACTTACCTTCTCTTTTTGTGATG

GCTCTAAGTGCAGTTGTATTGATTACTTGTGTTATATTGGTTGGTCTATTTGCTCTTCAG

CATGTTGGTACCCACAGAGTAGCTTTCTTGTTTGCACCAATAGTAATAATCTGGTTAATA

TCTATTTTTGCAATTGGGCTGTACAATACAATTGTCTGGAATCCAAATATTCTCTCTGCT

CTTTCTCCCTATTATATCGTCAAGTTCTTTCGGGAGACTGGAAAAGATGGTTGGATTTCA

CTCGGGGGAGTGCTCCTATCTGTAACAGGTACCGAAGCTATGTTCGCGGATCTTGGTCAT

TTTAATTCCTTCTCAATGAGGCTTGCATTTGTATTTGTTGTGTATCCTTGCTTAGTGGTA

CAATACATGGGTCAGGCTGCTTTTCTGTCTAAAAACATTCCCTCAATCTCAAATAGTTTC

TATGATTCTGTCCCCGGTGGTGCACATTGGCCTGTTTTTGTTATTGCTACCCTTGCAAGT

ATTGTTGGCAGTCAATCAATTATCACAGCTACATTCTCCATTGTCAAGCAATGTAATGCA

CTTGGATGCTTCCCAAGGGTCAAGATTGTCCACACGTCAAGGCATATTTATGGCCAGATT

TATATACCGGAAATAAATTGGATACTAATGATTCTTACTCTGGCAGTGGCCATTGGCTTC

CGTGATACAACTTTAATTGGAAATGCATATGAAGGTTTATACCTGTCGGCATCAGTCATT

AAAGTTCCACAGGGTGGATGGGTATCACTTGTTCTCTCTCTTGTATTCTTGTTTGTTATG

TTCGTCTGGCACTATGGAACTCGCAAGAAGTACAGCTTTGATCTTCACAACAAAGTTCCA

CTAAGATGGCTACTTGGCTTGGGTCCCAGCCTTGGTATCGTTCGTGTGCCAGGGATAGGC

TTGATATATTCAGAATTGGCAACAGGAGTCCCTTCAATTTTCTCACATTTTGTAACTAAC

CTTCCTGCATTTCATAGTGTGCTTGTATTTGTGTGTGTGAAGTCTGTTCCCGTGCCCTAT

GTTGCACCCGAGGAAAGGTTCCTTATTGGCCGCATTTGCCCTAGACCCTATCGCATGTAT

CGGTGCATTATCAGATATGGCTACAAGGATATACAGCGAAACGATGGAGACTTTGAGGAA

CAGCTGATACAAAGTATAGCGGAGTTCATTCAAATGGAAGCAGTAGAACCTCAGCTATCA

AGCTCTGAGAGTTTATCGTTTGATGGGAGGATGGCAGTTATAAGCACCAAGAGTATACAG

TCAAGTTCAAGTTTGATTGTACGAGATGATGATTTGGGTTCGAGTTATTCCATTCAAAGC

AGCAAATCCCCAACCCTCCAGAGTCTAAGATCAGCTTATGATGACGAGAACATGCAAGTC

AGGAGACGCCGTGTTAGGTTTCAATTAGCAGACACCCCCGGGATGGATCCTGCAGTTAGG

GAAGAGCTTACAGACTTGATTCAGGCAAAGGAAGCAGGGGCTGCATATATATTGGGCCAC

TCTTATGTGAAGGCGAGGAGATCATCATCGTTTCTGAAAAAGCTTGTTATCGATATTGCA

TATTCATTTCTGCGCAAGAATTGCAGGGGTCCTTCTGTCGCACTAAACATTCCACACATT

AGCCTCATCGAAGTTGGGATGATATATTATGTGTAG

>g30149.t1/IbHAK10

ATGACTGCTCAACTACCGCATTGGGCATCCCCACACGGCTCTCCTCTTCAGATTGGTGTC

AGGAATGCGTTTCTCCTGATGATATTGAGGCGACCCGTGATGGATATTGAAGGTTGGGGC

CGTGGCAATCCTGTCAAGAAGGAGTCTTGGAGGACAGTCTTGGCTTTAGCTTATCAGAGC

TTGGGAGTGGTGTATGGTGATTTGGCCACTTCACCTTTGTATGTGTACAAGAGCACTTTT

GCAGAGGACATTCACCATTCAGAGACTAATGAGGAGATATTTGGAGTTTTGTCATTTGTG

TTCTGGACATTGACTTTAATCCCACTTCTGAAATATGTGTTTATAGTGCTGAGAGCTGAT

GATAATGGGGAAGGTGGGACTTTTGCCTTGTACTCCCTGCTGTGCCGCCATGCCCGGGTG

AGCACTTTGCCCAATGGCCAGGTTGCTGATGAGGACCTATTTGAGTACAAGAAAGATGGG

ATTCATTCTGCAAATAAGGGTCTTGGGTTGAGCTTGAGATCCACCCTGGAGAAACATCAA

TGGCTGAGGAGAATTTTGCTGGTTTTGGCACTGATTGGAACTTGTATGGTCATTGGGGAT

GGAGTCTTGACACCTGCAATTTCTGTGTTTTCTGCTGTTTCTGGGTTAGAGCTTTCCATG

TCAAAACATCATCATGAATCTAATCTATTAGCTATTTACTCGTTTCTCAGTCAAGTTTGT

CTTAACGCTATGGAGGTTGTACCTGGACTAGATGAATATGTGGAAGTTCCGGTTGCATGT

GTCATTCTGGTTTTCATATTTTCCATCCAGCATTACGGGACCCATCGTATAGGATTCCTG

TTCGCGCCAATTGTGGTCACATGGCTTTTATGCATCAGTGCCATTGGACTGTATAACATT

TTCCGGTGGAATCCTCATGTTTACCAAGCACTGTCTCCATACTACATGTACAAATTCTTG

AAGAAGACCCAACGAGGTGGTTGGATGTCTTTGGGCGGGGATTTTGTTATGTATTACAGC

ATCTTATCATTCTGTTTACGGAACTGCATCAAGCACAGACTTTCCACCGAGATAGCTTTC

ACCTTTGCGGTTTATCCATCATTGATTCTGGCATATATGGGGCAAGCGGCGTATCTTTCC

AAGCATCACACCCTTGAAACTGACTATCGGATTGGATTCTACGTATCAGTTCCTGAAAAA

CTTCGGTGGCCTGTTCTTGGCATTGCCATACTTGCAGCCGTGGTGGGAAGCCAAGCCATC

ATCACCGGGACGTTTTCTATTATCAAGCAATGCTCGGCTTTAGGTTGTTTCCCGAGGGTG

AAGATAACACACACATCGTCGAAATTCCATGGCCAGATTTACATTCCAGAAATCAACTGG

ACCTTGATGGTCCTATGCTTGGCCGTCACTATTGGTTTTGAAGATACAAAACACATAAGC

AATGCATCAGGTTTGGCGGTTATAACTGTTATGCTGGTAACGACGTGCCTGATGTCTCTG

GTAATCGTCCTGTGCTGGCACAAAAGTGTGTTTCTAGCCATTTTCTTTATCTTCTTCTTC

GGGACGATTGAGGCGCTCTACTTCTCAGCTTCGCTGATTAAGTTCCTTGATGGTGCCTGG

GTTCCAGTTGCTCTATCGTTCATCTTCCTGCTTATTATGTACATTTGGCACTATGGCACG

CTGAAGAAATATGAGTTTGATGTTCAGAATAGAGTTCCAATCAACTGGCTGCTGCAATTG

AGCCCTAATCTCGGCATTGCTAGGGTGCGAGGAATTGGCCTCATTCACACTGAGCTTGTT

TCTGGAATTCCTGCAATTTTCTCTCACTTCGTCACCAATCTCCCGGCTTTCCACCAGGTT

CTCATCTTCCTCTGCATCAAATCAGTCCCGGTGCCGCATGTGAGGCCGGAGGAGAGTTCG

AGAAGGATCTACTTTGCAGTATCGAACAATTCATCCGATCGGAAACCCCCTCCAGAAGCT

CCGGAACGCGGTGACCTAATCGATTTCGACGACGACGAAAGGATGACCGTCATCGGAACC

ACTTCCACATACCTAGACGGCAAAACAATGAGCGAGGACGGCGAAAACATGTCCGCAACC

GCCACAAGCACGACGGAGCTCCGAGAGATCTCCTCCCCGGAGATGATGACCGTGACCGGA

CGGCGAGTGCGATTCCTGGTGCCGGAGAGTCCGCAGATGGATCCAAGCGTGCGAGCGGA
ACTGCGTGATCTGATGGAAGCAAGAGAAGCAGGCATGGCGTTCATACTGGGACGCTGCTA
CGTCAAGGCTAAAAGCGGTTCCAGTTTCATGAAGAAAGTAGTCATCGATTTAGGTTACGAT

TTCTTGAGGAGGAATTCTCGAGGGCCAACATATGCTCTCAGCTTCCCTCGCGCATCAACT

TTAGAAGTTGGCATGATCTACCTTCAAGCTTCCATGCGTAAACTAAACAAGACCCAAGGA

AGAAGGAAGGAATGTGGTGATCTTTACTCACTGACAACTTCAGCAACACCTACTGCAAAA

ATGGTCTGTGGGGTTGTCACGAATGTTGTCGCTGCTGTTTTCAAATGGTTGGTGTCGCTA

AAATCTGAGATGGAACCAGCTTTTTCCAGGATTAAATTTGAGAAAGGATACAATATTGAA

ACTATCTTTGATGGAAACAAATTGGGAGTTGAGCCATACTCTGTGGAGGTTTCGCCGGCC

GGAGAGGTCTTCATTTTGGATTCTGAAAACAGCAACATTTACAAGATCACAACACCACAC

TCTCTGTATAGCCAGCCTATTTTGGCCGTCGGATCACCTGAAGGGTGCTCTGGGCATGTG

GATGGGAAGCTGAGAGAGGCAAGGATGAACCACCCAAAAGGGCTAGCCATGGATGACAG
TGGAAACATGTATGTTGCAGATGCCATGAACATGGCAATCAGAAAAATCACTGATACAGGA

GTTGTGACAATTGCAGGAGGGAAGAGGTTCCAAGGCGACAGACCGAGGGAAGACGATGC
AAAACTTTCAAATGACTTTGATGTGGTTTATGTTAGAAGCAGCTGCTCACTTTTGGTTATC

GACAGAGGAAACCGGGCGATTAAACAGGTCCAACTTCACAACCATGACTGTTATGATCAG

TATGACAGCAATCTCCATCTAGGTGCAGCACTGCTCTTTGCATCCGGTTTCTTTGGATAC

ACGCTGGCTCTGTTACAACGAAAAGTTGCAGCGTTTTTCTCATCAATCCACCGGCACACC

AACCCTATACGACCTGGCTTAGTTCCAACAGAAGATGAACACAAGAAACCTGCAGAAAAC

ATGTTTGTTTCCATTGGAAGGCTGCTAGTCAACATTGCCTCATCTGTTACTGAAATGTTT

GGTCGAACGTTGTCTGCTTTTAGAAAGAAGCCTCTGCAGCCTCATTTTCAGCAGAATCTC

CATTACTCTCACAAGCATTCTCAATACTATACAAATACAATTGGTTATGAAGATGACCTC

CCATTTTCAGACCAGAGATACCCTCCACCAAGAACATCATCGTACTCGTCAATGGCCAAA

GATATGGAGAGGACTCAACATTTCAGACAACACCATCATCAGAAGAAACAGCCATCAAGT

CCTCAGACTTGCACAGAACAACGTTGTGAAAGGAATGAGGTTGTGTTTGGGGCAGTTCAG

GAACAAGATGGCTGCGGTGAAGCAATGGTGATAAAGAAAATCGATTACTCAGATCCTGCT

TATAACCATTATAACATCCGATCTAGATACGGGAATTGA

>g40997.t1/IbHAK16

ATGGATCTGGAGACTGGGTTTCGCCACAATAGTTCAAAGAGACAATCTTGGAGGACAGTT

TTGACATTGGCATATCAGAGTTTGGGTGTTGTGTATGGGGATTTGAGTACCTCCCCATTG

TATGTGTACAAGAGTACTTTTGCTGAGGATATTAAGCATTCAGAGACCAATGAAGAAATC

TATGGGGTTCTATCCTTTATCTTCTGGACATTGACTTTGGTCCCTCTCCTAAAATATGTG

TTCATTGTGCTTAAGGCTGATGATAATGGTGAAGGGGGAACATTTGCCTTGTATTCTCTC

CTTTGTAGACATGCCAGGGTGAACTCATTGCCCAGTTGCCAGTTAGAAGATGAGGAATTG

TCTTTTTATAAGAAGGACATAGTTTCTCCTGCACCCACTACTGTTGGGGCAAGGTTGAAA

TCAACTTTGGAGAGGCATAGGGTGTTGCAGAGATTTCTGCTTGTGTTGGCTTTAATTGGG

GCTTGTATGGTCATAGGGGACGGTATTCTTTCGCCCGCTATCTCTGTTTTTTCAGCAGTT

TCGGGTCTAGAGCTCGCCGTCTCTAAAGAGCATAACAAATATATAGAGGTTCCGGTTGCT

TGTATCATACTGATAGCATTGTTTGCCCTTCAACACTATGGCACTCATAGGGTCGGGTTT

TTGTTCGCTCCCGTGGTCATAACATGGCTTGTATGTTCAGAAGCCATGTTTGCTGATTTG

GGGCACTTCTCCCAGTTATCAATACAGCTTGCTTTCACGTTCCTGGTTTATCCGTCATTG

ATTCTTGCATATATGGGGCAAGCGGCTTATCTGTCACAACATCACGTTATTGAAAGTGAC

TATCACATTGGCTTTTATGTTTCTGTACCTGGTAGGTATCCGAAGCCAGTACATTTTTTG

CTAGGTAGATTTGGCAAGTTCGGTTTGTTAAAATTGTCTAAAACACTTCGTCCAGAGAAA

GTTAGATGGCCTGTTCTGGTGATAGCGATACTGGCTGCTGTTGTGGGGAGCCAAGCCATA

ATCACTGGAACTTTTTCAATTATCAAACAATGTTCTTCCCTAGGATGCTTTCCGGGAGTC

AAAATAGTACACACGTCATCAAAAATTCACGGGCAGATTTACATTCCGGAGATCAATTGG

ACCTTGATGCTGCTGTGCTTGGCTGTTACGATTGGTTTCAGAGACACTAAGCGCCTAGGC

AATGCATCGGGAGGGTGTTGGACTTGGCCCTTCAGTCCTCCGCCCAATAATCTCCAAGCC

ATTGGGTTTGGCTGTTATAACAGTTATGCTGGTCACAACGTGTTTGATGTCACTGGTTAT

GGTTCTGTGTTGGCACCAGAGCGTCTTCCTTGCGATTGGCTTTGTGCTCTTCTTCGGGAC

CATCGAAGCCTTATATTTCTCTGCATCTCTCATCAAATTTTTGGAAGGAGCATGGGTGCC

CATTGTAATGGCTTTCATCTTCATGATAGTCATGTGTGTTTGGCACTATGGATCCCTAAA

AAATACGAGTTCGATGTCCAAAACAAGGTCTCGGTTGATTGGCTGCTCAGCTTGGGTCCT

AGCCTCGGGATAGTACGTGTGAATGGCATTGGCCTGATCCACACCGAGCTCGTGTCGGGA

ATCCCAGCTATATTCTCCCATTTCGTCACCAACCTCCCCGCATTCCACCAAGTCCTCGTT

TTCCTTTGCATCAAGTCTGTCCCGATTCCTCATGTCAAACACGAGGAGCGGTTCCTTGTA

GGGCACATCGGTCCCCGAGAGTACAGGATATACCGTTGCATTGTCAGGTATGGTTACCGG

GATGTACACAAGGACGACTCCGGCTTCGAAAATGACCTCGTGTGCAGCATAGCCGAATAC

ATCCGCACAGGAAAGGCGGGATCGAATGGCGCAGCAACAGCAACAGCAGCAGCAGCAGC

TGAAGAAAACTCGGCACAAGAACGCGAGGAAATGGCCGTGATGGGAACGCCTTCAACCTA
CTTAGGCGGCGTGTACGTCTATGAAGACGGCGAAGGCCACTCCTCCTCGGCCGGAACATC
AGAGCTTCGAGAAATAAAGTCGCCCCCGGTACTAAAACCGAGAAAAAAAGTGCGGTTTGT
CGTGCCCGAGTCTCCGAAAATTGACCAGAGCGCCCGCACAGAGCTCCGGGAGCTGATGGA
AGCTAGGGAGGCAGGTATAGCTTACATCCTTGGACATTCCTATATGCAAGCCAAGCAAGGA

TCAAGCTTTGTAAAAAAAATGGTTATAAATTTTGGGTATGAGTTCTTGAGAAGAAATAGT

AGACCACCCACCTATGCTTTAAGTGTACCTCATGCTTCCACATTAGAGGTGGGAATGGTT

TACAATATCTGA

>g36794.t1/IbHAK11

ATGGCTTCTGGGATGGAGATTGATGAAGACGAAAATGAAACTAAGGGAGGGATGTGGGTT

TTGGACCAGAAGCTTGATCAGCCTATGGACGAAGAGGCTGGAAGAATAAGAAACATGTAT

AGAGAAAAGAGCCTGGGAGTAGTGTATGGAGACCTTGGGACATCTCCCTTGTATGTGTTT

TACAATACATTTCCTCGAGGAATTAAAGATACAGAGGATGTTATTGGTGCACTTTCGTTG

ATTATATACTCACTAACCCTTGTACCACTGCTCAAGTATGTTTTCTTAGTGTGTAAGGCA

AATGACAATGGCCAAGGTGGTACCTTTGCTCTTTATTCCTTACTCTGCCGACATGCTAAG

ATAAAAACAATTCCCAACCAACACCGGACAGATGAGGATCTAACAACTTATAGCCGTAAC

ACATTCCAAGAGCATTCATGTGCTGCAAAAACAAAGAGATGGCTGGAGGCATATGCGTTC

CGAAAGAATGCACTGCTTATTCTTGTCCTTGTTGGTACTTGCATGGTAATTGGGGATGGA

ATTCTTACTCCTGCAATATCAGTTCTTTCAGCTTCTGGTGGGATCAAGGTTGACCATCCA

AAGATGAGCAATGACGTAGTTGTGCTTGTTGCTGTCTTTATATTAGTTGGCCTGTTTAGC

ATGCAACATTACGGTACAGACAAGGTTGGTTGGATGTTTGCTCCAGTTGTACTGCTCTGG

TTTCTTATTATAGGAGGTATTGGCATTTTCAACATCTGGAAATATGATACCTCTGTTCTG

AAGGCCTTTTCCCCTCTATACATATATCACTATTTGAGAAGGGGGCGTAAAGAGGCTTGG

ATATCACTTGGAGGGATCATGCTCAGTATCACAGGGACAGAAGCACTTTTTGCTGACCTT

GCTCATTTTCCCCTCTCTGCAATTCAGCTTGCTTTCACAGTTATTGTATTCCCCTGCCTT

CTTTTGGCCTACATTGGACAAGCAGCCTACCTCATGCAACATAAAGATCATGTTGCCGAA

GCATTCTATCGTTCTATACCAGAGAGCATATATTGGCCGGTGTTTGTCATTGCTACTTTA

GCTGCTATTGTGGCCAGTCAAGCCACCATATCTGCTACTTTTTCAATAATCAAGCAAGCA

TTAGCATTAGGCTGTTTTCCAAGGGTCAAGGTTGTGCATACGTCAAAGAAGTTCCTTGGA

CAAATATATATTCCAGATATCAATTGGATCCTTATGATACTCTGCATTGCTGTGACAGCT

GGTTTCAGAAATCAAAGCCAAATTGGCAATGCCTATGGTGAGTTCACTAAGTAG

>g32726.t1/IbHAK17

ATGTTCTTCCTCACAAGCACTGCTCCTTTGTATGTATTTGGATCAATTGACGTAAAGGAT

GTCAAATCAAAGGAGGAGATATATGAACTCTTCTCCTTTGCGTTTTGGACTTTAACCATC

ATTCCCTTGTTGAAATATGCACTTATAGTTTTGAAGGCTGATGACCAGGGGGAAGGTGGT

ACGTTTGCATTGTATTCCTTGCTGTGTAGGCATGCCAAAGTTGGGCTGCTTCCAAGTGAT

AAAACTGCTGCTGAGACTATGCATCATGAGGAAATCCCATCTAAGATCAAAATGAGAACT

AGAGCGAGATGGGCAATTGAAAATCATAAAAGTTGTCACTATTTTCTGTTGCTTCTTGCC

CTGGTTGGGTCTTGTTTAATAATCTGCGATGGAGTTCTAACTCCTTCTATTTCTGCAGCC

TCTTCTGAAGAGGCAGACTCCATTGACAAGTATTTGACAAAATATATCCCAGTTCCTACA

GCATGTGCTATATTGGTTTGCCTTTTCACTTTGCAAAAGTACGGGACCCATAAGATTGGA

TTCCTTTTCGCTCCAGTTGTCATAATATGGATTGTTTTCATCAGCACGATGGGCATATAT

AACATTTCCCGTTACCCCTCAATCCTTAGATCAGTCTCCCCAGTGTATATTGTTAGGTTT

ATTAGGAATGCAGATATTACAAGTTGGAAACTATTAGGGCGCATTGTGCTATGTATAGCA

GGATCAGAAGCAATGTTTGCAGATCTAGGGCATTTTTCAAAGAAATCCATCCAGGGTACT

AGACTTGGCCTTTTATTGGCCTCCACCCAACAATCCCCTAGTCACATGGTGCTGGACTTG

GCCCTTACTGGCTTCCGCCCAACAATTGTGGTCACTTTTGCCCTAATAATATATCCTGTT

CTTATAATATGTTATGCTGGTCAAGCTGCATTTATCTCCAACCACTTAGGTGTAACACCT

TACGTTACGCATCTTAGTGAATCTCTACCTATTAGTAGAGATCTTCATCACGTTTTCACG

GTGTTATCCCTAATAGCGTCTCTTGTGGGTAGCCAGGCAACCATTACTGCAAGTTTTTCC

ATCATAAACCAATGTCAAGCACTGGGCTGCTTCCCCAGAGTCAAAGTTATACATACATCA

GATAAGATATATGGACAGGTTTATATTCCTGATGTCACATGGATTCTAATGGTCCTCAGT

TTAGGTGTTACATTCGGTTTGGGGGACATAACAACTATTGCGAATGCAACAGAGTTCGAA

TCCTTGCCCCCTTGGTTTGAGCCGGTTAACTATAGGAAACCTAGACTGGTTTACTTGCTT

GTAGTCCTTTGCTGGCTGGGTCACAAGGCAGGGTTTACTCACACCCAAAAGGGTTGTTTA

ACTGTTATTTGTGGGATGTCAGTCACATCTTGTCTTATGTCACTTGTTATCGCGCTCTAC

TGGGACAAAAGCCTATTCTTCTCGGTGTGCTTTGTGTTAGCCTTTGGGTCTGTCGAAGCC

ATGTACCTGTTCTCGAGTTTGATGAACTTTTCCAAGGGAACCTGGTGTGTACTTGTGCTA

ACCTCACTTTTTCTGATGACAATGCTCTCATGGCACTACGGCACTGTTAAGAAGTACGAG

TTTGATGTAGAAAACAAGGTTGCAGTGGATTGGTTAACAGATCTTAGCCCTGGTCTGGGT

GTTAACAGAGTACAAGGTATCGGCTTTATTTACACTGAAATTGTGACAGGAATCCCTTCT

TTTTTCTCCCATTTTGTCACCAATCTTCCAGCATTTCATGAACTTCTGGTGTTTGTGTCT

TTCAAGCCATTACCCGTGCCTTACATTCCCCAAAGTAAGCGATACCTTATAGGCAGGGTT

GGCCACAAGGAGTACAAAATCTACAGGTGCATTGTACGATATGGATACTGCGACCACATG

AGAGACACACATGATTTTGAGGAGCACATTATCAGTTCAATAGGGGAATTCATTGCCAGG

GAGGAACAGGAAGATGAAGATCAGGAAGCTATGGCTGAAGGAAGGATGATCGCTTTAGG
CGGGCCTAGTACAAGTGCAGCATTGATTCCAGTAACTGAAATTGAAGAAAATGAGGTTTCA

CAGACCTCAGCTGACATCGAGTCTCAAAGAAGTCCTCTCATAGATTCATACCCTCCTGTC

AAGAGAAAGAAGGTCCGCTTCTTCCTACCTCCAACCAGTCCCAAGGTGAATCCAGCCGTG

AGAAGGGAGCTCCAGGAACTAGTCGACGCCAGAGAAAGGGGCACTGCTTATTTCTTGGGC

AAGCCACACTTATCCGTACGTAAAGGCTCAAACTTTTTCAAGCGATTCCTTATCATGAAC

TACATTTTTCTCGACAAAAATTGTCGAGAGCCTCCAGTTGCATTGAACATACCTCATGCT

GCTCTGTTAGAGGTAGGAATGGCTTATATAGTGTAA

>g17727.t1/IbHAK15

ATGCCTTTGCTAAAGTATGTGTTTGTGGTACTGAGTGCGGATGATAATGGTGAAGGTGGG

CCATTTGCTCTATATTCTCTACTTTGTAGACATGCAAAGTTTAGTCTTCTTCCTAATCAA

CAAGCAGCTGATGAGGAGCTATCTGCCTACAAATATGGTCTCTCCAGGCAGTCTGCATCA

TCTTTGCCATTCAAGAGATTTCTTGAGAAGCATAAAAAATCACGGACAGTTCTACTCCTA

GTAGTATTACTGGGGGCTTGCATGGTCATCGGTGATGGTGTACTTACTCCTCCAATGGCA

GTTTTGTCATCAATTTCTGGGATTCAGGCTGCTGCAGAAAAGATGTCTAATGGTGAAGTG

CTATTGATTACTTGCATTATTTTGGTTGGGCTATTTGCACTTCAGCATTCTGGCACCCAC

AGGGTAGCTTTCTTGTTTGCCCCAATAATAATAATCTGGTTAGTATCAATGTTTTCACTT

GGGCTGTACAACACGATATTCTGGAATCCCAAAGTTGTCTCTGCTCTTTCTCCTCATTAT

ATCGTCAAGTTTTTTCGGGTAACTGGAAGAGATGGTTGGATTTCACTTGGAGGTGTCCTG

CTATCGGTAGCAGGTACTGAAGCTATGTTTGCAAACCTTGGCCATTTCACTGCCTTCTCA

ATGCGGCTTGCATTTGTATTTGGTGTATATCCTTGTTTAGTGGTGCAATACATGGGTCAG

GCTGCTTTTCTGTCCAAAAACATTGCTTCTATTCCAAATTCTTTCTACGGGACTGTACCT

GGCAGTGTATATTGGCCTGTTTTTGTTATAGCTACCCTTGCAGCTATTGTTGGCAGCCAA

TCTATTATCACAGCTACTTTCTCTATTGTCAATCAGTGCCTAGCATGCATGACTGTGATG

TTTATCACGACATTTCTCATGTCACTTATCATGGTCTTTGTCTGGCAAAGAAGCATCGCA

CTTGCCAGTATTTTTCTCCTCTTCTTTTGGGTTATTGAAGGTGTTTACCTGTCAGCATCA

TTAATGAAATTCCCACAAGGCGGATGGGTCTCCGTTGCGCTCTCTCTTGTATTCTTGTTT

GTCATGTTTGTTTGGCACTACGGATCCCGAAAGAGTCCGTTCCTGTTCCCCATGTTCCAC

CCGAGGAGCGTTTCCTTATTGGCCGTATTTGCCCAAGACCCTATCGCATGTACCGGTGCA

TCGTCAGATATGGGTACAAGGATATACAGAGATAATGGGAACTTTGAAGACCTGCTGATC

CAAAGCATAGCAGAGTTCATCCAAATGGAAGCCGAAGAGCCACAGCTATCGAGTTCTGAT

AGTTTCTATGGGAGAATGGCAGTTGTAAGTACCAGGACTATACAGTCAAGCTCAAGTCTA

GTAATACCTACAGAAGAAGACTTGGGTAAGAATCATTCCTTTCAAAACAGTAAATCTCCT

TCTCTCCAGAATCTAAGATCTTTTTATGCCGACGAGAACAAGAAAATCAGGAGACGGGGA

GTAAGGTTCCGGGTAGCAGAAAGACTTGCAATGGATACAGCAGTGAGGGAAGAGCTTGAA

GACTTGATTGAGGCAAAGGAAGCAGGGGTAGCATACATAATGGGACACTCTTATGTGAAG

GCAAGGAGGTCATCCTCATTTTTGAAAAAGATTGCTATTGATATTGCATATTTATTTCTT

AGGAGGAATTGCAGGGCTCCTTCTGTGGCTCTCAACATTCCCCACATAAGCCTCATTGAA

GTTGGGCAAGGCAATGGAGTGGGCCTTTCAGTGAGGCTCCTGCTCACATTTAAGGTGAGC

AATTGGGTGGATTTGAGGAAACAGCCTGAGCTGAGGAAATGCAAGTGGGAGAGGGGAAG
GGAGGGCAAGTCAGCTATAATTCGTAACTTCTCGTTGCGCCGTTTGGTAGGAGTTGCAAGT

GCAGTGTCATCTGACTTGTATTGCAATAACCAAATAATTGACGGGGGACGGGATGGAGAC

TATTTTCCATTCCCTGTCACGGAGATGGGGACGAGGGCGGGGAGTATACTCCCCGCCCCC

ACCCTGTTGCCATCCCTAGTCTCGCCAACACTGAATATGGCCAAGGCGGCAAGCCAAGGA

GCATATATGAAATATGGAATGGAATATAGCTTAACTTCAAGCTTCCGTTTTCATGGTTTG

CATGCCATGCAGTGTCTTGAAGAAGCAGCTTGCAAAGGCATATAG

>g46647.t1/IbHAK14

ATGGCGGACGAGGGATCGGAGAGGGAGTATGGCGGATTAGGCTCTCTCGACTCGATCGAG

TCGCGCTGGGTGTTTCAGGATGAGGAGGATGAGTCGGAGATCTATAATGACGGCACGGAT

GCGGGGGAGAGCGGGGACGATTCGCTTCCGCGGGATGGGATGGACTCTGACGACGAGGA
TAATGCGGAGCAGAGGCTGATTAGGACTGGTCCTCGGATTGATTCCTTCGATGTCGAGGCT

CTTGAGGTCCCTGGCGCTCAGAGGAATGACTTTGAGGATGCTAGTGTAATGAGGAAAATT

GCATTGGCTTTCCAGACGCTTGGAGTTGTGTTTGGTGATGTGGGGACAAGTCCGTTGTAT

ACTTTTAGTGTGATGTTTAGTAAAGCCCCTGTTACTGGGGATGAAGATGTTCTTGGGTCG

CTATCAATGGTCTTGTATACGTTGTTTCTGTTTCCACTGGTTAAGTATGTGTTTATTGTT

CTTTGGGCCAACGATGATGGAGAAGGTGGTACATTTGCTTTATACTCATTGCTTTGTAGG

CATGCTAATGTTAGCCTTCTTCCCAATCAACTGCCATCAGACAGTCGCATATCAAGCTTC

AGACTCAAGGTTCCATCTCCAGAACTTGAGAGGTCTTTAAAAATAAAGGAAAGACTTGAA

TCTTCAGTTACATTGAAAAAGCTTCTTCTGATGTTGGTTCTTGCTGGCACCTCCATGGTG

ATAGCTGTCGGTGTAGTAACTCCTGCTGTGTCAGTGATGTCTGCCATGGGTGGTTTAAAA

GTTGGAGTTGCTGCAATTCAGCAAGATGAAGTGAAGATGATTTCAGTGGCATTTCTTGTA

ATTTTGTTCAGTTTACAGAAGTATGGCACAAGTAAAATGGGGATTGTAGTTGGCCCGGCC

TTATTTATTTGGTTTTGTTCTCTTGGGGGCATTGGCCTTTATAACCTTACGAAGTATGAT

AACCGTGTATGGAGAGCATTTAATCCATTTCACATCTACAACTATTTCAAGCGTGATTCT

ACTAAGGCCTGGCGTGCACTTGGCGGCTGTCTTTTGTGTGCAACAGGTTCTGAAGCAATG

TTTGCAGATCTTTGCTACTTTTCTGTGCGATCGATTCAGCTTACTTTTTTGCTTCTTGTT

CTGCCGTGCCTTCTGCTGGGTTACCTTGGTCAAGCTGCTTACCTGATGGAGAACCATGCT

GATACAAAAGAAGTTTTTTTTTCTACTTTGCCAAGTATTGCTGAACTTGGCGTGATGATG

ATGAGTACAGCACTTGTAACTATTGTTATGCTTCTCATCTGGCAGATAAACATAATTATT

GTGCTGAGCTTTGTTTTTATTTTCTTGGGTTTGGAGTTGACATTTTTTTCTTCGGTGTTA

TGGTGTGTGGGAGATGGAAGTTGGATCATATTAGTCTTTGCTGTTGTCTTGTTTCTAATA

ATGTATATATGGAATTATGGAAGCAAGCTGAAGTATGAAACTGAAGTCATGAATAAAATG

TCCATGGATTTGTTGCGGGAATTGGGCCCCAACCTAGGAACAATCAGAGCTCCTGGAATT

GGATTGCTTTACAATGAACTGGCAAAGGGAGTACCAGCAATTTTCGGGCATTTCCTTACC

ACCCTTCCTGCTGTACATTCAATGGTAATTTTTGTGTGTATAAAATATGTTCCTGTTCCT

GTTGTGGCTCAAAAAGAAAGATTCTTGTTCCGACGCGTCTGCCTGAAAAGCTACCACATA

TTCCGTTGCATTGCCCGATATGGTTACAAGGATGCCCGCAAAGAAAATCACCAGGTTTTT

GAGCAGCTGCTGATCGAGAGTCTTGAGAAGTTCATTCGTAGAGAAGCTCAGGAAAGGCAA

CTCGAAAGTGATGGTGATGATGACTCGAATAGTGGAGACGAGTGCACCTTTTCAAGGGTC

GTTGTAGCTCCGAATGGGAGCATGTTTACACTAGGTGTTCCTCTCTTGGCTGATATCAGG

GACACAGGGAAAACAGTTGCCAAAGAAAGCACCTCTTATGGGCACGGGCCCGGGCCTTCA

GCTAACTCAAACTTGTCTGATGCAGAGCAGAGTCTTGAGATGGAGCTGTCATTTCTACAC

AAGGCCAAAGAATCAGGTGTTGTTTACCTTCTCGGTCATGGAAATATCAGGGCAAGGAAG

GATTCTTGGTTCATTAAGAAGCTCATCATAAATTATTTCTACGCCTTCCTGAGAAAGAAC

ATCAAATCAGCAGGGCGTCTAATGCCACGTTCTATCCCAACACAAGTCACATTCATACTA

AATCCTTCTCGTTCTGAAGCAAGATTTGCGGGAGGTTTTTGA

>g17621.t1/IbHAK13

ATGCCTTTGCTAAAGTATGTGTTTGTGGTACTGAGTGCGGATGATAATGGTGAAGGTGGG

CCATTTGCTCTATATTCTCTACTTTGTAGACATGCAAAGTTTAGTCTTCTTCCTAATCAA

CAAGCAGCTGATGAGGAGCTATCTGCCTACAAATATGGTCTCTCCAGGCAGTCTGCATCA

TCTTTGCCATTCAAGAGATTTCTTGAGAAGCATAAAAAATCACGGACAGTTCTACTCCTA

GTAGTATTACTGGGGGCTTGCATGGTCATCGGTGATGGTGTACTTACACCTCCAATGGCA

GTTTTGTCATCAATTTCTGGGATTCAGGCTGCTGCAGAAAAGATGTCTAATGGTGAAGTG

CTATTGATTACTTGCATTATTTTGGTTGGGCTATTTGCACTTCAGCATTCTGGCACCCAC

AGGGTAGCTTTCTTGTTTGCCCCAATAATAATAATCTGGTTAGTATCAATGTTTTCACTT

GGGCTGTACAACACGATATTCTGGAATCCCAAAGTTGTCTCTGCTCTTTCTCCTCATTAT

ATCGTCAAGTTTTTTCCGGTAACTGGAAGAGATGGTTGGATTTCACTTGGAGGTGTCCTG

CTATCGGTAGCAGGTACTGAAGCTATGTTTGCAAACCTTGGCCATTTCACTGCCTTCTCA

ATGCGGCTTGCATTTGTATTTGGTGTATATCCTTGTTTAGTGGTGCAATACATGGGTCAG

GCTGCTTTTCTGTCCAAAAACATTGCTTCTATTCCAAATTCTTTCTACGGGACTGTACCT

GGCAGTGTATATTGGCCTGTTTTTGTTATAGCTACCCTTGCAGCTATTGTTGGCAGCCAA

TCTATTATCACAGCTACTTTCTCTATTGTCAATCAGTGTAATGCACTTGGTTTGCTTCCA

CGGTCAAGATTGTCCACACCTCAAACCATATTTATGGGCAGGTGTTTACCTGTCAGCATC

ATTAATGAAATTCCCACAAGGCGGATGGGTCTCCGTTGCGCTGCTCTCTTGTATTCTTGT

TTGTCATGTTTGTTTGGCACTACGGATCCCGAAAGAGGAGTTCCTTCTGTTTTCTCACAC

TTTGTGACAAATCTCCCCGCATTTCATAGTGTTTTGGTATTTGCGTGTGTAAAGTCCGTT

CCTGTTCCCCATGTTCCACCCGAGGAGCGTTTCCTTATTGGCCGTATTTGCCCAAGACCC

TATCGCATGTACCGGTGCATCGTCAGATATGGGTACAAGGATATACAGAGAGATAATGGG

AACTTTGAAGACCTGCTGATCCAAAGCATAGCAGAGTTCATCCAAATGGAAGCCGAAGAG

CCACAGCTATCGAGTTCTGATAGTTTCTATGGGAGAATGGCAGTTGTAAGTACCAGGACT

ATACAGTCAAGCTCAAGTCTAGTAATACCTACAGAAGAAGACTTGGGTAAGAATCATTCC

TTTCAAAACAGTAAATCTCCTTCTCTCCAGAATCTAAGATCTTTTTATGCCGACGAGAAC

AAGAAAATCAGGAGACGGGGAGTAAGGTTCCGGGTAGCAGAAAGACTTGCAATGGATAC

AGCAGTGAGGGAAGAGCTTGAAGACTTGATTGAGGCAAAGGAAGCTGGGGTAGCATACAT

AATGGGACACTCTTATGTGAAGGCAAGGAGGTCATCCTCATTTTTGAAAAAGATTGCTATT

GATATTGCATATTTATTTCTTAGGAGGAATTGCAGGGCTCCTTCTGTGGCTCTCAACATT

CCCCACATAAGCCTCATTGAAGTTGGTATGATATATTATGTATGA

2000 bp promoters

>g13675.t1/IbHAK5A

ATATTTTAGTTAGTTTTATGGAAGAATATTAAAATCATTTTTCATTTACATGTCAATCTA

TATAATATGGTTAAATAATTGTATAATTTATGCTATCTCACACGAAAATACTTAACCTGT

TTTATGAGATGATATAGTAAAATTTGAAATTCAATTTGTCAAATTTAGTTTTCATTGAGT

TTTTTCGCTGGAAAAATATATACCTAATTTGAAGTCCCTATAAATTAATCTGATAAGATT

ATTTTACCAAAACTGCTTAAATTTTCATATTAAGTTGTTTCTGACTTTCTGACGTTTCTG

TGGTAAGAAATCTTGTCTAATGCCTTGTTTTACAATCACAATAGCCTATGGTTATCAACT

TTTATAATAGTTTGAAGTAAAACTTTTTTTTTGTTTGTTTTTAGAACTTTGATGACCAGA

TGGTTTGACTTTTTGTTGCATATTGATTAAATTTCACATGTAAATGCTGACTAACATTTA

AAATTAAGGTTTGGCATATGATGATGCCCATTTCTGATGGCAGTTTGGTATATAATTTTT

TTCAATTCAGTAAAAGCAAATACCCCATTTTATGCTATTTCACATGACCTAACCAATTTT

CTGAAATGATATACTAAAATCTGACATTTAATTTATTTGAACAAAGTTTGTTATTATTTC

CATTCTTATTATAATATAAAGTATTACACTTATTACTATTATTACTACTACAATAATTAT

TATTACTATAGTTAGTATTATTACTATTATTAATACTACTACCACTATTGTTAATTTGTT

ATTATTACTATTATTATTATTATTATTATTATTATTATTATTATTTTTACTACTCTTATT

ACTATTATTATATTACTACTACAATAATTATTATTACTATAGTTAGTATTATTCTTTTTA

CTATTATTATTAATTATTAATATTATTAGTTTTAGTATTTTTTTAAAATTATTATTAGAG

TAACACTAGTACTATAAAATTGACATTCTTTTCTTCCCCCCTCTCAATCTCTCATTCTCT

CTTTCTCTCAGCCCTCTCCGCTGCTCTCACCCCTCACACTCTCCTTCGAGGCTTATTCAG

GAAATCCCATATCTCCTTTGAGGCTTATTCTTGGATTTGTTGTTGAACAGTTCTCTATAT

ATGTGTGTGTGTGTATATATATATATATATATATATATATATATTTTTTTTTTTTTTTTT

TTTTTTTTTTTGTGTTTAGAAATTAGATATTCAGATATTGAAAAGTTTACGTCAAGATTT

TTCTTTTCCAGAAAATTAAATATTTTACCTGGAATCTTTTGAGTTCCCATGTCTAGTTAA

CAATTGTGTGTATTTTATTTCCTGAACAATTTTCAGTTGTTAACATGAATTTTGAATGTT

TCTTTTTTATTGTCTCTAGGGGCATATGCATATTAGCAGAGTTGTTAACTTGTTAACTAG

AAGCACATGTTTGTCATATCTTCTTTGTGTTAGCTTTTTTAATGGTTAATTGTCATTCTG

CTGTGCTATATTCTATATGTGCTTTGAATGCGTGTAGCTAAAATAATCCTAAAACTGAAT

TATTTGAACTTCTAGACAAATACAACTCGATGCCCAGGTAACCTCCAAAGTTTTTTAAAT

AACTGAACTATCTTCAATATAATACCATCATGACAGTAAGGTTAGCTTGCTGAGTTTATT

AAATATGTTAACAGTTTCCCCAGTTGTCTTGTTTGTTTCTCACTTGTGCCATTAGGAGGC

TGGGGTTTGGAACGTTAGAGAAGAAAGAAAAAGGGAAAAAGAGGGAGAGAAAAAGAATA

GATATATTATAGTGATGACTGATGAGTACTTGAGCACTTGACTACCTGATTGTTTTTATTT

TTTTCAAAATGTAAACTTCTATTTATTTATTTAGGTAAAACTTGATAATGGCATATTATT

GAACCATGCTCTGGCACTTTGCACTTTGCAGATCCTGTGCATTTCAGTAGTCTGTATAAT

TCCTACTTGTTTTAAAAAAC

>g13677.t1/IbHAK5C

AATCTCTATTGACTTGATCAATCATATTTACCTCAACAATAGCCTCAACCCTGCTCTTTA

CTCTGAGTTTGGTAAGTCTATACACGGTAAAATAGCCTTCACAGTTCACACACTTGATCT

GATAAAGGTGTCATTGCAAAAGCAGTAAGAAAGTTACGGAGCATCAGAACATGAGAAAGA

AAGAAAATGCAGTAAACTAAAAAATTCTATGGTAGGTAGGCCCACTATTATATGGACCCG

ATCCACCTTATAAGGTGGTCCCGTCACAATTTATATATTGAACGTTCATAATTTGAATTG

TAAGCGTTCAGTATGTAAATTGTGAACATTCAATATATAAATTGTAAATATTTAATATGT

AAATTGTGTATTCGACAAAATAATTTGTCGTAGGCTCAGGGTTGGGCCTAAAAAAATCTA

TTATAAACTCAGGTTGAACCTAAAAATGTCTATTTATCGGCTTAGACTTTAAATTCGCAT

AACAAGTCCATGTCTAATTTTTCGAGCCTAGCTTAACCTATTTTTATCGCTATATAGTAC

TAACTATAATTCGAGCATTGAGCTATTTAATTAATGTCCAATACGAAAATTAAAATAAAT

AAATACTTAGTACAATTTTTAAATATTAAATAATTAATATAAAATAAAAATAATTATTTT

CAATATAATTAAAATGAGTATTTTAATTAGGAGTAATAAAGATGTATTTTTGTAGATAGT

TATAATCATAAATAAAAATGGTGTCCTAACAATTTTTCATATTAAAATAAATTAAGTCTT

CTTAATGCAATATTATTGATGAGTTAGTAATTAAAATTTAACTTATTTTTTGTTAAAAGT

TAGATGATAAATAGCTTACTGATATTATCGAATAGTAATTAAAGAATTATGTAACTAAGT

TATAACATATGAATTCATAAGTTTTTGTTGTGTGTATATGATTGAGACCTCAATTGTTGT

TATATGTGAACTATGCATCTTATAAACTCTCATTGATATTTTAAAATATAATTGTTTTCC

CTATAAAATTGATTAGAGAATGAAATTTTTGCATTTAAATTCTTAAATCGAGAGAAATTT

TGTCTCAAACATAGTAAATTAGCTTGTAAAAAATGAACATTTTATTTAAAGCAATATAGA

TTTGGAATAGAAAATTAAAATTTACACTACAACAAATAAGAGTTGATTTAAATAAAGAGA

AGACAATGTATTATTAGAATAACTATAGTATGAAATTCATATTATTTTAATATGGAAATT

CAAAAAATAATAGATTTCACTTTTTTTTAATATATAGTTGAAAGAAACAAATCAAATTAA

ATAAATATTATTATATTTTAATTAATACCACAAAAAAAAAAGATTTGATAAGGAATAAAA

AATTGCATGAGTTTTATTTTGTGTACATTAATTTAATTGTTATGCACTGCATACATTATA

TGTTGTTGTCGTTGTTGTTATTATTATTATTATTATTATTAAAAAATTTAAAAAATAATT

TTTAATGAGTAGGATAAAAATAATAAATAGGTAACGCAATATTTTGTTACTAAAATTTGT

ACATAAATCGAAAGGTAAAAAGTGAAAGATGAGAACTTTAAAAATATTTTATAATTGTAC

ATACAATGACTAAATTAAAATTTACAAGTTATCAATTCATATTATTACACCATCCTAATT

TTTATTGATGTTATAACCTTACTAATATTAGCTGTTATTATATTTATTATTATATATATT

ATTAATTAGTAAATATCAACAATTTCACACGGTATATACACGCAAAATAAGTTTAGAATG

GTAATCCGTAATCCAAAGAAATTTTTTTCTTTTCTTGTCCAAACATTAAAATCTTCCCAT

AAATGTCTTCCTACGACAGATTTTTCTTATTTTGAGAGTTGACCCTATTTTACACATATA

TATACACACGTTTAGGTTTTGAAAAGGAACACAACAATCACCTCGTACTAGACTGCGGCA

GTGTAGATACCTGAGAAAAC

>g13676.t1/IbHAK5D

AACATGTTTACACAATGATTGATGTAAAATCAAGTGTTAGAACTCCAACATTGGCTATAG

CCTATAGGCAGCGAATTGGATGAAAAGATGAATGATATTGAATAAAGTAATTAGAGTAAA

TGAAGTTCTTAGTAGTTGTAATCAGAAAGTTGCAATTAACTTGTTGTGCTGCGCTCTGTT

ATAGAAACTATTTTTTAATTTCTTGGTTGATTAGAATTTAATTTGTCTGGCACTAGTGAA

TGTTCATTGCAACTTTCTGACATACATTTCATTTTCGATTAAGTTGCATTACATAACAAG

TTACGACTTGTCCGACCAGTATTATATTGTTCAATTTAATGGAATTAAAGTTCTTTAAAT

TTTCAATTCCTTCAAATATGAAGTTTCATGCATGTTTGATTGTTATGAAAAAATTTCAAT

ATAGTTTAATTATACATTTATTACTTTATACTCAACATGGCTACACAATTGTTGGTATAA

ATTAAGTGTTATAACCTCGATAATGATTATAGGCAACACATTGGATGAAAAGATGAATGA

TATATATTGAACAAAGTAATAAGAATGAATGAAGTTATTAGTAGCTTGGGGCTATACGAA

CAGTTTATTGATTTAAACATAGTATCAATCCTTTTAGGTCCTATAAAATACTATTTATAG

GACACAACATATTAGAACATTACAAATGTGTGATGGGGTGTCCTAACAATTTTCATATTA

AAATAAATTAAGTCTTCTTAATGCAATATTATTGATGAGTTAGTAAAATTTAACTTATTT

TTTGTTAAAGGATAGATGATAAATAGCTTGATATTACCGAATAGTAATTAAAGAGTTATG

CAAATAAGTTATAACATATGAATTCATAAGTTTTTCTTGTGTATATGATTGAGACCTCAA

TTGTTGTTATAGGTAAACTATGCATCTTATAAACTCTCATTGATATTTTAAAAAATATAT

ATAGTCATTTTCCTTATCAAATTGATTAGATAATGAAAATTTTACATTTAAATTCTTAAA

ATAGAGAGAAATTTTGTCTCAGATATAGTAAATTAGCTTGTAATAAAATTTACAATTTAA

TTAAAGCAATATAGATTTGGAATAGAAAATTAAAATTTACCCTACAACAAATAAGGGTTG

ATTTAAATAAAGAGAAGACAATGTATTATTAGAATAATTATAGTATGAAATTCATATTAT

TTTAGTATAAAAATTCAAAAAATAATAGATTTCACTTTTTTTTTAATATATAGTTGAAAG

AAACAAATCAAATAAAATAAATATTATTAGTATTCAAAAATTAATAAATATTATTATATT

TTAATTCTTACCACAAAAAAAAGAAGAAGAAAAGAATAAAAAAATAGCACGAGTTTTATT

TTATGTACATTAATTTAATTGTTATGCCATGCACTGCACACATTATATGTTGTTGTTATC

GTTGTTGTTGTTATTAAAAAGTTAGAAAATATTTTTTAATGAGTATGTCAAAAATAATAA

ATAGTTAACGTAATATTTTAATTTGTTACTAAATTTTTTACATAAATACAAAAGTAAAAA

GTGAAAGATGATAACTAATTTGAAAATGTTTTATAATTGTACAATAACTAAATTAATATT

TACAAATTATCAATTCATATTATTACGCGGTCATAATTTTTATTGATGTAATAACTTTAC

CAATAATATTAGCTGTTATTATATTTATTATTATATATATTATTAATATTAATTAGTAAA

TATCAACAATTTCACACGGTATATACACGCAAAATAAGTTTAGAAGGGTAATCAGTAATC

CAATGAAATTTTTTTCTTTTCTTGTCCAAACATTAAAATCTTCCCAAAAATGTCTTCCTA

CGACAGATTTTTCTTATTTTGAGAATTGACCCTAGACCCTAGTTTACACATATAAATACA

CACGTTTAGGTTTTGAAAAGAAACACAACAATCACTTCGTACAACTAGACTGCAGCGTAG

TAGCTTGGTCTGAGAAAAAC

>g13673.t1/IbHAK5B

TAAAATATTTCAGGGAAAAAAAATCTCATTGATACCTTTTAACCTAGATTTATTTTCACT

ATTTCAAGAAATTAGTGTGGCGTTTGGTTGGAAATTTGTGTACCCCTACAATAATTTGAT

ATTCATTACCAAGTTTGTTTGCCTATTTTTGTTACCTTACTATTACATTTTTTTTGTTAG

ATCACATGGTGTCCTGAGCAAGCCATATTATAGGAGCCACCTTTAAGCTCATACCATACT

CTAATCCTCGCTCAATTTTGATTAATTTTTCCATACAAAAGGGGTTCAACTCCGACTCTC

TTAAAGACGAAGTCGGCCGAAATCTTAAATTAATGGATCATTCATAGTATTTGTCATTTG

TCATTCACTTCACGGCATCGTTGGAATAACATTTGACAAATCGATGTTCCCTTTCTATTA

TTGCTCATCTTCCGTACGCAATCCAACGTTATTTGGTTTACTTTTTGTAATTATTGTTGT

AATAAAATCTTAATTAATAAAATAATTACAACAACTACGTATTAGTTATCAGTTTTGTTT

TTTGCGTATCCCACGAAGCTTCGCGGTATATCAGGCGCCGGTAGCAATTGAAAGAAATTA

ATTAAAACTCAACCCAATGTTTTTGAAGGGCAAAAGTCAACAATGTTAAATAGCAAAAAC

ACCTTGTAATTAAATATCCCAGTGATATGGATATTTGGAGCGTACACAACTACTCCCGAC

CTTATACCGACCTCGGTAGTGTTTGTCTTGAGCTCCCAGGTCGGGACTAACTTTATCAGG

CGAGAAAAAGACTAATAAAGTGTTTTCTCAACCATCTCATCAACTTTAGCAGACTCAATA

CAAACATGTAATACGATTGTTTTAACTACTTTATGGGGAAGCGTTGTATGCCTATTACCT

AGTACACTTCGTCAACAGGATATTCCCGACATGTATCCCCTGTATAGCATCATAAAAGCC

AATTGATCTTTCAAAATAGGAAACTTTAGAAAAATCATATCAAAATACTCCTAACTTGAC

TCAACAAATACGAAGGTTCCCGGCCTTAAAACTAGGATCACTTCCTACATCTTAGTCAAT

ACACACATTCATAATTGTGAAAAATCATTATCAAGTAATACTATATTTGTCTCCTCTTTT

TTTCCCCCCTTAAAATTTGATTTTTACGGTATTTGATGCGATACACCTTAACTGGCTTAA

AAAATCTACAATTTTTTCTTCGGACAAAATTAGAATAAGTAAAAGGTACAGTACACTATT

AGAATATTCTTAAGCATAGTTGACTTGGAAAAGAGTTGGCCTATAGTACGAAAAGAAAGT

GACGAACAACAAACGCAATGTGGAGAAAGTGGCAATTAATAAAAAAAATATATTAATACA

TACAAAATTTTAAATTATTTAGGAATAAAGTTCTTATTAGTTATTACTTTATGAGCAAGT

GCATGTAATATTCTTGAAAAAAAAACATAATACTTGTATATTTTGATTTAAAAGTATTAC

ATTTTTCATCATAAGTATTATGTTTTCTCTTATAAGTCACAAATACTTTATTCATAATTA

GTATTGTATTTATTTTTCAATATAAATATTACATTTTCATAATGACTTGACCCAACATCT

TATAATAAAGATTCACACTTGTGACCCATAAAATTTTAACATTAGAAGTTTGGGAGAAGA

AAAAAGTTTTTTTTTTTCTTTTGAAAAGATTTTTTTTTTTTTCTAAAAGCATAGTTGAAT

TGGAAAGAGTTGACATATATAATACATATGGATTAATTACTTTATGTTTTATAATGCAGA

TTCCAATTTAATTATCTTAATGATTGATAAAAGTCAGAATTCTGATGTAATTGTTAATAT

ATTATACACATGATTGTGAGCAAACAACGTTGTACTTTATATTATATATATACACATGTT

TAGGTTGTGGAAAGGAACAGAACAATCACCTCCTCCTAGACCGCAGTGTAGTAGCTTGGT

CGGTAGATACTGGAGAAAAC

>g49793.t1/IbHAK5E

TCGGTTAATAATAATAAAAAAATTATCCTATCGAAATAACCGACATCGTAGGCTTTTTTT

AAAACCCTTCACCTCAGTTCAGAACGTTACAACGCGCCGCATAGCCCCACACCCCTCTTC

TCCTCTTCTCTTCTCCGTCGAACCAACCACCGACATCCTCACCACCGCACTTCTCCCACC

ACCTACATTGTGAGTTTTTGTATATTTTTTGAATTTTATTTCAAATTTTGAATTTTTGGA

GTTGACAGTAATTATTTTATTATATATGGAATTAATTATTGATGTATTTTGAATTTTATG

AATTATTAGGTATATATTTTGAACTTTATAAGTTTTTGTGTATATTATTTCATGAATTAT

TATTTTAAAATTTGAATTTAAAAATAAATAAATATAACACGTCAATTTGTTTAAAAAATT

GACGTGACATCGTTATAAAAAATATAAATTTAAATGCATACGATGTCGATTAAGTGTATA

AACGATGTCGAATTTATTTATTTATTTTTTAAATTATCTTATCACGTTGGTTTTTAGTTA

AAAACTGACGCGGTATATTATTTAAATTTTTTAAAAAATAAACTAATGTACAATGTCGAT

TATTATATTTAATTTTTTTTTTAAAAATAAAATTATACGACGTTGGTTTTATCAAAAAATC

GACGTGGTGTAATTTTTTTTTTAAATAAATGTACAACGCCGGTTATTAATAAATAACTGG

TGTTGTATGTCCTTTTTAATAAATTAAAAAATATACGATGTCGATTTTTATTTAACCGAC

GTCGTATGTTTGTTGTACAACGTCTATTTATATAACGTCTTTTTTTAACTGACGTTAAAT

GCTCTAAAAAACTTACGTTAAATGTCATTTATGTACTAGTGCAACAAGAAGTATATATAG

GAAACTTGTAAAAAATGGTTCAAATATGACATGATCTATTCTTCCCGCGGCTGGAGATGA

AGAACCTTTTTGCTGATTTTCAGCTTAGAGATGAAGTTCTAGCTCAGAAATGAAGACCTT

TACTGTGTCTCTGGCCGGAGATGAAAATATTAGTGAAAATATAATATTCTTTAATGAAAA

TATAATACCGACCGTTCACCAAAAAACAAAATTCAGATATATCGCCACATTTTTTAAAAA

AATGCTTCCTTTGTCATATTCATTTATTAGGAAGATTCACGAAGTCAAATTATCTCCCCT

TTTTTTCTTTTGCTTAACAACAAGATTTAAAAGTGCCAAAAGCTCATTCATTTAAGGAGT

CTTAATTGTTCTTAGTTATAAGTTTTTTCTTAAAAAAATAATAATATAGGGGCATTTTGA

AAAAAAAAAGATCTTTTCTCAAAATGCCATGGGGTAAAATTGGAATAGAATTTTAATTTA

TTTGAGAATTAGGAATAATACCTAAGGGCAGGAATCTATTCCCTTGAGGAGAGGGTCATT

CCATTCGAACAATGTTGGAATAGGAGTTTTACCAAACAAGGAATGAAATAGGCTTATTAC

TGGAAATGTTATTCCATTCCAAGGCTATTATTCGAACCAAACGCCATAGTAATACTAATC

AAAATAGTACGAAAAATAGAATTGTAAAATAACTTTAATTTGCTTTCATCGAATTGGTAA

AAAATTGAAGGGGGTGCTACTTTAATTTAATTTCTCACACCGTGTGAGAGAGGTGTGAGA

AAGGATGATGTAGTGAAGAGTGAAGACCACTTTACAGTTATCCCTAAAAAATGGGACATT

GCAACACAATAATAGATGATGGCGAGAGTTTTGAATTAACGCGTGCACGTGCCCACCCAA

ATACAAAGAACCATTGCATGCATCTTCCCTTCCTGCCGCCTATAAGTACGCATCTTTCCC

TCCTGCTTTCCCTGCACTTTCTCAACCCACACCCATAGATGGCCCGTCTCTAATATTTTT

TCTCTTTTATAACCAAAACTCTCTCTATCTGCACATATATGCTACTGTAAAATTTCCCTT

CTCCGATCCTCAGAAAAAAA

>g3900.t1/IbHAK8

TTATGTTCAAATATATGTTTTTTAAAAGGGAAAATCCCACCTAATTTTAGTAGTTTAAGA

TGGATTTGAAAAAAAAAAAATTATTTCCAGAACTTCAAGAAGATAAAGAGTACATCTTGAT

ATGTGTTGGCAGATATCAGTGTTCAGAGGCTTGTTATTAGTTATGATGGGACATTATCTT

TTATCAGTAAGGTAATAATTTATGATTATTTTTCAATTATTTAATTATTACAAGTGAAAT

AATTGTGTCAAAAATGAACTGTTTCCAACAGTTATTAGGTTTTAATGATATATGGTGGAT

GTGAAAGGTTTGGCAACTGAACATTTATGGTTGAAAATGGCGGCCCCATTTTGTTGATAG

AATCCACCTAGATTGGGCTTTGAATAATGGTCATTTTCCAGTGGAATGCCACACAAGCTT

GAAGCATTTTGAAAATGGAGAAAGAGAGAGAGGAAAAGGAAAAGAATCTGTAAAAAAAG
TTTGAAGGCTGAGAAAAGACAAGTTGCAGAAATATGGAAAAAGAGTGCCCTTTTTAGCTTT

TATGCTTTGAAAATGGAAAGAAAAAAAAATGGGGCAAACGTTGAAGCTGTGACCCGTGAAA

GGGACTAGCCAGCAATGGTGGTTGAAGAAAAGGGTCCCGGTGCTTATCACGTGGAACTCC

TATATGGAATGTAGTCTACACGTTACTGCTACTGATTTTGATGATTTCTACTGTTCTTAG

CTTCAGCTTTTGGTTTTCTTGAATTCAAACAGCCATTTTTGCCCTGGGGTGTGCTCTCAA

GCTCAAAAAAAAGCTTCAATTTCTGGTAAATTCCTTGATTCTTTATTCTCTTGTTGAGTGA

AAAGGAAAACCCACCGCCACTGCTCGATCGAGGACGTTTGTGAATCTAGTTGAGAAAAGA

TCAGACTGACTCAAATCAAGAAGGTTAATAGTCGTTTCCTTGAAACTTTATGGTTATGAT

GAATTTGGCATTACCCAAAATTTGTTGATTCTTGAGAGTAGAAGAGGGTCTAGTTTATAG

TTTCTTGTGTTTCATTGCATCCTAAATCTTTGAAATTTGTATGCTTTTTATTGCTTGGGT

CAATTGCAATCAGCCCAGCTCATGTTTAATAAGACCCTTTTTGCCATTTTGCCAATTTGC

CAGTTTGAATATAGTACAAATGCATGAGTTATCTCAATCTTCTGGCAGTAGAGGTAGGTA

GAAGTTTGGCAGTCTATTCATCACTGCTGCATAAACGCGTACACCTATACTATACAGCTG

AGGGTATATTATACATGTATGTATGTATGTATGTATGGATATTGGGTAATATCTTTTGTG

TCTGTGTCCTTAAAATGGCATTTATTTCTGTGGGCTCAAACTTTTGGTATGATTTGTTTG

CTGTTGAATCATGTACTTTTGGCTTTCACTCTCCAATCCAACACCCCTCTGTACATTTTC

TTGTTTTCTTTTGCCTTCTCTATCTCTATTATGTTGTGTGTTAGTGTAATGGTATCTGGT

AAAGTGGAAAGAGTGCTGTAGTTATTCCACCAGTTTTCCCAGTTTGGTTAGGCAATCCCT

TTCTTGATTGATTGAGATGTGGATTGGGGTATCGATTCCCTCCGATAATTATCAAAAGAA

AGCAACCCGGAGCTAAAATTGATGAGTTTCAGCTTTCAATTCCCATCAATATTGAGTTTT

TTTTCGTGTAAGTGTACTGTGTGGTTTAAGGAATTAGGTATGGTAGCTGTAAGTTTTTGC

TTGAGCTGCATTTAGCCCTAGTGCTGTAATTGAAGAAAAAAATGTGCCATGTGGACTTCT

TGAATTGCTAGGCCAATATGTCTTCCTTGTGTCTTTGATTTTGTCGAGGGTAGAAGTCGG

CCCTTTTTATAGGGCAATTTGAGATGGTCGTCTAACACTCACTGCCTCGAGTCCTCTGTG

GGAATTCCTTTCGTTTTGACAAACCTGTCGTCTCTTTGACAGCAATCTACAATTCGGTAC

ATAGGAATATCGGGATAGAT

>g25454.t1/IbHAK2

CTAATGTAACTACAAATTTTGATGATAGGAATATAGGACTGACCGGATCAATCTATGTTA

TATGTAAACAAAAAAAACTGAAAGCTTTATAATTCAGCATTTCAGAACAGTTATCAATGA

CTAATGTAATAGGTCCGAGTTGAGGACTAATGTTATCGACCTATGTTATATCTAAATTTT

TAAAAAAAGTCAAGAAAGCTTTATAATCCAGCATTTCAGAACAGTTGGTCAATGGACGAC

TAATGTAACAATGAGGCCAAGAGAGGCTTCACTTGTGCTATATGCAAGAAAAAGGAAGAA

ACTTTATAACTAGCATTTCGGAACAGTTGGTAAGTCGGCTGTTAATGTAGCAACAAATCT

AAAGATCCATCTATAATAATAAAAAAAAAGTTAGAAACTTTATGATATAGGCCAGATAGTT

AATCAGCTAATGTACTAAATTAGCAAGTTCGGAGCATCCACTTATGCTATAGGCAAGAAA

AAGGTGGAAGATTTATTGGTGGGAACAATATGATGGAGGAAAAAGTGAGAGAGAGAGAG
AAATGTAAAACTGTTGTTTTACCAATTACCATGCACTCCTGAAATGGTAAATAATGATAAG

GCAAAAGGTGTGTTTTTCTCATCAAGCAACCATTGAAATGATTCATTGGGGTAATAATAA

AAACGTGTTTTGCCATTGAAGATCAGAGAGGGAGCTGAGAGAGAGGTGAAAACTTTGAAA

GAGAAAAGGACCAGCTACCAGAAGTGGTTGAAGACAGAAAGGGTCCATGTTCTAATCAGG

CAATCACGTGGGGTTGCCTCTATAAAACAACAAAATCTACTCCCCTCCTGCAACTTCCTC

AATCCTAAAACCATTATATTGCCCACCCTTTTTCCACTTTTCAGCTTTCCAATCACGTTT

CTTGATTTCCTCTCCATCACCATCGCCATTTTTTTGCAGCTAGCTAGGGTGGGTGCTAGT

GTGCTACTACTGCCTAAAAGGAGCATCAAGATTCTGCCTTTCTGAGTTTCTGGGTAATTT

CTTCATACCCTTTAGTGAAAAACCAGTTAAGAAAGCTGACATTTTGAATTCTTGAAATGC

TTGAGCCATATCTGTGTGTTTTGTGTTCTTTCTTGCCTGTGGAGATTTTTTTTTTTCTTT

CTTTTTGGGTTTCCCTCAGAAAGTCAGAAGGTGGATGGAAGTTTGGCAGTACAGTCACTT

ATTAGGGATGTGGGTATAGTCTATATTTTTGTCCATAAATCTCCATTATTTCCGTGGGGC

TCAAACTTGGTGTGATTGGTTTTTTTTTAATGACAAGAGTCACTATTTGACGATGTGCAC

CAGTTAAACCCTAGCCAACAAATGATCACGAGAGGTAAAAGTTTCTAAGCTTACTCATAC

CAAACCGAATAGGTCAGAGTCGAACTTGTAGCTTTGGTTACCAAGTTAAAAGCGTGACAA

ACTTGGATGGGGTTGTCTATGGTGTGATTGATTTTGATAATAATGCAGATGTGAACTGTC

TGTTGGATCATGTGCTTCTGGCTGGCTTTCCATTTGCAATTTCCTGTTTTCTTCTGGCTG

GCTGGGATGTTAATTATTAATATGAAATTGAAAGAGTGCTGTACTTACATTACTTTCATG

GTTGAACCTAACACTGATTTTGGCAAATTTCTTGTTCTCTAACAGCAAACTATCAACATC

CCACCACAAATTTATCAGCTTTAACCCAAATGCTGTATAGTGTTTTCATAATGAGAGAAC

AGGATGAGTAAAGATTAGATTCTGTGCAATTCGTCGGGTTGCTCGAGCTGAGTTTTTACT

TTTGTTTGTCAAGATCAAAGGCTGCTGAATAGCAAAGATGCAAGTGTGCCATGTGAGGGC

TTTCTTTTTTGTCATCTGATGCTGCTGCTCTGTCGTATTCAGCCACGAAAATATCATTCG

TTTGTATATAAAAGCAGCATAATCAGCTCAAAGTTCCATCAAAACTGATTAAATCAGGGC

AGGAATCTGGGAAAAAAAAC

>g12038.t1/IbHAK18

CCATACAGATATGCATATAATAATGCTAGAAACATAGATAAGTCTTGAAAAACTGAAACT

TACCGAGAGTAAAGGTAAGGGATTTTCAAAATCGGAATCGGGGGCCTAAAACCCTAGGAG

GTGTTCGGACAGTTTAGAACTCGGAGGAGAATGGTGGTGCAACTTTAGAAGCCCTCTAGA

CCCTTTTCCTTTTCGAGTAGTGAATTTGGACACGTGTCGGCAGTAGATGAGAGGAGATGG

GTGTCATTATTGGGCCACATATTTTGGAATAAGCCCAGATACAAACCCATTTAATATTTA

CATGTTAGATTGTTATCGGGCCATCGGGCTAGATTGTTTCAGCCACCTGTCTTATTGGGT

TAAAATATTTCGACTTTAACCTTAAAAATTTTCAATTTTTTTGAACCAACCTATTAATTT

CGGGCTAAAATTATCACCCCTGTTTGTGATACAATTTAGAAGTAGCATTATCCATTTATC

CTTTCCCTAATAATTGAATTGCAAATTCACAGTATTTTTCATAATGTCAAATAATATAAT

AAATGGACAGAATATATGTTTCAAATAGGCAGAATACGATACGGAGTATGATTTAATATG

TGGGGACCGCCAAGAAAAAAAAAATCCCAACTTGTAATATTATTGCGGCATTATTGGAAA

ATTAATTAAATGACAGGCCGCAAAAATAATTCAACTTACGTCACATACAATCATTTCCAA

TACACTGTATAGGGAAATAATAATAATAATTTTAAAAATATTTATCTATAATTGACTAGT

TCAAACTATAAAGGTCAATAAACAACTTTAATATAGAGGTGAACTTAAAACCTTGTAAAT

CTTAAAGGAGAATCATTATATCGCGAATTAAGTAGTTGCAGAACAATCATACTAACTAAT

GCTAAAATGCTACAATCACCTCCCATTTCACATTATTTATTCTTATATGATGATATTTGA

TTAGTTTATTTTATATTTGATTAATAAGTAGATTACTTTTTAAAGGTAGGTGTAGTTAAC

ATATTTAAGTAGTCAATAAATGAAAAATGTTATATTTAATTTGTTGAGTTTGTGATTTGT

GCATCTAGATGATGTCAATTGAGAAGATGAACATTAGAGCCATCAAAACGGATTTTACTT

GCTGGTTCAACCCTCTAAAGAGCATGCCTAAAAGGTTCACCCTACATGACTAAGCTGGCT

TTGGCCGCCTATATATATGTATATATAGTAAATGTTTTAATACTAATATATTAGTATATA

TATACTCCTTCTTTCTTATTTTGGATATCTTCATTTTGCACTTTATTATTATTCATTGGT

CAAACCAACTATTTATTTATTGTTTATTTTATTGAGTAATTTTAATTATTTTTAATTTTT

TTTTATTTAATAGTATTTTAATGTAGTTTATAAACATATAAATTTTATATATTAATATTA

AATTTAATATTATAAAAATTAAATTAAAAATAACTTCAGTCAAATCTTTTTAACTCAGAT

AAATAAAATGAGAATGAGTGAATAATAATTATTGAATTTTTTTATTATTTTTTGTATAAA

TGATTTTAGACAAAACTCATTTTGAGCAGATGGAGGGAGGAGGGAGGGAAAGTAAAAATG

AAAATCATGCATGACGGACGATTGCTTAGTAATTCACGCGAAAAAGGACAAAAATGAGAA

AGGAAAAGAGTCTCACGCTTACAAGTAACAACAAACGAAATAAGATTATATTCCAAAAAA

AAAAAAAAAAAATTATATTCCATGTGAGGTCTCGCGGTTTTTTGCAGTGTCCTGGGGTGC

TGACGAGTGACAACCCAGGGCCCGAGGGGGTCCTACTGATCCTCAAAACAAATTCAAAGA

AGTGTCAACACCAGGTTTGCTGCTTGGGTTTTGTCAGAAAATTATATCATTGCATTCACA

GAATATTTTAATCTCTTTTTATATTTATCAATAATTGCAGAGGTTGAGTGATTGGTTCAT

TCTTGATTGGGGAAAGAAGA

>g59493.t1/IbHAK7

TTGTTTCAACTCATTTTGGGTTTTGGGTTTTCTTTATCAAGAGTTGGTGAAGCATGCAAA

GATGAATGGATTACATACGGCATACGATGCTACGATTATACTGAGAATATACGTACACAT

ATACATATACATCTACACTCGCACTCTCAGGTACGGTCTCACGCCATTAAAGCTTGAGAA

AAGGAACGGCCTCTCACTCTCTCTCCACTCCCCTTTTATACTCACGCGTATATATAGAGA

CGATTATTTAATTATTTAATGCATAAATATTTACCAAAAATTGTACGAGGTTGATAATTT

AACCCCTTTTTTCCTTTTTTTTTTTTTATTTAAAATTTATAAAGTTTTTACTAGATTAAAC

TCTACGAGCACGAGACAGGTCAAATGTAGATTAGTCCGGTGATTATATCGAATGTTGTAT

TAAAAATATATATTTTTTTAAAACGATATCCTTTGTGTTTTGTATATAATAAAATATTCAC

AATGTCATGTTTGAATTTGTTTTTCTTTCTATAAATACGGTGAGTATATATTATTTTGTA

AATAAAATTATTATAAACAATTGTAATAAATATGTTTTTAAACTTTCAATCTTGATATGA

AAAAAAAACATCAATAAATCTTTTTTATTTTCTCTTTTAAAACTACAAAAATAATCAATA

TTCAATGTGCGAAAACAAAGGATCTCTCTATTATGCATTTAGAAAAATTCAACAAGTTTG

AACTTAGGATGTGCCTAATTTTGGAGATTTGCATTTGGGACATTAATTTTAAATGAGAAA

TTGAGAGTATTAATTATATAATTTGTAATAATAATAATAATAATAATAATAATAATAATA

ATAGCTTGTATAATTCTATGCAATGAAAATTGTTTTTGCATACAACATCCAATCTTACAT

TCTTACCAACTATCACAGATGAAACATAGATGGAGCATGGAGGACGAGTTCAGGCCTGAC

CAATGTGGATCCTTTTACACGATCATCTCATAGATAGATAGTCGAGTTTATTGTAAAATT

AAAATTACTAACAATTTTATTGTTGTATTGTGAAGTCTTATAAAGGGGTCATTCTTATTA

AAGAGGGGGTGTTCGACAAAGTTGTGGTGAAAATGAAATTGGAAGCAGCGATTAACTCGT

GCTTGGGAATACGACAATAATATATCACGCCTTATCCCTCCAGTGACAGACTATAAACAA

TTGTTCACGAGTAAAGACATCATGATGGGATTTCATGTTTATCCTCCAGTGACAGACTAT

AGACAATTGATCATCCACCGGATTGCTTACAAAAATATTCTATGGGTCGATGTGTATGGT

TTCACGATTTGGCTTAGTAAATGCCCGTCTGAGATTTTCTCCGATGCACCAACATGAAAA

AAAAAAAAAAAAAAATACAAACTACTAAGTTATTAAATATAATAGTTAATATTGTATTTGA

TTCACCTTTGTCTTGTATTGTATTGAAACATTATAGTCTTACCAAACTCACATAAATCAC

AAAGTTATAAAGTTTTGGCAAGGTCTATGCGTTGGGAAGTAAGACATACCATGCCGATCT

TATATTTGTTTTATTAAAAGTTGAACAAGTACCTAACAGAAAACATTTTTTTGTTAATTG

TCGTTTTATCAACTTATAGTCCTAACGTAATTAATATGGTATGAATGTTATTGTCTTTTT

AATTTTTTAAGGGAGGCATGACCATGGTTTGCGCCAAGCATTAATAGTATTTTAGTAAAT

GTAAGATAAATGTCAAAATGATATGTCAAAATGATTTGTTGGCAACTTGCCAACTTATTA

TGTGGATTCACATTACAAAGTCCAAAATACACAATAAATGTACTAGATGTTCATAATTTA

TATATTAAATGCTCACAATTTATGCAAAATAAATTGTGAACATTCAGTAGGTAAATTGTG

ATGAGTCCCGTAGACCCGGGTCTATGTATAATTATTGTGAATTGTGTGCGACGGTAATGG

AGAAAATTTAGTTTGCACAA

>g1704.t1/IbHAK9

ATTATACAATTTTGTTTATTTTTTTAGAAAATGAACCAAGCACAAAAATACAGTTTTCTT

GAATATAACGAAGCACTAGAAATGAAAATGATTTCTGAAAAAATAATTAATTTTTTAGAA

ATATTATTCAGAATTCATTTTTCTGTTTTCAAACACAATCTAAGTTTCTCAACCTAGGTT

AAATTATTTATAATTTTTCATCTAAGTTTCTCAACCTAGGTTAAATTATTTATAATTTTT

CATTAGAGGAGATAAATTACGAGTAGTTCAAGAATCTTTTAGTCAATCGCACGGGAGATC

ATCTCCACACTTTTATCAATTTAGAAAATCAAAAAGTCCCAAACTATAACTTAAAGAACC

TTAGCTTCAAGATTTTGGTGGAGTCGAACACTTACTCTTCTGTTAAAGTGGTTACTTTAA

ATTAGGTAATGCCCACTTGAACTAGTCTAGGGGGCAAAATTGGTGTTTTTTTAAAGATTA

TCTTTGACAATTGAAGTGGAATGCAACCTTTTACCCACTATTATGTTCAAATATATGTTT

TTTAAAAGGGAAAATCCCACCTAATTTTAGTAGTTTAAGATGGATTTGAAAAAAAAATTA

TTTCCAGAACTTCAAGAAGATAAAGAGTACATCTTGATATGTGTTGGCAGATATCAGTGT

TCAGAGGCTTGTTATTAGTTATGATGGGACATTATCTTTTATCAGTAAGGTAATAATTTA

TGATTATTTTTCAATTATTTAATTATTACAAGTGAAATAATTGTGTCAAAAATGAACTGT

TTCCAACAGTTATTAGGTTTTAATGATGGTGGATGTGAAAGGTTTGGCAACTGAACATTT

ATGGTTGAAAATGGCGGCCCCATTTTGTTGATAGAATCCACCTAGATTGGGCTTTGAATA

ATGGTCATTTTCCAGTGGAATGCCACACAAGCTTGAAGCATTTTGAAAATGGAGAAAGAG

AGAGAGGAAAAGGAAAAGAATCTGTAAAAAAAGTTTGAAGGCTGAGAAAAGACAAGTTGC

AGAAATATGGAAAAAGAGTGCCCTTTTTAGCTTTTATGCTTTGAAAATGGAAAGAAAAAA

AAAATGGGGCAAACGTTGAAGCCGTGACCCGTGAAAGGGACTAGCCAGCAATGGTGGTTG

AAGAAAAGGGTCCCGGTGCTTATCACGTGGAACTCCTATATGGAATGTAGTCTACACGTT

ACTGCTACTGATATTGATGATTTCTACTGTTCTTAGCTTCAGCTTTTGGTTTTTTTTGAA

TTCAAACAGCCATTTTTGCCCTAGGGTGTGCTCTCAAGCTCAAGAAAGCTTCAATTTCTG

GTAAATTCCTTGATTCTTTATTCTCTTGTTAAGTGAAAAGGAAAACCCACGGCCACTCCT

CGATCGAGGACGTTTGTGAATCTAGTTGAGAAAAGATCAAACTGACTCAAATCAAGAAGG

TCAATACTCGTCTCCTTGAAACTTTATGGTTATGATGAATTTGGCATTACCCAAAATTTG

TTGATTCTTGAGAGTAGAAGAGGGTCTAGTTTATAGTTTCTTGTGTTTCATTGCATCCTA

AATCTTTGAAATTTGTATGCTTTTTATTGCTTGGGTCAATTGCAATCAGCCCAGCTCATG

TTTAATAAGACCCTTTTTGCCATTTTGCCAATTTGCCAGTTTGAAGATAGTACAAATGCA

TGTGTTATCTCAATCTTCTGGCAGTAGAGGTAGGTAGAAGTTTGGCAGTCTACTCATCAC

TGCTGCATAAACGCGTACACCTATACTATACAGCTGAGGGTATATTATACATGTATGTAT

GTATGTATGGATATTGGGTAATATCTTTTGTGTCTGTGTCCTTAAAATGGCATTTATTTC

TGTGGGCTCAAACTTTTGGTATGATTTGTTTGCTGTTGAATCATGTACTTTTGGCTTTCA

CTCTCCAATCCAACACCCCTCTGTACATTTTCTTGTTTTCTTCTGCCTTCTCTATCTCTA

TTATGTTTTGTGTTAGTGTA

>g34971.t1/IbHAK12

TTTAGCTGTCATCAAACAGGTATTCTTTAATACATTTAATGTGATTTTACATTATAAATT

TAATATATCAATGTTAAATTAAATATAAGAAAAAAGATATAAATAACTTAAATTAAATCT

CATAATCAAATCAAAACCATAAAATATCTCTCTCTCATATAGATACATACATACACACAC

ACACACATATACTTTTGTTATAAAAAAATGTTGAGAAGCTAGTTTGGCATAGAGTATATA

CTTTTATCACATGTTCATCGTTGATTGACCGAAAGATAAAGGAAAAAATTTTTTTTAAA

AAGAATGACCTTTATTACCAACAATAACACTTTTAATGAGTAATGATACACATTCATCAT

AATATTCATATTATGATGTGGTAATATAATATTTAATTGTATTTTAATAATTGAATTGAT

TACTAAATGTATATATTTAAATTTTGGTGTAATCCATCAATTGATTACTAAATATTCATG

ATTTTTCAATGAATATAAAGTTTTATTACAACTTGGGAGTGAATTTAAGCGATAAACTTA

GAAGAGAAAAATAACATCATTCCTTTTTAATCACCTTTGGTATTTAATTATAAATTACGT

ATACTACTTCCTATTATGGTTATTATTAAATATTTCTATATTTGTTCAATGCCAACAATC

TTTTATTGTAATTTATGGATATAACATTATAACTTCATTTTATGGTTATTATTATTAAAT

ATTTTCTATATATGTTCAATGCCAACAATCTTTTGTTCTTATAAAATGTCAGAGGAGTTC

TTCTTCTTCTTCTTTTATTTTTTTTTAGAAAAAAAAAAGTTAAGAGAAAGAGAGAAAATC

AACTAACCCAGCCTACTATCAACGTAACATTGGGTATTCTCCTGAGATTTGCAACCCCAA

TAGCATCATCAATCAATACTCAATCCACCTTCGTTAAAGGAAATTCTAACAACCGTAAGT

CACATTCATATCTGTTCTTTTCTTTTCTTTTCTTTTTTGATCTTGATAGTTAGTAATTGT

TTGTGATTACTTAATTTATGAATATTTGCTCCACTTTAAATAAATTATTAATTTATACGG

AGTATGTGATAACTTATAAATAATAAGCATCCTAGGATAAAAATAAATAATTCCTCAAAA

AAGGATAAAATAAATAAATACTCCGTAAATGAAAAGCACGTTGACTTGTTATCTTAATAG

AGAGTAATTAGAACATGAGAAGAGTGATTTAAAGTACAGTAAGCAACGTTAAATAACCCA

GATTTGCAGTCTTTAAATCACAAGGTTGTCGCATCTTGTAATGATTCCGCGAAGATTCGT

ACAACTGACAACGACCACTCTCCGACTCTCTCTATCTCGTCCCCATCCTCTGCGGCTCTG

CCCATCTCTCTAGCTGTGTTCATCAAACAAGCTGATTGCGAGAGAGAGAGAGAGAGAAAA

GAGAACATTTTTGGTTTGGTAAAATTTGAAGTGCAGAAAGAAGTTGCCCTGAATAATTAC

TATTCTCAGAAACTTATTTTCCGTCCTTCTAAGTTCTAATCAAAGCTCGTTTCTTGCAAA

AAAAAATGGGTTGAAAATCGTGAGCTAAAAAAAGCAAGAAAACAAACGGGGTTCATTCAT

TTCAGGCGATTCAATCCTTTTGCCTATTGGGGTGGCTGTGGGGCGTTGTATTGTGTGGTT

GGAACTGATTGGAATTGGGGTTCTCAGCTTGACCCCACTGGGGAAATTCCTGTACCAATT

GCCGAGTAAAAGACAAATCTTTTTTTTTTTTTTTCTTTCTATTGAATACAGGTGGATGT

GAGGTATAGTGCTTGGTGTGGTGTGGGGGTTTTTGGTAAAAGTACCAGCTTTGCTCTGCA

ACTTTTTCTTGGATGCTTGTAATTAATTGAGTGGGAATTCTTGTTGGATTTTCTTCTGTT

TAAGCTTGTAGATATCCGCCTTCTATAGTTCTATCACCAGTGGAATCAACTTTTTTCTGT

AAAAAACAGAGGCTTCTGCA

>g5020.t1/IbHAK1

GCCCAAAGACACAGAAGAAAACAAGATAACTCACGTTATATGTGCATTACGACTTTGATT

AATATAGTTAAGTAGATTTGTGCAAGTTTGGTTTTCAAGTCGAAAGTGTAATAGCGTGAC

TTATTATTTGTTTAAATTTAACTTAATTAATTGCCAGCATCTTGAAAAACAGTTAAGACA

TGTCATAATCAGGTGCGAACCAGTCTTAACCTGCGAACCACTACATGATTGCACCGCAAC

TACTACATAATTGCACCTCCCAGACATGCTTGGAAGACCTTTGCGGTGCAATCGTATTCG

GTGCAATCATGTAGTAGTTGCATTACAATCCTTTAGTAATTCGCAGGTTCACAAGTTAAG

GCTAATTCGCACCTGATTATGGACCGTTAATATTACCCTGAAAAGGATGACCGAGTAGAT

GGAGCATGATTCTAACCATATTTAAAGGTCACGGTTCGATTCTTGTCAATACCTTCTTGG

TTGAGCCTTTCAAACGAAATCTTCCTAATGCGATTTATTTCCTCGATATACACCCTCAAA

TAATGGTTATGAGTGTTCCTAATTACTCTGGTGAATATGATAAATAATGCATATATTTAG

CGTAAGTTTTTGTAACCAAAGCTTCCAAATTTAGGGTTTAAAAAAAGAGTGAACCATATA

ACAATTGACAAGTTTATAATTGCTAATTCAATATGTTATAACTACACCGAAATTACAGAT

AACAATAATATTGAAATTTATAAACAAAAATATATTAAAATGCAAGATAATATAATAATC

AACGTGTGAATAACATTACAAATTAACAATAACTACAAAAATCATTATTTGTATCTGTTA

AAATATATAATATAGTTAACTTAAACATAAATAAGATCATTGTGTAAAGGGTGAATTCAA

TTGATAAGGGTATAATGCATATACCATGAAATTATACTTTCAAATGGCGATAAAACACTA

TTTCCTGGGAATGAAAGCTGTCTTTCGGATAATTTCACGAAGCTTTGTCCTTATCGGCCT

CCAAATCTTATCGGCCATTAACATAGACATCCGCTAAACCGATGAAAATTTCGCCATCAT

TAAGCAGAAACCATGTCATTATTACTTGTTCAAACAAAAGACAAACAAACCAATTGGTAC

GGCCATGTACTATGGTAACCTCAAATCTATAATTACAATTATTAATTATACAAAGTCATT

ATCTATTTTTTAAAAATCTTGTCCAACCATAATTAAAATATCTTCCTTAGTTCTGAAAGA

GACAAAGAGTATCACTAAATTGACCACAAAAAGGGAAATTTTCTACCAAGGCTCACCTGA

TAATATATGAGTAAATTACTGACGGTGACTGTCATTGAGTGAAAGGACCTGCGTCTTTCG

CTATCAAGGCCCACACATCCGCTCGATAGTGAATGAGTAAATCATGGTGACTCAACAACC

GATTAAGTGGGAAGATCGTGTTTGATAAGCACAAGTAGCTCATCACATTAGGTGAAACTC

TTACACAATAATTGCCAATGCTCGAGGATCACTAAGTTTGGGACTACGTCAGAAGACAAA

CTCTTGTATATATGTATTATGTTAAAAATTTTGAGTTAAACTTTTTAAAAGTAAGAATTA

AACTAGTGATTCGAGTTGAATCCAGATTAATTTTACAATAATAAACCCATTGAATGTTGT

TTGGAACATAATTAAAAAGTGTTCGAAAAATGGTATCTTTGGGGATTAAATATACATATT

CCTAAATGGACTGAGATTATGAAATTTAGGATTGGACATTTGCTAAAAGATAGTGGTTTC

TGGGGGGTGGTAGGAGGAAGGAACTAGGAAGGAGCATTGAATGCATTCTGCAACTTCTTT

GACCACACACAATTGCCTGCTTACTTACTCCCACTGCTCACTTCCTACATTCCCTCCTTC

TACATCTATCTTCCATTCTTTATATTTGCCCCTTTGAATCCCCGCCCACAACACTCAATC

TTCCACTTAGGTCTTCACAC

>g20629.t1/IbHAK4

GAAACTACTATTGGCTATTGACCACATTTTTGAATTTTGAGGCATTGCCAAATACCAAAT

TGCCAAATACCAAAAACCTGAAAAATAATTTCCTGAAAATAAAAATCATTTTCCAGACTA

CCAATGACACCCTAATTCAGATTCAGTTTGGGACATCCCACCTATAGTTGATTGACAAGT

TCTTGAGACAAGTACAGTGTGGCATAAAGTATTAGGAAAGACATGGTAAAAGCATTGGCA

AGTTCTTGAGACAATTCAAGTCACTCAACTCAAACCCACCATCACCAAATAGCTTTAGTT

TTCTCAACACAGCATAGATTATATATTCCTATCTTTAATGCCACGCACTGAACTGCCTGC

AAATTTGCTACGGATTCACTCAAACACCACAAACTTGATCATTAGCATATATTCCCCATG

CTTCAAGCCTTCAACAAACAGGTGACGATGGAAGAAAGACGCAATAACTACACCATAAAT

CAGGGTCTACTTTACAAGGTGGATTCGTGTCTATTTACATATTGAATGACAACTTACATA

CTGATACTCACAATTTGAATTGTGAACATTCATCAGTATATAACTTGTAAACATTCAGTA

TGTAAATTATGTATTTTGAACTGGGGTACACCTTGAGGGTCCACAGAATAATTTGTCAGA

AAGAGGATGGGGGGCCTGGCCCAAAGAATATGATATGCATCCATTAGTTGAAATGAAACG

GGAAAGTTTAAAATTAATTCTTTAATCGTGGTTTTGGTTTGCTTCCTCAAAGTAGGGGAA

TATTCCATTAACCAGCAAGATGATATTTGGATAGTTTCTGGAAAATTATTGTTGACAAAA

ATTGAAACAGAGAAACTTTTATTTTACTTTATAAAAGAGGAATTGGCAAAATTTTAAATA

TATTAATTTTGTGTTATGTTATATGACAAATGCTAATGCATCGATTTATTACGGTTGTTA

TGTCATGGATCTGGGTCCACCTTGCAAAGTGGACCCAGGTTCATGTTCACAATCTTAATA

CTCTATATTCACAATTTTAGAATTTTATGTTTACAGTTTTTGAACTCAAATGTTCACAAT

TTTTTTTAATTCTATATTCATAATTTTTGAACTCTATCTTCACAATTACAAAACTATATA

TTCACAATTTCTGAACTCTATGTTCACAATTTCAGAACTCTATGTTCACAATTTCAAAAC

TCTATGTTCACAATTTTATAACTTTATATTCAATAGTATAACACAATTTTTGAATCTATA

TAACAAAATTGTGACTATAGAGTTCAAAATTTTAAATATTGAGTAATACAATTTTGTATA

TTGAGATTTAAAATTGTGAATATAAAGTTCTAAAATTGTGGACTTAAAGTTCTAAAATTG

TAAACATGGACTTGAGTCCACCTTTCAAGGTAGGTCCATAGCATAATTTGTTGATTTATT

ATATGTGCCCGATACAATTATGTACTTTACATTTTACACACGATTCACTAAGTATATTAA

AATCATTGTCAACAATACCATGTACAATCATGTATGATGTATGCATATATATGATGGTAA

TCATGGTATCAGACTATCGGCGTATCATTTGTACCCTAAGAAAATTTTACTTTTGTGTAA

TAGTTCTCGAATCACATAAGATAGAGAAGTAAGCTGTATTAGAAAGATTTTGCGTGAAGG

TTTGATCGATAAGGTAGGTGTCGACAAGAATCAAACTTGTAATCTTTTACGAGAACTATG

ATTCATCTACTGGATCACCCTTTCGAGTCAAAGGGATGGAGTTTTTGGAGGTGGATAGAT

GGGCGGTGATTAATATGATTTTTCTATCAACTGTTGGGTCACATTAGTTAAGTGCTGGTG

TCAAATGATTTTGTTCGTACCCCAAGTGCCAAATCACAGTCCCAACAAGTGGTATTATTC

TGTTGGCTATTTAATCTCATTTCTTCTTGGCTTATGTTATACATCTCCGTCTAAACATTC

TTATGTAAAAGGTGGTCGGA

>g12041.t1/IbHAK6

TGCAGGTACATAACACATTACTTGCATGCAAATAATATATTTACAGCACATAAAATGAAT

AATTGCAGTACATAATATACATAATATATCAACTGTTAACAAATAATTTGAAAATTATTA

TGAATTAATGTCCATATTACAAGATAGACCATGATTTATGATATACGAATTGCGTAATCT

AAGGTGAAGCTAATCCTAAACTTAGATTACTTTTAGGCTAACTCAACATAATCTTGACTA

AATACAGGATGTGAGTTGGGCTCAAATCAACCCATGTGCCATGTAAATCTAGACCACATA

TATTGTTATATACGAGTAAGTAAGGCTAATTAATACATTATTAGGATAAAGATATTAAGA

AACCTAAAATAATTTTATAAAATAAAAAAGAGAAATAAAACTCCCAACCCCTTTCCACAA

TATAAGAAACGACAAGATGACGGAAGCAATTCAAACAAATCCATTAGAAACAACATAAAA

AATTCAATGTACCATCCAAATGGACAACATACATCTTAACTTAGACTCATACCTCACAAG

TCTCACCATATTTAATGATATTTACTTTGTTTTAAACACCCCTACTTTTGAACCCAACAT

ACTCCATAAATAAAGGTAAATTTATACACATATTAATATATTACGGATACAACTTTTACT

TTCTCAATTTAAAGTTTGACTTTATACAATTCCAATTCGATTAGGTGTCTTAATTCAATA

CGGAGGATTTTTTTAACATAAGGTAAATCAGTTGAATTTGCAAAGGATAATTTCAATATC

TTGACGTGTACTTTATCTCTATTTACTTAAAAACTGTTGAAAAAATCTTGCATAGGCGCC

GACCGGCCGCCTAGCGTATTACCACAATTGGTGTTTTAGGTAGCCGGCCGACTATGCCTC

TCAGGTCTCCTAGTCGACCGATTATCTATGCTTTTTTTTAATAGGTTATTTTACACAAAA

CGATGTCGTTTTACGTAAAATATCCCTAAATTGTTCAAATTCTAGATGTTTTAGGCTAAT

ATTTAATATTAATTATTAAAGTATTAAATAGTTTATAATTATTAGTCTCTAAACAATAAA

AAAAATTTATATAAATAAAAAAAAATGTCTAGGCGTCCGAGGAGCGCCTATCGATTTTTT

GATCATGCTTAAAAATGTGGCAATAGGGTATTGTTAAACTGTATTATAACTTTAACGGTA

ATTTATTATTTTATATTTTTTGTTAATTAGTTATAATATTAAATGCAAATATTGAAATTT

GTGAACATAAGGATATGGAAGCAATATTCCTCTTAACAAGAAAATAAAAATTAAAAGAAA

GAAAAGATATGGAAGCAAATCTTGTAGAAACTCCCGTCTTAATGACAAAGAGAATAATAA

AAAAGAAAGATCTGTAATATAAGGCTTCAAAGCTACAGTCTTTGCATAATCTGCAAAACA

ATGAAAGCTTATTTGAAGTAGAAAATAAGCATATCAGCATACCACTGGGCCAATACTCAG

AACCCATTATAAACTATCGCAAATACCATTGAAAAGGAAAGGTAAGATAAGCCACCTCTA

AAGTATAAGCACTGTATCTCACCCCTCCACCTGCCTTCTCTTTTTAGTTCTTATGCATAT

GCACATTCACGATACGCGCATATAATAAAAAAAAATTAAATGGGAAGGAAAGTCATTATG

TCTCCCTCCCTCGCTGTCATAGAAAATAAATTCTGTGATCTTATGCTCTGACTCTCCGAG

CAGTATTAAATTTCTGAGGAGTGTTTTTACAGCAAGCACTTTGGCTTTTTGTATTTTGTT

TACTTTAAAAGGTTGTTCAACAATTTGGATGCTAATATCCCAGTGCAAATACTATCATAT

TCTCCCAAATATAATTTTCTATTCTCTCTGCTCCGTCCATGCGTCAGTGGGTGTTGCCCT

GAAAGTTCCTTTTTTCTGAGTTTGAAATTTCTTGAATTCGCTGTGAAAATGATTTTCTTG

CAGTTGTGAGAGGTATTGCC

>g42476.t1/IbHAK3

GTTACATAGTCTTGGCGTTTATTCAATGTACACCTCTTAAGTATGAGTTTCTATCGTCAT

GAAAAAAAACATAATTATATTTTCATTGGGGCTCAAACCGATAAACTCTCATTTAGGAGA

ATCACTTCATGACATCATATCATAAGTTATTAGCGCGCGCGTACATATATATATATATAT

ATATATATATATATATATATATATATATATATAAAATTTTACTACATAACTTTAGCTAATTT

TATATTGCAATTAACTATTCACGGACATAATGAATAAATACGAAAAGTTGTGTATGCTTG

TCAAATATCACATAATATAATCAATAAGGTGGCTAGTGGGCTATGAAAATATTAATAATT

CCAACACAAAAAATGGTAGTGACGTTGTTGTTGTTGTTCTTATCATAACTGGAGAGTAAT

ATTTTATGGAATGCTGTTCAATTTAATTTTTTTAAAATATTATATTAAATGATCTCATTT

ACGACTAATATTGTAATTTTATGAACATAAAATTATCATGATTTTACCCTAAATTATCTA

CTCAAATCCCTTACATACACTGTATTCTTTTGAAAGTGAAGAAAAATAAAATTTTTCGTC

TCTTAGTTGTGACCGATATGTAGATCTAATCCATCAATTGTTTAAAGTATACATTTAATC

TCTTAATTATTACTAAATTGGTAAATTTGGTCCATTAATGACTATATTTGTCTCTTTGAT

AATAACTAAATTTGCATCACAAATAATTGAGAAACCAAATATACACAACGATTTTAAGGA

AATGCCTGAAGATATTATTTTTCATTTAATAAAATGACTTATTTGGTTATTGATAGATCA

AATATGTCACTTCAATAATAACTAAATGATGGAGGGGTGCTCGACAATTAGCTATTAATT

GTTTTAACCTATTAGTAATACTGGCGTTTTGTATGGAAGAATATTATAACTGAGAGATTA

AATATGTTATCTTTTCTTATTTTAATTCAATTAACATTGCAATTTCTAAAATCATTTTTT

ATAAAGAATAAGTGAAAATTGCGATTTAATTAAAACTCTGACTCAATTATTGGACATAAA

AAATAAAAATAAAAAAAGTGAAGGGGGCCATTTTAAATAGTACGTCAGCCTTCAATAATA

GTTATACGGAGTAATATAATACTATCTGCTTCAGTATACAACTATACACCCACCCGCCAG

TCGCCAGATCACACATAGATGAGGAAGCTGCGATGCGTGAGCGTTGGATAATTACACTCG

CCACCACCCGCTTCAACGCGCCGCCCCTCTGCTGCCGCCCTATTCCTCTTCCATTGCCGC

CGATTCCTTGCTAAAAAAAAGTTCGTTCCCTCTATCACTTCGCCTCTTGGGAGCTGGGTT

GTGTTTGAATGTCGCTTGCAGAATTGAGGATTTTATTTGGGTTTATAGTTTTTTTTTTTT

TTGCTTACCGGAATTGCTTTCCTTTTTGCGTATTTTTTGTGTTGTTTTCTCATTTTATCA

CTGATTTTTACTTCTGCCATCAGTTCTGGTATTTGCGTTTATCTAAGTCTGATCTCGTTT

GCTTTTTGCAAGTTCGATGCTTCAATTTGATTGCTTCTTTGAAGACACAAGTAGATTGTT

AGCATAATAAAGCTTAGTAATTCCGTTTCTTTTAGCAAGAGGGATTTAACTTGAGTCTAC

TCAGAGGTTTCAGGGTGTTTTGTATTTAACTAGGCTTCTGCTTGTGATGAATAACTTAAA

TTGTACGATACTGCCTTCCTCCTCCCCGTTAATTAATGTTTTTTTTAATATGTATATTAT

TATTTAGTTATCTTAACTCAGAATGTACAGAATGTTGAATTTGATTGTTTTAGTTATCAA

GTTGACATTATCAGTCTTTCCTTAGCTTTTATTGCTAAGTTTTTATGCCAAAACTCACTT

CATTTTTTTTTTGGGGTGTTTCTGTCAAATCTTAATTCTGTTTCAAATTAAATGCTGAAA

AAACTGTGGCAGGTGTAAAG

>g30149.t1/IbHAK10

CCAACTACTTTTTCTAAATATTAAATAGTATTAAATTTATAAGCTTCAAAGAAAGTAATC

AAGTCAAAAACTTGTAGTTGTTAAGTCGTGTTCGGGTCTTATTATGGTTAATAATAATAA

AATTAGTTCATGTAATAAAAATTAAATTAATAATAGAAATAAAAACAATTTAAAAAATTA

CTTTTGGGTTTCTTTTTGGTGCATGAGATGGACAATATTGATTCACTTCAATCAATTTTT

TTAAGCACATGTGTCATATCGAATAATCTTAGTCAAATTAGTTTTATAGTTGATTTGATA

ATCACAAAAACGTTGAATTGGCCGGTAAGGTTAATACGAATCCAAATGCTTTTTGAAGAC

TGCTATAGTGCTATACTGCTATTAGACAAATCAATAAAACAAATATGCACTGCTATTAGA

AAATTGAATAGTGCATATTTGTTTTATTCTTTTGGGAAGAATACAAGAAATATGCACTAT

TCAATTTTCTTTAGATTAAAATATATAATTAATATGAAAATATCTATATTGCTTCTGTGT

ATTGCAAGTTGTGTTTATATACGTTGCACATTTTATTTTGTTGTTAAAGGAATATAATTG

AGGTAAATTAGACCAATAATACGCATGAGATTAATAGTTCAAATTTATATAATTATATGT

ATGTAATAATGTTCTTGAAAGAGTTAATTCTAAACATATTTAACTAATTTTTTTGACAAA

TATAAGTACAAAATATCATGTATTTAATATTTGTACACAATCTTTCATTCCATCAATTTA

CTCGATTATCAAATGAGTTGATCCCTTTTATCCTATCGTGTGGCCAACTTTTTTGGTTTC

ATACCAAGTTTATTGGTGCACATTAACAATATTAGTCCTAGGAGCTACCGAATTTTTCTT

CTAACCAAATGGTATCAATAAAAGGGGCAAATCATTATGTGAACCGGGGTCCACATTTCA

AGATGGATCTGAGTTCAAAATACACCATTTACATACTAAATGCTCATAATTTATATACTG

AATGTTCATAATTCTCACTTGAATTGTAAATATTCAGCATGTAAATTGCTATGAATTCAC

TTTTCAATGCGGACATGTGTCCATGATGTAAGTATCGAATAAAAGGAATACATATTATAT

TTTATAGAAAAAGTTGATGTGTAAAAGATTAAAAGGAAAAGTATGGATAGTGAGCATACG

ACCCAGATCAAGTTGGGTATTCCATGATTCCATCTTGCCACGGCAGAACGTTGCTTTCAG

TGTCCCGGACTATAATTTAATTTAATGAATGAAAAAAATCTCATTAAAATTTTGTTTTCA

GTAGGTAATAGTCTGATTCCTATTACAAATATTCAAAGTTTAGCTTGTATGGCCTCAATT

GATGGTGAAATTTAAGAAGAAAAAATTAGTAATTCAATCTTATCGATCACGGTAAGAACAA

TCTCTCTCAAAATAAGGAGTTCTTTGAAAGGTCTCGCCTGGGATAAAACTTGAAAAAATG

TGGGTAATGATAAAGAAAACGTTGCTTTCAGTGGCCAGCTCAATGGATTAGAATATGGAA

AGGTGGAGAGAGTAGTGGCGTAGTGCTGCACTGTTGCCTGTACCAGTTTACTCATCACTG

CAAATTGCAAAACGCAAACCCAAACCCCACGTTTAAGGTGGCGAGGCAGAGAACCCAAAC

CTTCCCCACTATTCTACAATCTCTCTCTTTCTGTCTCTCTCTATATATATATATATTGTGGA

GTAATATTTTGAGTACACGAGGTTGAAAATTTGAAATGACCCATGGCAGGATGAAGTCTT

GTCCTTAGCTGAGTGTCCAATACAACACTCCAATTGCATTTGCACGCAAGACTAGGGGGA

AAAAGCGCACACACGCAAAACATATTGCCACTCATTCTTCTATTGCTCAAATATTTGGAC

AAAAAAAGGTTCATAAATTCTCACCACCACACCTTTCTATCTTCTTCAGATAGTACTGTA

TTTTCTCTGCATACTTGTTT

>g40997.t1/IbHAK16

CACTTGAATAGACTCTCGGTCTGAGACATCCTTTATATATATATATATATATATATATAT

ATATATATATATTTGTGTTAACCATTATGTTTAATTACTATTAACAACTTACGTTTATTC

TATTTTTATTTAAATTTATGTTTTTAATATTGAATTATTAACTACGGAGTATTTCTTTAT

AATTATAACATTATTAATAATTAAATAAAAAATTAATAGTTAAAATAATTAATAATAATA

ACAACAATAACAATAACAATAACAATAAATTGTAAAAGTAATTTAAAATAAATAATACGA

TATATTTATGCCTTTATAGGCCATTTTACATTTATGTGTGCAATTTTATGCTCATATAAA

CTTATCCATTAGAATTTATTCAATATACAAGTTAGTATATCACCAAAAATTAATCCACAA

AAGCTTGTTCATTATTTTGTTTGCATATATCCAATATATAAATAACTTTGTGTATATTGA

TTAAAGTTTATTCCATTTTTGCAATTAAACTTATCCACAAATGAAGAAAGACAAAGGCCA

GATGCAATTCATCGTTTAATTTGCTATTTATTTCCAAATATAATTCCAATTAAATTAAAT

CCAAAAACTTCAGTGTATATAATTTAATTAATACATCACCTAAAACTGATTCGTGGATGG

ATCTTTATTAATGCATTTACTATTCAAGAAACTCTTCTAGTTCTCTCTCTCTCTCAAAAG

CTTTTAAATTGTTTTTATCTTTAACTAAAATTTTAAAACCCATTATTTTGAAAATTTACT

TTTTTCTTTTTGTTATAATAATATTTTCAAGAGCTAAAAATTGGGTAAAAGGACTAGAAA

AGTCGAAAAATAGAGGAAAAAAGGTCAAATAAGCCCCTCAACTTTACTAAAAAAATCAAT

TAGGCTTAAACTTTCAAAAGTTGCAATTAAACTCATAAACATGATATTTTGAAGCATTGA

ATTCCAGAAACTGATTGGTAACCTATAATTACAAGTTACCTAGGGTTCCAACGAATTTTA

ATATCCACAAATATCGTAAATATCAAAGGATGTAATACCGTAACTTATAGAAATATGAGT

TACGATATGTAAGGATAACTTAGGGCAAAAGTAACCAGGACCAATACTTATGAACGAATT

CTACAATTACTATATAACTTTGGACAAAAAGTGTAATTTTTCCTTTTAAAATTATAAAAT

ATAATATTAACCTATTTTAAATAGTTTGAAACAAAATTTAATAGTTTTTTTAAAAAAAAA

CAAAATTTAATAAACTCAAAAAAAAAAAATATAAAATTTGAGATAATAAAAGTGAAATTAA

CCCCCAAAAAGCTATAGGTAAAAAGTGATTTGGCCATGAAAAAGCAAGTCGCCATTTTTA

CCTGTGCAGGCAAGTGTGATATCATATCATATTATCATCTCATCTCATCTCAGACAAGTA

AGCTTATTTTCAAGAGGGAGAAAACATGAACCCAAACGTAATCCAATAGGAAAAAGAAAA

AAAATTAAAAAAAAAAAAAAAAAAAAACAGCAATTGAAAACGAAACGTGAAAGCATAACT

CCAGACTTCACACCCGTATCTCACTTTCTTATATCTGCTGCAATCAAGGCAAGAAAAAGA

AAATTAAATAAAATTTGAAAGCAGTAAAAGATTGTGATGTCTTTCTCTCACTGTCATATG

GGAAATATTCTCTAATCTTATGCCCCGATTCTCCAACTGGCTTCCCTTAAAGTTTTTTAC

TGCAAAAAAATTGTGGCTTTTGGTGTTTGGTTTTCCCAGAGGAGGTTGTTCAACTGCTATT

TGCACAACTGCTATTATTTTATCTTGTTTTTGTGTATAAGAATATAAAAATTTCTCTCCC

CTTGGCTTTGTGCTCTGCGTCAGAGTGTGTTTTTCTTGAAAGTTCTTTTCTGGGGTGGGT

AGTATCACCAGATGGGTCAGCTTTGAAGTTGTTGAATTTGGTGTGGGAATGGTTTTCTTG

CAGTTTTGAGAGGATTTGCT

>g36794.t1/IbHAK11

ATGGTCCATGAAGATACCGCCGAATATTCGAAACTTTTTGTGGCGATGCATGCAAAGTGT

TCTTCCTACCATGTCGGCTCTCCATTCTCGTGGGGTGAGTGTTGATCCGCACGCCTCTTT

GTCGTCAGAGCGCTTCTGATTTAATGTTTGAATGTTCGCAAGTTGCACTACCGAATGACG

ACTCATCTCCTTGATGCTAGTTTGCCATTTGCGAGTTGGATTGAATCTGTTATTACTACA

GGCCCAGGGACACTGGTTCTCCGAAGCGTAGCAAACTGCTGGGGTATTTGGAGGGGACGG

AACGACCAGATATGGAATAGTAAACCTTGGTAGTCTCATTGGCAAACCGATCAATGCCTT

TATAAGTTGGCAACAAGTGATGAGTGAGGCTCCAAGTGTTGTTGGTCAATCCATACCCCA

TGGTGAAACTGTCACGGTGGGTGAGGGAATATTAAAAGTTTTTGTTGATGCAGCTGTATT

TCCTAATTCTGACCATGCTTATTATGGGATAGTTGTTCTGAGATGGTGGTGGTGAGTTTG

TTCTTTGCCGGGAATGGCTCTCTAGTTGTCATAAGGATGTTCACAAGCTAATGCAATAAA

GGAGAGCTTTGTCATGGCGAAGGATAGAAGCGGAGGATTAAGGTTTTCTCAGATTGTCAA

ATGGTTTGTCAACTGTTTGAGAGGTCTACTGATGATTTATCGTACACTGGATGCGTTGTT

GGTGATTGTAAGGTTTTACAACGACACTTTGAAGTAGAGTCTATTGGTTTTATTCCTAGA

TCAGTGAATAAGCTTGCCCACGCTGTGGCTAGGGCTGCTCATTCTCAACCTGGTCCTCCT

ATTTGGATGTCTTCTGCTCCTTTTTGTATTTCGCATTTGATTTAATAAGAATCTCTGAGT

TTATTTTCAAAAAAAAAAAATTGAAATTAATTAGAAAAGAAATTATGGAGAGTTTTTTTTT

TTAAAGAAAAGAAATCAATGGAAAAAGTTAAGGAGGTAATCTAGGGCTTTAATATGATAT

TTGATCAAAGTTGAGGGGTTAACGATAAATGTGCTTATAAGTATGTAAGTGGGGTTTTTC

CAAAAAAAAAAAAAGTATGTAAGTGGGGTATATCTAGAGAAGGAAATGTAATTTTTAATA

ATAATAAGAATTATTATTATTATATTATTGTTACTATAAAGATCCGCCAGTGTAGCA

GACGTGTCGCATCTGGTAGCGGTAAAAGAAAACAGAGACGACATTTACAAACTGTAACTA

CCGCCTCTCTCTCTCCTCCTCAGTCCTCCCCTTCTGTCGGTTTGATCTGAATGCTTCTAC

CACCGAGAAACACAGGAAGTTATGTTCCTCTTCTCACCTCTCTCGTTCGCAGTTTTTTCT

TTTCTGCAACTGGGTCTTGCTATCTGTCTTCGGGTTCTTTGAATTTTTAGGTGAAACAGG

ATAGAGTGGATACGGGTATTATTCATTTCAGATCGATTTGGTTCAGCTTTTGGAGCTATC

AAAATTTTCACAAACATGCAGAGATTGAATTGTATGCACTGATTCTAACTTCTAAGTTGG

TGAAGCTTTTCAAATTGGGTTTGGAATTTTGTGCGGCTGATATTGACTGTGGTTTGGGTT

CTAAGCTTGGCCCCACCTGAGAAAAATTCCAGTGGCTGGCTGCCAGCTATATATCCAGTA

AAAACCAAATATTTATAGGCATACATACAAATATACATACGTGTATATAACATACACGCA

CACATACATACAATGCATGTGCGTGTAGTCTACTGCGTTGTTTTAACTTGTTTTCCAATT

TTGGGTTTGGAGTTTATGGTAAAAGTACCAGCTTTCTCTGAGACTGTTGGGTGGGTTGTT

GGAATATAATTGCAAGTTCAATTTGAATTGGTTCAAGTAATTGGGCTGCTGCCTGCTGGT

CCTTGAATTGGATTTTCTTCTCTATTATATATTATTTTGAGGGATAATCAGAATCAGCAG

CTTTCCGGCCTTGAAAGAAA

>g32726.t1/IbHAK17

TTGGAGCTAACATTTCTCCGCAAAATCCTTGATTTTGCAGGAGGAAACTAAAAAAATCGC

ACGTAAGCTTTGGTTGCTTTGGCACGGCCGCCATGGCCACTCTGACTTTATACCACTTTT

TCATTTTTTTTTTTTATAAATTGTCAAAATTTCATTTGATTTTCTTTTTTTTTTTTTTTT

TCATTCCAACCCATTTTCTCTTTCCTAATCACACTTACAAAAACCCCTCAAATGCTCTAA

CTTCAAAAAATAATATTTAATGTATTTAAATGTAGTTTATGTCGCTAGTCTACATTACAA

ATTTAAAATGTTAGGTCAATGGTATAATAATTGCAATCAAACTGCACGGTTGAAACGTAA

AGGCGGAAAGCCACGCGGAGCCTATAAAATATGCTCATTTTCCAAATCTAAATCTACCCA

TCAGACAGTGACAGACAGGGATGTTAAAATGGAATTCACACTTCTGGGAACCGCAGGCCG

TCCAAATCTGCATTCAAAACAAGAAGAAAAGGATGCCTTCTTTTGACTTTCTACTTCCAC

AAATAATTTTACGACTACTACAGTATATGCCATTGTAATATCAACCATTCCTTCGTCTAA

GCAACGAGAGAAGATCCAATACCTTTTCTGACACTTGTAAACCAGCTACATTTTCATGAA

TATTTATATATGTAAAGTCTATAATATCTCCCTTTCATAGTTTGTGTCGTCTGCTACAGT

CCCTAGTTTTAATCGGCAGTTCGGCACCCAGTGTTTCTGAGGCTTGAGTAGACGTTTACT

CAATATATTATAGTAGGACTAGTAGCTCGTTTCAAGAAAATCAAACTATGGATCCTCCAC

TCAGCCCTGTGTCTCTCTCTTATCCTATGAACAAGGTAATCAACTAGCTCTCTCTTTCTC

TCTATTATGTAGGTTTGTTTCTGAATTCTTGTTATGATGACTGTTTAAATATTGTATCAT

GGCAATTGGTTTCTATCCATTTGTTGAGAAAACTGGGCGTGGAGTTGTAACAGTTTGAAT

ACGTTTCTAATTTTGGGTCCACAAGTGATTTTCAATTTCTGTTAATTGTTGAAATCCTCA

GTGTAAAAGATTTTTTTTTGATGTACAGTCTAGCAGTTTTTCCAGTTGATTTATGGACTC

TGACAAATTCTAGCATATGATTTTTGAGGAGAAAATTTGAAGTATAGTTATTGAATACAT

TTTTAATTTTGAAGTATTGTCTACATTTTTGTTAAAATATAATACTTTTCTATGCACTAA

TCAAAATAATTACATTTTATCTAAAAAGTAGAACACGTCTCATTAGTCTCACACAAGACT

CATTATTAATTTTGTGTTCAAAGTGATTTTAAATTTCTTTAATTGTTGAAGTATTTAGTG

TAGAAGAAATTTTTTTGATGGTTTTTAAAGTTGGCTTATGAGTTTTGGAGTGAAGAGGTG

TTGGGCAAAGGCCAAAGGGCCAAGTCCAATAATTGGTGGCTAGAGAGATTGTTGGGCAGA

GATTAGTAGAGATCCAAGTCCAACACCTTGCCTCTCGAAAATGTGATGGCGGCCTATAAG

GAAATAAATTTGCGTCTTTGCTACTAGTTATAGCTTTTGGCATAGTGGTGAGCGCTTGAT

CCTATCAAGTGGTATAGAGCCAGGTCAAGGATCAAGTCCATTAATTGGTGGCTAGGGGTT

CTGCCTCTATAAAGAAATAAAGTATGAAATAGAAAAGTGAATGGGCAGAATTGAAATTAT

TGCAAAAAGAAAAGCTACATGGATTGTATGTATTACTCTGGTAGTTCTTACCCCTCAGGG

TAAAAAGTAGAGAGGAATTTCATGTGGCTTGCAGTTTTACTTGAGATATATTTTAATTCA

GGTGGCTGTATATATTTAAGTGTACAAATTCAAAATAGTATCCAGTAAACTTGGGTCTAT

GGTAGTTGAGACTGATCTATGATACTAATAACCAATTCATTCTTAAAACTGCTACAGCTT

TGCCATCAATCACATTATCA

>g17727.t1/IbHAK15

GTGAGGAATCGACCCATGTAAAAAAGAATCATATATCAAAGGTAGACTGATTTCTTATGC

ACTATATTCAGTGTTTCATGAGTTAGTGATTGATTTCTGTTGTGCACAAGTTTGTAAACT

ATAGAAAGGTCAGATGGGCAATGTGATACTTCTTTCCAAGTTAATTGAGGACTTTGGCTT

TTAAACATGTTAGATTAGTATGTGAGACTGATGTTTTAAAACCTAAATGCAAAGCTGGTT

ATCAAGACATGCTATTCATCATGATGTATATGATTTTGTAATGACTAAATGCATGGAGTT

AGAATACCTTTAACTTTTTACTTAAGGTTAGCATGTTATTGGGGGTGACTTGCATTTGTG

CATGCTGATGACTGCCTGTCAAAAAATTCTTATAATGTATAACTTAATAGATTCGGTTAT

GTGACTCCACACTGTCATCACCCTATAAACACATCTCACATTCTATTGTCACCCTATAAA

GATACCTGACATTTTGTCATCATTTCTATGCCTGCCTTGATTACTATTTGGTGTTACTAG

GACTTGCTGATATCAGTCGTAACCATACTTGAAAAGGTTGGTACAAACTTGGTTCTAATA

GTAAGTTTAGTAAAGGTTTATATGCATGCATGATGCATGAACTCTTGACTTAATCAACTA

AATCTTATTCCCAATCAAATTTTGGTGGGCTTTGCAGTTATGACCATGTCAAATTCTCTG

CATCTTTTCATTCCCCAAGAGTTTTTACTCAATAATCACAATTAATTTTTCCTCTTCTTC

CCCTAGGTGTACTGGATAATATTTTTTGGTGTGGGCATGTATGGACTGTTGTATGGGGGT

AGGAGTCACTTAGAAGTTAGAACCCCGAGCATAAGTTATTCATGCTATATAAAAAAAAGT

GCAGATTTATTAGGAGGCAGGAGGCTTCACTAGCATCTAGAACCAATCTCTTTACTTTTT

CTTTTGAGTCTATATCCTTAGTAATATCTTCCACTGGGCTTTTGTGTGAGATTTTGGACA

CAAATACTCCAGAGGCTTCTTTAGGATCTTGAGCTGGTCTTTGATCTTTTCTTTTCCAAA

CTACTATTCTTAATGCCTTCTTGTGTGATCAGGTTCCTGGGTTCCTTATTTTGTTTTCCA

GTTATTATCTTGGTCTATTCTGTGCCCTCTGCTTTTTTTTTTGACCTTGTGAAAGATAGT

TGAACTTTCAAATGGGAGAGGATATCATTGATCTAAGGACACTGGGATTCGAAATATGAA

AATTGCACAAACTATCCAACTGTGTAGTATTAAGTAAAGCCTTTTTATTATTTATGGGAA

CTTACACCTTAAGCTTCACTAGTCAAATATATATCTATTTTTATTTACAGCTGTACAGGA

AAATGTCTGTTGTCCATGGTTGGTTTTATATGAAAGAATTGAATACATCCATGATCCATT

TACTACTTTTTGAAATGCTTGGGATTCTGTGTGCCCCCTTCTGTCAGTTCTGTGCAACTC

AATAGATGTTGTTTCCTAAATGAAAAGTAAAGAAAGAAACTTATGTCATCAGGGATTTTA

GAACAGCTAAAAGCAACCTGATGGAAAACAAAACAAATCACTGATTGATAGTCATAATGT

GTAATATGGAGTACTTGATATTTCCATTTGAAATTTTCTTGCAAGACAATCGTTAGGATG

GTAATGTAGTTACACACATGAATGTCCATGCGTATGTGTTATGTGTGTGTGTGTGTGTGT

GTGTTTTATTCATTTATTTTTGGGGAGGGAACAGGCAAGCTACGCATAACTAGAGACTAA

AGCTCATATGTGTTATTGTCCAGTTTTCTTTAGCCCGTGATCTACTGTTGGCATACCAGA

GTTTTGGTGTGGTATATGGAGATTTGAGCATTTCACCACTTTATGTCTATAAAAGTATTT

TTGAGGGAAAGTTGCAGAGTCATCAACATCCAGACGCAATACTTGGGGCATTTTCATTGA

TCTTTTGGACACTAACATTG

>g46647.t1/IbHAK14

ACAATGCCTTCTGTCTATCTTGGTTCCTCATTGCCTTATTATTGTTATTTTCTTGTAGGT

ATTACTATAGCAGCAGTTTGGGCTACTATTACGATCCATCATCAGGACTGTACTGCAACG

CAGCTTCAGGACAATGGTAAATGAATAACTGATTCACTTTTATTCATAGAAGAGGAAAAT

ACTTGACAGTTCCATTCTGATTTGGTTTTCTCATGTATCCTTTTCCTGTTTCACTTGTTC

AGCTAATGTGGTTCATGCATGTTTATTTAGAACTTATTTTCAGTTATCCATTATTTGGAC

CTTATGGTACAAATTTGACTTTTGAAAAGTTGTTTTGAGCTCATTTGCAGCTTATCAGAA

TACGCTCATTGAAAAACGCTTCATTAAAAGCTGTTTATCTAAACTGTGCCTTGACTTTGT

TGGTTGAATAAGTTGTTTAGTGAGCGTGATACAAAATCTTTCCTCTTGTCACTCATACAT

ACAAATCAAATGTAGGTATTCATATAAAGAAGAATCTGGCACATATGAAGAGGTTCCGCA

AGCCCAACCCCAACCCCAACCCGAAGCAAGCTGAGTGGAAAGCGAGTACAGTAAAAGTA
CAGGCAAGAAAGCATGTGATGGATGCAATTGCAACCACCACATCCCTGATGAGGTTTTCCA

CAGGCAGAGATTTCCCGACTTTCCTCATAGCGTCTTGTTAAAAAGATTTTTGTGGTAGAT

GTTTGAGTTTTGAGTCTGGTACTTTATCGACTTAAGCTGCTGACTAGAAAACTGGGTAGA

AGGTAGAACGACGAGAGAGACACTTGAATGACATATACATACTAGATTACATATTTACAC

ATCCCCTGTTGTAGGACTTGAGGTGGTTCATTTGTCTCCTTGTCAGACGAGTGGTGTTGT

AATCCTTTGTTATCTAGCCGTGTGATTTTTGAGAATAATAATCAAAGTTTGCAAGCACAT

TATTTAGGTTCCTTTGCATTTATCAAACAACGTTGATATCTAATCTAGGGTTGTGGTTGA

GTGAAAAGAACTCATTCATCTAATTGATATTTGACTTTGAATATGAGACAATCTTAAATC

TTTAGGTTAGTTGTTCTACTTATTAGAGGTGTTTATGATTTAACGGGGATTTATTACTGT

GTTCGATAGTGAAAATTTGTCTAGTACTTTGGAGCAATTCGTAAGTAAAATGTTAATTAT

AACTTTTTTAAAAAATATTTCAATTATATACAATTTTTACTTTTTTTTGTTGTTGAAATA

TTCATGATAAAGACAATATTTATTATATATTTTCTCAAACATTTTCAGCTCAAAAAATCT

AAACTTTTCCCATAAGTTCAAATCTAGGACCATCAATCTGAAGTGATAATCGGAATGTCA

CCTTACTACATGCTAATTCACATTTTTTACTTGTTTAATTATTCAGTTGCATAAATTTTA

AGTTCAACAATTAATTTGACACTTTTTTTAAACTAATGAATGAATCGTTGATTTGAGAAT

AATTTTATATTAAAAAAAGACTTGATTTATTATTATTAATTGTGCATTCCAAACTAATCC

TAAGATGGCAATTAATAATTGAAGTATCACAGAATCGGATAAACACATAGTCGAGTAGAC

GACATATACTTTTTTAACTTGTATTAAATTTCAGCGCGACAGTGCGACACAAAAAGAAGA

AGTTAAAGCGGGGTGGGAGCAGTACAACTGTACAGGCGCTGATGCTTCTTCACTCTCACG

CGGTAGTCTGTTATAACGACAGTTAACGCTTCAAATCATTTCCCTTCCAACTCTCATGAG

CTTCTGAGCTAACACACACACACACAAACCTCCACAGAAATCTCACCCGAATTCTTCTCA

ATTTCTAGGGTTTCACATCGCAATCACTCTGCTGGCTGTATCCGTGCTGTCTTCTGCGTG

CTTTTGCTGCGTGTAATGTGATGGTTACGGACGTGAGTTAGTTAGGGGGGAGCGGAGGAC

GGAGGCGGTGAGGTTTGGAG

>g17621.t1/IbHAK13

GGAATCGACCCATGTAAAAAAGAATCATATATCAAAGGTAGACTGATTTCTTATGCACTA

TATCCAGTGTTTCATGAGTTAGTGATTGATTTCTGTTGTGCACAAGTTTGTAAACTATAG

AAAGGTCAGATGGGCAATGTGATGCTTCTTTCCAAGTTAATTGAGGACTTTGGCTTTTAA

ACATGTTAGATTAGTGTGTGAGACTGATGTTTTAAAACCTAAATGCAAAGCTGGTTATCA

AGACATGCTATTCATCATGATGTATGATTTTGTAATGACTAAATGCATGGAGTTAGAATA

CCTTTAACTTTTTACTTAAGGTTAGCATGTTATTGGGGGTGACTTGCATTTGTGCATGCT

GATGACTGCCTGTCAAAAAATTCTTATAATGTATAACTTAATAGATTCGGTTATGTGACT

CCACACTGTCATCACCCTATAAACACATCTCACACTCTATTGTCACCCTATAAAGATACC

TGACATTTTGTCATCATTTCTATGCCTGCCTTGATTACTATTTGGTGTTACTAGGACTTG

CTGATATCAGTCGTAACCATACTTGAAAAGGTTGGTACAAACTTGGTTCTAATAGTAAGT

TTAGTAAGGTTTATATGCATGCATGATGCATGAACTCTTGACTTAATCAACTAAATCTTA

TTCCCAATCAAACTTTGGTGGGCTTTGCAGTTATGACCATGTCAAATTCTCTGCATCTTT

TCATTCCCCAAGAGTTTTTGCTCAATAATCACAATTAATTTTTCCTCTTCTTCCCCCCTA

GGTGTACTGGATAATATTTTTTGGTGTGGGCATGTATTGACTGTTGTATGGGGGTAGGAG

TCACTTAGAAGTTAGAACCCCGAGCATAAGTTATTCATGCTATATAAAAAAAAGTGCAGA

TTTATTAGGAGGCAGGAGGCTTCACTAGCATCTAGAACCAATCTCTTTACTTTTTCTTTT

GAGTCTATATCCTTAGTAATATCTTCCACTGGGCTTTTGTGTGAGATTTTGGACACAAAT

ACTCCAGAGGCTTCTTTAGGATCTTGAGCTGGTCTTTGATCTTTTCTTTTCCAAACTACT

ATTCTTAATGCCTTCTTGTGTGATCAGGTTCCTGGGTTCCTTATTTTGTTTTCCAGTTAT

TATCTTGGTCTATTCTGTGCCCTCTGCTTTTTTTTTTTTTTGACCTTGTGAAAGATAGTT

GAACTTTCAAATGGGAGAGGATATCATTGATCTAAGGACACTGGGATTCGAAATATGAAA

ATTGCACAAACTATCCAACTGTGTAGTATTAAGTAAAGCCTTTTTATTATTTATGGGAAC

TTACACCTTAAGCTTCACTAGTCAAATATATATCTATTTTTATTTACAGCTGTACAGGAA

AATGTCTGTTGTCCATGGTTGGTTTTATATGAAAGAATTGAATACATCCATGATCCATTT

ACTACTTTTTGAAATGCTTGGGATTCTGTGTGCCCCTTTCTGTCAGTTCTGTGCAACTCA

ATAGATGTTGTTTCCTAAATGAAAAGTAAAGAAAGAAACTTATGTCATCAGGGATTTTAG

AACAGCTAAAAGCAACCTGATGGAAAACAAAACAAATCACTGATTGATAGTCATAATGTG

TAATATGGAGTACTTGATATTTCCATTTGAAATTTTCTTGCAAGACAATCGTTAGGATGG

TAATGTAGTTACACACATGAATGTCCATGCGTATGTGTTATGTGTGTGTGTGTGTGTGTG

TGTGTTTATTCATTTATTTTTGGGGAGGGAACAGGCAAGCTACGCATAACTAGAGACTAA

AGCTCATATGTGTTATTGTCCAGTTTTCTTTAGCCCGTGATCTACTGTTGGCATACCAGA

GTTTTGGTGTGGTATATGGAGATTTGAGCATTTCACCACTTTATGTCTATAAAAGTATTT

TTGAGGGAAAGTTGCAGAGTCATCAACATCCAGACGCAATACTTGGGGCATTTTCATTGA

TCTTTTGGACACTAACATTG
