# Supplementary material for: Identification of the HAK/KUP/KT Potassium Transporter Gene Family in Sweet Potato and Functional Characterization of IbHAK5A
Source: Plants (Basel). 2026 Aug 12;15(16):2451. doi: 10.3390/plants15162451 (PMC13516474; doi:10.3390/plants15162451)
Supplement: Supplementary file 1 [file plants-15-02451-s001.zip › IbHAK5A-promoter analysis-PlantCARE/PlantCARE_20562_plantcare.html]

Content-Type: text/html; charset=ISO-8859-1


PlantCARE


Webmaster Firefox specific output  
To save the result:
click on the frame with the right mouse button and save the source code as a text file with extension .html  
REFERENCE:PlantCARE: a database of plant cis-acting regulatory elements and a portal to tools for in silico analysis of promoter sequences.  
Lescot, M., Déhais, P., Moreau, Y., De Moor, B., Rouzé ,P.,and Rombauts, S.  
Nucleic Acids Res., Database issue(2002), 30(1):325-327.   


---

>PlantCARE\_20562   
+ TATTTTAGTT AGTTTTATGG AAGAATATTA AAATCATTTT TCATTTACAT GTCAATCTAT ATAATATGGT   
  
  
+ TAAATAATTG TATAATTTAT GCTATCTCAC ACGAAAATAC TTAACCTGTT TTATGAGATG ATATAGTAAA   
  
  
+ ATTTGAAATT CAATTTGTCA AATTTAGTTT TCATTGAGTT TTTTCGCTGG AAAAATATAT ACCTAATTTG   
  
  
+ AAGTCCCTAT AAATTAATCT GATAAGATTA TTTTACCAAA ACTGCTTAAA TTTTCATATT AAGTTGTTTC   
  
  
+ TGACTTTCTG ACGTTTCTGT GGTAAGAAAT CTTGTCTAAT GCCTTGTTTT ACAATCACAA TAGCCTATGG   
  
  
+ TTATCAACTT TTATAATAGT TTGAAGTAAA ACTTTTTTTT TGTTTGTTTT TAGAACTTTG ATGACCAGAT   
  
  
+ GGTTTGACTT TTTGTTGCAT ATTGATTAAA TTTCACATGT AAATGCTGAC TAACATTTAA AATTAAGGTT   
  
  
+ TGGCATATGA TGATGCCCAT TTCTGATGGC AGTTTGGTAT ATAATTTTTT TCAATTCAGT AAAAGCAAAT   
  
  
+ ACCCCATTTT ATGCTATTTC ACATGACCTA ACCAATTTTC TGAAATGATA TACTAAAATC TGACATTTAA   
  
  
+ TTTATTTGAA CAAAGTTTGT TATTATTTCC ATTCTTATTA TAATATAAAG TATTACACTT ATTACTATTA   
  
  
+ TTACTACTAC AATAATTATT ATTACTATAG TTAGTATTAT TACTATTATT AATACTACTA CCACTATTGT   
  
  
+ TAATTTGTTA TTATTACTAT TATTATTATT ATTATTATTA TTATTATTAT TATTTTTACT ACTCTTATTA   
  
  
+ CTATTATTAT ATTACTACTA CAATAATTAT TATTACTATA GTTAGTATTA TTCTTTTTAC TATTATTATT   
  
  
+ AATTATTAAT ATTATTAGTT TTAGTATTTT TTTAAAATTA TTATTAGAGT AACACTAGTA CTATAAAATT   
  
  
+ GACATTCTTT TCTTCCCCCC TCTCAATCTC TCATTCTCTC TTTCTCTCAG CCCTCTCCGC TGCTCTCACC   
  
  
+ CCTCACACTC TCCTTCGAGG CTTATTCAGG AAATCCCATA TCTCCTTTGA GGCTTATTCT TGGATTTGTT   
  
  
+ GTTGAACAGT TCTCTATATA TGTGTGTGTG TGTATATATA TATATATATA TATATATATA TATTTTTTTT   
  
  
+ TTTTTTTTTT TTTTTTTTTT GTGTTTAGAA ATTAGATATT CAGATATTGA AAAGTTTACG TCAAGATTTT   
  
  
+ TCTTTTCCAG AAAATTAAAT ATTTTACCTG GAATCTTTTG AGTTCCCATG TCTAGTTAAC AATTGTGTGT   
  
  
+ ATTTTATTTC CTGAACAATT TTCAGTTGTT AACATGAATT TTGAATGTTT CTTTTTTATT GTCTCTAGGG   
  
  
+ GCATATGCAT ATTAGCAGAG TTGTTAACTT GTTAACTAGA AGCACATGTT TGTCATATCT TCTTTGTGTT   
  
  
+ AGCTTTTTTA ATGGTTAATT GTCATTCTGC TGTGCTATAT TCTATATGTG CTTTGAATGC GTGTAGCTAA   
  
  
+ AATAATCCTA AAACTGAATT ATTTGAACTT CTAGACAAAT ACAACTCGAT GCCCAGGTAA CCTCCAAAGT   
  
  
+ TTTTTAAATA ACTGAACTAT CTTCAATATA ATACCATCAT GACAGTAAGG TTAGCTTGCT GAGTTTATTA   
  
  
+ AATATGTTAA CAGTTTCCCC AGTTGTCTTG TTTGTTTCTC ACTTGTGCCA TTAGGAGGCT GGGGTTTGGA   
  
  
+ ACGTTAGAGA AGAAAGAAAA AGGGAAAAAG AGGGAGAGAA AAAGAATAGA TATATTATAG TGATGACTGA   
  
  
+ TGAGTACTTG AGCACTTGAC TACCTGATTG TTTTTATTTT TTTCAAAATG TAAACTTCTA TTTATTTATT   
  
  
+ TAGGTAAAAC TTGATAATGG CATATTATTG AACCATGCTC TGGCACTTTG CACTTTGCAG ATCCTGTGCA   
  
  
+ TTTCAGTAGT CTGTATAATT CCTACTTGTT TTAAAAAA  

- ATAAAATCAA TCAAAATACC TTCTTATAAT TTTAGTAAAA AGTAAATGTA CAGTTAGATA TATTATACCA   
  
  
- ATTTATTAAC ATATTAAATA CGATAGAGTG TGCTTTTATG AATTGGACAA AATACTCTAC TATATCATTT   
  
  
- TAAACTTTAA GTTAAACAGT TTAAATCAAA AGTAACTCAA AAAAGCGACC TTTTTATATA TGGATTAAAC   
  
  
- TTCAGGGATA TTTAATTAGA CTATTCTAAT AAAATGGTTT TGACGAATTT AAAAGTATAA TTCAACAAAG   
  
  
- ACTGAAAGAC TGCAAAGACA CCATTCTTTA GAACAGATTA CGGAACAAAA TGTTAGTGTT ATCGGATACC   
  
  
- AATAGTTGAA AATATTATCA AACTTCATTT TGAAAAAAAA ACAAACAAAA ATCTTGAAAC TACTGGTCTA   
  
  
- CCAAACTGAA AAACAACGTA TAACTAATTT AAAGTGTACA TTTACGACTG ATTGTAAATT TTAATTCCAA   
  
  
- ACCGTATACT ACTACGGGTA AAGACTACCG TCAAACCATA TATTAAAAAA AGTTAAGTCA TTTTCGTTTA   
  
  
- TGGGGTAAAA TACGATAAAG TGTACTGGAT TGGTTAAAAG ACTTTACTAT ATGATTTTAG ACTGTAAATT   
  
  
- AAATAAACTT GTTTCAAACA ATAATAAAGG TAAGAATAAT ATTATATTTC ATAATGTGAA TAATGATAAT   
  
  
- AATGATGATG TTATTAATAA TAATGATATC AATCATAATA ATGATAATAA TTATGATGAT GGTGATAACA   
  
  
- ATTAAACAAT AATAATGATA ATAATAATAA TAATAATAAT AATAATAATA ATAAAAATGA TGAGAATAAT   
  
  
- GATAATAATA TAATGATGAT GTTATTAATA ATAATGATAT CAATCATAAT AAGAAAAATG ATAATAATAA   
  
  
- TTAATAATTA TAATAATCAA AATCATAAAA AAATTTTAAT AATAATCTCA TTGTGATCAT GATATTTTAA   
  
  
- CTGTAAGAAA AGAAGGGGGG AGAGTTAGAG AGTAAGAGAG AAAGAGAGTC GGGAGAGGCG ACGAGAGTGG   
  
  
- GGAGTGTGAG AGGAAGCTCC GAATAAGTCC TTTAGGGTAT AGAGGAAACT CCGAATAAGA ACCTAAACAA   
  
  
- CAACTTGTCA AGAGATATAT ACACACACAC ACATATATAT ATATATATAT ATATATATAT ATAAAAAAAA   
  
  
- AAAAAAAAAA AAAAAAAAAA CACAAATCTT TAATCTATAA GTCTATAACT TTTCAAATGC AGTTCTAAAA   
  
  
- AGAAAAGGTC TTTTAATTTA TAAAATGGAC CTTAGAAAAC TCAAGGGTAC AGATCAATTG TTAACACACA   
  
  
- TAAAATAAAG GACTTGTTAA AAGTCAACAA TTGTACTTAA AACTTACAAA GAAAAAATAA CAGAGATCCC   
  
  
- CGTATACGTA TAATCGTCTC AACAATTGAA CAATTGATCT TCGTGTACAA ACAGTATAGA AGAAACACAA   
  
  
- TCGAAAAAAT TACCAATTAA CAGTAAGACG ACACGATATA AGATATACAC GAAACTTACG CACATCGATT   
  
  
- TTATTAGGAT TTTGACTTAA TAAACTTGAA GATCTGTTTA TGTTGAGCTA CGGGTCCATT GGAGGTTTCA   
  
  
- AAAAATTTAT TGACTTGATA GAAGTTATAT TATGGTAGTA CTGTCATTCC AATCGAACGA CTCAAATAAT   
  
  
- TTATACAATT GTCAAAGGGG TCAACAGAAC AAACAAAGAG TGAACACGGT AATCCTCCGA CCCCAAACCT   
  
  
- TGCAATCTCT TCTTTCTTTT TCCCTTTTTC TCCCTCTCTT TTTCTTATCT ATATAATATC ACTACTGACT   
  
  
- ACTCATGAAC TCGTGAACTG ATGGACTAAC AAAAATAAAA AAAGTTTTAC ATTTGAAGAT AAATAAATAA   
  
  
- ATCCATTTTG AACTATTACC GTATAATAAC TTGGTACGAG ACCGTGAAAC GTGAAACGTC TAGGACACGT   
  
  
- AAAGTCATCA GACATATTAA GGATGAACAA AATTTTTT

  
  
Motifs Found  

+   

| Site Name | Organism | Position | Strand | Matrix score. | sequence | function |
| --- | --- | --- | --- | --- | --- | --- |
|  | organism | 1058 | + | 4 | motif\_sequence | short\_function |
|  | organism | 1516 | + | 4 | motif\_sequence | short\_function |
|  | organism | 1415 | - | 4 | motif\_sequence | short\_function |
|  | organism | 1954 | + | 4 | motif\_sequence | short\_function |
|  | organism | 1090 | + | 4 | motif\_sequence | short\_function |
|  | organism | 1783 | - | 4 | motif\_sequence | short\_function |
|  | organism | 1457 | + | 4 | motif\_sequence | short\_function |
|  | organism | 1629 | + | 4 | motif\_sequence | short\_function |
|  | organism | 1033 | + | 4 | motif\_sequence | short\_function |
|  | organism | 1947 | - | 4 | motif\_sequence | short\_function |
|  | organism | 1442 | - | 4 | motif\_sequence | short\_function |
|  | organism | 1124 | - | 4 | motif\_sequence | short\_function |
|  | organism | 1500 | + | 4 | motif\_sequence | short\_function |

>PlantCARE\_20562   
+ TATTTTAGTT AGTTTTATGG AAGAATATTA AAATCATTTT TCATTTACAT GTCAATCTAT ATAATATGGT   
  
  
+ TAAATAATTG TATAATTTAT GCTATCTCAC ACGAAAATAC TTAACCTGTT TTATGAGATG ATATAGTAAA   
  
  
+ ATTTGAAATT CAATTTGTCA AATTTAGTTT TCATTGAGTT TTTTCGCTGG AAAAATATAT ACCTAATTTG   
  
  
+ AAGTCCCTAT AAATTAATCT GATAAGATTA TTTTACCAAA ACTGCTTAAA TTTTCATATT AAGTTGTTTC   
  
  
+ TGACTTTCTG ACGTTTCTGT GGTAAGAAAT CTTGTCTAAT GCCTTGTTTT ACAATCACAA TAGCCTATGG   
  
  
+ TTATCAACTT TTATAATAGT TTGAAGTAAA ACTTTTTTTT TGTTTGTTTT TAGAACTTTG ATGACCAGAT   
  
  
+ GGTTTGACTT TTTGTTGCAT ATTGATTAAA TTTCACATGT AAATGCTGAC TAACATTTAA AATTAAGGTT   
  
  
+ TGGCATATGA TGATGCCCAT TTCTGATGGC AGTTTGGTAT ATAATTTTTT TCAATTCAGT AAAAGCAAAT   
  
  
+ ACCCCATTTT ATGCTATTTC ACATGACCTA ACCAATTTTC TGAAATGATA TACTAAAATC TGACATTTAA   
  
  
+ TTTATTTGAA CAAAGTTTGT TATTATTTCC ATTCTTATTA TAATATAAAG TATTACACTT ATTACTATTA   
  
  
+ TTACTACTAC AATAATTATT ATTACTATAG TTAGTATTAT TACTATTATT AATACTACTA CCACTATTGT   
  
  
+ TAATTTGTTA TTATTACTAT TATTATTATT ATTATTATTA TTATTATTAT TATTTTTACT ACTCTTATTA   
  
  
+ CTATTATTAT ATTACTACTA CAATAATTAT TATTACTATA GTTAGTATTA TTCTTTTTAC TATTATTATT   
  
  
+ AATTATTAAT ATTATTAGTT TTAGTATTTT TTTAAAATTA TTATTAGAGT AACACTAGTA CTATAAAATT   
  
  
+ GACATTCTTT TCTTCCCCCC TCTCAATCTC TCATTCTCTC TTTCTCTCAG CCCTCTCCGC TGCTCTCACC   
  
  
+ CCTCACACTC TCCTTCGAGG CTTATTCAGG AAATCCCATA TCTCCTTTGA GGCTTATTCT TGGATTTGTT   
  
  
+ GTTGAACAGT TCTCTATATA TGTGTGTGTG TGTATATATA TATATATATA TATATATATA TATTTTTTTT   
  
  
+ TTTTTTTTTT TTTTTTTTTT GTGTTTAGAA ATTAGATATT CAGATATTGA AAAGTTTACG TCAAGATTTT   
  
  
+ TCTTTTCCAG AAAATTAAAT ATTTTACCTG GAATCTTTTG AGTTCCCATG TCTAGTTAAC AATTGTGTGT   
  
  
+ ATTTTATTTC CTGAACAATT TTCAGTTGTT AACATGAATT TTGAATGTTT CTTTTTTATT GTCTCTAGGG   
  
  
+ GCATATGCAT ATTAGCAGAG TTGTTAACTT GTTAACTAGA AGCACATGTT TGTCATATCT TCTTTGTGTT   
  
  
+ AGCTTTTTTA ATGGTTAATT GTCATTCTGC TGTGCTATAT TCTATATGTG CTTTGAATGC GTGTAGCTAA   
  
  
+ AATAATCCTA AAACTGAATT ATTTGAACTT CTAGACAAAT ACAACTCGAT GCCCAGGTAA CCTCCAAAGT   
  
  
+ TTTTTAAATA ACTGAACTAT CTTCAATATA ATACCATCAT GACAGTAAGG TTAGCTTGCT GAGTTTATTA   
  
  
+ AATATGTTAA CAGTTTCCCC AGTTGTCTTG TTTGTTTCTC ACTTGTGCCA TTAGGAGGCT GGGGTTTGGA   
  
  
+ ACGTTAGAGA AGAAAGAAAA AGGGAAAAAG AGGGAGAGAA AAAGAATAGA TATATTATAG TGATGACTGA   
  
  
+ TGAGTACTTG AGCACTTGAC TACCTGATTG TTTTTATTTT TTTCAAAATG TAAACTTCTA TTTATTTATT   
  
  
+ TAGGTAAAAC TTGATAATGG CATATTATTG AACCATGCTC TGGCACTTTG CACTTTGCAG ATCCTGTGCA   
  
  
+ TTTCAGTAGT CTGTATAATT CCTACTTGTT TTAAAAAA  

- ATAAAATCAA TCAAAATACC TTCTTATAAT TTTAGTAAAA AGTAAATGTA CAGTTAGATA TATTATACCA   
  
  
- ATTTATTAAC ATATTAAATA CGATAGAGTG TGCTTTTATG AATTGGACAA AATACTCTAC TATATCATTT   
  
  
- TAAACTTTAA GTTAAACAGT TTAAATCAAA AGTAACTCAA AAAAGCGACC TTTTTATATA TGGATTAAAC   
  
  
- TTCAGGGATA TTTAATTAGA CTATTCTAAT AAAATGGTTT TGACGAATTT AAAAGTATAA TTCAACAAAG   
  
  
- ACTGAAAGAC TGCAAAGACA CCATTCTTTA GAACAGATTA CGGAACAAAA TGTTAGTGTT ATCGGATACC   
  
  
- AATAGTTGAA AATATTATCA AACTTCATTT TGAAAAAAAA ACAAACAAAA ATCTTGAAAC TACTGGTCTA   
  
  
- CCAAACTGAA AAACAACGTA TAACTAATTT AAAGTGTACA TTTACGACTG ATTGTAAATT TTAATTCCAA   
  
  
- ACCGTATACT ACTACGGGTA AAGACTACCG TCAAACCATA TATTAAAAAA AGTTAAGTCA TTTTCGTTTA   
  
  
- TGGGGTAAAA TACGATAAAG TGTACTGGAT TGGTTAAAAG ACTTTACTAT ATGATTTTAG ACTGTAAATT   
  
  
- AAATAAACTT GTTTCAAACA ATAATAAAGG TAAGAATAAT ATTATATTTC ATAATGTGAA TAATGATAAT   
  
  
- AATGATGATG TTATTAATAA TAATGATATC AATCATAATA ATGATAATAA TTATGATGAT GGTGATAACA   
  
  
- ATTAAACAAT AATAATGATA ATAATAATAA TAATAATAAT AATAATAATA ATAAAAATGA TGAGAATAAT   
  
  
- GATAATAATA TAATGATGAT GTTATTAATA ATAATGATAT CAATCATAAT AAGAAAAATG ATAATAATAA   
  
  
- TTAATAATTA TAATAATCAA AATCATAAAA AAATTTTAAT AATAATCTCA TTGTGATCAT GATATTTTAA   
  
  
- CTGTAAGAAA AGAAGGGGGG AGAGTTAGAG AGTAAGAGAG AAAGAGAGTC GGGAGAGGCG ACGAGAGTGG   
  
  
- GGAGTGTGAG AGGAAGCTCC GAATAAGTCC TTTAGGGTAT AGAGGAAACT CCGAATAAGA ACCTAAACAA   
  
  
- CAACTTGTCA AGAGATATAT ACACACACAC ACATATATAT ATATATATAT ATATATATAT ATAAAAAAAA   
  
  
- AAAAAAAAAA AAAAAAAAAA CACAAATCTT TAATCTATAA GTCTATAACT TTTCAAATGC AGTTCTAAAA   
  
  
- AGAAAAGGTC TTTTAATTTA TAAAATGGAC CTTAGAAAAC TCAAGGGTAC AGATCAATTG TTAACACACA   
  
  
- TAAAATAAAG GACTTGTTAA AAGTCAACAA TTGTACTTAA AACTTACAAA GAAAAAATAA CAGAGATCCC   
  
  
- CGTATACGTA TAATCGTCTC AACAATTGAA CAATTGATCT TCGTGTACAA ACAGTATAGA AGAAACACAA   
  
  
- TCGAAAAAAT TACCAATTAA CAGTAAGACG ACACGATATA AGATATACAC GAAACTTACG CACATCGATT   
  
  
- TTATTAGGAT TTTGACTTAA TAAACTTGAA GATCTGTTTA TGTTGAGCTA CGGGTCCATT GGAGGTTTCA   
  
  
- AAAAATTTAT TGACTTGATA GAAGTTATAT TATGGTAGTA CTGTCATTCC AATCGAACGA CTCAAATAAT   
  
  
- TTATACAATT GTCAAAGGGG TCAACAGAAC AAACAAAGAG TGAACACGGT AATCCTCCGA CCCCAAACCT   
  
  
- TGCAATCTCT TCTTTCTTTT TCCCTTTTTC TCCCTCTCTT TTTCTTATCT ATATAATATC ACTACTGACT   
  
  
- ACTCATGAAC TCGTGAACTG ATGGACTAAC AAAAATAAAA AAAGTTTTAC ATTTGAAGAT AAATAAATAA   
  
  
- ATCCATTTTG AACTATTACC GTATAATAAC TTGGTACGAG ACCGTGAAAC GTGAAACGTC TAGGACACGT   
  
  
- AAAGTCATCA GACATATTAA GGATGAACAA AATTTTTT

+     AAGAA-motif

| Site Name | Organism | Position | Strand | Matrix score. | sequence | function |
| --- | --- | --- | --- | --- | --- | --- |
| AAGAA-motif | Avena sativa | 1259 | - | 9 | gGTAAAGAAA |  |
| AAGAA-motif | Avena sativa | 1762 | + | 7 | GAAAGAA |  |

>PlantCARE\_20562   
+ TATTTTAGTT AGTTTTATGG AAGAATATTA AAATCATTTT TCATTTACAT GTCAATCTAT ATAATATGGT   
  
  
+ TAAATAATTG TATAATTTAT GCTATCTCAC ACGAAAATAC TTAACCTGTT TTATGAGATG ATATAGTAAA   
  
  
+ ATTTGAAATT CAATTTGTCA AATTTAGTTT TCATTGAGTT TTTTCGCTGG AAAAATATAT ACCTAATTTG   
  
  
+ AAGTCCCTAT AAATTAATCT GATAAGATTA TTTTACCAAA ACTGCTTAAA TTTTCATATT AAGTTGTTTC   
  
  
+ TGACTTTCTG ACGTTTCTGT GGTAAGAAAT CTTGTCTAAT GCCTTGTTTT ACAATCACAA TAGCCTATGG   
  
  
+ TTATCAACTT TTATAATAGT TTGAAGTAAA ACTTTTTTTT TGTTTGTTTT TAGAACTTTG ATGACCAGAT   
  
  
+ GGTTTGACTT TTTGTTGCAT ATTGATTAAA TTTCACATGT AAATGCTGAC TAACATTTAA AATTAAGGTT   
  
  
+ TGGCATATGA TGATGCCCAT TTCTGATGGC AGTTTGGTAT ATAATTTTTT TCAATTCAGT AAAAGCAAAT   
  
  
+ ACCCCATTTT ATGCTATTTC ACATGACCTA ACCAATTTTC TGAAATGATA TACTAAAATC TGACATTTAA   
  
  
+ TTTATTTGAA CAAAGTTTGT TATTATTTCC ATTCTTATTA TAATATAAAG TATTACACTT ATTACTATTA   
  
  
+ TTACTACTAC AATAATTATT ATTACTATAG TTAGTATTAT TACTATTATT AATACTACTA CCACTATTGT   
  
  
+ TAATTTGTTA TTATTACTAT TATTATTATT ATTATTATTA TTATTATTAT TATTTTTACT ACTCTTATTA   
  
  
+ CTATTATTAT ATTACTACTA CAATAATTAT TATTACTATA GTTAGTATTA TTCTTTTTAC TATTATTATT   
  
  
+ AATTATTAAT ATTATTAGTT TTAGTATTTT TTTAAAATTA TTATTAGAGT AACACTAGTA CTATAAAATT   
  
  
+ GACATTCTTT TCTTCCCCCC TCTCAATCTC TCATTCTCTC TTTCTCTCAG CCCTCTCCGC TGCTCTCACC   
  
  
+ CCTCACACTC TCCTTCGAGG CTTATTCAGG AAATCCCATA TCTCCTTTGA GGCTTATTCT TGGATTTGTT   
  
  
+ GTTGAACAGT TCTCTATATA TGTGTGTGTG TGTATATATA TATATATATA TATATATATA TATTTTTTTT   
  
  
+ TTTTTTTTTT TTTTTTTTTT GTGTTTAGAA ATTAGATATT CAGATATTGA AAAGTTTACG TCAAGATTTT   
  
  
+ TCTTTTCCAG AAAATTAAAT ATTTTACCTG GAATCTTTTG AGTTCCCATG TCTAGTTAAC AATTGTGTGT   
  
  
+ ATTTTATTTC CTGAACAATT TTCAGTTGTT AACATGAATT TTGAATGTTT CTTTTTTATT GTCTCTAGGG   
  
  
+ GCATATGCAT ATTAGCAGAG TTGTTAACTT GTTAACTAGA AGCACATGTT TGTCATATCT TCTTTGTGTT   
  
  
+ AGCTTTTTTA ATGGTTAATT GTCATTCTGC TGTGCTATAT TCTATATGTG CTTTGAATGC GTGTAGCTAA   
  
  
+ AATAATCCTA AAACTGAATT ATTTGAACTT CTAGACAAAT ACAACTCGAT GCCCAGGTAA CCTCCAAAGT   
  
  
+ TTTTTAAATA ACTGAACTAT CTTCAATATA ATACCATCAT GACAGTAAGG TTAGCTTGCT GAGTTTATTA   
  
  
+ AATATGTTAA CAGTTTCCCC AGTTGTCTTG TTTGTTTCTC ACTTGTGCCA TTAGGAGGCT GGGGTTTGGA   
  
  
+ ACGTTAGAGA AGAAAGAAAA AGGGAAAAAG AGGGAGAGAA AAAGAATAGA TATATTATAG TGATGACTGA   
  
  
+ TGAGTACTTG AGCACTTGAC TACCTGATTG TTTTTATTTT TTTCAAAATG TAAACTTCTA TTTATTTATT   
  
  
+ TAGGTAAAAC TTGATAATGG CATATTATTG AACCATGCTC TGGCACTTTG CACTTTGCAG ATCCTGTGCA   
  
  
+ TTTCAGTAGT CTGTATAATT CCTACTTGTT TTAAAAAA  

- ATAAAATCAA TCAAAATACC TTCTTATAAT TTTAGTAAAA AGTAAATGTA CAGTTAGATA TATTATACCA   
  
  
- ATTTATTAAC ATATTAAATA CGATAGAGTG TGCTTTTATG AATTGGACAA AATACTCTAC TATATCATTT   
  
  
- TAAACTTTAA GTTAAACAGT TTAAATCAAA AGTAACTCAA AAAAGCGACC TTTTTATATA TGGATTAAAC   
  
  
- TTCAGGGATA TTTAATTAGA CTATTCTAAT AAAATGGTTT TGACGAATTT AAAAGTATAA TTCAACAAAG   
  
  
- ACTGAAAGAC TGCAAAGACA CCATTCTTTA GAACAGATTA CGGAACAAAA TGTTAGTGTT ATCGGATACC   
  
  
- AATAGTTGAA AATATTATCA AACTTCATTT TGAAAAAAAA ACAAACAAAA ATCTTGAAAC TACTGGTCTA   
  
  
- CCAAACTGAA AAACAACGTA TAACTAATTT AAAGTGTACA TTTACGACTG ATTGTAAATT TTAATTCCAA   
  
  
- ACCGTATACT ACTACGGGTA AAGACTACCG TCAAACCATA TATTAAAAAA AGTTAAGTCA TTTTCGTTTA   
  
  
- TGGGGTAAAA TACGATAAAG TGTACTGGAT TGGTTAAAAG ACTTTACTAT ATGATTTTAG ACTGTAAATT   
  
  
- AAATAAACTT GTTTCAAACA ATAATAAAGG TAAGAATAAT ATTATATTTC ATAATGTGAA TAATGATAAT   
  
  
- AATGATGATG TTATTAATAA TAATGATATC AATCATAATA ATGATAATAA TTATGATGAT GGTGATAACA   
  
  
- ATTAAACAAT AATAATGATA ATAATAATAA TAATAATAAT AATAATAATA ATAAAAATGA TGAGAATAAT   
  
  
- GATAATAATA TAATGATGAT GTTATTAATA ATAATGATAT CAATCATAAT AAGAAAAATG ATAATAATAA   
  
  
- TTAATAATTA TAATAATCAA AATCATAAAA AAATTTTAAT AATAATCTCA TTGTGATCAT GATATTTTAA   
  
  
- CTGTAAGAAA AGAAGGGGGG AGAGTTAGAG AGTAAGAGAG AAAGAGAGTC GGGAGAGGCG ACGAGAGTGG   
  
  
- GGAGTGTGAG AGGAAGCTCC GAATAAGTCC TTTAGGGTAT AGAGGAAACT CCGAATAAGA ACCTAAACAA   
  
  
- CAACTTGTCA AGAGATATAT ACACACACAC ACATATATAT ATATATATAT ATATATATAT ATAAAAAAAA   
  
  
- AAAAAAAAAA AAAAAAAAAA CACAAATCTT TAATCTATAA GTCTATAACT TTTCAAATGC AGTTCTAAAA   
  
  
- AGAAAAGGTC TTTTAATTTA TAAAATGGAC CTTAGAAAAC TCAAGGGTAC AGATCAATTG TTAACACACA   
  
  
- TAAAATAAAG GACTTGTTAA AAGTCAACAA TTGTACTTAA AACTTACAAA GAAAAAATAA CAGAGATCCC   
  
  
- CGTATACGTA TAATCGTCTC AACAATTGAA CAATTGATCT TCGTGTACAA ACAGTATAGA AGAAACACAA   
  
  
- TCGAAAAAAT TACCAATTAA CAGTAAGACG ACACGATATA AGATATACAC GAAACTTACG CACATCGATT   
  
  
- TTATTAGGAT TTTGACTTAA TAAACTTGAA GATCTGTTTA TGTTGAGCTA CGGGTCCATT GGAGGTTTCA   
  
  
- AAAAATTTAT TGACTTGATA GAAGTTATAT TATGGTAGTA CTGTCATTCC AATCGAACGA CTCAAATAAT   
  
  
- TTATACAATT GTCAAAGGGG TCAACAGAAC AAACAAAGAG TGAACACGGT AATCCTCCGA CCCCAAACCT   
  
  
- TGCAATCTCT TCTTTCTTTT TCCCTTTTTC TCCCTCTCTT TTTCTTATCT ATATAATATC ACTACTGACT   
  
  
- ACTCATGAAC TCGTGAACTG ATGGACTAAC AAAAATAAAA AAAGTTTTAC ATTTGAAGAT AAATAAATAA   
  
  
- ATCCATTTTG AACTATTACC GTATAATAAC TTGGTACGAG ACCGTGAAAC GTGAAACGTC TAGGACACGT   
  
  
- AAAGTCATCA GACATATTAA GGATGAACAA AATTTTTT

+     AE-box

| Site Name | Organism | Position | Strand | Matrix score. | sequence | function |
| --- | --- | --- | --- | --- | --- | --- |
| AE-box | Arabidopsis thaliana | 274 | - | 8 | AGAAACAA | part of a module for light response |
| AE-box | Arabidopsis thaliana | 1712 | - | 8 | AGAAACAA | part of a module for light response |

>PlantCARE\_20562   
+ TATTTTAGTT AGTTTTATGG AAGAATATTA AAATCATTTT TCATTTACAT GTCAATCTAT ATAATATGGT   
  
  
+ TAAATAATTG TATAATTTAT GCTATCTCAC ACGAAAATAC TTAACCTGTT TTATGAGATG ATATAGTAAA   
  
  
+ ATTTGAAATT CAATTTGTCA AATTTAGTTT TCATTGAGTT TTTTCGCTGG AAAAATATAT ACCTAATTTG   
  
  
+ AAGTCCCTAT AAATTAATCT GATAAGATTA TTTTACCAAA ACTGCTTAAA TTTTCATATT AAGTTGTTTC   
  
  
+ TGACTTTCTG ACGTTTCTGT GGTAAGAAAT CTTGTCTAAT GCCTTGTTTT ACAATCACAA TAGCCTATGG   
  
  
+ TTATCAACTT TTATAATAGT TTGAAGTAAA ACTTTTTTTT TGTTTGTTTT TAGAACTTTG ATGACCAGAT   
  
  
+ GGTTTGACTT TTTGTTGCAT ATTGATTAAA TTTCACATGT AAATGCTGAC TAACATTTAA AATTAAGGTT   
  
  
+ TGGCATATGA TGATGCCCAT TTCTGATGGC AGTTTGGTAT ATAATTTTTT TCAATTCAGT AAAAGCAAAT   
  
  
+ ACCCCATTTT ATGCTATTTC ACATGACCTA ACCAATTTTC TGAAATGATA TACTAAAATC TGACATTTAA   
  
  
+ TTTATTTGAA CAAAGTTTGT TATTATTTCC ATTCTTATTA TAATATAAAG TATTACACTT ATTACTATTA   
  
  
+ TTACTACTAC AATAATTATT ATTACTATAG TTAGTATTAT TACTATTATT AATACTACTA CCACTATTGT   
  
  
+ TAATTTGTTA TTATTACTAT TATTATTATT ATTATTATTA TTATTATTAT TATTTTTACT ACTCTTATTA   
  
  
+ CTATTATTAT ATTACTACTA CAATAATTAT TATTACTATA GTTAGTATTA TTCTTTTTAC TATTATTATT   
  
  
+ AATTATTAAT ATTATTAGTT TTAGTATTTT TTTAAAATTA TTATTAGAGT AACACTAGTA CTATAAAATT   
  
  
+ GACATTCTTT TCTTCCCCCC TCTCAATCTC TCATTCTCTC TTTCTCTCAG CCCTCTCCGC TGCTCTCACC   
  
  
+ CCTCACACTC TCCTTCGAGG CTTATTCAGG AAATCCCATA TCTCCTTTGA GGCTTATTCT TGGATTTGTT   
  
  
+ GTTGAACAGT TCTCTATATA TGTGTGTGTG TGTATATATA TATATATATA TATATATATA TATTTTTTTT   
  
  
+ TTTTTTTTTT TTTTTTTTTT GTGTTTAGAA ATTAGATATT CAGATATTGA AAAGTTTACG TCAAGATTTT   
  
  
+ TCTTTTCCAG AAAATTAAAT ATTTTACCTG GAATCTTTTG AGTTCCCATG TCTAGTTAAC AATTGTGTGT   
  
  
+ ATTTTATTTC CTGAACAATT TTCAGTTGTT AACATGAATT TTGAATGTTT CTTTTTTATT GTCTCTAGGG   
  
  
+ GCATATGCAT ATTAGCAGAG TTGTTAACTT GTTAACTAGA AGCACATGTT TGTCATATCT TCTTTGTGTT   
  
  
+ AGCTTTTTTA ATGGTTAATT GTCATTCTGC TGTGCTATAT TCTATATGTG CTTTGAATGC GTGTAGCTAA   
  
  
+ AATAATCCTA AAACTGAATT ATTTGAACTT CTAGACAAAT ACAACTCGAT GCCCAGGTAA CCTCCAAAGT   
  
  
+ TTTTTAAATA ACTGAACTAT CTTCAATATA ATACCATCAT GACAGTAAGG TTAGCTTGCT GAGTTTATTA   
  
  
+ AATATGTTAA CAGTTTCCCC AGTTGTCTTG TTTGTTTCTC ACTTGTGCCA TTAGGAGGCT GGGGTTTGGA   
  
  
+ ACGTTAGAGA AGAAAGAAAA AGGGAAAAAG AGGGAGAGAA AAAGAATAGA TATATTATAG TGATGACTGA   
  
  
+ TGAGTACTTG AGCACTTGAC TACCTGATTG TTTTTATTTT TTTCAAAATG TAAACTTCTA TTTATTTATT   
  
  
+ TAGGTAAAAC TTGATAATGG CATATTATTG AACCATGCTC TGGCACTTTG CACTTTGCAG ATCCTGTGCA   
  
  
+ TTTCAGTAGT CTGTATAATT CCTACTTGTT TTAAAAAA  

- ATAAAATCAA TCAAAATACC TTCTTATAAT TTTAGTAAAA AGTAAATGTA CAGTTAGATA TATTATACCA   
  
  
- ATTTATTAAC ATATTAAATA CGATAGAGTG TGCTTTTATG AATTGGACAA AATACTCTAC TATATCATTT   
  
  
- TAAACTTTAA GTTAAACAGT TTAAATCAAA AGTAACTCAA AAAAGCGACC TTTTTATATA TGGATTAAAC   
  
  
- TTCAGGGATA TTTAATTAGA CTATTCTAAT AAAATGGTTT TGACGAATTT AAAAGTATAA TTCAACAAAG   
  
  
- ACTGAAAGAC TGCAAAGACA CCATTCTTTA GAACAGATTA CGGAACAAAA TGTTAGTGTT ATCGGATACC   
  
  
- AATAGTTGAA AATATTATCA AACTTCATTT TGAAAAAAAA ACAAACAAAA ATCTTGAAAC TACTGGTCTA   
  
  
- CCAAACTGAA AAACAACGTA TAACTAATTT AAAGTGTACA TTTACGACTG ATTGTAAATT TTAATTCCAA   
  
  
- ACCGTATACT ACTACGGGTA AAGACTACCG TCAAACCATA TATTAAAAAA AGTTAAGTCA TTTTCGTTTA   
  
  
- TGGGGTAAAA TACGATAAAG TGTACTGGAT TGGTTAAAAG ACTTTACTAT ATGATTTTAG ACTGTAAATT   
  
  
- AAATAAACTT GTTTCAAACA ATAATAAAGG TAAGAATAAT ATTATATTTC ATAATGTGAA TAATGATAAT   
  
  
- AATGATGATG TTATTAATAA TAATGATATC AATCATAATA ATGATAATAA TTATGATGAT GGTGATAACA   
  
  
- ATTAAACAAT AATAATGATA ATAATAATAA TAATAATAAT AATAATAATA ATAAAAATGA TGAGAATAAT   
  
  
- GATAATAATA TAATGATGAT GTTATTAATA ATAATGATAT CAATCATAAT AAGAAAAATG ATAATAATAA   
  
  
- TTAATAATTA TAATAATCAA AATCATAAAA AAATTTTAAT AATAATCTCA TTGTGATCAT GATATTTTAA   
  
  
- CTGTAAGAAA AGAAGGGGGG AGAGTTAGAG AGTAAGAGAG AAAGAGAGTC GGGAGAGGCG ACGAGAGTGG   
  
  
- GGAGTGTGAG AGGAAGCTCC GAATAAGTCC TTTAGGGTAT AGAGGAAACT CCGAATAAGA ACCTAAACAA   
  
  
- CAACTTGTCA AGAGATATAT ACACACACAC ACATATATAT ATATATATAT ATATATATAT ATAAAAAAAA   
  
  
- AAAAAAAAAA AAAAAAAAAA CACAAATCTT TAATCTATAA GTCTATAACT TTTCAAATGC AGTTCTAAAA   
  
  
- AGAAAAGGTC TTTTAATTTA TAAAATGGAC CTTAGAAAAC TCAAGGGTAC AGATCAATTG TTAACACACA   
  
  
- TAAAATAAAG GACTTGTTAA AAGTCAACAA TTGTACTTAA AACTTACAAA GAAAAAATAA CAGAGATCCC   
  
  
- CGTATACGTA TAATCGTCTC AACAATTGAA CAATTGATCT TCGTGTACAA ACAGTATAGA AGAAACACAA   
  
  
- TCGAAAAAAT TACCAATTAA CAGTAAGACG ACACGATATA AGATATACAC GAAACTTACG CACATCGATT   
  
  
- TTATTAGGAT TTTGACTTAA TAAACTTGAA GATCTGTTTA TGTTGAGCTA CGGGTCCATT GGAGGTTTCA   
  
  
- AAAAATTTAT TGACTTGATA GAAGTTATAT TATGGTAGTA CTGTCATTCC AATCGAACGA CTCAAATAAT   
  
  
- TTATACAATT GTCAAAGGGG TCAACAGAAC AAACAAAGAG TGAACACGGT AATCCTCCGA CCCCAAACCT   
  
  
- TGCAATCTCT TCTTTCTTTT TCCCTTTTTC TCCCTCTCTT TTTCTTATCT ATATAATATC ACTACTGACT   
  
  
- ACTCATGAAC TCGTGAACTG ATGGACTAAC AAAAATAAAA AAAGTTTTAC ATTTGAAGAT AAATAAATAA   
  
  
- ATCCATTTTG AACTATTACC GTATAATAAC TTGGTACGAG ACCGTGAAAC GTGAAACGTC TAGGACACGT   
  
  
- AAAGTCATCA GACATATTAA GGATGAACAA AATTTTTT

+     ARE

| Site Name | Organism | Position | Strand | Matrix score. | sequence | function |
| --- | --- | --- | --- | --- | --- | --- |
| ARE | Zea mays | 420 | - | 6 | AAACCA | cis-acting regulatory element essential for the anaerobic induction |

>PlantCARE\_20562   
+ TATTTTAGTT AGTTTTATGG AAGAATATTA AAATCATTTT TCATTTACAT GTCAATCTAT ATAATATGGT   
  
  
+ TAAATAATTG TATAATTTAT GCTATCTCAC ACGAAAATAC TTAACCTGTT TTATGAGATG ATATAGTAAA   
  
  
+ ATTTGAAATT CAATTTGTCA AATTTAGTTT TCATTGAGTT TTTTCGCTGG AAAAATATAT ACCTAATTTG   
  
  
+ AAGTCCCTAT AAATTAATCT GATAAGATTA TTTTACCAAA ACTGCTTAAA TTTTCATATT AAGTTGTTTC   
  
  
+ TGACTTTCTG ACGTTTCTGT GGTAAGAAAT CTTGTCTAAT GCCTTGTTTT ACAATCACAA TAGCCTATGG   
  
  
+ TTATCAACTT TTATAATAGT TTGAAGTAAA ACTTTTTTTT TGTTTGTTTT TAGAACTTTG ATGACCAGAT   
  
  
+ GGTTTGACTT TTTGTTGCAT ATTGATTAAA TTTCACATGT AAATGCTGAC TAACATTTAA AATTAAGGTT   
  
  
+ TGGCATATGA TGATGCCCAT TTCTGATGGC AGTTTGGTAT ATAATTTTTT TCAATTCAGT AAAAGCAAAT   
  
  
+ ACCCCATTTT ATGCTATTTC ACATGACCTA ACCAATTTTC TGAAATGATA TACTAAAATC TGACATTTAA   
  
  
+ TTTATTTGAA CAAAGTTTGT TATTATTTCC ATTCTTATTA TAATATAAAG TATTACACTT ATTACTATTA   
  
  
+ TTACTACTAC AATAATTATT ATTACTATAG TTAGTATTAT TACTATTATT AATACTACTA CCACTATTGT   
  
  
+ TAATTTGTTA TTATTACTAT TATTATTATT ATTATTATTA TTATTATTAT TATTTTTACT ACTCTTATTA   
  
  
+ CTATTATTAT ATTACTACTA CAATAATTAT TATTACTATA GTTAGTATTA TTCTTTTTAC TATTATTATT   
  
  
+ AATTATTAAT ATTATTAGTT TTAGTATTTT TTTAAAATTA TTATTAGAGT AACACTAGTA CTATAAAATT   
  
  
+ GACATTCTTT TCTTCCCCCC TCTCAATCTC TCATTCTCTC TTTCTCTCAG CCCTCTCCGC TGCTCTCACC   
  
  
+ CCTCACACTC TCCTTCGAGG CTTATTCAGG AAATCCCATA TCTCCTTTGA GGCTTATTCT TGGATTTGTT   
  
  
+ GTTGAACAGT TCTCTATATA TGTGTGTGTG TGTATATATA TATATATATA TATATATATA TATTTTTTTT   
  
  
+ TTTTTTTTTT TTTTTTTTTT GTGTTTAGAA ATTAGATATT CAGATATTGA AAAGTTTACG TCAAGATTTT   
  
  
+ TCTTTTCCAG AAAATTAAAT ATTTTACCTG GAATCTTTTG AGTTCCCATG TCTAGTTAAC AATTGTGTGT   
  
  
+ ATTTTATTTC CTGAACAATT TTCAGTTGTT AACATGAATT TTGAATGTTT CTTTTTTATT GTCTCTAGGG   
  
  
+ GCATATGCAT ATTAGCAGAG TTGTTAACTT GTTAACTAGA AGCACATGTT TGTCATATCT TCTTTGTGTT   
  
  
+ AGCTTTTTTA ATGGTTAATT GTCATTCTGC TGTGCTATAT TCTATATGTG CTTTGAATGC GTGTAGCTAA   
  
  
+ AATAATCCTA AAACTGAATT ATTTGAACTT CTAGACAAAT ACAACTCGAT GCCCAGGTAA CCTCCAAAGT   
  
  
+ TTTTTAAATA ACTGAACTAT CTTCAATATA ATACCATCAT GACAGTAAGG TTAGCTTGCT GAGTTTATTA   
  
  
+ AATATGTTAA CAGTTTCCCC AGTTGTCTTG TTTGTTTCTC ACTTGTGCCA TTAGGAGGCT GGGGTTTGGA   
  
  
+ ACGTTAGAGA AGAAAGAAAA AGGGAAAAAG AGGGAGAGAA AAAGAATAGA TATATTATAG TGATGACTGA   
  
  
+ TGAGTACTTG AGCACTTGAC TACCTGATTG TTTTTATTTT TTTCAAAATG TAAACTTCTA TTTATTTATT   
  
  
+ TAGGTAAAAC TTGATAATGG CATATTATTG AACCATGCTC TGGCACTTTG CACTTTGCAG ATCCTGTGCA   
  
  
+ TTTCAGTAGT CTGTATAATT CCTACTTGTT TTAAAAAA  

- ATAAAATCAA TCAAAATACC TTCTTATAAT TTTAGTAAAA AGTAAATGTA CAGTTAGATA TATTATACCA   
  
  
- ATTTATTAAC ATATTAAATA CGATAGAGTG TGCTTTTATG AATTGGACAA AATACTCTAC TATATCATTT   
  
  
- TAAACTTTAA GTTAAACAGT TTAAATCAAA AGTAACTCAA AAAAGCGACC TTTTTATATA TGGATTAAAC   
  
  
- TTCAGGGATA TTTAATTAGA CTATTCTAAT AAAATGGTTT TGACGAATTT AAAAGTATAA TTCAACAAAG   
  
  
- ACTGAAAGAC TGCAAAGACA CCATTCTTTA GAACAGATTA CGGAACAAAA TGTTAGTGTT ATCGGATACC   
  
  
- AATAGTTGAA AATATTATCA AACTTCATTT TGAAAAAAAA ACAAACAAAA ATCTTGAAAC TACTGGTCTA   
  
  
- CCAAACTGAA AAACAACGTA TAACTAATTT AAAGTGTACA TTTACGACTG ATTGTAAATT TTAATTCCAA   
  
  
- ACCGTATACT ACTACGGGTA AAGACTACCG TCAAACCATA TATTAAAAAA AGTTAAGTCA TTTTCGTTTA   
  
  
- TGGGGTAAAA TACGATAAAG TGTACTGGAT TGGTTAAAAG ACTTTACTAT ATGATTTTAG ACTGTAAATT   
  
  
- AAATAAACTT GTTTCAAACA ATAATAAAGG TAAGAATAAT ATTATATTTC ATAATGTGAA TAATGATAAT   
  
  
- AATGATGATG TTATTAATAA TAATGATATC AATCATAATA ATGATAATAA TTATGATGAT GGTGATAACA   
  
  
- ATTAAACAAT AATAATGATA ATAATAATAA TAATAATAAT AATAATAATA ATAAAAATGA TGAGAATAAT   
  
  
- GATAATAATA TAATGATGAT GTTATTAATA ATAATGATAT CAATCATAAT AAGAAAAATG ATAATAATAA   
  
  
- TTAATAATTA TAATAATCAA AATCATAAAA AAATTTTAAT AATAATCTCA TTGTGATCAT GATATTTTAA   
  
  
- CTGTAAGAAA AGAAGGGGGG AGAGTTAGAG AGTAAGAGAG AAAGAGAGTC GGGAGAGGCG ACGAGAGTGG   
  
  
- GGAGTGTGAG AGGAAGCTCC GAATAAGTCC TTTAGGGTAT AGAGGAAACT CCGAATAAGA ACCTAAACAA   
  
  
- CAACTTGTCA AGAGATATAT ACACACACAC ACATATATAT ATATATATAT ATATATATAT ATAAAAAAAA   
  
  
- AAAAAAAAAA AAAAAAAAAA CACAAATCTT TAATCTATAA GTCTATAACT TTTCAAATGC AGTTCTAAAA   
  
  
- AGAAAAGGTC TTTTAATTTA TAAAATGGAC CTTAGAAAAC TCAAGGGTAC AGATCAATTG TTAACACACA   
  
  
- TAAAATAAAG GACTTGTTAA AAGTCAACAA TTGTACTTAA AACTTACAAA GAAAAAATAA CAGAGATCCC   
  
  
- CGTATACGTA TAATCGTCTC AACAATTGAA CAATTGATCT TCGTGTACAA ACAGTATAGA AGAAACACAA   
  
  
- TCGAAAAAAT TACCAATTAA CAGTAAGACG ACACGATATA AGATATACAC GAAACTTACG CACATCGATT   
  
  
- TTATTAGGAT TTTGACTTAA TAAACTTGAA GATCTGTTTA TGTTGAGCTA CGGGTCCATT GGAGGTTTCA   
  
  
- AAAAATTTAT TGACTTGATA GAAGTTATAT TATGGTAGTA CTGTCATTCC AATCGAACGA CTCAAATAAT   
  
  
- TTATACAATT GTCAAAGGGG TCAACAGAAC AAACAAAGAG TGAACACGGT AATCCTCCGA CCCCAAACCT   
  
  
- TGCAATCTCT TCTTTCTTTT TCCCTTTTTC TCCCTCTCTT TTTCTTATCT ATATAATATC ACTACTGACT   
  
  
- ACTCATGAAC TCGTGAACTG ATGGACTAAC AAAAATAAAA AAAGTTTTAC ATTTGAAGAT AAATAAATAA   
  
  
- ATCCATTTTG AACTATTACC GTATAATAAC TTGGTACGAG ACCGTGAAAC GTGAAACGTC TAGGACACGT   
  
  
- AAAGTCATCA GACATATTAA GGATGAACAA AATTTTTT

+     AT~TATA-box

| Site Name | Organism | Position | Strand | Matrix score. | sequence | function |
| --- | --- | --- | --- | --- | --- | --- |
| AT~TATA-box | Arabidopsis thaliana | 1155 | - | 6 | TATATA |  |
| AT~TATA-box | Arabidopsis thaliana | 1165 | - | 6 | TATATA |  |
| AT~TATA-box | Arabidopsis thaliana | 1157 | - | 6 | TATATA |  |
| AT~TATA-box | Arabidopsis thaliana | 1173 | - | 6 | TATATA |  |
| AT~TATA-box | Arabidopsis thaliana | 528 | + | 6 | TATATA |  |
| AT~TATA-box | Arabidopsis thaliana | 1169 | - | 6 | TATATA |  |
| AT~TATA-box | Arabidopsis thaliana | 1161 | - | 6 | TATATA |  |
| AT~TATA-box | Arabidopsis thaliana | 1177 | - | 6 | TATATA |  |
| AT~TATA-box | Arabidopsis thaliana | 58 | + | 6 | TATATA |  |
| AT~TATA-box | Arabidopsis thaliana | 1167 | - | 6 | TATATA |  |
| AT~TATA-box | Arabidopsis thaliana | 1159 | - | 6 | TATATA |  |
| AT~TATA-box | Arabidopsis thaliana | 1175 | - | 6 | TATATA |  |
| AT~TATA-box | Arabidopsis thaliana | 1153 | - | 6 | TATATA |  |
| AT~TATA-box | Arabidopsis thaliana | 1171 | - | 6 | TATATA |  |
| AT~TATA-box | Arabidopsis thaliana | 1163 | - | 6 | TATATA |  |
| AT~TATA-box | Arabidopsis thaliana | 196 | + | 6 | TATATA |  |
| AT~TATA-box | Arabidopsis thaliana | 1135 | - | 6 | TATATA |  |

>PlantCARE\_20562   
+ TATTTTAGTT AGTTTTATGG AAGAATATTA AAATCATTTT TCATTTACAT GTCAATCTAT ATAATATGGT   
  
  
+ TAAATAATTG TATAATTTAT GCTATCTCAC ACGAAAATAC TTAACCTGTT TTATGAGATG ATATAGTAAA   
  
  
+ ATTTGAAATT CAATTTGTCA AATTTAGTTT TCATTGAGTT TTTTCGCTGG AAAAATATAT ACCTAATTTG   
  
  
+ AAGTCCCTAT AAATTAATCT GATAAGATTA TTTTACCAAA ACTGCTTAAA TTTTCATATT AAGTTGTTTC   
  
  
+ TGACTTTCTG ACGTTTCTGT GGTAAGAAAT CTTGTCTAAT GCCTTGTTTT ACAATCACAA TAGCCTATGG   
  
  
+ TTATCAACTT TTATAATAGT TTGAAGTAAA ACTTTTTTTT TGTTTGTTTT TAGAACTTTG ATGACCAGAT   
  
  
+ GGTTTGACTT TTTGTTGCAT ATTGATTAAA TTTCACATGT AAATGCTGAC TAACATTTAA AATTAAGGTT   
  
  
+ TGGCATATGA TGATGCCCAT TTCTGATGGC AGTTTGGTAT ATAATTTTTT TCAATTCAGT AAAAGCAAAT   
  
  
+ ACCCCATTTT ATGCTATTTC ACATGACCTA ACCAATTTTC TGAAATGATA TACTAAAATC TGACATTTAA   
  
  
+ TTTATTTGAA CAAAGTTTGT TATTATTTCC ATTCTTATTA TAATATAAAG TATTACACTT ATTACTATTA   
  
  
+ TTACTACTAC AATAATTATT ATTACTATAG TTAGTATTAT TACTATTATT AATACTACTA CCACTATTGT   
  
  
+ TAATTTGTTA TTATTACTAT TATTATTATT ATTATTATTA TTATTATTAT TATTTTTACT ACTCTTATTA   
  
  
+ CTATTATTAT ATTACTACTA CAATAATTAT TATTACTATA GTTAGTATTA TTCTTTTTAC TATTATTATT   
  
  
+ AATTATTAAT ATTATTAGTT TTAGTATTTT TTTAAAATTA TTATTAGAGT AACACTAGTA CTATAAAATT   
  
  
+ GACATTCTTT TCTTCCCCCC TCTCAATCTC TCATTCTCTC TTTCTCTCAG CCCTCTCCGC TGCTCTCACC   
  
  
+ CCTCACACTC TCCTTCGAGG CTTATTCAGG AAATCCCATA TCTCCTTTGA GGCTTATTCT TGGATTTGTT   
  
  
+ GTTGAACAGT TCTCTATATA TGTGTGTGTG TGTATATATA TATATATATA TATATATATA TATTTTTTTT   
  
  
+ TTTTTTTTTT TTTTTTTTTT GTGTTTAGAA ATTAGATATT CAGATATTGA AAAGTTTACG TCAAGATTTT   
  
  
+ TCTTTTCCAG AAAATTAAAT ATTTTACCTG GAATCTTTTG AGTTCCCATG TCTAGTTAAC AATTGTGTGT   
  
  
+ ATTTTATTTC CTGAACAATT TTCAGTTGTT AACATGAATT TTGAATGTTT CTTTTTTATT GTCTCTAGGG   
  
  
+ GCATATGCAT ATTAGCAGAG TTGTTAACTT GTTAACTAGA AGCACATGTT TGTCATATCT TCTTTGTGTT   
  
  
+ AGCTTTTTTA ATGGTTAATT GTCATTCTGC TGTGCTATAT TCTATATGTG CTTTGAATGC GTGTAGCTAA   
  
  
+ AATAATCCTA AAACTGAATT ATTTGAACTT CTAGACAAAT ACAACTCGAT GCCCAGGTAA CCTCCAAAGT   
  
  
+ TTTTTAAATA ACTGAACTAT CTTCAATATA ATACCATCAT GACAGTAAGG TTAGCTTGCT GAGTTTATTA   
  
  
+ AATATGTTAA CAGTTTCCCC AGTTGTCTTG TTTGTTTCTC ACTTGTGCCA TTAGGAGGCT GGGGTTTGGA   
  
  
+ ACGTTAGAGA AGAAAGAAAA AGGGAAAAAG AGGGAGAGAA AAAGAATAGA TATATTATAG TGATGACTGA   
  
  
+ TGAGTACTTG AGCACTTGAC TACCTGATTG TTTTTATTTT TTTCAAAATG TAAACTTCTA TTTATTTATT   
  
  
+ TAGGTAAAAC TTGATAATGG CATATTATTG AACCATGCTC TGGCACTTTG CACTTTGCAG ATCCTGTGCA   
  
  
+ TTTCAGTAGT CTGTATAATT CCTACTTGTT TTAAAAAA  

- ATAAAATCAA TCAAAATACC TTCTTATAAT TTTAGTAAAA AGTAAATGTA CAGTTAGATA TATTATACCA   
  
  
- ATTTATTAAC ATATTAAATA CGATAGAGTG TGCTTTTATG AATTGGACAA AATACTCTAC TATATCATTT   
  
  
- TAAACTTTAA GTTAAACAGT TTAAATCAAA AGTAACTCAA AAAAGCGACC TTTTTATATA TGGATTAAAC   
  
  
- TTCAGGGATA TTTAATTAGA CTATTCTAAT AAAATGGTTT TGACGAATTT AAAAGTATAA TTCAACAAAG   
  
  
- ACTGAAAGAC TGCAAAGACA CCATTCTTTA GAACAGATTA CGGAACAAAA TGTTAGTGTT ATCGGATACC   
  
  
- AATAGTTGAA AATATTATCA AACTTCATTT TGAAAAAAAA ACAAACAAAA ATCTTGAAAC TACTGGTCTA   
  
  
- CCAAACTGAA AAACAACGTA TAACTAATTT AAAGTGTACA TTTACGACTG ATTGTAAATT TTAATTCCAA   
  
  
- ACCGTATACT ACTACGGGTA AAGACTACCG TCAAACCATA TATTAAAAAA AGTTAAGTCA TTTTCGTTTA   
  
  
- TGGGGTAAAA TACGATAAAG TGTACTGGAT TGGTTAAAAG ACTTTACTAT ATGATTTTAG ACTGTAAATT   
  
  
- AAATAAACTT GTTTCAAACA ATAATAAAGG TAAGAATAAT ATTATATTTC ATAATGTGAA TAATGATAAT   
  
  
- AATGATGATG TTATTAATAA TAATGATATC AATCATAATA ATGATAATAA TTATGATGAT GGTGATAACA   
  
  
- ATTAAACAAT AATAATGATA ATAATAATAA TAATAATAAT AATAATAATA ATAAAAATGA TGAGAATAAT   
  
  
- GATAATAATA TAATGATGAT GTTATTAATA ATAATGATAT CAATCATAAT AAGAAAAATG ATAATAATAA   
  
  
- TTAATAATTA TAATAATCAA AATCATAAAA AAATTTTAAT AATAATCTCA TTGTGATCAT GATATTTTAA   
  
  
- CTGTAAGAAA AGAAGGGGGG AGAGTTAGAG AGTAAGAGAG AAAGAGAGTC GGGAGAGGCG ACGAGAGTGG   
  
  
- GGAGTGTGAG AGGAAGCTCC GAATAAGTCC TTTAGGGTAT AGAGGAAACT CCGAATAAGA ACCTAAACAA   
  
  
- CAACTTGTCA AGAGATATAT ACACACACAC ACATATATAT ATATATATAT ATATATATAT ATAAAAAAAA   
  
  
- AAAAAAAAAA AAAAAAAAAA CACAAATCTT TAATCTATAA GTCTATAACT TTTCAAATGC AGTTCTAAAA   
  
  
- AGAAAAGGTC TTTTAATTTA TAAAATGGAC CTTAGAAAAC TCAAGGGTAC AGATCAATTG TTAACACACA   
  
  
- TAAAATAAAG GACTTGTTAA AAGTCAACAA TTGTACTTAA AACTTACAAA GAAAAAATAA CAGAGATCCC   
  
  
- CGTATACGTA TAATCGTCTC AACAATTGAA CAATTGATCT TCGTGTACAA ACAGTATAGA AGAAACACAA   
  
  
- TCGAAAAAAT TACCAATTAA CAGTAAGACG ACACGATATA AGATATACAC GAAACTTACG CACATCGATT   
  
  
- TTATTAGGAT TTTGACTTAA TAAACTTGAA GATCTGTTTA TGTTGAGCTA CGGGTCCATT GGAGGTTTCA   
  
  
- AAAAATTTAT TGACTTGATA GAAGTTATAT TATGGTAGTA CTGTCATTCC AATCGAACGA CTCAAATAAT   
  
  
- TTATACAATT GTCAAAGGGG TCAACAGAAC AAACAAAGAG TGAACACGGT AATCCTCCGA CCCCAAACCT   
  
  
- TGCAATCTCT TCTTTCTTTT TCCCTTTTTC TCCCTCTCTT TTTCTTATCT ATATAATATC ACTACTGACT   
  
  
- ACTCATGAAC TCGTGAACTG ATGGACTAAC AAAAATAAAA AAAGTTTTAC ATTTGAAGAT AAATAAATAA   
  
  
- ATCCATTTTG AACTATTACC GTATAATAAC TTGGTACGAG ACCGTGAAAC GTGAAACGTC TAGGACACGT   
  
  
- AAAGTCATCA GACATATTAA GGATGAACAA AATTTTTT

+     Box 4

| Site Name | Organism | Position | Strand | Matrix score. | sequence | function |
| --- | --- | --- | --- | --- | --- | --- |
| Box 4 | Petroselinum crispum | 223 | + | 6 | ATTAAT | part of a conserved DNA module involved in light responsiveness |
| Box 4 | Petroselinum crispum | 908 | + | 6 | ATTAAT | part of a conserved DNA module involved in light responsiveness |
| Box 4 | Petroselinum crispum | 748 | + | 6 | ATTAAT | part of a conserved DNA module involved in light responsiveness |
| Box 4 | Petroselinum crispum | 915 | + | 6 | ATTAAT | part of a conserved DNA module involved in light responsiveness |

>PlantCARE\_20562   
+ TATTTTAGTT AGTTTTATGG AAGAATATTA AAATCATTTT TCATTTACAT GTCAATCTAT ATAATATGGT   
  
  
+ TAAATAATTG TATAATTTAT GCTATCTCAC ACGAAAATAC TTAACCTGTT TTATGAGATG ATATAGTAAA   
  
  
+ ATTTGAAATT CAATTTGTCA AATTTAGTTT TCATTGAGTT TTTTCGCTGG AAAAATATAT ACCTAATTTG   
  
  
+ AAGTCCCTAT AAATTAATCT GATAAGATTA TTTTACCAAA ACTGCTTAAA TTTTCATATT AAGTTGTTTC   
  
  
+ TGACTTTCTG ACGTTTCTGT GGTAAGAAAT CTTGTCTAAT GCCTTGTTTT ACAATCACAA TAGCCTATGG   
  
  
+ TTATCAACTT TTATAATAGT TTGAAGTAAA ACTTTTTTTT TGTTTGTTTT TAGAACTTTG ATGACCAGAT   
  
  
+ GGTTTGACTT TTTGTTGCAT ATTGATTAAA TTTCACATGT AAATGCTGAC TAACATTTAA AATTAAGGTT   
  
  
+ TGGCATATGA TGATGCCCAT TTCTGATGGC AGTTTGGTAT ATAATTTTTT TCAATTCAGT AAAAGCAAAT   
  
  
+ ACCCCATTTT ATGCTATTTC ACATGACCTA ACCAATTTTC TGAAATGATA TACTAAAATC TGACATTTAA   
  
  
+ TTTATTTGAA CAAAGTTTGT TATTATTTCC ATTCTTATTA TAATATAAAG TATTACACTT ATTACTATTA   
  
  
+ TTACTACTAC AATAATTATT ATTACTATAG TTAGTATTAT TACTATTATT AATACTACTA CCACTATTGT   
  
  
+ TAATTTGTTA TTATTACTAT TATTATTATT ATTATTATTA TTATTATTAT TATTTTTACT ACTCTTATTA   
  
  
+ CTATTATTAT ATTACTACTA CAATAATTAT TATTACTATA GTTAGTATTA TTCTTTTTAC TATTATTATT   
  
  
+ AATTATTAAT ATTATTAGTT TTAGTATTTT TTTAAAATTA TTATTAGAGT AACACTAGTA CTATAAAATT   
  
  
+ GACATTCTTT TCTTCCCCCC TCTCAATCTC TCATTCTCTC TTTCTCTCAG CCCTCTCCGC TGCTCTCACC   
  
  
+ CCTCACACTC TCCTTCGAGG CTTATTCAGG AAATCCCATA TCTCCTTTGA GGCTTATTCT TGGATTTGTT   
  
  
+ GTTGAACAGT TCTCTATATA TGTGTGTGTG TGTATATATA TATATATATA TATATATATA TATTTTTTTT   
  
  
+ TTTTTTTTTT TTTTTTTTTT GTGTTTAGAA ATTAGATATT CAGATATTGA AAAGTTTACG TCAAGATTTT   
  
  
+ TCTTTTCCAG AAAATTAAAT ATTTTACCTG GAATCTTTTG AGTTCCCATG TCTAGTTAAC AATTGTGTGT   
  
  
+ ATTTTATTTC CTGAACAATT TTCAGTTGTT AACATGAATT TTGAATGTTT CTTTTTTATT GTCTCTAGGG   
  
  
+ GCATATGCAT ATTAGCAGAG TTGTTAACTT GTTAACTAGA AGCACATGTT TGTCATATCT TCTTTGTGTT   
  
  
+ AGCTTTTTTA ATGGTTAATT GTCATTCTGC TGTGCTATAT TCTATATGTG CTTTGAATGC GTGTAGCTAA   
  
  
+ AATAATCCTA AAACTGAATT ATTTGAACTT CTAGACAAAT ACAACTCGAT GCCCAGGTAA CCTCCAAAGT   
  
  
+ TTTTTAAATA ACTGAACTAT CTTCAATATA ATACCATCAT GACAGTAAGG TTAGCTTGCT GAGTTTATTA   
  
  
+ AATATGTTAA CAGTTTCCCC AGTTGTCTTG TTTGTTTCTC ACTTGTGCCA TTAGGAGGCT GGGGTTTGGA   
  
  
+ ACGTTAGAGA AGAAAGAAAA AGGGAAAAAG AGGGAGAGAA AAAGAATAGA TATATTATAG TGATGACTGA   
  
  
+ TGAGTACTTG AGCACTTGAC TACCTGATTG TTTTTATTTT TTTCAAAATG TAAACTTCTA TTTATTTATT   
  
  
+ TAGGTAAAAC TTGATAATGG CATATTATTG AACCATGCTC TGGCACTTTG CACTTTGCAG ATCCTGTGCA   
  
  
+ TTTCAGTAGT CTGTATAATT CCTACTTGTT TTAAAAAA  

- ATAAAATCAA TCAAAATACC TTCTTATAAT TTTAGTAAAA AGTAAATGTA CAGTTAGATA TATTATACCA   
  
  
- ATTTATTAAC ATATTAAATA CGATAGAGTG TGCTTTTATG AATTGGACAA AATACTCTAC TATATCATTT   
  
  
- TAAACTTTAA GTTAAACAGT TTAAATCAAA AGTAACTCAA AAAAGCGACC TTTTTATATA TGGATTAAAC   
  
  
- TTCAGGGATA TTTAATTAGA CTATTCTAAT AAAATGGTTT TGACGAATTT AAAAGTATAA TTCAACAAAG   
  
  
- ACTGAAAGAC TGCAAAGACA CCATTCTTTA GAACAGATTA CGGAACAAAA TGTTAGTGTT ATCGGATACC   
  
  
- AATAGTTGAA AATATTATCA AACTTCATTT TGAAAAAAAA ACAAACAAAA ATCTTGAAAC TACTGGTCTA   
  
  
- CCAAACTGAA AAACAACGTA TAACTAATTT AAAGTGTACA TTTACGACTG ATTGTAAATT TTAATTCCAA   
  
  
- ACCGTATACT ACTACGGGTA AAGACTACCG TCAAACCATA TATTAAAAAA AGTTAAGTCA TTTTCGTTTA   
  
  
- TGGGGTAAAA TACGATAAAG TGTACTGGAT TGGTTAAAAG ACTTTACTAT ATGATTTTAG ACTGTAAATT   
  
  
- AAATAAACTT GTTTCAAACA ATAATAAAGG TAAGAATAAT ATTATATTTC ATAATGTGAA TAATGATAAT   
  
  
- AATGATGATG TTATTAATAA TAATGATATC AATCATAATA ATGATAATAA TTATGATGAT GGTGATAACA   
  
  
- ATTAAACAAT AATAATGATA ATAATAATAA TAATAATAAT AATAATAATA ATAAAAATGA TGAGAATAAT   
  
  
- GATAATAATA TAATGATGAT GTTATTAATA ATAATGATAT CAATCATAAT AAGAAAAATG ATAATAATAA   
  
  
- TTAATAATTA TAATAATCAA AATCATAAAA AAATTTTAAT AATAATCTCA TTGTGATCAT GATATTTTAA   
  
  
- CTGTAAGAAA AGAAGGGGGG AGAGTTAGAG AGTAAGAGAG AAAGAGAGTC GGGAGAGGCG ACGAGAGTGG   
  
  
- GGAGTGTGAG AGGAAGCTCC GAATAAGTCC TTTAGGGTAT AGAGGAAACT CCGAATAAGA ACCTAAACAA   
  
  
- CAACTTGTCA AGAGATATAT ACACACACAC ACATATATAT ATATATATAT ATATATATAT ATAAAAAAAA   
  
  
- AAAAAAAAAA AAAAAAAAAA CACAAATCTT TAATCTATAA GTCTATAACT TTTCAAATGC AGTTCTAAAA   
  
  
- AGAAAAGGTC TTTTAATTTA TAAAATGGAC CTTAGAAAAC TCAAGGGTAC AGATCAATTG TTAACACACA   
  
  
- TAAAATAAAG GACTTGTTAA AAGTCAACAA TTGTACTTAA AACTTACAAA GAAAAAATAA CAGAGATCCC   
  
  
- CGTATACGTA TAATCGTCTC AACAATTGAA CAATTGATCT TCGTGTACAA ACAGTATAGA AGAAACACAA   
  
  
- TCGAAAAAAT TACCAATTAA CAGTAAGACG ACACGATATA AGATATACAC GAAACTTACG CACATCGATT   
  
  
- TTATTAGGAT TTTGACTTAA TAAACTTGAA GATCTGTTTA TGTTGAGCTA CGGGTCCATT GGAGGTTTCA   
  
  
- AAAAATTTAT TGACTTGATA GAAGTTATAT TATGGTAGTA CTGTCATTCC AATCGAACGA CTCAAATAAT   
  
  
- TTATACAATT GTCAAAGGGG TCAACAGAAC AAACAAAGAG TGAACACGGT AATCCTCCGA CCCCAAACCT   
  
  
- TGCAATCTCT TCTTTCTTTT TCCCTTTTTC TCCCTCTCTT TTTCTTATCT ATATAATATC ACTACTGACT   
  
  
- ACTCATGAAC TCGTGAACTG ATGGACTAAC AAAAATAAAA AAAGTTTTAC ATTTGAAGAT AAATAAATAA   
  
  
- ATCCATTTTG AACTATTACC GTATAATAAC TTGGTACGAG ACCGTGAAAC GTGAAACGTC TAGGACACGT   
  
  
- AAAGTCATCA GACATATTAA GGATGAACAA AATTTTTT

+     CAAT-box

| Site Name | Organism | Position | Strand | Matrix score. | sequence | function |
| --- | --- | --- | --- | --- | --- | --- |
| CAAT-box | Nicotiana glutinosa | 53 | + | 4 | CAAT |  |
| CAAT-box | Nicotiana glutinosa | 332 | + | 4 | CAAT |  |
| CAAT-box | Pisum sativum | 153 | - | 5 | CAAAT | common cis-acting element in promoter and enhancer regions |
| CAAT-box | Arabidopsis thaliana | 592 | + | 5 | CCAAT | common cis-acting element in promoter and enhancer regions |
| CAAT-box | Pisum sativum | 141 | - | 5 | CAAAT | common cis-acting element in promoter and enhancer regions |
| CAAT-box | Nicotiana glutinosa | 441 | - | 4 | CAAT |  |
| CAAT-box | Nicotiana glutinosa | 173 | - | 4 | CAAT |  |
| CAAT-box | Nicotiana glutinosa | 593 | + | 4 | CAAT |  |
| CAAT-box | Nicotiana glutinosa | 77 | - | 4 | CAAT |  |
| CAAT-box | Nicotiana glutinosa | 338 | + | 4 | CAAT |  |
| CAAT-box | Pisum sativum | 159 | + | 5 | CAAAT | common cis-acting element in promoter and enhancer regions |
| CAAT-box | Pisum sativum | 556 | + | 5 | CAAAT | common cis-acting element in promoter and enhancer regions |
| CAAT-box | Nicotiana glutinosa | 151 | + | 4 | CAAT |  |
| CAAT-box | Nicotiana glutinosa | 542 | + | 4 | CAAT |  |
| CAAT-box | Pisum sativum | 206 | - | 5 | CAAAT | common cis-acting element in promoter and enhancer regions |
| CAAT-box | Pisum sativum | 634 | - | 5 | CAAAT | common cis-acting element in promoter and enhancer regions |
| CAAT-box | Nicotiana glutinosa | 710 | + | 4 | CAAT |  |
| CAAT-box | Nicotiana glutinosa | 766 | - | 4 | CAAT |  |
| CAAT-box | Pisum sativum | 773 | - | 5 | CAAAT | common cis-acting element in promoter and enhancer regions |
| CAAT-box | Nicotiana glutinosa | 861 | + | 4 | CAAT |  |
| CAAT-box | Nicotiana glutinosa | 978 | - | 4 | CAAT |  |
| CAAT-box | Nicotiana glutinosa | 1004 | + | 4 | CAAT |  |
| CAAT-box | Pisum sativum | 1114 | - | 5 | CAAAT | common cis-acting element in promoter and enhancer regions |
| CAAT-box | Nicotiana glutinosa | 1236 | - | 4 | CAAT |  |
| CAAT-box | Nicotiana glutinosa | 1320 | + | 4 | CAAT |  |
| CAAT-box | Nicotiana glutinosa | 1322 | - | 4 | CAAT |  |
| CAAT-box | Nicotiana glutinosa | 1346 | + | 4 | CAAT |  |
| CAAT-box | Nicotiana glutinosa | 1388 | - | 4 | CAAT |  |
| CAAT-box | Nicotiana glutinosa | 1488 | - | 4 | CAAT |  |
| CAAT-box | Pisum sativum | 1561 | - | 5 | CAAAT | common cis-acting element in promoter and enhancer regions |
| CAAT-box | Pisum sativum | 1576 | + | 5 | CAAAT | common cis-acting element in promoter and enhancer regions |
| CAAT-box | Nicotiana glutinosa | 1634 | + | 4 | CAAT |  |
| CAAT-box | Nicotiana glutinosa | 1847 | - | 4 | CAAT |  |
| CAAT-box | Nicotiana glutinosa | 1917 | - | 4 | CAAT |  |

>PlantCARE\_20562   
+ TATTTTAGTT AGTTTTATGG AAGAATATTA AAATCATTTT TCATTTACAT GTCAATCTAT ATAATATGGT   
  
  
+ TAAATAATTG TATAATTTAT GCTATCTCAC ACGAAAATAC TTAACCTGTT TTATGAGATG ATATAGTAAA   
  
  
+ ATTTGAAATT CAATTTGTCA AATTTAGTTT TCATTGAGTT TTTTCGCTGG AAAAATATAT ACCTAATTTG   
  
  
+ AAGTCCCTAT AAATTAATCT GATAAGATTA TTTTACCAAA ACTGCTTAAA TTTTCATATT AAGTTGTTTC   
  
  
+ TGACTTTCTG ACGTTTCTGT GGTAAGAAAT CTTGTCTAAT GCCTTGTTTT ACAATCACAA TAGCCTATGG   
  
  
+ TTATCAACTT TTATAATAGT TTGAAGTAAA ACTTTTTTTT TGTTTGTTTT TAGAACTTTG ATGACCAGAT   
  
  
+ GGTTTGACTT TTTGTTGCAT ATTGATTAAA TTTCACATGT AAATGCTGAC TAACATTTAA AATTAAGGTT   
  
  
+ TGGCATATGA TGATGCCCAT TTCTGATGGC AGTTTGGTAT ATAATTTTTT TCAATTCAGT AAAAGCAAAT   
  
  
+ ACCCCATTTT ATGCTATTTC ACATGACCTA ACCAATTTTC TGAAATGATA TACTAAAATC TGACATTTAA   
  
  
+ TTTATTTGAA CAAAGTTTGT TATTATTTCC ATTCTTATTA TAATATAAAG TATTACACTT ATTACTATTA   
  
  
+ TTACTACTAC AATAATTATT ATTACTATAG TTAGTATTAT TACTATTATT AATACTACTA CCACTATTGT   
  
  
+ TAATTTGTTA TTATTACTAT TATTATTATT ATTATTATTA TTATTATTAT TATTTTTACT ACTCTTATTA   
  
  
+ CTATTATTAT ATTACTACTA CAATAATTAT TATTACTATA GTTAGTATTA TTCTTTTTAC TATTATTATT   
  
  
+ AATTATTAAT ATTATTAGTT TTAGTATTTT TTTAAAATTA TTATTAGAGT AACACTAGTA CTATAAAATT   
  
  
+ GACATTCTTT TCTTCCCCCC TCTCAATCTC TCATTCTCTC TTTCTCTCAG CCCTCTCCGC TGCTCTCACC   
  
  
+ CCTCACACTC TCCTTCGAGG CTTATTCAGG AAATCCCATA TCTCCTTTGA GGCTTATTCT TGGATTTGTT   
  
  
+ GTTGAACAGT TCTCTATATA TGTGTGTGTG TGTATATATA TATATATATA TATATATATA TATTTTTTTT   
  
  
+ TTTTTTTTTT TTTTTTTTTT GTGTTTAGAA ATTAGATATT CAGATATTGA AAAGTTTACG TCAAGATTTT   
  
  
+ TCTTTTCCAG AAAATTAAAT ATTTTACCTG GAATCTTTTG AGTTCCCATG TCTAGTTAAC AATTGTGTGT   
  
  
+ ATTTTATTTC CTGAACAATT TTCAGTTGTT AACATGAATT TTGAATGTTT CTTTTTTATT GTCTCTAGGG   
  
  
+ GCATATGCAT ATTAGCAGAG TTGTTAACTT GTTAACTAGA AGCACATGTT TGTCATATCT TCTTTGTGTT   
  
  
+ AGCTTTTTTA ATGGTTAATT GTCATTCTGC TGTGCTATAT TCTATATGTG CTTTGAATGC GTGTAGCTAA   
  
  
+ AATAATCCTA AAACTGAATT ATTTGAACTT CTAGACAAAT ACAACTCGAT GCCCAGGTAA CCTCCAAAGT   
  
  
+ TTTTTAAATA ACTGAACTAT CTTCAATATA ATACCATCAT GACAGTAAGG TTAGCTTGCT GAGTTTATTA   
  
  
+ AATATGTTAA CAGTTTCCCC AGTTGTCTTG TTTGTTTCTC ACTTGTGCCA TTAGGAGGCT GGGGTTTGGA   
  
  
+ ACGTTAGAGA AGAAAGAAAA AGGGAAAAAG AGGGAGAGAA AAAGAATAGA TATATTATAG TGATGACTGA   
  
  
+ TGAGTACTTG AGCACTTGAC TACCTGATTG TTTTTATTTT TTTCAAAATG TAAACTTCTA TTTATTTATT   
  
  
+ TAGGTAAAAC TTGATAATGG CATATTATTG AACCATGCTC TGGCACTTTG CACTTTGCAG ATCCTGTGCA   
  
  
+ TTTCAGTAGT CTGTATAATT CCTACTTGTT TTAAAAAA  

- ATAAAATCAA TCAAAATACC TTCTTATAAT TTTAGTAAAA AGTAAATGTA CAGTTAGATA TATTATACCA   
  
  
- ATTTATTAAC ATATTAAATA CGATAGAGTG TGCTTTTATG AATTGGACAA AATACTCTAC TATATCATTT   
  
  
- TAAACTTTAA GTTAAACAGT TTAAATCAAA AGTAACTCAA AAAAGCGACC TTTTTATATA TGGATTAAAC   
  
  
- TTCAGGGATA TTTAATTAGA CTATTCTAAT AAAATGGTTT TGACGAATTT AAAAGTATAA TTCAACAAAG   
  
  
- ACTGAAAGAC TGCAAAGACA CCATTCTTTA GAACAGATTA CGGAACAAAA TGTTAGTGTT ATCGGATACC   
  
  
- AATAGTTGAA AATATTATCA AACTTCATTT TGAAAAAAAA ACAAACAAAA ATCTTGAAAC TACTGGTCTA   
  
  
- CCAAACTGAA AAACAACGTA TAACTAATTT AAAGTGTACA TTTACGACTG ATTGTAAATT TTAATTCCAA   
  
  
- ACCGTATACT ACTACGGGTA AAGACTACCG TCAAACCATA TATTAAAAAA AGTTAAGTCA TTTTCGTTTA   
  
  
- TGGGGTAAAA TACGATAAAG TGTACTGGAT TGGTTAAAAG ACTTTACTAT ATGATTTTAG ACTGTAAATT   
  
  
- AAATAAACTT GTTTCAAACA ATAATAAAGG TAAGAATAAT ATTATATTTC ATAATGTGAA TAATGATAAT   
  
  
- AATGATGATG TTATTAATAA TAATGATATC AATCATAATA ATGATAATAA TTATGATGAT GGTGATAACA   
  
  
- ATTAAACAAT AATAATGATA ATAATAATAA TAATAATAAT AATAATAATA ATAAAAATGA TGAGAATAAT   
  
  
- GATAATAATA TAATGATGAT GTTATTAATA ATAATGATAT CAATCATAAT AAGAAAAATG ATAATAATAA   
  
  
- TTAATAATTA TAATAATCAA AATCATAAAA AAATTTTAAT AATAATCTCA TTGTGATCAT GATATTTTAA   
  
  
- CTGTAAGAAA AGAAGGGGGG AGAGTTAGAG AGTAAGAGAG AAAGAGAGTC GGGAGAGGCG ACGAGAGTGG   
  
  
- GGAGTGTGAG AGGAAGCTCC GAATAAGTCC TTTAGGGTAT AGAGGAAACT CCGAATAAGA ACCTAAACAA   
  
  
- CAACTTGTCA AGAGATATAT ACACACACAC ACATATATAT ATATATATAT ATATATATAT ATAAAAAAAA   
  
  
- AAAAAAAAAA AAAAAAAAAA CACAAATCTT TAATCTATAA GTCTATAACT TTTCAAATGC AGTTCTAAAA   
  
  
- AGAAAAGGTC TTTTAATTTA TAAAATGGAC CTTAGAAAAC TCAAGGGTAC AGATCAATTG TTAACACACA   
  
  
- TAAAATAAAG GACTTGTTAA AAGTCAACAA TTGTACTTAA AACTTACAAA GAAAAAATAA CAGAGATCCC   
  
  
- CGTATACGTA TAATCGTCTC AACAATTGAA CAATTGATCT TCGTGTACAA ACAGTATAGA AGAAACACAA   
  
  
- TCGAAAAAAT TACCAATTAA CAGTAAGACG ACACGATATA AGATATACAC GAAACTTACG CACATCGATT   
  
  
- TTATTAGGAT TTTGACTTAA TAAACTTGAA GATCTGTTTA TGTTGAGCTA CGGGTCCATT GGAGGTTTCA   
  
  
- AAAAATTTAT TGACTTGATA GAAGTTATAT TATGGTAGTA CTGTCATTCC AATCGAACGA CTCAAATAAT   
  
  
- TTATACAATT GTCAAAGGGG TCAACAGAAC AAACAAAGAG TGAACACGGT AATCCTCCGA CCCCAAACCT   
  
  
- TGCAATCTCT TCTTTCTTTT TCCCTTTTTC TCCCTCTCTT TTTCTTATCT ATATAATATC ACTACTGACT   
  
  
- ACTCATGAAC TCGTGAACTG ATGGACTAAC AAAAATAAAA AAAGTTTTAC ATTTGAAGAT AAATAAATAA   
  
  
- ATCCATTTTG AACTATTACC GTATAATAAC TTGGTACGAG ACCGTGAAAC GTGAAACGTC TAGGACACGT   
  
  
- AAAGTCATCA GACATATTAA GGATGAACAA AATTTTTT

+     CGTCA-motif

| Site Name | Organism | Position | Strand | Matrix score. | sequence | function |
| --- | --- | --- | --- | --- | --- | --- |
| CGTCA-motif | Hordeum vulgare | 289 | - | 5 | CGTCA | cis-acting regulatory element involved in the MeJA-responsiveness |
| CGTCA-motif | Hordeum vulgare | 1249 | + | 5 | CGTCA | cis-acting regulatory element involved in the MeJA-responsiveness |

>PlantCARE\_20562   
+ TATTTTAGTT AGTTTTATGG AAGAATATTA AAATCATTTT TCATTTACAT GTCAATCTAT ATAATATGGT   
  
  
+ TAAATAATTG TATAATTTAT GCTATCTCAC ACGAAAATAC TTAACCTGTT TTATGAGATG ATATAGTAAA   
  
  
+ ATTTGAAATT CAATTTGTCA AATTTAGTTT TCATTGAGTT TTTTCGCTGG AAAAATATAT ACCTAATTTG   
  
  
+ AAGTCCCTAT AAATTAATCT GATAAGATTA TTTTACCAAA ACTGCTTAAA TTTTCATATT AAGTTGTTTC   
  
  
+ TGACTTTCTG ACGTTTCTGT GGTAAGAAAT CTTGTCTAAT GCCTTGTTTT ACAATCACAA TAGCCTATGG   
  
  
+ TTATCAACTT TTATAATAGT TTGAAGTAAA ACTTTTTTTT TGTTTGTTTT TAGAACTTTG ATGACCAGAT   
  
  
+ GGTTTGACTT TTTGTTGCAT ATTGATTAAA TTTCACATGT AAATGCTGAC TAACATTTAA AATTAAGGTT   
  
  
+ TGGCATATGA TGATGCCCAT TTCTGATGGC AGTTTGGTAT ATAATTTTTT TCAATTCAGT AAAAGCAAAT   
  
  
+ ACCCCATTTT ATGCTATTTC ACATGACCTA ACCAATTTTC TGAAATGATA TACTAAAATC TGACATTTAA   
  
  
+ TTTATTTGAA CAAAGTTTGT TATTATTTCC ATTCTTATTA TAATATAAAG TATTACACTT ATTACTATTA   
  
  
+ TTACTACTAC AATAATTATT ATTACTATAG TTAGTATTAT TACTATTATT AATACTACTA CCACTATTGT   
  
  
+ TAATTTGTTA TTATTACTAT TATTATTATT ATTATTATTA TTATTATTAT TATTTTTACT ACTCTTATTA   
  
  
+ CTATTATTAT ATTACTACTA CAATAATTAT TATTACTATA GTTAGTATTA TTCTTTTTAC TATTATTATT   
  
  
+ AATTATTAAT ATTATTAGTT TTAGTATTTT TTTAAAATTA TTATTAGAGT AACACTAGTA CTATAAAATT   
  
  
+ GACATTCTTT TCTTCCCCCC TCTCAATCTC TCATTCTCTC TTTCTCTCAG CCCTCTCCGC TGCTCTCACC   
  
  
+ CCTCACACTC TCCTTCGAGG CTTATTCAGG AAATCCCATA TCTCCTTTGA GGCTTATTCT TGGATTTGTT   
  
  
+ GTTGAACAGT TCTCTATATA TGTGTGTGTG TGTATATATA TATATATATA TATATATATA TATTTTTTTT   
  
  
+ TTTTTTTTTT TTTTTTTTTT GTGTTTAGAA ATTAGATATT CAGATATTGA AAAGTTTACG TCAAGATTTT   
  
  
+ TCTTTTCCAG AAAATTAAAT ATTTTACCTG GAATCTTTTG AGTTCCCATG TCTAGTTAAC AATTGTGTGT   
  
  
+ ATTTTATTTC CTGAACAATT TTCAGTTGTT AACATGAATT TTGAATGTTT CTTTTTTATT GTCTCTAGGG   
  
  
+ GCATATGCAT ATTAGCAGAG TTGTTAACTT GTTAACTAGA AGCACATGTT TGTCATATCT TCTTTGTGTT   
  
  
+ AGCTTTTTTA ATGGTTAATT GTCATTCTGC TGTGCTATAT TCTATATGTG CTTTGAATGC GTGTAGCTAA   
  
  
+ AATAATCCTA AAACTGAATT ATTTGAACTT CTAGACAAAT ACAACTCGAT GCCCAGGTAA CCTCCAAAGT   
  
  
+ TTTTTAAATA ACTGAACTAT CTTCAATATA ATACCATCAT GACAGTAAGG TTAGCTTGCT GAGTTTATTA   
  
  
+ AATATGTTAA CAGTTTCCCC AGTTGTCTTG TTTGTTTCTC ACTTGTGCCA TTAGGAGGCT GGGGTTTGGA   
  
  
+ ACGTTAGAGA AGAAAGAAAA AGGGAAAAAG AGGGAGAGAA AAAGAATAGA TATATTATAG TGATGACTGA   
  
  
+ TGAGTACTTG AGCACTTGAC TACCTGATTG TTTTTATTTT TTTCAAAATG TAAACTTCTA TTTATTTATT   
  
  
+ TAGGTAAAAC TTGATAATGG CATATTATTG AACCATGCTC TGGCACTTTG CACTTTGCAG ATCCTGTGCA   
  
  
+ TTTCAGTAGT CTGTATAATT CCTACTTGTT TTAAAAAA  

- ATAAAATCAA TCAAAATACC TTCTTATAAT TTTAGTAAAA AGTAAATGTA CAGTTAGATA TATTATACCA   
  
  
- ATTTATTAAC ATATTAAATA CGATAGAGTG TGCTTTTATG AATTGGACAA AATACTCTAC TATATCATTT   
  
  
- TAAACTTTAA GTTAAACAGT TTAAATCAAA AGTAACTCAA AAAAGCGACC TTTTTATATA TGGATTAAAC   
  
  
- TTCAGGGATA TTTAATTAGA CTATTCTAAT AAAATGGTTT TGACGAATTT AAAAGTATAA TTCAACAAAG   
  
  
- ACTGAAAGAC TGCAAAGACA CCATTCTTTA GAACAGATTA CGGAACAAAA TGTTAGTGTT ATCGGATACC   
  
  
- AATAGTTGAA AATATTATCA AACTTCATTT TGAAAAAAAA ACAAACAAAA ATCTTGAAAC TACTGGTCTA   
  
  
- CCAAACTGAA AAACAACGTA TAACTAATTT AAAGTGTACA TTTACGACTG ATTGTAAATT TTAATTCCAA   
  
  
- ACCGTATACT ACTACGGGTA AAGACTACCG TCAAACCATA TATTAAAAAA AGTTAAGTCA TTTTCGTTTA   
  
  
- TGGGGTAAAA TACGATAAAG TGTACTGGAT TGGTTAAAAG ACTTTACTAT ATGATTTTAG ACTGTAAATT   
  
  
- AAATAAACTT GTTTCAAACA ATAATAAAGG TAAGAATAAT ATTATATTTC ATAATGTGAA TAATGATAAT   
  
  
- AATGATGATG TTATTAATAA TAATGATATC AATCATAATA ATGATAATAA TTATGATGAT GGTGATAACA   
  
  
- ATTAAACAAT AATAATGATA ATAATAATAA TAATAATAAT AATAATAATA ATAAAAATGA TGAGAATAAT   
  
  
- GATAATAATA TAATGATGAT GTTATTAATA ATAATGATAT CAATCATAAT AAGAAAAATG ATAATAATAA   
  
  
- TTAATAATTA TAATAATCAA AATCATAAAA AAATTTTAAT AATAATCTCA TTGTGATCAT GATATTTTAA   
  
  
- CTGTAAGAAA AGAAGGGGGG AGAGTTAGAG AGTAAGAGAG AAAGAGAGTC GGGAGAGGCG ACGAGAGTGG   
  
  
- GGAGTGTGAG AGGAAGCTCC GAATAAGTCC TTTAGGGTAT AGAGGAAACT CCGAATAAGA ACCTAAACAA   
  
  
- CAACTTGTCA AGAGATATAT ACACACACAC ACATATATAT ATATATATAT ATATATATAT ATAAAAAAAA   
  
  
- AAAAAAAAAA AAAAAAAAAA CACAAATCTT TAATCTATAA GTCTATAACT TTTCAAATGC AGTTCTAAAA   
  
  
- AGAAAAGGTC TTTTAATTTA TAAAATGGAC CTTAGAAAAC TCAAGGGTAC AGATCAATTG TTAACACACA   
  
  
- TAAAATAAAG GACTTGTTAA AAGTCAACAA TTGTACTTAA AACTTACAAA GAAAAAATAA CAGAGATCCC   
  
  
- CGTATACGTA TAATCGTCTC AACAATTGAA CAATTGATCT TCGTGTACAA ACAGTATAGA AGAAACACAA   
  
  
- TCGAAAAAAT TACCAATTAA CAGTAAGACG ACACGATATA AGATATACAC GAAACTTACG CACATCGATT   
  
  
- TTATTAGGAT TTTGACTTAA TAAACTTGAA GATCTGTTTA TGTTGAGCTA CGGGTCCATT GGAGGTTTCA   
  
  
- AAAAATTTAT TGACTTGATA GAAGTTATAT TATGGTAGTA CTGTCATTCC AATCGAACGA CTCAAATAAT   
  
  
- TTATACAATT GTCAAAGGGG TCAACAGAAC AAACAAAGAG TGAACACGGT AATCCTCCGA CCCCAAACCT   
  
  
- TGCAATCTCT TCTTTCTTTT TCCCTTTTTC TCCCTCTCTT TTTCTTATCT ATATAATATC ACTACTGACT   
  
  
- ACTCATGAAC TCGTGAACTG ATGGACTAAC AAAAATAAAA AAAGTTTTAC ATTTGAAGAT AAATAAATAA   
  
  
- ATCCATTTTG AACTATTACC GTATAATAAC TTGGTACGAG ACCGTGAAAC GTGAAACGTC TAGGACACGT   
  
  
- AAAGTCATCA GACATATTAA GGATGAACAA AATTTTTT

+     ERE

| Site Name | Organism | Position | Strand | Matrix score. | sequence | function |
| --- | --- | --- | --- | --- | --- | --- |
| ERE | Nicotiana glutinos | 476 | - | 8 | ATTTTAAA |  |
| ERE | Nicotiana glutinos | 941 | - | 8 | ATTTTAAA |  |

>PlantCARE\_20562   
+ TATTTTAGTT AGTTTTATGG AAGAATATTA AAATCATTTT TCATTTACAT GTCAATCTAT ATAATATGGT   
  
  
+ TAAATAATTG TATAATTTAT GCTATCTCAC ACGAAAATAC TTAACCTGTT TTATGAGATG ATATAGTAAA   
  
  
+ ATTTGAAATT CAATTTGTCA AATTTAGTTT TCATTGAGTT TTTTCGCTGG AAAAATATAT ACCTAATTTG   
  
  
+ AAGTCCCTAT AAATTAATCT GATAAGATTA TTTTACCAAA ACTGCTTAAA TTTTCATATT AAGTTGTTTC   
  
  
+ TGACTTTCTG ACGTTTCTGT GGTAAGAAAT CTTGTCTAAT GCCTTGTTTT ACAATCACAA TAGCCTATGG   
  
  
+ TTATCAACTT TTATAATAGT TTGAAGTAAA ACTTTTTTTT TGTTTGTTTT TAGAACTTTG ATGACCAGAT   
  
  
+ GGTTTGACTT TTTGTTGCAT ATTGATTAAA TTTCACATGT AAATGCTGAC TAACATTTAA AATTAAGGTT   
  
  
+ TGGCATATGA TGATGCCCAT TTCTGATGGC AGTTTGGTAT ATAATTTTTT TCAATTCAGT AAAAGCAAAT   
  
  
+ ACCCCATTTT ATGCTATTTC ACATGACCTA ACCAATTTTC TGAAATGATA TACTAAAATC TGACATTTAA   
  
  
+ TTTATTTGAA CAAAGTTTGT TATTATTTCC ATTCTTATTA TAATATAAAG TATTACACTT ATTACTATTA   
  
  
+ TTACTACTAC AATAATTATT ATTACTATAG TTAGTATTAT TACTATTATT AATACTACTA CCACTATTGT   
  
  
+ TAATTTGTTA TTATTACTAT TATTATTATT ATTATTATTA TTATTATTAT TATTTTTACT ACTCTTATTA   
  
  
+ CTATTATTAT ATTACTACTA CAATAATTAT TATTACTATA GTTAGTATTA TTCTTTTTAC TATTATTATT   
  
  
+ AATTATTAAT ATTATTAGTT TTAGTATTTT TTTAAAATTA TTATTAGAGT AACACTAGTA CTATAAAATT   
  
  
+ GACATTCTTT TCTTCCCCCC TCTCAATCTC TCATTCTCTC TTTCTCTCAG CCCTCTCCGC TGCTCTCACC   
  
  
+ CCTCACACTC TCCTTCGAGG CTTATTCAGG AAATCCCATA TCTCCTTTGA GGCTTATTCT TGGATTTGTT   
  
  
+ GTTGAACAGT TCTCTATATA TGTGTGTGTG TGTATATATA TATATATATA TATATATATA TATTTTTTTT   
  
  
+ TTTTTTTTTT TTTTTTTTTT GTGTTTAGAA ATTAGATATT CAGATATTGA AAAGTTTACG TCAAGATTTT   
  
  
+ TCTTTTCCAG AAAATTAAAT ATTTTACCTG GAATCTTTTG AGTTCCCATG TCTAGTTAAC AATTGTGTGT   
  
  
+ ATTTTATTTC CTGAACAATT TTCAGTTGTT AACATGAATT TTGAATGTTT CTTTTTTATT GTCTCTAGGG   
  
  
+ GCATATGCAT ATTAGCAGAG TTGTTAACTT GTTAACTAGA AGCACATGTT TGTCATATCT TCTTTGTGTT   
  
  
+ AGCTTTTTTA ATGGTTAATT GTCATTCTGC TGTGCTATAT TCTATATGTG CTTTGAATGC GTGTAGCTAA   
  
  
+ AATAATCCTA AAACTGAATT ATTTGAACTT CTAGACAAAT ACAACTCGAT GCCCAGGTAA CCTCCAAAGT   
  
  
+ TTTTTAAATA ACTGAACTAT CTTCAATATA ATACCATCAT GACAGTAAGG TTAGCTTGCT GAGTTTATTA   
  
  
+ AATATGTTAA CAGTTTCCCC AGTTGTCTTG TTTGTTTCTC ACTTGTGCCA TTAGGAGGCT GGGGTTTGGA   
  
  
+ ACGTTAGAGA AGAAAGAAAA AGGGAAAAAG AGGGAGAGAA AAAGAATAGA TATATTATAG TGATGACTGA   
  
  
+ TGAGTACTTG AGCACTTGAC TACCTGATTG TTTTTATTTT TTTCAAAATG TAAACTTCTA TTTATTTATT   
  
  
+ TAGGTAAAAC TTGATAATGG CATATTATTG AACCATGCTC TGGCACTTTG CACTTTGCAG ATCCTGTGCA   
  
  
+ TTTCAGTAGT CTGTATAATT CCTACTTGTT TTAAAAAA  

- ATAAAATCAA TCAAAATACC TTCTTATAAT TTTAGTAAAA AGTAAATGTA CAGTTAGATA TATTATACCA   
  
  
- ATTTATTAAC ATATTAAATA CGATAGAGTG TGCTTTTATG AATTGGACAA AATACTCTAC TATATCATTT   
  
  
- TAAACTTTAA GTTAAACAGT TTAAATCAAA AGTAACTCAA AAAAGCGACC TTTTTATATA TGGATTAAAC   
  
  
- TTCAGGGATA TTTAATTAGA CTATTCTAAT AAAATGGTTT TGACGAATTT AAAAGTATAA TTCAACAAAG   
  
  
- ACTGAAAGAC TGCAAAGACA CCATTCTTTA GAACAGATTA CGGAACAAAA TGTTAGTGTT ATCGGATACC   
  
  
- AATAGTTGAA AATATTATCA AACTTCATTT TGAAAAAAAA ACAAACAAAA ATCTTGAAAC TACTGGTCTA   
  
  
- CCAAACTGAA AAACAACGTA TAACTAATTT AAAGTGTACA TTTACGACTG ATTGTAAATT TTAATTCCAA   
  
  
- ACCGTATACT ACTACGGGTA AAGACTACCG TCAAACCATA TATTAAAAAA AGTTAAGTCA TTTTCGTTTA   
  
  
- TGGGGTAAAA TACGATAAAG TGTACTGGAT TGGTTAAAAG ACTTTACTAT ATGATTTTAG ACTGTAAATT   
  
  
- AAATAAACTT GTTTCAAACA ATAATAAAGG TAAGAATAAT ATTATATTTC ATAATGTGAA TAATGATAAT   
  
  
- AATGATGATG TTATTAATAA TAATGATATC AATCATAATA ATGATAATAA TTATGATGAT GGTGATAACA   
  
  
- ATTAAACAAT AATAATGATA ATAATAATAA TAATAATAAT AATAATAATA ATAAAAATGA TGAGAATAAT   
  
  
- GATAATAATA TAATGATGAT GTTATTAATA ATAATGATAT CAATCATAAT AAGAAAAATG ATAATAATAA   
  
  
- TTAATAATTA TAATAATCAA AATCATAAAA AAATTTTAAT AATAATCTCA TTGTGATCAT GATATTTTAA   
  
  
- CTGTAAGAAA AGAAGGGGGG AGAGTTAGAG AGTAAGAGAG AAAGAGAGTC GGGAGAGGCG ACGAGAGTGG   
  
  
- GGAGTGTGAG AGGAAGCTCC GAATAAGTCC TTTAGGGTAT AGAGGAAACT CCGAATAAGA ACCTAAACAA   
  
  
- CAACTTGTCA AGAGATATAT ACACACACAC ACATATATAT ATATATATAT ATATATATAT ATAAAAAAAA   
  
  
- AAAAAAAAAA AAAAAAAAAA CACAAATCTT TAATCTATAA GTCTATAACT TTTCAAATGC AGTTCTAAAA   
  
  
- AGAAAAGGTC TTTTAATTTA TAAAATGGAC CTTAGAAAAC TCAAGGGTAC AGATCAATTG TTAACACACA   
  
  
- TAAAATAAAG GACTTGTTAA AAGTCAACAA TTGTACTTAA AACTTACAAA GAAAAAATAA CAGAGATCCC   
  
  
- CGTATACGTA TAATCGTCTC AACAATTGAA CAATTGATCT TCGTGTACAA ACAGTATAGA AGAAACACAA   
  
  
- TCGAAAAAAT TACCAATTAA CAGTAAGACG ACACGATATA AGATATACAC GAAACTTACG CACATCGATT   
  
  
- TTATTAGGAT TTTGACTTAA TAAACTTGAA GATCTGTTTA TGTTGAGCTA CGGGTCCATT GGAGGTTTCA   
  
  
- AAAAATTTAT TGACTTGATA GAAGTTATAT TATGGTAGTA CTGTCATTCC AATCGAACGA CTCAAATAAT   
  
  
- TTATACAATT GTCAAAGGGG TCAACAGAAC AAACAAAGAG TGAACACGGT AATCCTCCGA CCCCAAACCT   
  
  
- TGCAATCTCT TCTTTCTTTT TCCCTTTTTC TCCCTCTCTT TTTCTTATCT ATATAATATC ACTACTGACT   
  
  
- ACTCATGAAC TCGTGAACTG ATGGACTAAC AAAAATAAAA AAAGTTTTAC ATTTGAAGAT AAATAAATAA   
  
  
- ATCCATTTTG AACTATTACC GTATAATAAC TTGGTACGAG ACCGTGAAAC GTGAAACGTC TAGGACACGT   
  
  
- AAAGTCATCA GACATATTAA GGATGAACAA AATTTTTT

+     GT1-motif

| Site Name | Organism | Position | Strand | Matrix score. | sequence | function |
| --- | --- | --- | --- | --- | --- | --- |
| GT1-motif | Arabidopsis thaliana | 68 | + | 6 | GGTTAA | light responsive element |
| GT1-motif | Avena sativa | 1483 | + | 7 | GGTTAAT | light responsive element |
| GT1-motif | Arabidopsis thaliana | 111 | - | 6 | GGTTAA | light responsive element |

>PlantCARE\_20562   
+ TATTTTAGTT AGTTTTATGG AAGAATATTA AAATCATTTT TCATTTACAT GTCAATCTAT ATAATATGGT   
  
  
+ TAAATAATTG TATAATTTAT GCTATCTCAC ACGAAAATAC TTAACCTGTT TTATGAGATG ATATAGTAAA   
  
  
+ ATTTGAAATT CAATTTGTCA AATTTAGTTT TCATTGAGTT TTTTCGCTGG AAAAATATAT ACCTAATTTG   
  
  
+ AAGTCCCTAT AAATTAATCT GATAAGATTA TTTTACCAAA ACTGCTTAAA TTTTCATATT AAGTTGTTTC   
  
  
+ TGACTTTCTG ACGTTTCTGT GGTAAGAAAT CTTGTCTAAT GCCTTGTTTT ACAATCACAA TAGCCTATGG   
  
  
+ TTATCAACTT TTATAATAGT TTGAAGTAAA ACTTTTTTTT TGTTTGTTTT TAGAACTTTG ATGACCAGAT   
  
  
+ GGTTTGACTT TTTGTTGCAT ATTGATTAAA TTTCACATGT AAATGCTGAC TAACATTTAA AATTAAGGTT   
  
  
+ TGGCATATGA TGATGCCCAT TTCTGATGGC AGTTTGGTAT ATAATTTTTT TCAATTCAGT AAAAGCAAAT   
  
  
+ ACCCCATTTT ATGCTATTTC ACATGACCTA ACCAATTTTC TGAAATGATA TACTAAAATC TGACATTTAA   
  
  
+ TTTATTTGAA CAAAGTTTGT TATTATTTCC ATTCTTATTA TAATATAAAG TATTACACTT ATTACTATTA   
  
  
+ TTACTACTAC AATAATTATT ATTACTATAG TTAGTATTAT TACTATTATT AATACTACTA CCACTATTGT   
  
  
+ TAATTTGTTA TTATTACTAT TATTATTATT ATTATTATTA TTATTATTAT TATTTTTACT ACTCTTATTA   
  
  
+ CTATTATTAT ATTACTACTA CAATAATTAT TATTACTATA GTTAGTATTA TTCTTTTTAC TATTATTATT   
  
  
+ AATTATTAAT ATTATTAGTT TTAGTATTTT TTTAAAATTA TTATTAGAGT AACACTAGTA CTATAAAATT   
  
  
+ GACATTCTTT TCTTCCCCCC TCTCAATCTC TCATTCTCTC TTTCTCTCAG CCCTCTCCGC TGCTCTCACC   
  
  
+ CCTCACACTC TCCTTCGAGG CTTATTCAGG AAATCCCATA TCTCCTTTGA GGCTTATTCT TGGATTTGTT   
  
  
+ GTTGAACAGT TCTCTATATA TGTGTGTGTG TGTATATATA TATATATATA TATATATATA TATTTTTTTT   
  
  
+ TTTTTTTTTT TTTTTTTTTT GTGTTTAGAA ATTAGATATT CAGATATTGA AAAGTTTACG TCAAGATTTT   
  
  
+ TCTTTTCCAG AAAATTAAAT ATTTTACCTG GAATCTTTTG AGTTCCCATG TCTAGTTAAC AATTGTGTGT   
  
  
+ ATTTTATTTC CTGAACAATT TTCAGTTGTT AACATGAATT TTGAATGTTT CTTTTTTATT GTCTCTAGGG   
  
  
+ GCATATGCAT ATTAGCAGAG TTGTTAACTT GTTAACTAGA AGCACATGTT TGTCATATCT TCTTTGTGTT   
  
  
+ AGCTTTTTTA ATGGTTAATT GTCATTCTGC TGTGCTATAT TCTATATGTG CTTTGAATGC GTGTAGCTAA   
  
  
+ AATAATCCTA AAACTGAATT ATTTGAACTT CTAGACAAAT ACAACTCGAT GCCCAGGTAA CCTCCAAAGT   
  
  
+ TTTTTAAATA ACTGAACTAT CTTCAATATA ATACCATCAT GACAGTAAGG TTAGCTTGCT GAGTTTATTA   
  
  
+ AATATGTTAA CAGTTTCCCC AGTTGTCTTG TTTGTTTCTC ACTTGTGCCA TTAGGAGGCT GGGGTTTGGA   
  
  
+ ACGTTAGAGA AGAAAGAAAA AGGGAAAAAG AGGGAGAGAA AAAGAATAGA TATATTATAG TGATGACTGA   
  
  
+ TGAGTACTTG AGCACTTGAC TACCTGATTG TTTTTATTTT TTTCAAAATG TAAACTTCTA TTTATTTATT   
  
  
+ TAGGTAAAAC TTGATAATGG CATATTATTG AACCATGCTC TGGCACTTTG CACTTTGCAG ATCCTGTGCA   
  
  
+ TTTCAGTAGT CTGTATAATT CCTACTTGTT TTAAAAAA  

- ATAAAATCAA TCAAAATACC TTCTTATAAT TTTAGTAAAA AGTAAATGTA CAGTTAGATA TATTATACCA   
  
  
- ATTTATTAAC ATATTAAATA CGATAGAGTG TGCTTTTATG AATTGGACAA AATACTCTAC TATATCATTT   
  
  
- TAAACTTTAA GTTAAACAGT TTAAATCAAA AGTAACTCAA AAAAGCGACC TTTTTATATA TGGATTAAAC   
  
  
- TTCAGGGATA TTTAATTAGA CTATTCTAAT AAAATGGTTT TGACGAATTT AAAAGTATAA TTCAACAAAG   
  
  
- ACTGAAAGAC TGCAAAGACA CCATTCTTTA GAACAGATTA CGGAACAAAA TGTTAGTGTT ATCGGATACC   
  
  
- AATAGTTGAA AATATTATCA AACTTCATTT TGAAAAAAAA ACAAACAAAA ATCTTGAAAC TACTGGTCTA   
  
  
- CCAAACTGAA AAACAACGTA TAACTAATTT AAAGTGTACA TTTACGACTG ATTGTAAATT TTAATTCCAA   
  
  
- ACCGTATACT ACTACGGGTA AAGACTACCG TCAAACCATA TATTAAAAAA AGTTAAGTCA TTTTCGTTTA   
  
  
- TGGGGTAAAA TACGATAAAG TGTACTGGAT TGGTTAAAAG ACTTTACTAT ATGATTTTAG ACTGTAAATT   
  
  
- AAATAAACTT GTTTCAAACA ATAATAAAGG TAAGAATAAT ATTATATTTC ATAATGTGAA TAATGATAAT   
  
  
- AATGATGATG TTATTAATAA TAATGATATC AATCATAATA ATGATAATAA TTATGATGAT GGTGATAACA   
  
  
- ATTAAACAAT AATAATGATA ATAATAATAA TAATAATAAT AATAATAATA ATAAAAATGA TGAGAATAAT   
  
  
- GATAATAATA TAATGATGAT GTTATTAATA ATAATGATAT CAATCATAAT AAGAAAAATG ATAATAATAA   
  
  
- TTAATAATTA TAATAATCAA AATCATAAAA AAATTTTAAT AATAATCTCA TTGTGATCAT GATATTTTAA   
  
  
- CTGTAAGAAA AGAAGGGGGG AGAGTTAGAG AGTAAGAGAG AAAGAGAGTC GGGAGAGGCG ACGAGAGTGG   
  
  
- GGAGTGTGAG AGGAAGCTCC GAATAAGTCC TTTAGGGTAT AGAGGAAACT CCGAATAAGA ACCTAAACAA   
  
  
- CAACTTGTCA AGAGATATAT ACACACACAC ACATATATAT ATATATATAT ATATATATAT ATAAAAAAAA   
  
  
- AAAAAAAAAA AAAAAAAAAA CACAAATCTT TAATCTATAA GTCTATAACT TTTCAAATGC AGTTCTAAAA   
  
  
- AGAAAAGGTC TTTTAATTTA TAAAATGGAC CTTAGAAAAC TCAAGGGTAC AGATCAATTG TTAACACACA   
  
  
- TAAAATAAAG GACTTGTTAA AAGTCAACAA TTGTACTTAA AACTTACAAA GAAAAAATAA CAGAGATCCC   
  
  
- CGTATACGTA TAATCGTCTC AACAATTGAA CAATTGATCT TCGTGTACAA ACAGTATAGA AGAAACACAA   
  
  
- TCGAAAAAAT TACCAATTAA CAGTAAGACG ACACGATATA AGATATACAC GAAACTTACG CACATCGATT   
  
  
- TTATTAGGAT TTTGACTTAA TAAACTTGAA GATCTGTTTA TGTTGAGCTA CGGGTCCATT GGAGGTTTCA   
  
  
- AAAAATTTAT TGACTTGATA GAAGTTATAT TATGGTAGTA CTGTCATTCC AATCGAACGA CTCAAATAAT   
  
  
- TTATACAATT GTCAAAGGGG TCAACAGAAC AAACAAAGAG TGAACACGGT AATCCTCCGA CCCCAAACCT   
  
  
- TGCAATCTCT TCTTTCTTTT TCCCTTTTTC TCCCTCTCTT TTTCTTATCT ATATAATATC ACTACTGACT   
  
  
- ACTCATGAAC TCGTGAACTG ATGGACTAAC AAAAATAAAA AAAGTTTTAC ATTTGAAGAT AAATAAATAA   
  
  
- ATCCATTTTG AACTATTACC GTATAATAAC TTGGTACGAG ACCGTGAAAC GTGAAACGTC TAGGACACGT   
  
  
- AAAGTCATCA GACATATTAA GGATGAACAA AATTTTTT

+     MBS

| Site Name | Organism | Position | Strand | Matrix score. | sequence | function |
| --- | --- | --- | --- | --- | --- | --- |
| MBS | Arabidopsis thaliana | 1353 | - | 6 | CAACTG | MYB binding site involved in drought-inducibility |
| MBS | Arabidopsis thaliana | 1700 | - | 6 | CAACTG | MYB binding site involved in drought-inducibility |

>PlantCARE\_20562   
+ TATTTTAGTT AGTTTTATGG AAGAATATTA AAATCATTTT TCATTTACAT GTCAATCTAT ATAATATGGT   
  
  
+ TAAATAATTG TATAATTTAT GCTATCTCAC ACGAAAATAC TTAACCTGTT TTATGAGATG ATATAGTAAA   
  
  
+ ATTTGAAATT CAATTTGTCA AATTTAGTTT TCATTGAGTT TTTTCGCTGG AAAAATATAT ACCTAATTTG   
  
  
+ AAGTCCCTAT AAATTAATCT GATAAGATTA TTTTACCAAA ACTGCTTAAA TTTTCATATT AAGTTGTTTC   
  
  
+ TGACTTTCTG ACGTTTCTGT GGTAAGAAAT CTTGTCTAAT GCCTTGTTTT ACAATCACAA TAGCCTATGG   
  
  
+ TTATCAACTT TTATAATAGT TTGAAGTAAA ACTTTTTTTT TGTTTGTTTT TAGAACTTTG ATGACCAGAT   
  
  
+ GGTTTGACTT TTTGTTGCAT ATTGATTAAA TTTCACATGT AAATGCTGAC TAACATTTAA AATTAAGGTT   
  
  
+ TGGCATATGA TGATGCCCAT TTCTGATGGC AGTTTGGTAT ATAATTTTTT TCAATTCAGT AAAAGCAAAT   
  
  
+ ACCCCATTTT ATGCTATTTC ACATGACCTA ACCAATTTTC TGAAATGATA TACTAAAATC TGACATTTAA   
  
  
+ TTTATTTGAA CAAAGTTTGT TATTATTTCC ATTCTTATTA TAATATAAAG TATTACACTT ATTACTATTA   
  
  
+ TTACTACTAC AATAATTATT ATTACTATAG TTAGTATTAT TACTATTATT AATACTACTA CCACTATTGT   
  
  
+ TAATTTGTTA TTATTACTAT TATTATTATT ATTATTATTA TTATTATTAT TATTTTTACT ACTCTTATTA   
  
  
+ CTATTATTAT ATTACTACTA CAATAATTAT TATTACTATA GTTAGTATTA TTCTTTTTAC TATTATTATT   
  
  
+ AATTATTAAT ATTATTAGTT TTAGTATTTT TTTAAAATTA TTATTAGAGT AACACTAGTA CTATAAAATT   
  
  
+ GACATTCTTT TCTTCCCCCC TCTCAATCTC TCATTCTCTC TTTCTCTCAG CCCTCTCCGC TGCTCTCACC   
  
  
+ CCTCACACTC TCCTTCGAGG CTTATTCAGG AAATCCCATA TCTCCTTTGA GGCTTATTCT TGGATTTGTT   
  
  
+ GTTGAACAGT TCTCTATATA TGTGTGTGTG TGTATATATA TATATATATA TATATATATA TATTTTTTTT   
  
  
+ TTTTTTTTTT TTTTTTTTTT GTGTTTAGAA ATTAGATATT CAGATATTGA AAAGTTTACG TCAAGATTTT   
  
  
+ TCTTTTCCAG AAAATTAAAT ATTTTACCTG GAATCTTTTG AGTTCCCATG TCTAGTTAAC AATTGTGTGT   
  
  
+ ATTTTATTTC CTGAACAATT TTCAGTTGTT AACATGAATT TTGAATGTTT CTTTTTTATT GTCTCTAGGG   
  
  
+ GCATATGCAT ATTAGCAGAG TTGTTAACTT GTTAACTAGA AGCACATGTT TGTCATATCT TCTTTGTGTT   
  
  
+ AGCTTTTTTA ATGGTTAATT GTCATTCTGC TGTGCTATAT TCTATATGTG CTTTGAATGC GTGTAGCTAA   
  
  
+ AATAATCCTA AAACTGAATT ATTTGAACTT CTAGACAAAT ACAACTCGAT GCCCAGGTAA CCTCCAAAGT   
  
  
+ TTTTTAAATA ACTGAACTAT CTTCAATATA ATACCATCAT GACAGTAAGG TTAGCTTGCT GAGTTTATTA   
  
  
+ AATATGTTAA CAGTTTCCCC AGTTGTCTTG TTTGTTTCTC ACTTGTGCCA TTAGGAGGCT GGGGTTTGGA   
  
  
+ ACGTTAGAGA AGAAAGAAAA AGGGAAAAAG AGGGAGAGAA AAAGAATAGA TATATTATAG TGATGACTGA   
  
  
+ TGAGTACTTG AGCACTTGAC TACCTGATTG TTTTTATTTT TTTCAAAATG TAAACTTCTA TTTATTTATT   
  
  
+ TAGGTAAAAC TTGATAATGG CATATTATTG AACCATGCTC TGGCACTTTG CACTTTGCAG ATCCTGTGCA   
  
  
+ TTTCAGTAGT CTGTATAATT CCTACTTGTT TTAAAAAA  

- ATAAAATCAA TCAAAATACC TTCTTATAAT TTTAGTAAAA AGTAAATGTA CAGTTAGATA TATTATACCA   
  
  
- ATTTATTAAC ATATTAAATA CGATAGAGTG TGCTTTTATG AATTGGACAA AATACTCTAC TATATCATTT   
  
  
- TAAACTTTAA GTTAAACAGT TTAAATCAAA AGTAACTCAA AAAAGCGACC TTTTTATATA TGGATTAAAC   
  
  
- TTCAGGGATA TTTAATTAGA CTATTCTAAT AAAATGGTTT TGACGAATTT AAAAGTATAA TTCAACAAAG   
  
  
- ACTGAAAGAC TGCAAAGACA CCATTCTTTA GAACAGATTA CGGAACAAAA TGTTAGTGTT ATCGGATACC   
  
  
- AATAGTTGAA AATATTATCA AACTTCATTT TGAAAAAAAA ACAAACAAAA ATCTTGAAAC TACTGGTCTA   
  
  
- CCAAACTGAA AAACAACGTA TAACTAATTT AAAGTGTACA TTTACGACTG ATTGTAAATT TTAATTCCAA   
  
  
- ACCGTATACT ACTACGGGTA AAGACTACCG TCAAACCATA TATTAAAAAA AGTTAAGTCA TTTTCGTTTA   
  
  
- TGGGGTAAAA TACGATAAAG TGTACTGGAT TGGTTAAAAG ACTTTACTAT ATGATTTTAG ACTGTAAATT   
  
  
- AAATAAACTT GTTTCAAACA ATAATAAAGG TAAGAATAAT ATTATATTTC ATAATGTGAA TAATGATAAT   
  
  
- AATGATGATG TTATTAATAA TAATGATATC AATCATAATA ATGATAATAA TTATGATGAT GGTGATAACA   
  
  
- ATTAAACAAT AATAATGATA ATAATAATAA TAATAATAAT AATAATAATA ATAAAAATGA TGAGAATAAT   
  
  
- GATAATAATA TAATGATGAT GTTATTAATA ATAATGATAT CAATCATAAT AAGAAAAATG ATAATAATAA   
  
  
- TTAATAATTA TAATAATCAA AATCATAAAA AAATTTTAAT AATAATCTCA TTGTGATCAT GATATTTTAA   
  
  
- CTGTAAGAAA AGAAGGGGGG AGAGTTAGAG AGTAAGAGAG AAAGAGAGTC GGGAGAGGCG ACGAGAGTGG   
  
  
- GGAGTGTGAG AGGAAGCTCC GAATAAGTCC TTTAGGGTAT AGAGGAAACT CCGAATAAGA ACCTAAACAA   
  
  
- CAACTTGTCA AGAGATATAT ACACACACAC ACATATATAT ATATATATAT ATATATATAT ATAAAAAAAA   
  
  
- AAAAAAAAAA AAAAAAAAAA CACAAATCTT TAATCTATAA GTCTATAACT TTTCAAATGC AGTTCTAAAA   
  
  
- AGAAAAGGTC TTTTAATTTA TAAAATGGAC CTTAGAAAAC TCAAGGGTAC AGATCAATTG TTAACACACA   
  
  
- TAAAATAAAG GACTTGTTAA AAGTCAACAA TTGTACTTAA AACTTACAAA GAAAAAATAA CAGAGATCCC   
  
  
- CGTATACGTA TAATCGTCTC AACAATTGAA CAATTGATCT TCGTGTACAA ACAGTATAGA AGAAACACAA   
  
  
- TCGAAAAAAT TACCAATTAA CAGTAAGACG ACACGATATA AGATATACAC GAAACTTACG CACATCGATT   
  
  
- TTATTAGGAT TTTGACTTAA TAAACTTGAA GATCTGTTTA TGTTGAGCTA CGGGTCCATT GGAGGTTTCA   
  
  
- AAAAATTTAT TGACTTGATA GAAGTTATAT TATGGTAGTA CTGTCATTCC AATCGAACGA CTCAAATAAT   
  
  
- TTATACAATT GTCAAAGGGG TCAACAGAAC AAACAAAGAG TGAACACGGT AATCCTCCGA CCCCAAACCT   
  
  
- TGCAATCTCT TCTTTCTTTT TCCCTTTTTC TCCCTCTCTT TTTCTTATCT ATATAATATC ACTACTGACT   
  
  
- ACTCATGAAC TCGTGAACTG ATGGACTAAC AAAAATAAAA AAAGTTTTAC ATTTGAAGAT AAATAAATAA   
  
  
- ATCCATTTTG AACTATTACC GTATAATAAC TTGGTACGAG ACCGTGAAAC GTGAAACGTC TAGGACACGT   
  
  
- AAAGTCATCA GACATATTAA GGATGAACAA AATTTTTT

+     MYB

| Site Name | Organism | Position | Strand | Matrix score. | sequence | function |
| --- | --- | --- | --- | --- | --- | --- |
| MYB | Arabidopsis thaliana | 67 | - | 6 | TAACCA |  |
| MYB | Arabidopsis thaliana | 589 | + | 6 | TAACCA |  |
| MYB | Arabidopsis thaliana | 348 | - | 6 | TAACCA |  |
| MYB | Arabidopsis thaliana | 1482 | - | 6 | TAACCA |  |

>PlantCARE\_20562   
+ TATTTTAGTT AGTTTTATGG AAGAATATTA AAATCATTTT TCATTTACAT GTCAATCTAT ATAATATGGT   
  
  
+ TAAATAATTG TATAATTTAT GCTATCTCAC ACGAAAATAC TTAACCTGTT TTATGAGATG ATATAGTAAA   
  
  
+ ATTTGAAATT CAATTTGTCA AATTTAGTTT TCATTGAGTT TTTTCGCTGG AAAAATATAT ACCTAATTTG   
  
  
+ AAGTCCCTAT AAATTAATCT GATAAGATTA TTTTACCAAA ACTGCTTAAA TTTTCATATT AAGTTGTTTC   
  
  
+ TGACTTTCTG ACGTTTCTGT GGTAAGAAAT CTTGTCTAAT GCCTTGTTTT ACAATCACAA TAGCCTATGG   
  
  
+ TTATCAACTT TTATAATAGT TTGAAGTAAA ACTTTTTTTT TGTTTGTTTT TAGAACTTTG ATGACCAGAT   
  
  
+ GGTTTGACTT TTTGTTGCAT ATTGATTAAA TTTCACATGT AAATGCTGAC TAACATTTAA AATTAAGGTT   
  
  
+ TGGCATATGA TGATGCCCAT TTCTGATGGC AGTTTGGTAT ATAATTTTTT TCAATTCAGT AAAAGCAAAT   
  
  
+ ACCCCATTTT ATGCTATTTC ACATGACCTA ACCAATTTTC TGAAATGATA TACTAAAATC TGACATTTAA   
  
  
+ TTTATTTGAA CAAAGTTTGT TATTATTTCC ATTCTTATTA TAATATAAAG TATTACACTT ATTACTATTA   
  
  
+ TTACTACTAC AATAATTATT ATTACTATAG TTAGTATTAT TACTATTATT AATACTACTA CCACTATTGT   
  
  
+ TAATTTGTTA TTATTACTAT TATTATTATT ATTATTATTA TTATTATTAT TATTTTTACT ACTCTTATTA   
  
  
+ CTATTATTAT ATTACTACTA CAATAATTAT TATTACTATA GTTAGTATTA TTCTTTTTAC TATTATTATT   
  
  
+ AATTATTAAT ATTATTAGTT TTAGTATTTT TTTAAAATTA TTATTAGAGT AACACTAGTA CTATAAAATT   
  
  
+ GACATTCTTT TCTTCCCCCC TCTCAATCTC TCATTCTCTC TTTCTCTCAG CCCTCTCCGC TGCTCTCACC   
  
  
+ CCTCACACTC TCCTTCGAGG CTTATTCAGG AAATCCCATA TCTCCTTTGA GGCTTATTCT TGGATTTGTT   
  
  
+ GTTGAACAGT TCTCTATATA TGTGTGTGTG TGTATATATA TATATATATA TATATATATA TATTTTTTTT   
  
  
+ TTTTTTTTTT TTTTTTTTTT GTGTTTAGAA ATTAGATATT CAGATATTGA AAAGTTTACG TCAAGATTTT   
  
  
+ TCTTTTCCAG AAAATTAAAT ATTTTACCTG GAATCTTTTG AGTTCCCATG TCTAGTTAAC AATTGTGTGT   
  
  
+ ATTTTATTTC CTGAACAATT TTCAGTTGTT AACATGAATT TTGAATGTTT CTTTTTTATT GTCTCTAGGG   
  
  
+ GCATATGCAT ATTAGCAGAG TTGTTAACTT GTTAACTAGA AGCACATGTT TGTCATATCT TCTTTGTGTT   
  
  
+ AGCTTTTTTA ATGGTTAATT GTCATTCTGC TGTGCTATAT TCTATATGTG CTTTGAATGC GTGTAGCTAA   
  
  
+ AATAATCCTA AAACTGAATT ATTTGAACTT CTAGACAAAT ACAACTCGAT GCCCAGGTAA CCTCCAAAGT   
  
  
+ TTTTTAAATA ACTGAACTAT CTTCAATATA ATACCATCAT GACAGTAAGG TTAGCTTGCT GAGTTTATTA   
  
  
+ AATATGTTAA CAGTTTCCCC AGTTGTCTTG TTTGTTTCTC ACTTGTGCCA TTAGGAGGCT GGGGTTTGGA   
  
  
+ ACGTTAGAGA AGAAAGAAAA AGGGAAAAAG AGGGAGAGAA AAAGAATAGA TATATTATAG TGATGACTGA   
  
  
+ TGAGTACTTG AGCACTTGAC TACCTGATTG TTTTTATTTT TTTCAAAATG TAAACTTCTA TTTATTTATT   
  
  
+ TAGGTAAAAC TTGATAATGG CATATTATTG AACCATGCTC TGGCACTTTG CACTTTGCAG ATCCTGTGCA   
  
  
+ TTTCAGTAGT CTGTATAATT CCTACTTGTT TTAAAAAA  

- ATAAAATCAA TCAAAATACC TTCTTATAAT TTTAGTAAAA AGTAAATGTA CAGTTAGATA TATTATACCA   
  
  
- ATTTATTAAC ATATTAAATA CGATAGAGTG TGCTTTTATG AATTGGACAA AATACTCTAC TATATCATTT   
  
  
- TAAACTTTAA GTTAAACAGT TTAAATCAAA AGTAACTCAA AAAAGCGACC TTTTTATATA TGGATTAAAC   
  
  
- TTCAGGGATA TTTAATTAGA CTATTCTAAT AAAATGGTTT TGACGAATTT AAAAGTATAA TTCAACAAAG   
  
  
- ACTGAAAGAC TGCAAAGACA CCATTCTTTA GAACAGATTA CGGAACAAAA TGTTAGTGTT ATCGGATACC   
  
  
- AATAGTTGAA AATATTATCA AACTTCATTT TGAAAAAAAA ACAAACAAAA ATCTTGAAAC TACTGGTCTA   
  
  
- CCAAACTGAA AAACAACGTA TAACTAATTT AAAGTGTACA TTTACGACTG ATTGTAAATT TTAATTCCAA   
  
  
- ACCGTATACT ACTACGGGTA AAGACTACCG TCAAACCATA TATTAAAAAA AGTTAAGTCA TTTTCGTTTA   
  
  
- TGGGGTAAAA TACGATAAAG TGTACTGGAT TGGTTAAAAG ACTTTACTAT ATGATTTTAG ACTGTAAATT   
  
  
- AAATAAACTT GTTTCAAACA ATAATAAAGG TAAGAATAAT ATTATATTTC ATAATGTGAA TAATGATAAT   
  
  
- AATGATGATG TTATTAATAA TAATGATATC AATCATAATA ATGATAATAA TTATGATGAT GGTGATAACA   
  
  
- ATTAAACAAT AATAATGATA ATAATAATAA TAATAATAAT AATAATAATA ATAAAAATGA TGAGAATAAT   
  
  
- GATAATAATA TAATGATGAT GTTATTAATA ATAATGATAT CAATCATAAT AAGAAAAATG ATAATAATAA   
  
  
- TTAATAATTA TAATAATCAA AATCATAAAA AAATTTTAAT AATAATCTCA TTGTGATCAT GATATTTTAA   
  
  
- CTGTAAGAAA AGAAGGGGGG AGAGTTAGAG AGTAAGAGAG AAAGAGAGTC GGGAGAGGCG ACGAGAGTGG   
  
  
- GGAGTGTGAG AGGAAGCTCC GAATAAGTCC TTTAGGGTAT AGAGGAAACT CCGAATAAGA ACCTAAACAA   
  
  
- CAACTTGTCA AGAGATATAT ACACACACAC ACATATATAT ATATATATAT ATATATATAT ATAAAAAAAA   
  
  
- AAAAAAAAAA AAAAAAAAAA CACAAATCTT TAATCTATAA GTCTATAACT TTTCAAATGC AGTTCTAAAA   
  
  
- AGAAAAGGTC TTTTAATTTA TAAAATGGAC CTTAGAAAAC TCAAGGGTAC AGATCAATTG TTAACACACA   
  
  
- TAAAATAAAG GACTTGTTAA AAGTCAACAA TTGTACTTAA AACTTACAAA GAAAAAATAA CAGAGATCCC   
  
  
- CGTATACGTA TAATCGTCTC AACAATTGAA CAATTGATCT TCGTGTACAA ACAGTATAGA AGAAACACAA   
  
  
- TCGAAAAAAT TACCAATTAA CAGTAAGACG ACACGATATA AGATATACAC GAAACTTACG CACATCGATT   
  
  
- TTATTAGGAT TTTGACTTAA TAAACTTGAA GATCTGTTTA TGTTGAGCTA CGGGTCCATT GGAGGTTTCA   
  
  
- AAAAATTTAT TGACTTGATA GAAGTTATAT TATGGTAGTA CTGTCATTCC AATCGAACGA CTCAAATAAT   
  
  
- TTATACAATT GTCAAAGGGG TCAACAGAAC AAACAAAGAG TGAACACGGT AATCCTCCGA CCCCAAACCT   
  
  
- TGCAATCTCT TCTTTCTTTT TCCCTTTTTC TCCCTCTCTT TTTCTTATCT ATATAATATC ACTACTGACT   
  
  
- ACTCATGAAC TCGTGAACTG ATGGACTAAC AAAAATAAAA AAAGTTTTAC ATTTGAAGAT AAATAAATAA   
  
  
- ATCCATTTTG AACTATTACC GTATAATAAC TTGGTACGAG ACCGTGAAAC GTGAAACGTC TAGGACACGT   
  
  
- AAAGTCATCA GACATATTAA GGATGAACAA AATTTTTT

+     MYB-like sequence

| Site Name | Organism | Position | Strand | Matrix score. | sequence | function |
| --- | --- | --- | --- | --- | --- | --- |
| MYB-like sequence | Arabidopsis thaliana | 67 | - | 6 | TAACCA |  |
| MYB-like sequence | Arabidopsis thaliana | 589 | + | 6 | TAACCA |  |
| MYB-like sequence | Arabidopsis thaliana | 348 | - | 6 | TAACCA |  |
| MYB-like sequence | Arabidopsis thaliana | 1482 | - | 6 | TAACCA |  |

>PlantCARE\_20562   
+ TATTTTAGTT AGTTTTATGG AAGAATATTA AAATCATTTT TCATTTACAT GTCAATCTAT ATAATATGGT   
  
  
+ TAAATAATTG TATAATTTAT GCTATCTCAC ACGAAAATAC TTAACCTGTT TTATGAGATG ATATAGTAAA   
  
  
+ ATTTGAAATT CAATTTGTCA AATTTAGTTT TCATTGAGTT TTTTCGCTGG AAAAATATAT ACCTAATTTG   
  
  
+ AAGTCCCTAT AAATTAATCT GATAAGATTA TTTTACCAAA ACTGCTTAAA TTTTCATATT AAGTTGTTTC   
  
  
+ TGACTTTCTG ACGTTTCTGT GGTAAGAAAT CTTGTCTAAT GCCTTGTTTT ACAATCACAA TAGCCTATGG   
  
  
+ TTATCAACTT TTATAATAGT TTGAAGTAAA ACTTTTTTTT TGTTTGTTTT TAGAACTTTG ATGACCAGAT   
  
  
+ GGTTTGACTT TTTGTTGCAT ATTGATTAAA TTTCACATGT AAATGCTGAC TAACATTTAA AATTAAGGTT   
  
  
+ TGGCATATGA TGATGCCCAT TTCTGATGGC AGTTTGGTAT ATAATTTTTT TCAATTCAGT AAAAGCAAAT   
  
  
+ ACCCCATTTT ATGCTATTTC ACATGACCTA ACCAATTTTC TGAAATGATA TACTAAAATC TGACATTTAA   
  
  
+ TTTATTTGAA CAAAGTTTGT TATTATTTCC ATTCTTATTA TAATATAAAG TATTACACTT ATTACTATTA   
  
  
+ TTACTACTAC AATAATTATT ATTACTATAG TTAGTATTAT TACTATTATT AATACTACTA CCACTATTGT   
  
  
+ TAATTTGTTA TTATTACTAT TATTATTATT ATTATTATTA TTATTATTAT TATTTTTACT ACTCTTATTA   
  
  
+ CTATTATTAT ATTACTACTA CAATAATTAT TATTACTATA GTTAGTATTA TTCTTTTTAC TATTATTATT   
  
  
+ AATTATTAAT ATTATTAGTT TTAGTATTTT TTTAAAATTA TTATTAGAGT AACACTAGTA CTATAAAATT   
  
  
+ GACATTCTTT TCTTCCCCCC TCTCAATCTC TCATTCTCTC TTTCTCTCAG CCCTCTCCGC TGCTCTCACC   
  
  
+ CCTCACACTC TCCTTCGAGG CTTATTCAGG AAATCCCATA TCTCCTTTGA GGCTTATTCT TGGATTTGTT   
  
  
+ GTTGAACAGT TCTCTATATA TGTGTGTGTG TGTATATATA TATATATATA TATATATATA TATTTTTTTT   
  
  
+ TTTTTTTTTT TTTTTTTTTT GTGTTTAGAA ATTAGATATT CAGATATTGA AAAGTTTACG TCAAGATTTT   
  
  
+ TCTTTTCCAG AAAATTAAAT ATTTTACCTG GAATCTTTTG AGTTCCCATG TCTAGTTAAC AATTGTGTGT   
  
  
+ ATTTTATTTC CTGAACAATT TTCAGTTGTT AACATGAATT TTGAATGTTT CTTTTTTATT GTCTCTAGGG   
  
  
+ GCATATGCAT ATTAGCAGAG TTGTTAACTT GTTAACTAGA AGCACATGTT TGTCATATCT TCTTTGTGTT   
  
  
+ AGCTTTTTTA ATGGTTAATT GTCATTCTGC TGTGCTATAT TCTATATGTG CTTTGAATGC GTGTAGCTAA   
  
  
+ AATAATCCTA AAACTGAATT ATTTGAACTT CTAGACAAAT ACAACTCGAT GCCCAGGTAA CCTCCAAAGT   
  
  
+ TTTTTAAATA ACTGAACTAT CTTCAATATA ATACCATCAT GACAGTAAGG TTAGCTTGCT GAGTTTATTA   
  
  
+ AATATGTTAA CAGTTTCCCC AGTTGTCTTG TTTGTTTCTC ACTTGTGCCA TTAGGAGGCT GGGGTTTGGA   
  
  
+ ACGTTAGAGA AGAAAGAAAA AGGGAAAAAG AGGGAGAGAA AAAGAATAGA TATATTATAG TGATGACTGA   
  
  
+ TGAGTACTTG AGCACTTGAC TACCTGATTG TTTTTATTTT TTTCAAAATG TAAACTTCTA TTTATTTATT   
  
  
+ TAGGTAAAAC TTGATAATGG CATATTATTG AACCATGCTC TGGCACTTTG CACTTTGCAG ATCCTGTGCA   
  
  
+ TTTCAGTAGT CTGTATAATT CCTACTTGTT TTAAAAAA  

- ATAAAATCAA TCAAAATACC TTCTTATAAT TTTAGTAAAA AGTAAATGTA CAGTTAGATA TATTATACCA   
  
  
- ATTTATTAAC ATATTAAATA CGATAGAGTG TGCTTTTATG AATTGGACAA AATACTCTAC TATATCATTT   
  
  
- TAAACTTTAA GTTAAACAGT TTAAATCAAA AGTAACTCAA AAAAGCGACC TTTTTATATA TGGATTAAAC   
  
  
- TTCAGGGATA TTTAATTAGA CTATTCTAAT AAAATGGTTT TGACGAATTT AAAAGTATAA TTCAACAAAG   
  
  
- ACTGAAAGAC TGCAAAGACA CCATTCTTTA GAACAGATTA CGGAACAAAA TGTTAGTGTT ATCGGATACC   
  
  
- AATAGTTGAA AATATTATCA AACTTCATTT TGAAAAAAAA ACAAACAAAA ATCTTGAAAC TACTGGTCTA   
  
  
- CCAAACTGAA AAACAACGTA TAACTAATTT AAAGTGTACA TTTACGACTG ATTGTAAATT TTAATTCCAA   
  
  
- ACCGTATACT ACTACGGGTA AAGACTACCG TCAAACCATA TATTAAAAAA AGTTAAGTCA TTTTCGTTTA   
  
  
- TGGGGTAAAA TACGATAAAG TGTACTGGAT TGGTTAAAAG ACTTTACTAT ATGATTTTAG ACTGTAAATT   
  
  
- AAATAAACTT GTTTCAAACA ATAATAAAGG TAAGAATAAT ATTATATTTC ATAATGTGAA TAATGATAAT   
  
  
- AATGATGATG TTATTAATAA TAATGATATC AATCATAATA ATGATAATAA TTATGATGAT GGTGATAACA   
  
  
- ATTAAACAAT AATAATGATA ATAATAATAA TAATAATAAT AATAATAATA ATAAAAATGA TGAGAATAAT   
  
  
- GATAATAATA TAATGATGAT GTTATTAATA ATAATGATAT CAATCATAAT AAGAAAAATG ATAATAATAA   
  
  
- TTAATAATTA TAATAATCAA AATCATAAAA AAATTTTAAT AATAATCTCA TTGTGATCAT GATATTTTAA   
  
  
- CTGTAAGAAA AGAAGGGGGG AGAGTTAGAG AGTAAGAGAG AAAGAGAGTC GGGAGAGGCG ACGAGAGTGG   
  
  
- GGAGTGTGAG AGGAAGCTCC GAATAAGTCC TTTAGGGTAT AGAGGAAACT CCGAATAAGA ACCTAAACAA   
  
  
- CAACTTGTCA AGAGATATAT ACACACACAC ACATATATAT ATATATATAT ATATATATAT ATAAAAAAAA   
  
  
- AAAAAAAAAA AAAAAAAAAA CACAAATCTT TAATCTATAA GTCTATAACT TTTCAAATGC AGTTCTAAAA   
  
  
- AGAAAAGGTC TTTTAATTTA TAAAATGGAC CTTAGAAAAC TCAAGGGTAC AGATCAATTG TTAACACACA   
  
  
- TAAAATAAAG GACTTGTTAA AAGTCAACAA TTGTACTTAA AACTTACAAA GAAAAAATAA CAGAGATCCC   
  
  
- CGTATACGTA TAATCGTCTC AACAATTGAA CAATTGATCT TCGTGTACAA ACAGTATAGA AGAAACACAA   
  
  
- TCGAAAAAAT TACCAATTAA CAGTAAGACG ACACGATATA AGATATACAC GAAACTTACG CACATCGATT   
  
  
- TTATTAGGAT TTTGACTTAA TAAACTTGAA GATCTGTTTA TGTTGAGCTA CGGGTCCATT GGAGGTTTCA   
  
  
- AAAAATTTAT TGACTTGATA GAAGTTATAT TATGGTAGTA CTGTCATTCC AATCGAACGA CTCAAATAAT   
  
  
- TTATACAATT GTCAAAGGGG TCAACAGAAC AAACAAAGAG TGAACACGGT AATCCTCCGA CCCCAAACCT   
  
  
- TGCAATCTCT TCTTTCTTTT TCCCTTTTTC TCCCTCTCTT TTTCTTATCT ATATAATATC ACTACTGACT   
  
  
- ACTCATGAAC TCGTGAACTG ATGGACTAAC AAAAATAAAA AAAGTTTTAC ATTTGAAGAT AAATAAATAA   
  
  
- ATCCATTTTG AACTATTACC GTATAATAAC TTGGTACGAG ACCGTGAAAC GTGAAACGTC TAGGACACGT   
  
  
- AAAGTCATCA GACATATTAA GGATGAACAA AATTTTTT

+     MYC

| Site Name | Organism | Position | Strand | Matrix score. | sequence | function |
| --- | --- | --- | --- | --- | --- | --- |
| MYC | Arabidopsis thaliana | 454 | - | 6 | CATGTG |  |
| MYC | Arabidopsis thaliana | 1320 | - | 6 | CAATTG |  |
| MYC | Arabidopsis thaliana | 580 | - | 6 | CATGTG |  |
| MYC | Arabidopsis thaliana | 1443 | - | 6 | CATGTG |  |

>PlantCARE\_20562   
+ TATTTTAGTT AGTTTTATGG AAGAATATTA AAATCATTTT TCATTTACAT GTCAATCTAT ATAATATGGT   
  
  
+ TAAATAATTG TATAATTTAT GCTATCTCAC ACGAAAATAC TTAACCTGTT TTATGAGATG ATATAGTAAA   
  
  
+ ATTTGAAATT CAATTTGTCA AATTTAGTTT TCATTGAGTT TTTTCGCTGG AAAAATATAT ACCTAATTTG   
  
  
+ AAGTCCCTAT AAATTAATCT GATAAGATTA TTTTACCAAA ACTGCTTAAA TTTTCATATT AAGTTGTTTC   
  
  
+ TGACTTTCTG ACGTTTCTGT GGTAAGAAAT CTTGTCTAAT GCCTTGTTTT ACAATCACAA TAGCCTATGG   
  
  
+ TTATCAACTT TTATAATAGT TTGAAGTAAA ACTTTTTTTT TGTTTGTTTT TAGAACTTTG ATGACCAGAT   
  
  
+ GGTTTGACTT TTTGTTGCAT ATTGATTAAA TTTCACATGT AAATGCTGAC TAACATTTAA AATTAAGGTT   
  
  
+ TGGCATATGA TGATGCCCAT TTCTGATGGC AGTTTGGTAT ATAATTTTTT TCAATTCAGT AAAAGCAAAT   
  
  
+ ACCCCATTTT ATGCTATTTC ACATGACCTA ACCAATTTTC TGAAATGATA TACTAAAATC TGACATTTAA   
  
  
+ TTTATTTGAA CAAAGTTTGT TATTATTTCC ATTCTTATTA TAATATAAAG TATTACACTT ATTACTATTA   
  
  
+ TTACTACTAC AATAATTATT ATTACTATAG TTAGTATTAT TACTATTATT AATACTACTA CCACTATTGT   
  
  
+ TAATTTGTTA TTATTACTAT TATTATTATT ATTATTATTA TTATTATTAT TATTTTTACT ACTCTTATTA   
  
  
+ CTATTATTAT ATTACTACTA CAATAATTAT TATTACTATA GTTAGTATTA TTCTTTTTAC TATTATTATT   
  
  
+ AATTATTAAT ATTATTAGTT TTAGTATTTT TTTAAAATTA TTATTAGAGT AACACTAGTA CTATAAAATT   
  
  
+ GACATTCTTT TCTTCCCCCC TCTCAATCTC TCATTCTCTC TTTCTCTCAG CCCTCTCCGC TGCTCTCACC   
  
  
+ CCTCACACTC TCCTTCGAGG CTTATTCAGG AAATCCCATA TCTCCTTTGA GGCTTATTCT TGGATTTGTT   
  
  
+ GTTGAACAGT TCTCTATATA TGTGTGTGTG TGTATATATA TATATATATA TATATATATA TATTTTTTTT   
  
  
+ TTTTTTTTTT TTTTTTTTTT GTGTTTAGAA ATTAGATATT CAGATATTGA AAAGTTTACG TCAAGATTTT   
  
  
+ TCTTTTCCAG AAAATTAAAT ATTTTACCTG GAATCTTTTG AGTTCCCATG TCTAGTTAAC AATTGTGTGT   
  
  
+ ATTTTATTTC CTGAACAATT TTCAGTTGTT AACATGAATT TTGAATGTTT CTTTTTTATT GTCTCTAGGG   
  
  
+ GCATATGCAT ATTAGCAGAG TTGTTAACTT GTTAACTAGA AGCACATGTT TGTCATATCT TCTTTGTGTT   
  
  
+ AGCTTTTTTA ATGGTTAATT GTCATTCTGC TGTGCTATAT TCTATATGTG CTTTGAATGC GTGTAGCTAA   
  
  
+ AATAATCCTA AAACTGAATT ATTTGAACTT CTAGACAAAT ACAACTCGAT GCCCAGGTAA CCTCCAAAGT   
  
  
+ TTTTTAAATA ACTGAACTAT CTTCAATATA ATACCATCAT GACAGTAAGG TTAGCTTGCT GAGTTTATTA   
  
  
+ AATATGTTAA CAGTTTCCCC AGTTGTCTTG TTTGTTTCTC ACTTGTGCCA TTAGGAGGCT GGGGTTTGGA   
  
  
+ ACGTTAGAGA AGAAAGAAAA AGGGAAAAAG AGGGAGAGAA AAAGAATAGA TATATTATAG TGATGACTGA   
  
  
+ TGAGTACTTG AGCACTTGAC TACCTGATTG TTTTTATTTT TTTCAAAATG TAAACTTCTA TTTATTTATT   
  
  
+ TAGGTAAAAC TTGATAATGG CATATTATTG AACCATGCTC TGGCACTTTG CACTTTGCAG ATCCTGTGCA   
  
  
+ TTTCAGTAGT CTGTATAATT CCTACTTGTT TTAAAAAA  

- ATAAAATCAA TCAAAATACC TTCTTATAAT TTTAGTAAAA AGTAAATGTA CAGTTAGATA TATTATACCA   
  
  
- ATTTATTAAC ATATTAAATA CGATAGAGTG TGCTTTTATG AATTGGACAA AATACTCTAC TATATCATTT   
  
  
- TAAACTTTAA GTTAAACAGT TTAAATCAAA AGTAACTCAA AAAAGCGACC TTTTTATATA TGGATTAAAC   
  
  
- TTCAGGGATA TTTAATTAGA CTATTCTAAT AAAATGGTTT TGACGAATTT AAAAGTATAA TTCAACAAAG   
  
  
- ACTGAAAGAC TGCAAAGACA CCATTCTTTA GAACAGATTA CGGAACAAAA TGTTAGTGTT ATCGGATACC   
  
  
- AATAGTTGAA AATATTATCA AACTTCATTT TGAAAAAAAA ACAAACAAAA ATCTTGAAAC TACTGGTCTA   
  
  
- CCAAACTGAA AAACAACGTA TAACTAATTT AAAGTGTACA TTTACGACTG ATTGTAAATT TTAATTCCAA   
  
  
- ACCGTATACT ACTACGGGTA AAGACTACCG TCAAACCATA TATTAAAAAA AGTTAAGTCA TTTTCGTTTA   
  
  
- TGGGGTAAAA TACGATAAAG TGTACTGGAT TGGTTAAAAG ACTTTACTAT ATGATTTTAG ACTGTAAATT   
  
  
- AAATAAACTT GTTTCAAACA ATAATAAAGG TAAGAATAAT ATTATATTTC ATAATGTGAA TAATGATAAT   
  
  
- AATGATGATG TTATTAATAA TAATGATATC AATCATAATA ATGATAATAA TTATGATGAT GGTGATAACA   
  
  
- ATTAAACAAT AATAATGATA ATAATAATAA TAATAATAAT AATAATAATA ATAAAAATGA TGAGAATAAT   
  
  
- GATAATAATA TAATGATGAT GTTATTAATA ATAATGATAT CAATCATAAT AAGAAAAATG ATAATAATAA   
  
  
- TTAATAATTA TAATAATCAA AATCATAAAA AAATTTTAAT AATAATCTCA TTGTGATCAT GATATTTTAA   
  
  
- CTGTAAGAAA AGAAGGGGGG AGAGTTAGAG AGTAAGAGAG AAAGAGAGTC GGGAGAGGCG ACGAGAGTGG   
  
  
- GGAGTGTGAG AGGAAGCTCC GAATAAGTCC TTTAGGGTAT AGAGGAAACT CCGAATAAGA ACCTAAACAA   
  
  
- CAACTTGTCA AGAGATATAT ACACACACAC ACATATATAT ATATATATAT ATATATATAT ATAAAAAAAA   
  
  
- AAAAAAAAAA AAAAAAAAAA CACAAATCTT TAATCTATAA GTCTATAACT TTTCAAATGC AGTTCTAAAA   
  
  
- AGAAAAGGTC TTTTAATTTA TAAAATGGAC CTTAGAAAAC TCAAGGGTAC AGATCAATTG TTAACACACA   
  
  
- TAAAATAAAG GACTTGTTAA AAGTCAACAA TTGTACTTAA AACTTACAAA GAAAAAATAA CAGAGATCCC   
  
  
- CGTATACGTA TAATCGTCTC AACAATTGAA CAATTGATCT TCGTGTACAA ACAGTATAGA AGAAACACAA   
  
  
- TCGAAAAAAT TACCAATTAA CAGTAAGACG ACACGATATA AGATATACAC GAAACTTACG CACATCGATT   
  
  
- TTATTAGGAT TTTGACTTAA TAAACTTGAA GATCTGTTTA TGTTGAGCTA CGGGTCCATT GGAGGTTTCA   
  
  
- AAAAATTTAT TGACTTGATA GAAGTTATAT TATGGTAGTA CTGTCATTCC AATCGAACGA CTCAAATAAT   
  
  
- TTATACAATT GTCAAAGGGG TCAACAGAAC AAACAAAGAG TGAACACGGT AATCCTCCGA CCCCAAACCT   
  
  
- TGCAATCTCT TCTTTCTTTT TCCCTTTTTC TCCCTCTCTT TTTCTTATCT ATATAATATC ACTACTGACT   
  
  
- ACTCATGAAC TCGTGAACTG ATGGACTAAC AAAAATAAAA AAAGTTTTAC ATTTGAAGAT AAATAAATAA   
  
  
- ATCCATTTTG AACTATTACC GTATAATAAC TTGGTACGAG ACCGTGAAAC GTGAAACGTC TAGGACACGT   
  
  
- AAAGTCATCA GACATATTAA GGATGAACAA AATTTTTT

+     Myb

| Site Name | Organism | Position | Strand | Matrix score. | sequence | function |
| --- | --- | --- | --- | --- | --- | --- |
| Myb | Arabidopsis thaliana | 1353 | - | 6 | CAACTG |  |
| Myb | Arabidopsis thaliana | 1700 | - | 6 | CAACTG |  |
| Myb | Arabidopsis thaliana | 1619 | + | 6 | TAACTG |  |

>PlantCARE\_20562   
+ TATTTTAGTT AGTTTTATGG AAGAATATTA AAATCATTTT TCATTTACAT GTCAATCTAT ATAATATGGT   
  
  
+ TAAATAATTG TATAATTTAT GCTATCTCAC ACGAAAATAC TTAACCTGTT TTATGAGATG ATATAGTAAA   
  
  
+ ATTTGAAATT CAATTTGTCA AATTTAGTTT TCATTGAGTT TTTTCGCTGG AAAAATATAT ACCTAATTTG   
  
  
+ AAGTCCCTAT AAATTAATCT GATAAGATTA TTTTACCAAA ACTGCTTAAA TTTTCATATT AAGTTGTTTC   
  
  
+ TGACTTTCTG ACGTTTCTGT GGTAAGAAAT CTTGTCTAAT GCCTTGTTTT ACAATCACAA TAGCCTATGG   
  
  
+ TTATCAACTT TTATAATAGT TTGAAGTAAA ACTTTTTTTT TGTTTGTTTT TAGAACTTTG ATGACCAGAT   
  
  
+ GGTTTGACTT TTTGTTGCAT ATTGATTAAA TTTCACATGT AAATGCTGAC TAACATTTAA AATTAAGGTT   
  
  
+ TGGCATATGA TGATGCCCAT TTCTGATGGC AGTTTGGTAT ATAATTTTTT TCAATTCAGT AAAAGCAAAT   
  
  
+ ACCCCATTTT ATGCTATTTC ACATGACCTA ACCAATTTTC TGAAATGATA TACTAAAATC TGACATTTAA   
  
  
+ TTTATTTGAA CAAAGTTTGT TATTATTTCC ATTCTTATTA TAATATAAAG TATTACACTT ATTACTATTA   
  
  
+ TTACTACTAC AATAATTATT ATTACTATAG TTAGTATTAT TACTATTATT AATACTACTA CCACTATTGT   
  
  
+ TAATTTGTTA TTATTACTAT TATTATTATT ATTATTATTA TTATTATTAT TATTTTTACT ACTCTTATTA   
  
  
+ CTATTATTAT ATTACTACTA CAATAATTAT TATTACTATA GTTAGTATTA TTCTTTTTAC TATTATTATT   
  
  
+ AATTATTAAT ATTATTAGTT TTAGTATTTT TTTAAAATTA TTATTAGAGT AACACTAGTA CTATAAAATT   
  
  
+ GACATTCTTT TCTTCCCCCC TCTCAATCTC TCATTCTCTC TTTCTCTCAG CCCTCTCCGC TGCTCTCACC   
  
  
+ CCTCACACTC TCCTTCGAGG CTTATTCAGG AAATCCCATA TCTCCTTTGA GGCTTATTCT TGGATTTGTT   
  
  
+ GTTGAACAGT TCTCTATATA TGTGTGTGTG TGTATATATA TATATATATA TATATATATA TATTTTTTTT   
  
  
+ TTTTTTTTTT TTTTTTTTTT GTGTTTAGAA ATTAGATATT CAGATATTGA AAAGTTTACG TCAAGATTTT   
  
  
+ TCTTTTCCAG AAAATTAAAT ATTTTACCTG GAATCTTTTG AGTTCCCATG TCTAGTTAAC AATTGTGTGT   
  
  
+ ATTTTATTTC CTGAACAATT TTCAGTTGTT AACATGAATT TTGAATGTTT CTTTTTTATT GTCTCTAGGG   
  
  
+ GCATATGCAT ATTAGCAGAG TTGTTAACTT GTTAACTAGA AGCACATGTT TGTCATATCT TCTTTGTGTT   
  
  
+ AGCTTTTTTA ATGGTTAATT GTCATTCTGC TGTGCTATAT TCTATATGTG CTTTGAATGC GTGTAGCTAA   
  
  
+ AATAATCCTA AAACTGAATT ATTTGAACTT CTAGACAAAT ACAACTCGAT GCCCAGGTAA CCTCCAAAGT   
  
  
+ TTTTTAAATA ACTGAACTAT CTTCAATATA ATACCATCAT GACAGTAAGG TTAGCTTGCT GAGTTTATTA   
  
  
+ AATATGTTAA CAGTTTCCCC AGTTGTCTTG TTTGTTTCTC ACTTGTGCCA TTAGGAGGCT GGGGTTTGGA   
  
  
+ ACGTTAGAGA AGAAAGAAAA AGGGAAAAAG AGGGAGAGAA AAAGAATAGA TATATTATAG TGATGACTGA   
  
  
+ TGAGTACTTG AGCACTTGAC TACCTGATTG TTTTTATTTT TTTCAAAATG TAAACTTCTA TTTATTTATT   
  
  
+ TAGGTAAAAC TTGATAATGG CATATTATTG AACCATGCTC TGGCACTTTG CACTTTGCAG ATCCTGTGCA   
  
  
+ TTTCAGTAGT CTGTATAATT CCTACTTGTT TTAAAAAA  

- ATAAAATCAA TCAAAATACC TTCTTATAAT TTTAGTAAAA AGTAAATGTA CAGTTAGATA TATTATACCA   
  
  
- ATTTATTAAC ATATTAAATA CGATAGAGTG TGCTTTTATG AATTGGACAA AATACTCTAC TATATCATTT   
  
  
- TAAACTTTAA GTTAAACAGT TTAAATCAAA AGTAACTCAA AAAAGCGACC TTTTTATATA TGGATTAAAC   
  
  
- TTCAGGGATA TTTAATTAGA CTATTCTAAT AAAATGGTTT TGACGAATTT AAAAGTATAA TTCAACAAAG   
  
  
- ACTGAAAGAC TGCAAAGACA CCATTCTTTA GAACAGATTA CGGAACAAAA TGTTAGTGTT ATCGGATACC   
  
  
- AATAGTTGAA AATATTATCA AACTTCATTT TGAAAAAAAA ACAAACAAAA ATCTTGAAAC TACTGGTCTA   
  
  
- CCAAACTGAA AAACAACGTA TAACTAATTT AAAGTGTACA TTTACGACTG ATTGTAAATT TTAATTCCAA   
  
  
- ACCGTATACT ACTACGGGTA AAGACTACCG TCAAACCATA TATTAAAAAA AGTTAAGTCA TTTTCGTTTA   
  
  
- TGGGGTAAAA TACGATAAAG TGTACTGGAT TGGTTAAAAG ACTTTACTAT ATGATTTTAG ACTGTAAATT   
  
  
- AAATAAACTT GTTTCAAACA ATAATAAAGG TAAGAATAAT ATTATATTTC ATAATGTGAA TAATGATAAT   
  
  
- AATGATGATG TTATTAATAA TAATGATATC AATCATAATA ATGATAATAA TTATGATGAT GGTGATAACA   
  
  
- ATTAAACAAT AATAATGATA ATAATAATAA TAATAATAAT AATAATAATA ATAAAAATGA TGAGAATAAT   
  
  
- GATAATAATA TAATGATGAT GTTATTAATA ATAATGATAT CAATCATAAT AAGAAAAATG ATAATAATAA   
  
  
- TTAATAATTA TAATAATCAA AATCATAAAA AAATTTTAAT AATAATCTCA TTGTGATCAT GATATTTTAA   
  
  
- CTGTAAGAAA AGAAGGGGGG AGAGTTAGAG AGTAAGAGAG AAAGAGAGTC GGGAGAGGCG ACGAGAGTGG   
  
  
- GGAGTGTGAG AGGAAGCTCC GAATAAGTCC TTTAGGGTAT AGAGGAAACT CCGAATAAGA ACCTAAACAA   
  
  
- CAACTTGTCA AGAGATATAT ACACACACAC ACATATATAT ATATATATAT ATATATATAT ATAAAAAAAA   
  
  
- AAAAAAAAAA AAAAAAAAAA CACAAATCTT TAATCTATAA GTCTATAACT TTTCAAATGC AGTTCTAAAA   
  
  
- AGAAAAGGTC TTTTAATTTA TAAAATGGAC CTTAGAAAAC TCAAGGGTAC AGATCAATTG TTAACACACA   
  
  
- TAAAATAAAG GACTTGTTAA AAGTCAACAA TTGTACTTAA AACTTACAAA GAAAAAATAA CAGAGATCCC   
  
  
- CGTATACGTA TAATCGTCTC AACAATTGAA CAATTGATCT TCGTGTACAA ACAGTATAGA AGAAACACAA   
  
  
- TCGAAAAAAT TACCAATTAA CAGTAAGACG ACACGATATA AGATATACAC GAAACTTACG CACATCGATT   
  
  
- TTATTAGGAT TTTGACTTAA TAAACTTGAA GATCTGTTTA TGTTGAGCTA CGGGTCCATT GGAGGTTTCA   
  
  
- AAAAATTTAT TGACTTGATA GAAGTTATAT TATGGTAGTA CTGTCATTCC AATCGAACGA CTCAAATAAT   
  
  
- TTATACAATT GTCAAAGGGG TCAACAGAAC AAACAAAGAG TGAACACGGT AATCCTCCGA CCCCAAACCT   
  
  
- TGCAATCTCT TCTTTCTTTT TCCCTTTTTC TCCCTCTCTT TTTCTTATCT ATATAATATC ACTACTGACT   
  
  
- ACTCATGAAC TCGTGAACTG ATGGACTAAC AAAAATAAAA AAAGTTTTAC ATTTGAAGAT AAATAAATAA   
  
  
- ATCCATTTTG AACTATTACC GTATAATAAC TTGGTACGAG ACCGTGAAAC GTGAAACGTC TAGGACACGT   
  
  
- AAAGTCATCA GACATATTAA GGATGAACAA AATTTTTT

+     STRE

| Site Name | Organism | Position | Strand | Matrix score. | sequence | function |
| --- | --- | --- | --- | --- | --- | --- |
| STRE | Arabidopsis thaliana | 997 | - | 5 | AGGGG |  |
| STRE | Arabidopsis thaliana | 1397 | + | 5 | AGGGG |  |
| STRE | Arabidopsis thaliana | 1049 | - | 5 | AGGGG |  |

>PlantCARE\_20562   
+ TATTTTAGTT AGTTTTATGG AAGAATATTA AAATCATTTT TCATTTACAT GTCAATCTAT ATAATATGGT   
  
  
+ TAAATAATTG TATAATTTAT GCTATCTCAC ACGAAAATAC TTAACCTGTT TTATGAGATG ATATAGTAAA   
  
  
+ ATTTGAAATT CAATTTGTCA AATTTAGTTT TCATTGAGTT TTTTCGCTGG AAAAATATAT ACCTAATTTG   
  
  
+ AAGTCCCTAT AAATTAATCT GATAAGATTA TTTTACCAAA ACTGCTTAAA TTTTCATATT AAGTTGTTTC   
  
  
+ TGACTTTCTG ACGTTTCTGT GGTAAGAAAT CTTGTCTAAT GCCTTGTTTT ACAATCACAA TAGCCTATGG   
  
  
+ TTATCAACTT TTATAATAGT TTGAAGTAAA ACTTTTTTTT TGTTTGTTTT TAGAACTTTG ATGACCAGAT   
  
  
+ GGTTTGACTT TTTGTTGCAT ATTGATTAAA TTTCACATGT AAATGCTGAC TAACATTTAA AATTAAGGTT   
  
  
+ TGGCATATGA TGATGCCCAT TTCTGATGGC AGTTTGGTAT ATAATTTTTT TCAATTCAGT AAAAGCAAAT   
  
  
+ ACCCCATTTT ATGCTATTTC ACATGACCTA ACCAATTTTC TGAAATGATA TACTAAAATC TGACATTTAA   
  
  
+ TTTATTTGAA CAAAGTTTGT TATTATTTCC ATTCTTATTA TAATATAAAG TATTACACTT ATTACTATTA   
  
  
+ TTACTACTAC AATAATTATT ATTACTATAG TTAGTATTAT TACTATTATT AATACTACTA CCACTATTGT   
  
  
+ TAATTTGTTA TTATTACTAT TATTATTATT ATTATTATTA TTATTATTAT TATTTTTACT ACTCTTATTA   
  
  
+ CTATTATTAT ATTACTACTA CAATAATTAT TATTACTATA GTTAGTATTA TTCTTTTTAC TATTATTATT   
  
  
+ AATTATTAAT ATTATTAGTT TTAGTATTTT TTTAAAATTA TTATTAGAGT AACACTAGTA CTATAAAATT   
  
  
+ GACATTCTTT TCTTCCCCCC TCTCAATCTC TCATTCTCTC TTTCTCTCAG CCCTCTCCGC TGCTCTCACC   
  
  
+ CCTCACACTC TCCTTCGAGG CTTATTCAGG AAATCCCATA TCTCCTTTGA GGCTTATTCT TGGATTTGTT   
  
  
+ GTTGAACAGT TCTCTATATA TGTGTGTGTG TGTATATATA TATATATATA TATATATATA TATTTTTTTT   
  
  
+ TTTTTTTTTT TTTTTTTTTT GTGTTTAGAA ATTAGATATT CAGATATTGA AAAGTTTACG TCAAGATTTT   
  
  
+ TCTTTTCCAG AAAATTAAAT ATTTTACCTG GAATCTTTTG AGTTCCCATG TCTAGTTAAC AATTGTGTGT   
  
  
+ ATTTTATTTC CTGAACAATT TTCAGTTGTT AACATGAATT TTGAATGTTT CTTTTTTATT GTCTCTAGGG   
  
  
+ GCATATGCAT ATTAGCAGAG TTGTTAACTT GTTAACTAGA AGCACATGTT TGTCATATCT TCTTTGTGTT   
  
  
+ AGCTTTTTTA ATGGTTAATT GTCATTCTGC TGTGCTATAT TCTATATGTG CTTTGAATGC GTGTAGCTAA   
  
  
+ AATAATCCTA AAACTGAATT ATTTGAACTT CTAGACAAAT ACAACTCGAT GCCCAGGTAA CCTCCAAAGT   
  
  
+ TTTTTAAATA ACTGAACTAT CTTCAATATA ATACCATCAT GACAGTAAGG TTAGCTTGCT GAGTTTATTA   
  
  
+ AATATGTTAA CAGTTTCCCC AGTTGTCTTG TTTGTTTCTC ACTTGTGCCA TTAGGAGGCT GGGGTTTGGA   
  
  
+ ACGTTAGAGA AGAAAGAAAA AGGGAAAAAG AGGGAGAGAA AAAGAATAGA TATATTATAG TGATGACTGA   
  
  
+ TGAGTACTTG AGCACTTGAC TACCTGATTG TTTTTATTTT TTTCAAAATG TAAACTTCTA TTTATTTATT   
  
  
+ TAGGTAAAAC TTGATAATGG CATATTATTG AACCATGCTC TGGCACTTTG CACTTTGCAG ATCCTGTGCA   
  
  
+ TTTCAGTAGT CTGTATAATT CCTACTTGTT TTAAAAAA  

- ATAAAATCAA TCAAAATACC TTCTTATAAT TTTAGTAAAA AGTAAATGTA CAGTTAGATA TATTATACCA   
  
  
- ATTTATTAAC ATATTAAATA CGATAGAGTG TGCTTTTATG AATTGGACAA AATACTCTAC TATATCATTT   
  
  
- TAAACTTTAA GTTAAACAGT TTAAATCAAA AGTAACTCAA AAAAGCGACC TTTTTATATA TGGATTAAAC   
  
  
- TTCAGGGATA TTTAATTAGA CTATTCTAAT AAAATGGTTT TGACGAATTT AAAAGTATAA TTCAACAAAG   
  
  
- ACTGAAAGAC TGCAAAGACA CCATTCTTTA GAACAGATTA CGGAACAAAA TGTTAGTGTT ATCGGATACC   
  
  
- AATAGTTGAA AATATTATCA AACTTCATTT TGAAAAAAAA ACAAACAAAA ATCTTGAAAC TACTGGTCTA   
  
  
- CCAAACTGAA AAACAACGTA TAACTAATTT AAAGTGTACA TTTACGACTG ATTGTAAATT TTAATTCCAA   
  
  
- ACCGTATACT ACTACGGGTA AAGACTACCG TCAAACCATA TATTAAAAAA AGTTAAGTCA TTTTCGTTTA   
  
  
- TGGGGTAAAA TACGATAAAG TGTACTGGAT TGGTTAAAAG ACTTTACTAT ATGATTTTAG ACTGTAAATT   
  
  
- AAATAAACTT GTTTCAAACA ATAATAAAGG TAAGAATAAT ATTATATTTC ATAATGTGAA TAATGATAAT   
  
  
- AATGATGATG TTATTAATAA TAATGATATC AATCATAATA ATGATAATAA TTATGATGAT GGTGATAACA   
  
  
- ATTAAACAAT AATAATGATA ATAATAATAA TAATAATAAT AATAATAATA ATAAAAATGA TGAGAATAAT   
  
  
- GATAATAATA TAATGATGAT GTTATTAATA ATAATGATAT CAATCATAAT AAGAAAAATG ATAATAATAA   
  
  
- TTAATAATTA TAATAATCAA AATCATAAAA AAATTTTAAT AATAATCTCA TTGTGATCAT GATATTTTAA   
  
  
- CTGTAAGAAA AGAAGGGGGG AGAGTTAGAG AGTAAGAGAG AAAGAGAGTC GGGAGAGGCG ACGAGAGTGG   
  
  
- GGAGTGTGAG AGGAAGCTCC GAATAAGTCC TTTAGGGTAT AGAGGAAACT CCGAATAAGA ACCTAAACAA   
  
  
- CAACTTGTCA AGAGATATAT ACACACACAC ACATATATAT ATATATATAT ATATATATAT ATAAAAAAAA   
  
  
- AAAAAAAAAA AAAAAAAAAA CACAAATCTT TAATCTATAA GTCTATAACT TTTCAAATGC AGTTCTAAAA   
  
  
- AGAAAAGGTC TTTTAATTTA TAAAATGGAC CTTAGAAAAC TCAAGGGTAC AGATCAATTG TTAACACACA   
  
  
- TAAAATAAAG GACTTGTTAA AAGTCAACAA TTGTACTTAA AACTTACAAA GAAAAAATAA CAGAGATCCC   
  
  
- CGTATACGTA TAATCGTCTC AACAATTGAA CAATTGATCT TCGTGTACAA ACAGTATAGA AGAAACACAA   
  
  
- TCGAAAAAAT TACCAATTAA CAGTAAGACG ACACGATATA AGATATACAC GAAACTTACG CACATCGATT   
  
  
- TTATTAGGAT TTTGACTTAA TAAACTTGAA GATCTGTTTA TGTTGAGCTA CGGGTCCATT GGAGGTTTCA   
  
  
- AAAAATTTAT TGACTTGATA GAAGTTATAT TATGGTAGTA CTGTCATTCC AATCGAACGA CTCAAATAAT   
  
  
- TTATACAATT GTCAAAGGGG TCAACAGAAC AAACAAAGAG TGAACACGGT AATCCTCCGA CCCCAAACCT   
  
  
- TGCAATCTCT TCTTTCTTTT TCCCTTTTTC TCCCTCTCTT TTTCTTATCT ATATAATATC ACTACTGACT   
  
  
- ACTCATGAAC TCGTGAACTG ATGGACTAAC AAAAATAAAA AAAGTTTTAC ATTTGAAGAT AAATAAATAA   
  
  
- ATCCATTTTG AACTATTACC GTATAATAAC TTGGTACGAG ACCGTGAAAC GTGAAACGTC TAGGACACGT   
  
  
- AAAGTCATCA GACATATTAA GGATGAACAA AATTTTTT

+     TATA

| Site Name | Organism | Position | Strand | Matrix score. | sequence | function |
| --- | --- | --- | --- | --- | --- | --- |
| TATA | Arabidopsis thaliana | 972 | + | 8 | TATAAAAT |  |

>PlantCARE\_20562   
+ TATTTTAGTT AGTTTTATGG AAGAATATTA AAATCATTTT TCATTTACAT GTCAATCTAT ATAATATGGT   
  
  
+ TAAATAATTG TATAATTTAT GCTATCTCAC ACGAAAATAC TTAACCTGTT TTATGAGATG ATATAGTAAA   
  
  
+ ATTTGAAATT CAATTTGTCA AATTTAGTTT TCATTGAGTT TTTTCGCTGG AAAAATATAT ACCTAATTTG   
  
  
+ AAGTCCCTAT AAATTAATCT GATAAGATTA TTTTACCAAA ACTGCTTAAA TTTTCATATT AAGTTGTTTC   
  
  
+ TGACTTTCTG ACGTTTCTGT GGTAAGAAAT CTTGTCTAAT GCCTTGTTTT ACAATCACAA TAGCCTATGG   
  
  
+ TTATCAACTT TTATAATAGT TTGAAGTAAA ACTTTTTTTT TGTTTGTTTT TAGAACTTTG ATGACCAGAT   
  
  
+ GGTTTGACTT TTTGTTGCAT ATTGATTAAA TTTCACATGT AAATGCTGAC TAACATTTAA AATTAAGGTT   
  
  
+ TGGCATATGA TGATGCCCAT TTCTGATGGC AGTTTGGTAT ATAATTTTTT TCAATTCAGT AAAAGCAAAT   
  
  
+ ACCCCATTTT ATGCTATTTC ACATGACCTA ACCAATTTTC TGAAATGATA TACTAAAATC TGACATTTAA   
  
  
+ TTTATTTGAA CAAAGTTTGT TATTATTTCC ATTCTTATTA TAATATAAAG TATTACACTT ATTACTATTA   
  
  
+ TTACTACTAC AATAATTATT ATTACTATAG TTAGTATTAT TACTATTATT AATACTACTA CCACTATTGT   
  
  
+ TAATTTGTTA TTATTACTAT TATTATTATT ATTATTATTA TTATTATTAT TATTTTTACT ACTCTTATTA   
  
  
+ CTATTATTAT ATTACTACTA CAATAATTAT TATTACTATA GTTAGTATTA TTCTTTTTAC TATTATTATT   
  
  
+ AATTATTAAT ATTATTAGTT TTAGTATTTT TTTAAAATTA TTATTAGAGT AACACTAGTA CTATAAAATT   
  
  
+ GACATTCTTT TCTTCCCCCC TCTCAATCTC TCATTCTCTC TTTCTCTCAG CCCTCTCCGC TGCTCTCACC   
  
  
+ CCTCACACTC TCCTTCGAGG CTTATTCAGG AAATCCCATA TCTCCTTTGA GGCTTATTCT TGGATTTGTT   
  
  
+ GTTGAACAGT TCTCTATATA TGTGTGTGTG TGTATATATA TATATATATA TATATATATA TATTTTTTTT   
  
  
+ TTTTTTTTTT TTTTTTTTTT GTGTTTAGAA ATTAGATATT CAGATATTGA AAAGTTTACG TCAAGATTTT   
  
  
+ TCTTTTCCAG AAAATTAAAT ATTTTACCTG GAATCTTTTG AGTTCCCATG TCTAGTTAAC AATTGTGTGT   
  
  
+ ATTTTATTTC CTGAACAATT TTCAGTTGTT AACATGAATT TTGAATGTTT CTTTTTTATT GTCTCTAGGG   
  
  
+ GCATATGCAT ATTAGCAGAG TTGTTAACTT GTTAACTAGA AGCACATGTT TGTCATATCT TCTTTGTGTT   
  
  
+ AGCTTTTTTA ATGGTTAATT GTCATTCTGC TGTGCTATAT TCTATATGTG CTTTGAATGC GTGTAGCTAA   
  
  
+ AATAATCCTA AAACTGAATT ATTTGAACTT CTAGACAAAT ACAACTCGAT GCCCAGGTAA CCTCCAAAGT   
  
  
+ TTTTTAAATA ACTGAACTAT CTTCAATATA ATACCATCAT GACAGTAAGG TTAGCTTGCT GAGTTTATTA   
  
  
+ AATATGTTAA CAGTTTCCCC AGTTGTCTTG TTTGTTTCTC ACTTGTGCCA TTAGGAGGCT GGGGTTTGGA   
  
  
+ ACGTTAGAGA AGAAAGAAAA AGGGAAAAAG AGGGAGAGAA AAAGAATAGA TATATTATAG TGATGACTGA   
  
  
+ TGAGTACTTG AGCACTTGAC TACCTGATTG TTTTTATTTT TTTCAAAATG TAAACTTCTA TTTATTTATT   
  
  
+ TAGGTAAAAC TTGATAATGG CATATTATTG AACCATGCTC TGGCACTTTG CACTTTGCAG ATCCTGTGCA   
  
  
+ TTTCAGTAGT CTGTATAATT CCTACTTGTT TTAAAAAA  

- ATAAAATCAA TCAAAATACC TTCTTATAAT TTTAGTAAAA AGTAAATGTA CAGTTAGATA TATTATACCA   
  
  
- ATTTATTAAC ATATTAAATA CGATAGAGTG TGCTTTTATG AATTGGACAA AATACTCTAC TATATCATTT   
  
  
- TAAACTTTAA GTTAAACAGT TTAAATCAAA AGTAACTCAA AAAAGCGACC TTTTTATATA TGGATTAAAC   
  
  
- TTCAGGGATA TTTAATTAGA CTATTCTAAT AAAATGGTTT TGACGAATTT AAAAGTATAA TTCAACAAAG   
  
  
- ACTGAAAGAC TGCAAAGACA CCATTCTTTA GAACAGATTA CGGAACAAAA TGTTAGTGTT ATCGGATACC   
  
  
- AATAGTTGAA AATATTATCA AACTTCATTT TGAAAAAAAA ACAAACAAAA ATCTTGAAAC TACTGGTCTA   
  
  
- CCAAACTGAA AAACAACGTA TAACTAATTT AAAGTGTACA TTTACGACTG ATTGTAAATT TTAATTCCAA   
  
  
- ACCGTATACT ACTACGGGTA AAGACTACCG TCAAACCATA TATTAAAAAA AGTTAAGTCA TTTTCGTTTA   
  
  
- TGGGGTAAAA TACGATAAAG TGTACTGGAT TGGTTAAAAG ACTTTACTAT ATGATTTTAG ACTGTAAATT   
  
  
- AAATAAACTT GTTTCAAACA ATAATAAAGG TAAGAATAAT ATTATATTTC ATAATGTGAA TAATGATAAT   
  
  
- AATGATGATG TTATTAATAA TAATGATATC AATCATAATA ATGATAATAA TTATGATGAT GGTGATAACA   
  
  
- ATTAAACAAT AATAATGATA ATAATAATAA TAATAATAAT AATAATAATA ATAAAAATGA TGAGAATAAT   
  
  
- GATAATAATA TAATGATGAT GTTATTAATA ATAATGATAT CAATCATAAT AAGAAAAATG ATAATAATAA   
  
  
- TTAATAATTA TAATAATCAA AATCATAAAA AAATTTTAAT AATAATCTCA TTGTGATCAT GATATTTTAA   
  
  
- CTGTAAGAAA AGAAGGGGGG AGAGTTAGAG AGTAAGAGAG AAAGAGAGTC GGGAGAGGCG ACGAGAGTGG   
  
  
- GGAGTGTGAG AGGAAGCTCC GAATAAGTCC TTTAGGGTAT AGAGGAAACT CCGAATAAGA ACCTAAACAA   
  
  
- CAACTTGTCA AGAGATATAT ACACACACAC ACATATATAT ATATATATAT ATATATATAT ATAAAAAAAA   
  
  
- AAAAAAAAAA AAAAAAAAAA CACAAATCTT TAATCTATAA GTCTATAACT TTTCAAATGC AGTTCTAAAA   
  
  
- AGAAAAGGTC TTTTAATTTA TAAAATGGAC CTTAGAAAAC TCAAGGGTAC AGATCAATTG TTAACACACA   
  
  
- TAAAATAAAG GACTTGTTAA AAGTCAACAA TTGTACTTAA AACTTACAAA GAAAAAATAA CAGAGATCCC   
  
  
- CGTATACGTA TAATCGTCTC AACAATTGAA CAATTGATCT TCGTGTACAA ACAGTATAGA AGAAACACAA   
  
  
- TCGAAAAAAT TACCAATTAA CAGTAAGACG ACACGATATA AGATATACAC GAAACTTACG CACATCGATT   
  
  
- TTATTAGGAT TTTGACTTAA TAAACTTGAA GATCTGTTTA TGTTGAGCTA CGGGTCCATT GGAGGTTTCA   
  
  
- AAAAATTTAT TGACTTGATA GAAGTTATAT TATGGTAGTA CTGTCATTCC AATCGAACGA CTCAAATAAT   
  
  
- TTATACAATT GTCAAAGGGG TCAACAGAAC AAACAAAGAG TGAACACGGT AATCCTCCGA CCCCAAACCT   
  
  
- TGCAATCTCT TCTTTCTTTT TCCCTTTTTC TCCCTCTCTT TTTCTTATCT ATATAATATC ACTACTGACT   
  
  
- ACTCATGAAC TCGTGAACTG ATGGACTAAC AAAAATAAAA AAAGTTTTAC ATTTGAAGAT AAATAAATAA   
  
  
- ATCCATTTTG AACTATTACC GTATAATAAC TTGGTACGAG ACCGTGAAAC GTGAAACGTC TAGGACACGT   
  
  
- AAAGTCATCA GACATATTAA GGATGAACAA AATTTTTT

+     TATA-box

| Site Name | Organism | Position | Strand | Matrix score. | sequence | function |
| --- | --- | --- | --- | --- | --- | --- |
| TATA-box | Arabidopsis thaliana | 198 | + | 4 | TATA | core promoter element around -30 of transcription start |
| TATA-box | Arabidopsis thaliana | 669 | + | 4 | TATA | core promoter element around -30 of transcription start |
| TATA-box | Arabidopsis thaliana | 361 | - | 5 | TATAA | core promoter element around -30 of transcription start |
| TATA-box | Brassica napus | 846 | + | 6 | ATTATA | core promoter element around -30 of transcription start |
| TATA-box | Arabidopsis thaliana | 528 | + | 6 | TATATA | core promoter element around -30 of transcription start |
| TATA-box | Arabidopsis thaliana | 530 | + | 4 | TATA | core promoter element around -30 of transcription start |
| TATA-box | Arabidopsis thaliana | 362 | + | 4 | TATA | core promoter element around -30 of transcription start |
| TATA-box | Arabidopsis thaliana | 972 | + | 4 | TATA | core promoter element around -30 of transcription start |
| TATA-box | Brassica oleracea | 529 | + | 6 | ATATAA | core promoter element around -30 of transcription start |
| TATA-box | Arabidopsis thaliana | 674 | + | 4 | TATA | core promoter element around -30 of transcription start |
| TATA-box | Arabidopsis thaliana | 58 | + | 6 | TATATA | core promoter element around -30 of transcription start |
| TATA-box | Arabidopsis thaliana | 877 | + | 4 | TATA | core promoter element around -30 of transcription start |
| TATA-box | Arabidopsis thaliana | 81 | + | 4 | TATA | core promoter element around -30 of transcription start |
| TATA-box | Arabidopsis thaliana | 609 | + | 4 | TATA | core promoter element around -30 of transcription start |
| TATA-box | Pisum sativum | 359 | - | 7 | TATAAAA | core promoter element around -30 of transcription start |
| TATA-box | Arabidopsis thaliana | 1135 | - | 6 | TATATA | core promoter element around -30 of transcription start |
| TATA-box | Arabidopsis thaliana | 60 | + | 4 | TATA | core promoter element around -30 of transcription start |
| TATA-box | Brassica napus | 667 | + | 6 | ATTATA | core promoter element around -30 of transcription start |
| TATA-box | Arabidopsis thaliana | 218 | + | 4 | TATA | core promoter element around -30 of transcription start |
| TATA-box | Arabidopsis thaliana | 847 | - | 5 | TATAA | core promoter element around -30 of transcription start |
| TATA-box | Brassica napus | 195 | + | 6 | ATATAT | core promoter element around -30 of transcription start |
| TATA-box | Arabidopsis thaliana | 726 | + | 4 | TATA | core promoter element around -30 of transcription start |
| TATA-box | Helianthus annuus | 360 | - | 6 | TATAAA | core promoter element around -30 of transcription start |
| TATA-box | Brassica napus | 1136 | - | 6 | ATATAT | core promoter element around -30 of transcription start |
| TATA-box | Brassica oleracea | 59 | + | 6 | ATATAA | core promoter element around -30 of transcription start |
| TATA-box | Arabidopsis thaliana | 668 | - | 5 | TATAA | core promoter element around -30 of transcription start |
| TATA-box | Arabidopsis thaliana | 132 | + | 4 | TATA | core promoter element around -30 of transcription start |
| TATA-box | Arabidopsis thaliana | 848 | + | 4 | TATA | core promoter element around -30 of transcription start |
| TATA-box | Helianthus annuus | 79 | - | 6 | TATACA | core promoter element around -30 of transcription start |
| TATA-box | Brassica oleracea | 673 | + | 6 | ATATAA | core promoter element around -30 of transcription start |
| TATA-box | Arabidopsis thaliana | 196 | + | 6 | TATATA | core promoter element around -30 of transcription start |
| TATA-box | Arabidopsis thaliana | 1137 | - | 4 | TATA | core promoter element around -30 of transcription start |
| TATA-box | Helianthus annuus | 1151 | - | 6 | TATACA | core promoter element around -30 of transcription start |
| TATA-box | Arabidopsis thaliana | 1153 | - | 6 | TATATA | core promoter element around -30 of transcription start |
| TATA-box | Brassica napus | 1154 | - | 6 | ATATAT | core promoter element around -30 of transcription start |
| TATA-box | Arabidopsis thaliana | 1155 | - | 6 | TATATA | core promoter element around -30 of transcription start |
| TATA-box | Brassica napus | 1156 | - | 6 | ATATAT | core promoter element around -30 of transcription start |
| TATA-box | Arabidopsis thaliana | 1157 | - | 6 | TATATA | core promoter element around -30 of transcription start |
| TATA-box | Brassica napus | 1158 | - | 6 | ATATAT | core promoter element around -30 of transcription start |
| TATA-box | Arabidopsis thaliana | 1159 | - | 6 | TATATA | core promoter element around -30 of transcription start |
| TATA-box | Brassica napus | 1160 | - | 6 | ATATAT | core promoter element around -30 of transcription start |
| TATA-box | Arabidopsis thaliana | 1161 | - | 6 | TATATA | core promoter element around -30 of transcription start |
| TATA-box | Brassica napus | 1162 | - | 6 | ATATAT | core promoter element around -30 of transcription start |
| TATA-box | Arabidopsis thaliana | 1163 | - | 6 | TATATA | core promoter element around -30 of transcription start |
| TATA-box | Brassica napus | 1164 | - | 6 | ATATAT | core promoter element around -30 of transcription start |
| TATA-box | Arabidopsis thaliana | 1165 | - | 6 | TATATA | core promoter element around -30 of transcription start |
| TATA-box | Brassica napus | 1166 | - | 6 | ATATAT | core promoter element around -30 of transcription start |
| TATA-box | Arabidopsis thaliana | 1167 | - | 6 | TATATA | core promoter element around -30 of transcription start |
| TATA-box | Brassica napus | 1168 | - | 6 | ATATAT | core promoter element around -30 of transcription start |
| TATA-box | Arabidopsis thaliana | 1169 | - | 6 | TATATA | core promoter element around -30 of transcription start |
| TATA-box | Brassica napus | 1170 | - | 6 | ATATAT | core promoter element around -30 of transcription start |
| TATA-box | Arabidopsis thaliana | 1171 | - | 6 | TATATA | core promoter element around -30 of transcription start |
| TATA-box | Brassica napus | 1172 | - | 6 | ATATAT | core promoter element around -30 of transcription start |
| TATA-box | Avena sativa | 1173 | + | 12 | TATATTTATATTT | core promoter element around -30 of transcription start |
| TATA-box | Brassica napus | 1174 | - | 6 | ATATAT | core promoter element around -30 of transcription start |
| TATA-box | Arabidopsis thaliana | 1175 | - | 6 | TATATA | core promoter element around -30 of transcription start |
| TATA-box | Brassica napus | 1176 | - | 6 | ATATAT | core promoter element around -30 of transcription start |
| TATA-box | Arabidopsis thaliana | 1177 | - | 6 | TATATA | core promoter element around -30 of transcription start |
| TATA-box | Brassica napus | 1178 | - | 6 | ATATAT | core promoter element around -30 of transcription start |
| TATA-box | Arabidopsis thaliana | 1179 | - | 4 | TATA | core promoter element around -30 of transcription start |
| TATA-box | Arabidopsis thaliana | 1506 | - | 4 | TATA | core promoter element around -30 of transcription start |
| TATA-box | Arabidopsis thaliana | 1513 | - | 4 | TATA | core promoter element around -30 of transcription start |
| TATA-box | Arabidopsis thaliana | 1613 | - | 8 | TATTTAAA | core promoter element around -30 of transcription start |
| TATA-box | Brassica oleracea | 1636 | + | 6 | ATATAA | core promoter element around -30 of transcription start |
| TATA-box | Arabidopsis thaliana | 1637 | - | 4 | TATA | core promoter element around -30 of transcription start |
| TATA-box | Brassica napus | 1800 | - | 6 | ATATAT | core promoter element around -30 of transcription start |
| TATA-box | Arabidopsis thaliana | 1801 | - | 4 | TATA | core promoter element around -30 of transcription start |
| TATA-box | Brassica napus | 1804 | + | 6 | ATTATA | core promoter element around -30 of transcription start |
| TATA-box | Arabidopsis thaliana | 1805 | - | 5 | TATAA | core promoter element around -30 of transcription start |
| TATA-box | Arabidopsis thaliana | 1806 | - | 4 | TATA | core promoter element around -30 of transcription start |
| TATA-box | Helianthus annuus | 1972 | - | 6 | TATACA | core promoter element around -30 of transcription start |
| TATA-box | Arabidopsis thaliana | 1974 | - | 4 | TATA | core promoter element around -30 of transcription start |

>PlantCARE\_20562   
+ TATTTTAGTT AGTTTTATGG AAGAATATTA AAATCATTTT TCATTTACAT GTCAATCTAT ATAATATGGT   
  
  
+ TAAATAATTG TATAATTTAT GCTATCTCAC ACGAAAATAC TTAACCTGTT TTATGAGATG ATATAGTAAA   
  
  
+ ATTTGAAATT CAATTTGTCA AATTTAGTTT TCATTGAGTT TTTTCGCTGG AAAAATATAT ACCTAATTTG   
  
  
+ AAGTCCCTAT AAATTAATCT GATAAGATTA TTTTACCAAA ACTGCTTAAA TTTTCATATT AAGTTGTTTC   
  
  
+ TGACTTTCTG ACGTTTCTGT GGTAAGAAAT CTTGTCTAAT GCCTTGTTTT ACAATCACAA TAGCCTATGG   
  
  
+ TTATCAACTT TTATAATAGT TTGAAGTAAA ACTTTTTTTT TGTTTGTTTT TAGAACTTTG ATGACCAGAT   
  
  
+ GGTTTGACTT TTTGTTGCAT ATTGATTAAA TTTCACATGT AAATGCTGAC TAACATTTAA AATTAAGGTT   
  
  
+ TGGCATATGA TGATGCCCAT TTCTGATGGC AGTTTGGTAT ATAATTTTTT TCAATTCAGT AAAAGCAAAT   
  
  
+ ACCCCATTTT ATGCTATTTC ACATGACCTA ACCAATTTTC TGAAATGATA TACTAAAATC TGACATTTAA   
  
  
+ TTTATTTGAA CAAAGTTTGT TATTATTTCC ATTCTTATTA TAATATAAAG TATTACACTT ATTACTATTA   
  
  
+ TTACTACTAC AATAATTATT ATTACTATAG TTAGTATTAT TACTATTATT AATACTACTA CCACTATTGT   
  
  
+ TAATTTGTTA TTATTACTAT TATTATTATT ATTATTATTA TTATTATTAT TATTTTTACT ACTCTTATTA   
  
  
+ CTATTATTAT ATTACTACTA CAATAATTAT TATTACTATA GTTAGTATTA TTCTTTTTAC TATTATTATT   
  
  
+ AATTATTAAT ATTATTAGTT TTAGTATTTT TTTAAAATTA TTATTAGAGT AACACTAGTA CTATAAAATT   
  
  
+ GACATTCTTT TCTTCCCCCC TCTCAATCTC TCATTCTCTC TTTCTCTCAG CCCTCTCCGC TGCTCTCACC   
  
  
+ CCTCACACTC TCCTTCGAGG CTTATTCAGG AAATCCCATA TCTCCTTTGA GGCTTATTCT TGGATTTGTT   
  
  
+ GTTGAACAGT TCTCTATATA TGTGTGTGTG TGTATATATA TATATATATA TATATATATA TATTTTTTTT   
  
  
+ TTTTTTTTTT TTTTTTTTTT GTGTTTAGAA ATTAGATATT CAGATATTGA AAAGTTTACG TCAAGATTTT   
  
  
+ TCTTTTCCAG AAAATTAAAT ATTTTACCTG GAATCTTTTG AGTTCCCATG TCTAGTTAAC AATTGTGTGT   
  
  
+ ATTTTATTTC CTGAACAATT TTCAGTTGTT AACATGAATT TTGAATGTTT CTTTTTTATT GTCTCTAGGG   
  
  
+ GCATATGCAT ATTAGCAGAG TTGTTAACTT GTTAACTAGA AGCACATGTT TGTCATATCT TCTTTGTGTT   
  
  
+ AGCTTTTTTA ATGGTTAATT GTCATTCTGC TGTGCTATAT TCTATATGTG CTTTGAATGC GTGTAGCTAA   
  
  
+ AATAATCCTA AAACTGAATT ATTTGAACTT CTAGACAAAT ACAACTCGAT GCCCAGGTAA CCTCCAAAGT   
  
  
+ TTTTTAAATA ACTGAACTAT CTTCAATATA ATACCATCAT GACAGTAAGG TTAGCTTGCT GAGTTTATTA   
  
  
+ AATATGTTAA CAGTTTCCCC AGTTGTCTTG TTTGTTTCTC ACTTGTGCCA TTAGGAGGCT GGGGTTTGGA   
  
  
+ ACGTTAGAGA AGAAAGAAAA AGGGAAAAAG AGGGAGAGAA AAAGAATAGA TATATTATAG TGATGACTGA   
  
  
+ TGAGTACTTG AGCACTTGAC TACCTGATTG TTTTTATTTT TTTCAAAATG TAAACTTCTA TTTATTTATT   
  
  
+ TAGGTAAAAC TTGATAATGG CATATTATTG AACCATGCTC TGGCACTTTG CACTTTGCAG ATCCTGTGCA   
  
  
+ TTTCAGTAGT CTGTATAATT CCTACTTGTT TTAAAAAA  

- ATAAAATCAA TCAAAATACC TTCTTATAAT TTTAGTAAAA AGTAAATGTA CAGTTAGATA TATTATACCA   
  
  
- ATTTATTAAC ATATTAAATA CGATAGAGTG TGCTTTTATG AATTGGACAA AATACTCTAC TATATCATTT   
  
  
- TAAACTTTAA GTTAAACAGT TTAAATCAAA AGTAACTCAA AAAAGCGACC TTTTTATATA TGGATTAAAC   
  
  
- TTCAGGGATA TTTAATTAGA CTATTCTAAT AAAATGGTTT TGACGAATTT AAAAGTATAA TTCAACAAAG   
  
  
- ACTGAAAGAC TGCAAAGACA CCATTCTTTA GAACAGATTA CGGAACAAAA TGTTAGTGTT ATCGGATACC   
  
  
- AATAGTTGAA AATATTATCA AACTTCATTT TGAAAAAAAA ACAAACAAAA ATCTTGAAAC TACTGGTCTA   
  
  
- CCAAACTGAA AAACAACGTA TAACTAATTT AAAGTGTACA TTTACGACTG ATTGTAAATT TTAATTCCAA   
  
  
- ACCGTATACT ACTACGGGTA AAGACTACCG TCAAACCATA TATTAAAAAA AGTTAAGTCA TTTTCGTTTA   
  
  
- TGGGGTAAAA TACGATAAAG TGTACTGGAT TGGTTAAAAG ACTTTACTAT ATGATTTTAG ACTGTAAATT   
  
  
- AAATAAACTT GTTTCAAACA ATAATAAAGG TAAGAATAAT ATTATATTTC ATAATGTGAA TAATGATAAT   
  
  
- AATGATGATG TTATTAATAA TAATGATATC AATCATAATA ATGATAATAA TTATGATGAT GGTGATAACA   
  
  
- ATTAAACAAT AATAATGATA ATAATAATAA TAATAATAAT AATAATAATA ATAAAAATGA TGAGAATAAT   
  
  
- GATAATAATA TAATGATGAT GTTATTAATA ATAATGATAT CAATCATAAT AAGAAAAATG ATAATAATAA   
  
  
- TTAATAATTA TAATAATCAA AATCATAAAA AAATTTTAAT AATAATCTCA TTGTGATCAT GATATTTTAA   
  
  
- CTGTAAGAAA AGAAGGGGGG AGAGTTAGAG AGTAAGAGAG AAAGAGAGTC GGGAGAGGCG ACGAGAGTGG   
  
  
- GGAGTGTGAG AGGAAGCTCC GAATAAGTCC TTTAGGGTAT AGAGGAAACT CCGAATAAGA ACCTAAACAA   
  
  
- CAACTTGTCA AGAGATATAT ACACACACAC ACATATATAT ATATATATAT ATATATATAT ATAAAAAAAA   
  
  
- AAAAAAAAAA AAAAAAAAAA CACAAATCTT TAATCTATAA GTCTATAACT TTTCAAATGC AGTTCTAAAA   
  
  
- AGAAAAGGTC TTTTAATTTA TAAAATGGAC CTTAGAAAAC TCAAGGGTAC AGATCAATTG TTAACACACA   
  
  
- TAAAATAAAG GACTTGTTAA AAGTCAACAA TTGTACTTAA AACTTACAAA GAAAAAATAA CAGAGATCCC   
  
  
- CGTATACGTA TAATCGTCTC AACAATTGAA CAATTGATCT TCGTGTACAA ACAGTATAGA AGAAACACAA   
  
  
- TCGAAAAAAT TACCAATTAA CAGTAAGACG ACACGATATA AGATATACAC GAAACTTACG CACATCGATT   
  
  
- TTATTAGGAT TTTGACTTAA TAAACTTGAA GATCTGTTTA TGTTGAGCTA CGGGTCCATT GGAGGTTTCA   
  
  
- AAAAATTTAT TGACTTGATA GAAGTTATAT TATGGTAGTA CTGTCATTCC AATCGAACGA CTCAAATAAT   
  
  
- TTATACAATT GTCAAAGGGG TCAACAGAAC AAACAAAGAG TGAACACGGT AATCCTCCGA CCCCAAACCT   
  
  
- TGCAATCTCT TCTTTCTTTT TCCCTTTTTC TCCCTCTCTT TTTCTTATCT ATATAATATC ACTACTGACT   
  
  
- ACTCATGAAC TCGTGAACTG ATGGACTAAC AAAAATAAAA AAAGTTTTAC ATTTGAAGAT AAATAAATAA   
  
  
- ATCCATTTTG AACTATTACC GTATAATAAC TTGGTACGAG ACCGTGAAAC GTGAAACGTC TAGGACACGT   
  
  
- AAAGTCATCA GACATATTAA GGATGAACAA AATTTTTT

+     TC-rich repeats

| Site Name | Organism | Position | Strand | Matrix score. | sequence | function |
| --- | --- | --- | --- | --- | --- | --- |
| TC-rich repeats | Nicotiana tabacum | 302 | - | 9 | GTTTTCTTAC | cis-acting element involved in defense and stress responsiveness |
| TC-rich repeats | Nicotiana tabacum | 1753 | - | 9 | ATTCTCTAAC | cis-acting element involved in defense and stress responsiveness |

>PlantCARE\_20562   
+ TATTTTAGTT AGTTTTATGG AAGAATATTA AAATCATTTT TCATTTACAT GTCAATCTAT ATAATATGGT   
  
  
+ TAAATAATTG TATAATTTAT GCTATCTCAC ACGAAAATAC TTAACCTGTT TTATGAGATG ATATAGTAAA   
  
  
+ ATTTGAAATT CAATTTGTCA AATTTAGTTT TCATTGAGTT TTTTCGCTGG AAAAATATAT ACCTAATTTG   
  
  
+ AAGTCCCTAT AAATTAATCT GATAAGATTA TTTTACCAAA ACTGCTTAAA TTTTCATATT AAGTTGTTTC   
  
  
+ TGACTTTCTG ACGTTTCTGT GGTAAGAAAT CTTGTCTAAT GCCTTGTTTT ACAATCACAA TAGCCTATGG   
  
  
+ TTATCAACTT TTATAATAGT TTGAAGTAAA ACTTTTTTTT TGTTTGTTTT TAGAACTTTG ATGACCAGAT   
  
  
+ GGTTTGACTT TTTGTTGCAT ATTGATTAAA TTTCACATGT AAATGCTGAC TAACATTTAA AATTAAGGTT   
  
  
+ TGGCATATGA TGATGCCCAT TTCTGATGGC AGTTTGGTAT ATAATTTTTT TCAATTCAGT AAAAGCAAAT   
  
  
+ ACCCCATTTT ATGCTATTTC ACATGACCTA ACCAATTTTC TGAAATGATA TACTAAAATC TGACATTTAA   
  
  
+ TTTATTTGAA CAAAGTTTGT TATTATTTCC ATTCTTATTA TAATATAAAG TATTACACTT ATTACTATTA   
  
  
+ TTACTACTAC AATAATTATT ATTACTATAG TTAGTATTAT TACTATTATT AATACTACTA CCACTATTGT   
  
  
+ TAATTTGTTA TTATTACTAT TATTATTATT ATTATTATTA TTATTATTAT TATTTTTACT ACTCTTATTA   
  
  
+ CTATTATTAT ATTACTACTA CAATAATTAT TATTACTATA GTTAGTATTA TTCTTTTTAC TATTATTATT   
  
  
+ AATTATTAAT ATTATTAGTT TTAGTATTTT TTTAAAATTA TTATTAGAGT AACACTAGTA CTATAAAATT   
  
  
+ GACATTCTTT TCTTCCCCCC TCTCAATCTC TCATTCTCTC TTTCTCTCAG CCCTCTCCGC TGCTCTCACC   
  
  
+ CCTCACACTC TCCTTCGAGG CTTATTCAGG AAATCCCATA TCTCCTTTGA GGCTTATTCT TGGATTTGTT   
  
  
+ GTTGAACAGT TCTCTATATA TGTGTGTGTG TGTATATATA TATATATATA TATATATATA TATTTTTTTT   
  
  
+ TTTTTTTTTT TTTTTTTTTT GTGTTTAGAA ATTAGATATT CAGATATTGA AAAGTTTACG TCAAGATTTT   
  
  
+ TCTTTTCCAG AAAATTAAAT ATTTTACCTG GAATCTTTTG AGTTCCCATG TCTAGTTAAC AATTGTGTGT   
  
  
+ ATTTTATTTC CTGAACAATT TTCAGTTGTT AACATGAATT TTGAATGTTT CTTTTTTATT GTCTCTAGGG   
  
  
+ GCATATGCAT ATTAGCAGAG TTGTTAACTT GTTAACTAGA AGCACATGTT TGTCATATCT TCTTTGTGTT   
  
  
+ AGCTTTTTTA ATGGTTAATT GTCATTCTGC TGTGCTATAT TCTATATGTG CTTTGAATGC GTGTAGCTAA   
  
  
+ AATAATCCTA AAACTGAATT ATTTGAACTT CTAGACAAAT ACAACTCGAT GCCCAGGTAA CCTCCAAAGT   
  
  
+ TTTTTAAATA ACTGAACTAT CTTCAATATA ATACCATCAT GACAGTAAGG TTAGCTTGCT GAGTTTATTA   
  
  
+ AATATGTTAA CAGTTTCCCC AGTTGTCTTG TTTGTTTCTC ACTTGTGCCA TTAGGAGGCT GGGGTTTGGA   
  
  
+ ACGTTAGAGA AGAAAGAAAA AGGGAAAAAG AGGGAGAGAA AAAGAATAGA TATATTATAG TGATGACTGA   
  
  
+ TGAGTACTTG AGCACTTGAC TACCTGATTG TTTTTATTTT TTTCAAAATG TAAACTTCTA TTTATTTATT   
  
  
+ TAGGTAAAAC TTGATAATGG CATATTATTG AACCATGCTC TGGCACTTTG CACTTTGCAG ATCCTGTGCA   
  
  
+ TTTCAGTAGT CTGTATAATT CCTACTTGTT TTAAAAAA  

- ATAAAATCAA TCAAAATACC TTCTTATAAT TTTAGTAAAA AGTAAATGTA CAGTTAGATA TATTATACCA   
  
  
- ATTTATTAAC ATATTAAATA CGATAGAGTG TGCTTTTATG AATTGGACAA AATACTCTAC TATATCATTT   
  
  
- TAAACTTTAA GTTAAACAGT TTAAATCAAA AGTAACTCAA AAAAGCGACC TTTTTATATA TGGATTAAAC   
  
  
- TTCAGGGATA TTTAATTAGA CTATTCTAAT AAAATGGTTT TGACGAATTT AAAAGTATAA TTCAACAAAG   
  
  
- ACTGAAAGAC TGCAAAGACA CCATTCTTTA GAACAGATTA CGGAACAAAA TGTTAGTGTT ATCGGATACC   
  
  
- AATAGTTGAA AATATTATCA AACTTCATTT TGAAAAAAAA ACAAACAAAA ATCTTGAAAC TACTGGTCTA   
  
  
- CCAAACTGAA AAACAACGTA TAACTAATTT AAAGTGTACA TTTACGACTG ATTGTAAATT TTAATTCCAA   
  
  
- ACCGTATACT ACTACGGGTA AAGACTACCG TCAAACCATA TATTAAAAAA AGTTAAGTCA TTTTCGTTTA   
  
  
- TGGGGTAAAA TACGATAAAG TGTACTGGAT TGGTTAAAAG ACTTTACTAT ATGATTTTAG ACTGTAAATT   
  
  
- AAATAAACTT GTTTCAAACA ATAATAAAGG TAAGAATAAT ATTATATTTC ATAATGTGAA TAATGATAAT   
  
  
- AATGATGATG TTATTAATAA TAATGATATC AATCATAATA ATGATAATAA TTATGATGAT GGTGATAACA   
  
  
- ATTAAACAAT AATAATGATA ATAATAATAA TAATAATAAT AATAATAATA ATAAAAATGA TGAGAATAAT   
  
  
- GATAATAATA TAATGATGAT GTTATTAATA ATAATGATAT CAATCATAAT AAGAAAAATG ATAATAATAA   
  
  
- TTAATAATTA TAATAATCAA AATCATAAAA AAATTTTAAT AATAATCTCA TTGTGATCAT GATATTTTAA   
  
  
- CTGTAAGAAA AGAAGGGGGG AGAGTTAGAG AGTAAGAGAG AAAGAGAGTC GGGAGAGGCG ACGAGAGTGG   
  
  
- GGAGTGTGAG AGGAAGCTCC GAATAAGTCC TTTAGGGTAT AGAGGAAACT CCGAATAAGA ACCTAAACAA   
  
  
- CAACTTGTCA AGAGATATAT ACACACACAC ACATATATAT ATATATATAT ATATATATAT ATAAAAAAAA   
  
  
- AAAAAAAAAA AAAAAAAAAA CACAAATCTT TAATCTATAA GTCTATAACT TTTCAAATGC AGTTCTAAAA   
  
  
- AGAAAAGGTC TTTTAATTTA TAAAATGGAC CTTAGAAAAC TCAAGGGTAC AGATCAATTG TTAACACACA   
  
  
- TAAAATAAAG GACTTGTTAA AAGTCAACAA TTGTACTTAA AACTTACAAA GAAAAAATAA CAGAGATCCC   
  
  
- CGTATACGTA TAATCGTCTC AACAATTGAA CAATTGATCT TCGTGTACAA ACAGTATAGA AGAAACACAA   
  
  
- TCGAAAAAAT TACCAATTAA CAGTAAGACG ACACGATATA AGATATACAC GAAACTTACG CACATCGATT   
  
  
- TTATTAGGAT TTTGACTTAA TAAACTTGAA GATCTGTTTA TGTTGAGCTA CGGGTCCATT GGAGGTTTCA   
  
  
- AAAAATTTAT TGACTTGATA GAAGTTATAT TATGGTAGTA CTGTCATTCC AATCGAACGA CTCAAATAAT   
  
  
- TTATACAATT GTCAAAGGGG TCAACAGAAC AAACAAAGAG TGAACACGGT AATCCTCCGA CCCCAAACCT   
  
  
- TGCAATCTCT TCTTTCTTTT TCCCTTTTTC TCCCTCTCTT TTTCTTATCT ATATAATATC ACTACTGACT   
  
  
- ACTCATGAAC TCGTGAACTG ATGGACTAAC AAAAATAAAA AAAGTTTTAC ATTTGAAGAT AAATAAATAA   
  
  
- ATCCATTTTG AACTATTACC GTATAATAAC TTGGTACGAG ACCGTGAAAC GTGAAACGTC TAGGACACGT   
  
  
- AAAGTCATCA GACATATTAA GGATGAACAA AATTTTTT

+     TCA-element

| Site Name | Organism | Position | Strand | Matrix score. | sequence | function |
| --- | --- | --- | --- | --- | --- | --- |
| TCA-element | Nicotiana tabacum | 1775 | - | 9 | CCATCTTTTT | cis-acting element involved in salicylic acid responsiveness |

>PlantCARE\_20562   
+ TATTTTAGTT AGTTTTATGG AAGAATATTA AAATCATTTT TCATTTACAT GTCAATCTAT ATAATATGGT   
  
  
+ TAAATAATTG TATAATTTAT GCTATCTCAC ACGAAAATAC TTAACCTGTT TTATGAGATG ATATAGTAAA   
  
  
+ ATTTGAAATT CAATTTGTCA AATTTAGTTT TCATTGAGTT TTTTCGCTGG AAAAATATAT ACCTAATTTG   
  
  
+ AAGTCCCTAT AAATTAATCT GATAAGATTA TTTTACCAAA ACTGCTTAAA TTTTCATATT AAGTTGTTTC   
  
  
+ TGACTTTCTG ACGTTTCTGT GGTAAGAAAT CTTGTCTAAT GCCTTGTTTT ACAATCACAA TAGCCTATGG   
  
  
+ TTATCAACTT TTATAATAGT TTGAAGTAAA ACTTTTTTTT TGTTTGTTTT TAGAACTTTG ATGACCAGAT   
  
  
+ GGTTTGACTT TTTGTTGCAT ATTGATTAAA TTTCACATGT AAATGCTGAC TAACATTTAA AATTAAGGTT   
  
  
+ TGGCATATGA TGATGCCCAT TTCTGATGGC AGTTTGGTAT ATAATTTTTT TCAATTCAGT AAAAGCAAAT   
  
  
+ ACCCCATTTT ATGCTATTTC ACATGACCTA ACCAATTTTC TGAAATGATA TACTAAAATC TGACATTTAA   
  
  
+ TTTATTTGAA CAAAGTTTGT TATTATTTCC ATTCTTATTA TAATATAAAG TATTACACTT ATTACTATTA   
  
  
+ TTACTACTAC AATAATTATT ATTACTATAG TTAGTATTAT TACTATTATT AATACTACTA CCACTATTGT   
  
  
+ TAATTTGTTA TTATTACTAT TATTATTATT ATTATTATTA TTATTATTAT TATTTTTACT ACTCTTATTA   
  
  
+ CTATTATTAT ATTACTACTA CAATAATTAT TATTACTATA GTTAGTATTA TTCTTTTTAC TATTATTATT   
  
  
+ AATTATTAAT ATTATTAGTT TTAGTATTTT TTTAAAATTA TTATTAGAGT AACACTAGTA CTATAAAATT   
  
  
+ GACATTCTTT TCTTCCCCCC TCTCAATCTC TCATTCTCTC TTTCTCTCAG CCCTCTCCGC TGCTCTCACC   
  
  
+ CCTCACACTC TCCTTCGAGG CTTATTCAGG AAATCCCATA TCTCCTTTGA GGCTTATTCT TGGATTTGTT   
  
  
+ GTTGAACAGT TCTCTATATA TGTGTGTGTG TGTATATATA TATATATATA TATATATATA TATTTTTTTT   
  
  
+ TTTTTTTTTT TTTTTTTTTT GTGTTTAGAA ATTAGATATT CAGATATTGA AAAGTTTACG TCAAGATTTT   
  
  
+ TCTTTTCCAG AAAATTAAAT ATTTTACCTG GAATCTTTTG AGTTCCCATG TCTAGTTAAC AATTGTGTGT   
  
  
+ ATTTTATTTC CTGAACAATT TTCAGTTGTT AACATGAATT TTGAATGTTT CTTTTTTATT GTCTCTAGGG   
  
  
+ GCATATGCAT ATTAGCAGAG TTGTTAACTT GTTAACTAGA AGCACATGTT TGTCATATCT TCTTTGTGTT   
  
  
+ AGCTTTTTTA ATGGTTAATT GTCATTCTGC TGTGCTATAT TCTATATGTG CTTTGAATGC GTGTAGCTAA   
  
  
+ AATAATCCTA AAACTGAATT ATTTGAACTT CTAGACAAAT ACAACTCGAT GCCCAGGTAA CCTCCAAAGT   
  
  
+ TTTTTAAATA ACTGAACTAT CTTCAATATA ATACCATCAT GACAGTAAGG TTAGCTTGCT GAGTTTATTA   
  
  
+ AATATGTTAA CAGTTTCCCC AGTTGTCTTG TTTGTTTCTC ACTTGTGCCA TTAGGAGGCT GGGGTTTGGA   
  
  
+ ACGTTAGAGA AGAAAGAAAA AGGGAAAAAG AGGGAGAGAA AAAGAATAGA TATATTATAG TGATGACTGA   
  
  
+ TGAGTACTTG AGCACTTGAC TACCTGATTG TTTTTATTTT TTTCAAAATG TAAACTTCTA TTTATTTATT   
  
  
+ TAGGTAAAAC TTGATAATGG CATATTATTG AACCATGCTC TGGCACTTTG CACTTTGCAG ATCCTGTGCA   
  
  
+ TTTCAGTAGT CTGTATAATT CCTACTTGTT TTAAAAAA  

- ATAAAATCAA TCAAAATACC TTCTTATAAT TTTAGTAAAA AGTAAATGTA CAGTTAGATA TATTATACCA   
  
  
- ATTTATTAAC ATATTAAATA CGATAGAGTG TGCTTTTATG AATTGGACAA AATACTCTAC TATATCATTT   
  
  
- TAAACTTTAA GTTAAACAGT TTAAATCAAA AGTAACTCAA AAAAGCGACC TTTTTATATA TGGATTAAAC   
  
  
- TTCAGGGATA TTTAATTAGA CTATTCTAAT AAAATGGTTT TGACGAATTT AAAAGTATAA TTCAACAAAG   
  
  
- ACTGAAAGAC TGCAAAGACA CCATTCTTTA GAACAGATTA CGGAACAAAA TGTTAGTGTT ATCGGATACC   
  
  
- AATAGTTGAA AATATTATCA AACTTCATTT TGAAAAAAAA ACAAACAAAA ATCTTGAAAC TACTGGTCTA   
  
  
- CCAAACTGAA AAACAACGTA TAACTAATTT AAAGTGTACA TTTACGACTG ATTGTAAATT TTAATTCCAA   
  
  
- ACCGTATACT ACTACGGGTA AAGACTACCG TCAAACCATA TATTAAAAAA AGTTAAGTCA TTTTCGTTTA   
  
  
- TGGGGTAAAA TACGATAAAG TGTACTGGAT TGGTTAAAAG ACTTTACTAT ATGATTTTAG ACTGTAAATT   
  
  
- AAATAAACTT GTTTCAAACA ATAATAAAGG TAAGAATAAT ATTATATTTC ATAATGTGAA TAATGATAAT   
  
  
- AATGATGATG TTATTAATAA TAATGATATC AATCATAATA ATGATAATAA TTATGATGAT GGTGATAACA   
  
  
- ATTAAACAAT AATAATGATA ATAATAATAA TAATAATAAT AATAATAATA ATAAAAATGA TGAGAATAAT   
  
  
- GATAATAATA TAATGATGAT GTTATTAATA ATAATGATAT CAATCATAAT AAGAAAAATG ATAATAATAA   
  
  
- TTAATAATTA TAATAATCAA AATCATAAAA AAATTTTAAT AATAATCTCA TTGTGATCAT GATATTTTAA   
  
  
- CTGTAAGAAA AGAAGGGGGG AGAGTTAGAG AGTAAGAGAG AAAGAGAGTC GGGAGAGGCG ACGAGAGTGG   
  
  
- GGAGTGTGAG AGGAAGCTCC GAATAAGTCC TTTAGGGTAT AGAGGAAACT CCGAATAAGA ACCTAAACAA   
  
  
- CAACTTGTCA AGAGATATAT ACACACACAC ACATATATAT ATATATATAT ATATATATAT ATAAAAAAAA   
  
  
- AAAAAAAAAA AAAAAAAAAA CACAAATCTT TAATCTATAA GTCTATAACT TTTCAAATGC AGTTCTAAAA   
  
  
- AGAAAAGGTC TTTTAATTTA TAAAATGGAC CTTAGAAAAC TCAAGGGTAC AGATCAATTG TTAACACACA   
  
  
- TAAAATAAAG GACTTGTTAA AAGTCAACAA TTGTACTTAA AACTTACAAA GAAAAAATAA CAGAGATCCC   
  
  
- CGTATACGTA TAATCGTCTC AACAATTGAA CAATTGATCT TCGTGTACAA ACAGTATAGA AGAAACACAA   
  
  
- TCGAAAAAAT TACCAATTAA CAGTAAGACG ACACGATATA AGATATACAC GAAACTTACG CACATCGATT   
  
  
- TTATTAGGAT TTTGACTTAA TAAACTTGAA GATCTGTTTA TGTTGAGCTA CGGGTCCATT GGAGGTTTCA   
  
  
- AAAAATTTAT TGACTTGATA GAAGTTATAT TATGGTAGTA CTGTCATTCC AATCGAACGA CTCAAATAAT   
  
  
- TTATACAATT GTCAAAGGGG TCAACAGAAC AAACAAAGAG TGAACACGGT AATCCTCCGA CCCCAAACCT   
  
  
- TGCAATCTCT TCTTTCTTTT TCCCTTTTTC TCCCTCTCTT TTTCTTATCT ATATAATATC ACTACTGACT   
  
  
- ACTCATGAAC TCGTGAACTG ATGGACTAAC AAAAATAAAA AAAGTTTTAC ATTTGAAGAT AAATAAATAA   
  
  
- ATCCATTTTG AACTATTACC GTATAATAAC TTGGTACGAG ACCGTGAAAC GTGAAACGTC TAGGACACGT   
  
  
- AAAGTCATCA GACATATTAA GGATGAACAA AATTTTTT

+     TCCC-motif

| Site Name | Organism | Position | Strand | Matrix score. | sequence | function |
| --- | --- | --- | --- | --- | --- | --- |
| TCCC-motif | Spinacia oleracea | 1781 | - | 7 | TCTCCCT | part of a light responsive element |

>PlantCARE\_20562   
+ TATTTTAGTT AGTTTTATGG AAGAATATTA AAATCATTTT TCATTTACAT GTCAATCTAT ATAATATGGT   
  
  
+ TAAATAATTG TATAATTTAT GCTATCTCAC ACGAAAATAC TTAACCTGTT TTATGAGATG ATATAGTAAA   
  
  
+ ATTTGAAATT CAATTTGTCA AATTTAGTTT TCATTGAGTT TTTTCGCTGG AAAAATATAT ACCTAATTTG   
  
  
+ AAGTCCCTAT AAATTAATCT GATAAGATTA TTTTACCAAA ACTGCTTAAA TTTTCATATT AAGTTGTTTC   
  
  
+ TGACTTTCTG ACGTTTCTGT GGTAAGAAAT CTTGTCTAAT GCCTTGTTTT ACAATCACAA TAGCCTATGG   
  
  
+ TTATCAACTT TTATAATAGT TTGAAGTAAA ACTTTTTTTT TGTTTGTTTT TAGAACTTTG ATGACCAGAT   
  
  
+ GGTTTGACTT TTTGTTGCAT ATTGATTAAA TTTCACATGT AAATGCTGAC TAACATTTAA AATTAAGGTT   
  
  
+ TGGCATATGA TGATGCCCAT TTCTGATGGC AGTTTGGTAT ATAATTTTTT TCAATTCAGT AAAAGCAAAT   
  
  
+ ACCCCATTTT ATGCTATTTC ACATGACCTA ACCAATTTTC TGAAATGATA TACTAAAATC TGACATTTAA   
  
  
+ TTTATTTGAA CAAAGTTTGT TATTATTTCC ATTCTTATTA TAATATAAAG TATTACACTT ATTACTATTA   
  
  
+ TTACTACTAC AATAATTATT ATTACTATAG TTAGTATTAT TACTATTATT AATACTACTA CCACTATTGT   
  
  
+ TAATTTGTTA TTATTACTAT TATTATTATT ATTATTATTA TTATTATTAT TATTTTTACT ACTCTTATTA   
  
  
+ CTATTATTAT ATTACTACTA CAATAATTAT TATTACTATA GTTAGTATTA TTCTTTTTAC TATTATTATT   
  
  
+ AATTATTAAT ATTATTAGTT TTAGTATTTT TTTAAAATTA TTATTAGAGT AACACTAGTA CTATAAAATT   
  
  
+ GACATTCTTT TCTTCCCCCC TCTCAATCTC TCATTCTCTC TTTCTCTCAG CCCTCTCCGC TGCTCTCACC   
  
  
+ CCTCACACTC TCCTTCGAGG CTTATTCAGG AAATCCCATA TCTCCTTTGA GGCTTATTCT TGGATTTGTT   
  
  
+ GTTGAACAGT TCTCTATATA TGTGTGTGTG TGTATATATA TATATATATA TATATATATA TATTTTTTTT   
  
  
+ TTTTTTTTTT TTTTTTTTTT GTGTTTAGAA ATTAGATATT CAGATATTGA AAAGTTTACG TCAAGATTTT   
  
  
+ TCTTTTCCAG AAAATTAAAT ATTTTACCTG GAATCTTTTG AGTTCCCATG TCTAGTTAAC AATTGTGTGT   
  
  
+ ATTTTATTTC CTGAACAATT TTCAGTTGTT AACATGAATT TTGAATGTTT CTTTTTTATT GTCTCTAGGG   
  
  
+ GCATATGCAT ATTAGCAGAG TTGTTAACTT GTTAACTAGA AGCACATGTT TGTCATATCT TCTTTGTGTT   
  
  
+ AGCTTTTTTA ATGGTTAATT GTCATTCTGC TGTGCTATAT TCTATATGTG CTTTGAATGC GTGTAGCTAA   
  
  
+ AATAATCCTA AAACTGAATT ATTTGAACTT CTAGACAAAT ACAACTCGAT GCCCAGGTAA CCTCCAAAGT   
  
  
+ TTTTTAAATA ACTGAACTAT CTTCAATATA ATACCATCAT GACAGTAAGG TTAGCTTGCT GAGTTTATTA   
  
  
+ AATATGTTAA CAGTTTCCCC AGTTGTCTTG TTTGTTTCTC ACTTGTGCCA TTAGGAGGCT GGGGTTTGGA   
  
  
+ ACGTTAGAGA AGAAAGAAAA AGGGAAAAAG AGGGAGAGAA AAAGAATAGA TATATTATAG TGATGACTGA   
  
  
+ TGAGTACTTG AGCACTTGAC TACCTGATTG TTTTTATTTT TTTCAAAATG TAAACTTCTA TTTATTTATT   
  
  
+ TAGGTAAAAC TTGATAATGG CATATTATTG AACCATGCTC TGGCACTTTG CACTTTGCAG ATCCTGTGCA   
  
  
+ TTTCAGTAGT CTGTATAATT CCTACTTGTT TTAAAAAA  

- ATAAAATCAA TCAAAATACC TTCTTATAAT TTTAGTAAAA AGTAAATGTA CAGTTAGATA TATTATACCA   
  
  
- ATTTATTAAC ATATTAAATA CGATAGAGTG TGCTTTTATG AATTGGACAA AATACTCTAC TATATCATTT   
  
  
- TAAACTTTAA GTTAAACAGT TTAAATCAAA AGTAACTCAA AAAAGCGACC TTTTTATATA TGGATTAAAC   
  
  
- TTCAGGGATA TTTAATTAGA CTATTCTAAT AAAATGGTTT TGACGAATTT AAAAGTATAA TTCAACAAAG   
  
  
- ACTGAAAGAC TGCAAAGACA CCATTCTTTA GAACAGATTA CGGAACAAAA TGTTAGTGTT ATCGGATACC   
  
  
- AATAGTTGAA AATATTATCA AACTTCATTT TGAAAAAAAA ACAAACAAAA ATCTTGAAAC TACTGGTCTA   
  
  
- CCAAACTGAA AAACAACGTA TAACTAATTT AAAGTGTACA TTTACGACTG ATTGTAAATT TTAATTCCAA   
  
  
- ACCGTATACT ACTACGGGTA AAGACTACCG TCAAACCATA TATTAAAAAA AGTTAAGTCA TTTTCGTTTA   
  
  
- TGGGGTAAAA TACGATAAAG TGTACTGGAT TGGTTAAAAG ACTTTACTAT ATGATTTTAG ACTGTAAATT   
  
  
- AAATAAACTT GTTTCAAACA ATAATAAAGG TAAGAATAAT ATTATATTTC ATAATGTGAA TAATGATAAT   
  
  
- AATGATGATG TTATTAATAA TAATGATATC AATCATAATA ATGATAATAA TTATGATGAT GGTGATAACA   
  
  
- ATTAAACAAT AATAATGATA ATAATAATAA TAATAATAAT AATAATAATA ATAAAAATGA TGAGAATAAT   
  
  
- GATAATAATA TAATGATGAT GTTATTAATA ATAATGATAT CAATCATAAT AAGAAAAATG ATAATAATAA   
  
  
- TTAATAATTA TAATAATCAA AATCATAAAA AAATTTTAAT AATAATCTCA TTGTGATCAT GATATTTTAA   
  
  
- CTGTAAGAAA AGAAGGGGGG AGAGTTAGAG AGTAAGAGAG AAAGAGAGTC GGGAGAGGCG ACGAGAGTGG   
  
  
- GGAGTGTGAG AGGAAGCTCC GAATAAGTCC TTTAGGGTAT AGAGGAAACT CCGAATAAGA ACCTAAACAA   
  
  
- CAACTTGTCA AGAGATATAT ACACACACAC ACATATATAT ATATATATAT ATATATATAT ATAAAAAAAA   
  
  
- AAAAAAAAAA AAAAAAAAAA CACAAATCTT TAATCTATAA GTCTATAACT TTTCAAATGC AGTTCTAAAA   
  
  
- AGAAAAGGTC TTTTAATTTA TAAAATGGAC CTTAGAAAAC TCAAGGGTAC AGATCAATTG TTAACACACA   
  
  
- TAAAATAAAG GACTTGTTAA AAGTCAACAA TTGTACTTAA AACTTACAAA GAAAAAATAA CAGAGATCCC   
  
  
- CGTATACGTA TAATCGTCTC AACAATTGAA CAATTGATCT TCGTGTACAA ACAGTATAGA AGAAACACAA   
  
  
- TCGAAAAAAT TACCAATTAA CAGTAAGACG ACACGATATA AGATATACAC GAAACTTACG CACATCGATT   
  
  
- TTATTAGGAT TTTGACTTAA TAAACTTGAA GATCTGTTTA TGTTGAGCTA CGGGTCCATT GGAGGTTTCA   
  
  
- AAAAATTTAT TGACTTGATA GAAGTTATAT TATGGTAGTA CTGTCATTCC AATCGAACGA CTCAAATAAT   
  
  
- TTATACAATT GTCAAAGGGG TCAACAGAAC AAACAAAGAG TGAACACGGT AATCCTCCGA CCCCAAACCT   
  
  
- TGCAATCTCT TCTTTCTTTT TCCCTTTTTC TCCCTCTCTT TTTCTTATCT ATATAATATC ACTACTGACT   
  
  
- ACTCATGAAC TCGTGAACTG ATGGACTAAC AAAAATAAAA AAAGTTTTAC ATTTGAAGAT AAATAAATAA   
  
  
- ATCCATTTTG AACTATTACC GTATAATAAC TTGGTACGAG ACCGTGAAAC GTGAAACGTC TAGGACACGT   
  
  
- AAAGTCATCA GACATATTAA GGATGAACAA AATTTTTT

+     TCT-motif

| Site Name | Organism | Position | Strand | Matrix score. | sequence | function |
| --- | --- | --- | --- | --- | --- | --- |
| TCT-motif | Arabidopsis thaliana | 302 | - | 6 | TCTTAC | part of a light responsive element |

>PlantCARE\_20562   
+ TATTTTAGTT AGTTTTATGG AAGAATATTA AAATCATTTT TCATTTACAT GTCAATCTAT ATAATATGGT   
  
  
+ TAAATAATTG TATAATTTAT GCTATCTCAC ACGAAAATAC TTAACCTGTT TTATGAGATG ATATAGTAAA   
  
  
+ ATTTGAAATT CAATTTGTCA AATTTAGTTT TCATTGAGTT TTTTCGCTGG AAAAATATAT ACCTAATTTG   
  
  
+ AAGTCCCTAT AAATTAATCT GATAAGATTA TTTTACCAAA ACTGCTTAAA TTTTCATATT AAGTTGTTTC   
  
  
+ TGACTTTCTG ACGTTTCTGT GGTAAGAAAT CTTGTCTAAT GCCTTGTTTT ACAATCACAA TAGCCTATGG   
  
  
+ TTATCAACTT TTATAATAGT TTGAAGTAAA ACTTTTTTTT TGTTTGTTTT TAGAACTTTG ATGACCAGAT   
  
  
+ GGTTTGACTT TTTGTTGCAT ATTGATTAAA TTTCACATGT AAATGCTGAC TAACATTTAA AATTAAGGTT   
  
  
+ TGGCATATGA TGATGCCCAT TTCTGATGGC AGTTTGGTAT ATAATTTTTT TCAATTCAGT AAAAGCAAAT   
  
  
+ ACCCCATTTT ATGCTATTTC ACATGACCTA ACCAATTTTC TGAAATGATA TACTAAAATC TGACATTTAA   
  
  
+ TTTATTTGAA CAAAGTTTGT TATTATTTCC ATTCTTATTA TAATATAAAG TATTACACTT ATTACTATTA   
  
  
+ TTACTACTAC AATAATTATT ATTACTATAG TTAGTATTAT TACTATTATT AATACTACTA CCACTATTGT   
  
  
+ TAATTTGTTA TTATTACTAT TATTATTATT ATTATTATTA TTATTATTAT TATTTTTACT ACTCTTATTA   
  
  
+ CTATTATTAT ATTACTACTA CAATAATTAT TATTACTATA GTTAGTATTA TTCTTTTTAC TATTATTATT   
  
  
+ AATTATTAAT ATTATTAGTT TTAGTATTTT TTTAAAATTA TTATTAGAGT AACACTAGTA CTATAAAATT   
  
  
+ GACATTCTTT TCTTCCCCCC TCTCAATCTC TCATTCTCTC TTTCTCTCAG CCCTCTCCGC TGCTCTCACC   
  
  
+ CCTCACACTC TCCTTCGAGG CTTATTCAGG AAATCCCATA TCTCCTTTGA GGCTTATTCT TGGATTTGTT   
  
  
+ GTTGAACAGT TCTCTATATA TGTGTGTGTG TGTATATATA TATATATATA TATATATATA TATTTTTTTT   
  
  
+ TTTTTTTTTT TTTTTTTTTT GTGTTTAGAA ATTAGATATT CAGATATTGA AAAGTTTACG TCAAGATTTT   
  
  
+ TCTTTTCCAG AAAATTAAAT ATTTTACCTG GAATCTTTTG AGTTCCCATG TCTAGTTAAC AATTGTGTGT   
  
  
+ ATTTTATTTC CTGAACAATT TTCAGTTGTT AACATGAATT TTGAATGTTT CTTTTTTATT GTCTCTAGGG   
  
  
+ GCATATGCAT ATTAGCAGAG TTGTTAACTT GTTAACTAGA AGCACATGTT TGTCATATCT TCTTTGTGTT   
  
  
+ AGCTTTTTTA ATGGTTAATT GTCATTCTGC TGTGCTATAT TCTATATGTG CTTTGAATGC GTGTAGCTAA   
  
  
+ AATAATCCTA AAACTGAATT ATTTGAACTT CTAGACAAAT ACAACTCGAT GCCCAGGTAA CCTCCAAAGT   
  
  
+ TTTTTAAATA ACTGAACTAT CTTCAATATA ATACCATCAT GACAGTAAGG TTAGCTTGCT GAGTTTATTA   
  
  
+ AATATGTTAA CAGTTTCCCC AGTTGTCTTG TTTGTTTCTC ACTTGTGCCA TTAGGAGGCT GGGGTTTGGA   
  
  
+ ACGTTAGAGA AGAAAGAAAA AGGGAAAAAG AGGGAGAGAA AAAGAATAGA TATATTATAG TGATGACTGA   
  
  
+ TGAGTACTTG AGCACTTGAC TACCTGATTG TTTTTATTTT TTTCAAAATG TAAACTTCTA TTTATTTATT   
  
  
+ TAGGTAAAAC TTGATAATGG CATATTATTG AACCATGCTC TGGCACTTTG CACTTTGCAG ATCCTGTGCA   
  
  
+ TTTCAGTAGT CTGTATAATT CCTACTTGTT TTAAAAAA  

- ATAAAATCAA TCAAAATACC TTCTTATAAT TTTAGTAAAA AGTAAATGTA CAGTTAGATA TATTATACCA   
  
  
- ATTTATTAAC ATATTAAATA CGATAGAGTG TGCTTTTATG AATTGGACAA AATACTCTAC TATATCATTT   
  
  
- TAAACTTTAA GTTAAACAGT TTAAATCAAA AGTAACTCAA AAAAGCGACC TTTTTATATA TGGATTAAAC   
  
  
- TTCAGGGATA TTTAATTAGA CTATTCTAAT AAAATGGTTT TGACGAATTT AAAAGTATAA TTCAACAAAG   
  
  
- ACTGAAAGAC TGCAAAGACA CCATTCTTTA GAACAGATTA CGGAACAAAA TGTTAGTGTT ATCGGATACC   
  
  
- AATAGTTGAA AATATTATCA AACTTCATTT TGAAAAAAAA ACAAACAAAA ATCTTGAAAC TACTGGTCTA   
  
  
- CCAAACTGAA AAACAACGTA TAACTAATTT AAAGTGTACA TTTACGACTG ATTGTAAATT TTAATTCCAA   
  
  
- ACCGTATACT ACTACGGGTA AAGACTACCG TCAAACCATA TATTAAAAAA AGTTAAGTCA TTTTCGTTTA   
  
  
- TGGGGTAAAA TACGATAAAG TGTACTGGAT TGGTTAAAAG ACTTTACTAT ATGATTTTAG ACTGTAAATT   
  
  
- AAATAAACTT GTTTCAAACA ATAATAAAGG TAAGAATAAT ATTATATTTC ATAATGTGAA TAATGATAAT   
  
  
- AATGATGATG TTATTAATAA TAATGATATC AATCATAATA ATGATAATAA TTATGATGAT GGTGATAACA   
  
  
- ATTAAACAAT AATAATGATA ATAATAATAA TAATAATAAT AATAATAATA ATAAAAATGA TGAGAATAAT   
  
  
- GATAATAATA TAATGATGAT GTTATTAATA ATAATGATAT CAATCATAAT AAGAAAAATG ATAATAATAA   
  
  
- TTAATAATTA TAATAATCAA AATCATAAAA AAATTTTAAT AATAATCTCA TTGTGATCAT GATATTTTAA   
  
  
- CTGTAAGAAA AGAAGGGGGG AGAGTTAGAG AGTAAGAGAG AAAGAGAGTC GGGAGAGGCG ACGAGAGTGG   
  
  
- GGAGTGTGAG AGGAAGCTCC GAATAAGTCC TTTAGGGTAT AGAGGAAACT CCGAATAAGA ACCTAAACAA   
  
  
- CAACTTGTCA AGAGATATAT ACACACACAC ACATATATAT ATATATATAT ATATATATAT ATAAAAAAAA   
  
  
- AAAAAAAAAA AAAAAAAAAA CACAAATCTT TAATCTATAA GTCTATAACT TTTCAAATGC AGTTCTAAAA   
  
  
- AGAAAAGGTC TTTTAATTTA TAAAATGGAC CTTAGAAAAC TCAAGGGTAC AGATCAATTG TTAACACACA   
  
  
- TAAAATAAAG GACTTGTTAA AAGTCAACAA TTGTACTTAA AACTTACAAA GAAAAAATAA CAGAGATCCC   
  
  
- CGTATACGTA TAATCGTCTC AACAATTGAA CAATTGATCT TCGTGTACAA ACAGTATAGA AGAAACACAA   
  
  
- TCGAAAAAAT TACCAATTAA CAGTAAGACG ACACGATATA AGATATACAC GAAACTTACG CACATCGATT   
  
  
- TTATTAGGAT TTTGACTTAA TAAACTTGAA GATCTGTTTA TGTTGAGCTA CGGGTCCATT GGAGGTTTCA   
  
  
- AAAAATTTAT TGACTTGATA GAAGTTATAT TATGGTAGTA CTGTCATTCC AATCGAACGA CTCAAATAAT   
  
  
- TTATACAATT GTCAAAGGGG TCAACAGAAC AAACAAAGAG TGAACACGGT AATCCTCCGA CCCCAAACCT   
  
  
- TGCAATCTCT TCTTTCTTTT TCCCTTTTTC TCCCTCTCTT TTTCTTATCT ATATAATATC ACTACTGACT   
  
  
- ACTCATGAAC TCGTGAACTG ATGGACTAAC AAAAATAAAA AAAGTTTTAC ATTTGAAGAT AAATAAATAA   
  
  
- ATCCATTTTG AACTATTACC GTATAATAAC TTGGTACGAG ACCGTGAAAC GTGAAACGTC TAGGACACGT   
  
  
- AAAGTCATCA GACATATTAA GGATGAACAA AATTTTTT

+     TGA-box

| Site Name | Organism | Position | Strand | Matrix score. | sequence | function |
| --- | --- | --- | --- | --- | --- | --- |
| TGA-box | Glycine max | 1246 | - | 8 | TGACGTAA | part of an auxin-responsive element |

>PlantCARE\_20562   
+ TATTTTAGTT AGTTTTATGG AAGAATATTA AAATCATTTT TCATTTACAT GTCAATCTAT ATAATATGGT   
  
  
+ TAAATAATTG TATAATTTAT GCTATCTCAC ACGAAAATAC TTAACCTGTT TTATGAGATG ATATAGTAAA   
  
  
+ ATTTGAAATT CAATTTGTCA AATTTAGTTT TCATTGAGTT TTTTCGCTGG AAAAATATAT ACCTAATTTG   
  
  
+ AAGTCCCTAT AAATTAATCT GATAAGATTA TTTTACCAAA ACTGCTTAAA TTTTCATATT AAGTTGTTTC   
  
  
+ TGACTTTCTG ACGTTTCTGT GGTAAGAAAT CTTGTCTAAT GCCTTGTTTT ACAATCACAA TAGCCTATGG   
  
  
+ TTATCAACTT TTATAATAGT TTGAAGTAAA ACTTTTTTTT TGTTTGTTTT TAGAACTTTG ATGACCAGAT   
  
  
+ GGTTTGACTT TTTGTTGCAT ATTGATTAAA TTTCACATGT AAATGCTGAC TAACATTTAA AATTAAGGTT   
  
  
+ TGGCATATGA TGATGCCCAT TTCTGATGGC AGTTTGGTAT ATAATTTTTT TCAATTCAGT AAAAGCAAAT   
  
  
+ ACCCCATTTT ATGCTATTTC ACATGACCTA ACCAATTTTC TGAAATGATA TACTAAAATC TGACATTTAA   
  
  
+ TTTATTTGAA CAAAGTTTGT TATTATTTCC ATTCTTATTA TAATATAAAG TATTACACTT ATTACTATTA   
  
  
+ TTACTACTAC AATAATTATT ATTACTATAG TTAGTATTAT TACTATTATT AATACTACTA CCACTATTGT   
  
  
+ TAATTTGTTA TTATTACTAT TATTATTATT ATTATTATTA TTATTATTAT TATTTTTACT ACTCTTATTA   
  
  
+ CTATTATTAT ATTACTACTA CAATAATTAT TATTACTATA GTTAGTATTA TTCTTTTTAC TATTATTATT   
  
  
+ AATTATTAAT ATTATTAGTT TTAGTATTTT TTTAAAATTA TTATTAGAGT AACACTAGTA CTATAAAATT   
  
  
+ GACATTCTTT TCTTCCCCCC TCTCAATCTC TCATTCTCTC TTTCTCTCAG CCCTCTCCGC TGCTCTCACC   
  
  
+ CCTCACACTC TCCTTCGAGG CTTATTCAGG AAATCCCATA TCTCCTTTGA GGCTTATTCT TGGATTTGTT   
  
  
+ GTTGAACAGT TCTCTATATA TGTGTGTGTG TGTATATATA TATATATATA TATATATATA TATTTTTTTT   
  
  
+ TTTTTTTTTT TTTTTTTTTT GTGTTTAGAA ATTAGATATT CAGATATTGA AAAGTTTACG TCAAGATTTT   
  
  
+ TCTTTTCCAG AAAATTAAAT ATTTTACCTG GAATCTTTTG AGTTCCCATG TCTAGTTAAC AATTGTGTGT   
  
  
+ ATTTTATTTC CTGAACAATT TTCAGTTGTT AACATGAATT TTGAATGTTT CTTTTTTATT GTCTCTAGGG   
  
  
+ GCATATGCAT ATTAGCAGAG TTGTTAACTT GTTAACTAGA AGCACATGTT TGTCATATCT TCTTTGTGTT   
  
  
+ AGCTTTTTTA ATGGTTAATT GTCATTCTGC TGTGCTATAT TCTATATGTG CTTTGAATGC GTGTAGCTAA   
  
  
+ AATAATCCTA AAACTGAATT ATTTGAACTT CTAGACAAAT ACAACTCGAT GCCCAGGTAA CCTCCAAAGT   
  
  
+ TTTTTAAATA ACTGAACTAT CTTCAATATA ATACCATCAT GACAGTAAGG TTAGCTTGCT GAGTTTATTA   
  
  
+ AATATGTTAA CAGTTTCCCC AGTTGTCTTG TTTGTTTCTC ACTTGTGCCA TTAGGAGGCT GGGGTTTGGA   
  
  
+ ACGTTAGAGA AGAAAGAAAA AGGGAAAAAG AGGGAGAGAA AAAGAATAGA TATATTATAG TGATGACTGA   
  
  
+ TGAGTACTTG AGCACTTGAC TACCTGATTG TTTTTATTTT TTTCAAAATG TAAACTTCTA TTTATTTATT   
  
  
+ TAGGTAAAAC TTGATAATGG CATATTATTG AACCATGCTC TGGCACTTTG CACTTTGCAG ATCCTGTGCA   
  
  
+ TTTCAGTAGT CTGTATAATT CCTACTTGTT TTAAAAAA  

- ATAAAATCAA TCAAAATACC TTCTTATAAT TTTAGTAAAA AGTAAATGTA CAGTTAGATA TATTATACCA   
  
  
- ATTTATTAAC ATATTAAATA CGATAGAGTG TGCTTTTATG AATTGGACAA AATACTCTAC TATATCATTT   
  
  
- TAAACTTTAA GTTAAACAGT TTAAATCAAA AGTAACTCAA AAAAGCGACC TTTTTATATA TGGATTAAAC   
  
  
- TTCAGGGATA TTTAATTAGA CTATTCTAAT AAAATGGTTT TGACGAATTT AAAAGTATAA TTCAACAAAG   
  
  
- ACTGAAAGAC TGCAAAGACA CCATTCTTTA GAACAGATTA CGGAACAAAA TGTTAGTGTT ATCGGATACC   
  
  
- AATAGTTGAA AATATTATCA AACTTCATTT TGAAAAAAAA ACAAACAAAA ATCTTGAAAC TACTGGTCTA   
  
  
- CCAAACTGAA AAACAACGTA TAACTAATTT AAAGTGTACA TTTACGACTG ATTGTAAATT TTAATTCCAA   
  
  
- ACCGTATACT ACTACGGGTA AAGACTACCG TCAAACCATA TATTAAAAAA AGTTAAGTCA TTTTCGTTTA   
  
  
- TGGGGTAAAA TACGATAAAG TGTACTGGAT TGGTTAAAAG ACTTTACTAT ATGATTTTAG ACTGTAAATT   
  
  
- AAATAAACTT GTTTCAAACA ATAATAAAGG TAAGAATAAT ATTATATTTC ATAATGTGAA TAATGATAAT   
  
  
- AATGATGATG TTATTAATAA TAATGATATC AATCATAATA ATGATAATAA TTATGATGAT GGTGATAACA   
  
  
- ATTAAACAAT AATAATGATA ATAATAATAA TAATAATAAT AATAATAATA ATAAAAATGA TGAGAATAAT   
  
  
- GATAATAATA TAATGATGAT GTTATTAATA ATAATGATAT CAATCATAAT AAGAAAAATG ATAATAATAA   
  
  
- TTAATAATTA TAATAATCAA AATCATAAAA AAATTTTAAT AATAATCTCA TTGTGATCAT GATATTTTAA   
  
  
- CTGTAAGAAA AGAAGGGGGG AGAGTTAGAG AGTAAGAGAG AAAGAGAGTC GGGAGAGGCG ACGAGAGTGG   
  
  
- GGAGTGTGAG AGGAAGCTCC GAATAAGTCC TTTAGGGTAT AGAGGAAACT CCGAATAAGA ACCTAAACAA   
  
  
- CAACTTGTCA AGAGATATAT ACACACACAC ACATATATAT ATATATATAT ATATATATAT ATAAAAAAAA   
  
  
- AAAAAAAAAA AAAAAAAAAA CACAAATCTT TAATCTATAA GTCTATAACT TTTCAAATGC AGTTCTAAAA   
  
  
- AGAAAAGGTC TTTTAATTTA TAAAATGGAC CTTAGAAAAC TCAAGGGTAC AGATCAATTG TTAACACACA   
  
  
- TAAAATAAAG GACTTGTTAA AAGTCAACAA TTGTACTTAA AACTTACAAA GAAAAAATAA CAGAGATCCC   
  
  
- CGTATACGTA TAATCGTCTC AACAATTGAA CAATTGATCT TCGTGTACAA ACAGTATAGA AGAAACACAA   
  
  
- TCGAAAAAAT TACCAATTAA CAGTAAGACG ACACGATATA AGATATACAC GAAACTTACG CACATCGATT   
  
  
- TTATTAGGAT TTTGACTTAA TAAACTTGAA GATCTGTTTA TGTTGAGCTA CGGGTCCATT GGAGGTTTCA   
  
  
- AAAAATTTAT TGACTTGATA GAAGTTATAT TATGGTAGTA CTGTCATTCC AATCGAACGA CTCAAATAAT   
  
  
- TTATACAATT GTCAAAGGGG TCAACAGAAC AAACAAAGAG TGAACACGGT AATCCTCCGA CCCCAAACCT   
  
  
- TGCAATCTCT TCTTTCTTTT TCCCTTTTTC TCCCTCTCTT TTTCTTATCT ATATAATATC ACTACTGACT   
  
  
- ACTCATGAAC TCGTGAACTG ATGGACTAAC AAAAATAAAA AAAGTTTTAC ATTTGAAGAT AAATAAATAA   
  
  
- ATCCATTTTG AACTATTACC GTATAATAAC TTGGTACGAG ACCGTGAAAC GTGAAACGTC TAGGACACGT   
  
  
- AAAGTCATCA GACATATTAA GGATGAACAA AATTTTTT

+     TGACG-motif

| Site Name | Organism | Position | Strand | Matrix score. | sequence | function |
| --- | --- | --- | --- | --- | --- | --- |
| TGACG-motif | Hordeum vulgare | 289 | + | 5 | TGACG | cis-acting regulatory element involved in the MeJA-responsiveness |
| TGACG-motif | Hordeum vulgare | 1249 | - | 5 | TGACG | cis-acting regulatory element involved in the MeJA-responsiveness |

>PlantCARE\_20562   
+ TATTTTAGTT AGTTTTATGG AAGAATATTA AAATCATTTT TCATTTACAT GTCAATCTAT ATAATATGGT   
  
  
+ TAAATAATTG TATAATTTAT GCTATCTCAC ACGAAAATAC TTAACCTGTT TTATGAGATG ATATAGTAAA   
  
  
+ ATTTGAAATT CAATTTGTCA AATTTAGTTT TCATTGAGTT TTTTCGCTGG AAAAATATAT ACCTAATTTG   
  
  
+ AAGTCCCTAT AAATTAATCT GATAAGATTA TTTTACCAAA ACTGCTTAAA TTTTCATATT AAGTTGTTTC   
  
  
+ TGACTTTCTG ACGTTTCTGT GGTAAGAAAT CTTGTCTAAT GCCTTGTTTT ACAATCACAA TAGCCTATGG   
  
  
+ TTATCAACTT TTATAATAGT TTGAAGTAAA ACTTTTTTTT TGTTTGTTTT TAGAACTTTG ATGACCAGAT   
  
  
+ GGTTTGACTT TTTGTTGCAT ATTGATTAAA TTTCACATGT AAATGCTGAC TAACATTTAA AATTAAGGTT   
  
  
+ TGGCATATGA TGATGCCCAT TTCTGATGGC AGTTTGGTAT ATAATTTTTT TCAATTCAGT AAAAGCAAAT   
  
  
+ ACCCCATTTT ATGCTATTTC ACATGACCTA ACCAATTTTC TGAAATGATA TACTAAAATC TGACATTTAA   
  
  
+ TTTATTTGAA CAAAGTTTGT TATTATTTCC ATTCTTATTA TAATATAAAG TATTACACTT ATTACTATTA   
  
  
+ TTACTACTAC AATAATTATT ATTACTATAG TTAGTATTAT TACTATTATT AATACTACTA CCACTATTGT   
  
  
+ TAATTTGTTA TTATTACTAT TATTATTATT ATTATTATTA TTATTATTAT TATTTTTACT ACTCTTATTA   
  
  
+ CTATTATTAT ATTACTACTA CAATAATTAT TATTACTATA GTTAGTATTA TTCTTTTTAC TATTATTATT   
  
  
+ AATTATTAAT ATTATTAGTT TTAGTATTTT TTTAAAATTA TTATTAGAGT AACACTAGTA CTATAAAATT   
  
  
+ GACATTCTTT TCTTCCCCCC TCTCAATCTC TCATTCTCTC TTTCTCTCAG CCCTCTCCGC TGCTCTCACC   
  
  
+ CCTCACACTC TCCTTCGAGG CTTATTCAGG AAATCCCATA TCTCCTTTGA GGCTTATTCT TGGATTTGTT   
  
  
+ GTTGAACAGT TCTCTATATA TGTGTGTGTG TGTATATATA TATATATATA TATATATATA TATTTTTTTT   
  
  
+ TTTTTTTTTT TTTTTTTTTT GTGTTTAGAA ATTAGATATT CAGATATTGA AAAGTTTACG TCAAGATTTT   
  
  
+ TCTTTTCCAG AAAATTAAAT ATTTTACCTG GAATCTTTTG AGTTCCCATG TCTAGTTAAC AATTGTGTGT   
  
  
+ ATTTTATTTC CTGAACAATT TTCAGTTGTT AACATGAATT TTGAATGTTT CTTTTTTATT GTCTCTAGGG   
  
  
+ GCATATGCAT ATTAGCAGAG TTGTTAACTT GTTAACTAGA AGCACATGTT TGTCATATCT TCTTTGTGTT   
  
  
+ AGCTTTTTTA ATGGTTAATT GTCATTCTGC TGTGCTATAT TCTATATGTG CTTTGAATGC GTGTAGCTAA   
  
  
+ AATAATCCTA AAACTGAATT ATTTGAACTT CTAGACAAAT ACAACTCGAT GCCCAGGTAA CCTCCAAAGT   
  
  
+ TTTTTAAATA ACTGAACTAT CTTCAATATA ATACCATCAT GACAGTAAGG TTAGCTTGCT GAGTTTATTA   
  
  
+ AATATGTTAA CAGTTTCCCC AGTTGTCTTG TTTGTTTCTC ACTTGTGCCA TTAGGAGGCT GGGGTTTGGA   
  
  
+ ACGTTAGAGA AGAAAGAAAA AGGGAAAAAG AGGGAGAGAA AAAGAATAGA TATATTATAG TGATGACTGA   
  
  
+ TGAGTACTTG AGCACTTGAC TACCTGATTG TTTTTATTTT TTTCAAAATG TAAACTTCTA TTTATTTATT   
  
  
+ TAGGTAAAAC TTGATAATGG CATATTATTG AACCATGCTC TGGCACTTTG CACTTTGCAG ATCCTGTGCA   
  
  
+ TTTCAGTAGT CTGTATAATT CCTACTTGTT TTAAAAAA  

- ATAAAATCAA TCAAAATACC TTCTTATAAT TTTAGTAAAA AGTAAATGTA CAGTTAGATA TATTATACCA   
  
  
- ATTTATTAAC ATATTAAATA CGATAGAGTG TGCTTTTATG AATTGGACAA AATACTCTAC TATATCATTT   
  
  
- TAAACTTTAA GTTAAACAGT TTAAATCAAA AGTAACTCAA AAAAGCGACC TTTTTATATA TGGATTAAAC   
  
  
- TTCAGGGATA TTTAATTAGA CTATTCTAAT AAAATGGTTT TGACGAATTT AAAAGTATAA TTCAACAAAG   
  
  
- ACTGAAAGAC TGCAAAGACA CCATTCTTTA GAACAGATTA CGGAACAAAA TGTTAGTGTT ATCGGATACC   
  
  
- AATAGTTGAA AATATTATCA AACTTCATTT TGAAAAAAAA ACAAACAAAA ATCTTGAAAC TACTGGTCTA   
  
  
- CCAAACTGAA AAACAACGTA TAACTAATTT AAAGTGTACA TTTACGACTG ATTGTAAATT TTAATTCCAA   
  
  
- ACCGTATACT ACTACGGGTA AAGACTACCG TCAAACCATA TATTAAAAAA AGTTAAGTCA TTTTCGTTTA   
  
  
- TGGGGTAAAA TACGATAAAG TGTACTGGAT TGGTTAAAAG ACTTTACTAT ATGATTTTAG ACTGTAAATT   
  
  
- AAATAAACTT GTTTCAAACA ATAATAAAGG TAAGAATAAT ATTATATTTC ATAATGTGAA TAATGATAAT   
  
  
- AATGATGATG TTATTAATAA TAATGATATC AATCATAATA ATGATAATAA TTATGATGAT GGTGATAACA   
  
  
- ATTAAACAAT AATAATGATA ATAATAATAA TAATAATAAT AATAATAATA ATAAAAATGA TGAGAATAAT   
  
  
- GATAATAATA TAATGATGAT GTTATTAATA ATAATGATAT CAATCATAAT AAGAAAAATG ATAATAATAA   
  
  
- TTAATAATTA TAATAATCAA AATCATAAAA AAATTTTAAT AATAATCTCA TTGTGATCAT GATATTTTAA   
  
  
- CTGTAAGAAA AGAAGGGGGG AGAGTTAGAG AGTAAGAGAG AAAGAGAGTC GGGAGAGGCG ACGAGAGTGG   
  
  
- GGAGTGTGAG AGGAAGCTCC GAATAAGTCC TTTAGGGTAT AGAGGAAACT CCGAATAAGA ACCTAAACAA   
  
  
- CAACTTGTCA AGAGATATAT ACACACACAC ACATATATAT ATATATATAT ATATATATAT ATAAAAAAAA   
  
  
- AAAAAAAAAA AAAAAAAAAA CACAAATCTT TAATCTATAA GTCTATAACT TTTCAAATGC AGTTCTAAAA   
  
  
- AGAAAAGGTC TTTTAATTTA TAAAATGGAC CTTAGAAAAC TCAAGGGTAC AGATCAATTG TTAACACACA   
  
  
- TAAAATAAAG GACTTGTTAA AAGTCAACAA TTGTACTTAA AACTTACAAA GAAAAAATAA CAGAGATCCC   
  
  
- CGTATACGTA TAATCGTCTC AACAATTGAA CAATTGATCT TCGTGTACAA ACAGTATAGA AGAAACACAA   
  
  
- TCGAAAAAAT TACCAATTAA CAGTAAGACG ACACGATATA AGATATACAC GAAACTTACG CACATCGATT   
  
  
- TTATTAGGAT TTTGACTTAA TAAACTTGAA GATCTGTTTA TGTTGAGCTA CGGGTCCATT GGAGGTTTCA   
  
  
- AAAAATTTAT TGACTTGATA GAAGTTATAT TATGGTAGTA CTGTCATTCC AATCGAACGA CTCAAATAAT   
  
  
- TTATACAATT GTCAAAGGGG TCAACAGAAC AAACAAAGAG TGAACACGGT AATCCTCCGA CCCCAAACCT   
  
  
- TGCAATCTCT TCTTTCTTTT TCCCTTTTTC TCCCTCTCTT TTTCTTATCT ATATAATATC ACTACTGACT   
  
  
- ACTCATGAAC TCGTGAACTG ATGGACTAAC AAAAATAAAA AAAGTTTTAC ATTTGAAGAT AAATAAATAA   
  
  
- ATCCATTTTG AACTATTACC GTATAATAAC TTGGTACGAG ACCGTGAAAC GTGAAACGTC TAGGACACGT   
  
  
- AAAGTCATCA GACATATTAA GGATGAACAA AATTTTTT

+     Unnamed\_\_2

| Site Name | Organism | Position | Strand | Matrix score. | sequence | function |
| --- | --- | --- | --- | --- | --- | --- |
| Unnamed\_\_2 | Petroselinum hortense | 1658 | - | 9 | AACCTAACCT |  |

>PlantCARE\_20562   
+ TATTTTAGTT AGTTTTATGG AAGAATATTA AAATCATTTT TCATTTACAT GTCAATCTAT ATAATATGGT   
  
  
+ TAAATAATTG TATAATTTAT GCTATCTCAC ACGAAAATAC TTAACCTGTT TTATGAGATG ATATAGTAAA   
  
  
+ ATTTGAAATT CAATTTGTCA AATTTAGTTT TCATTGAGTT TTTTCGCTGG AAAAATATAT ACCTAATTTG   
  
  
+ AAGTCCCTAT AAATTAATCT GATAAGATTA TTTTACCAAA ACTGCTTAAA TTTTCATATT AAGTTGTTTC   
  
  
+ TGACTTTCTG ACGTTTCTGT GGTAAGAAAT CTTGTCTAAT GCCTTGTTTT ACAATCACAA TAGCCTATGG   
  
  
+ TTATCAACTT TTATAATAGT TTGAAGTAAA ACTTTTTTTT TGTTTGTTTT TAGAACTTTG ATGACCAGAT   
  
  
+ GGTTTGACTT TTTGTTGCAT ATTGATTAAA TTTCACATGT AAATGCTGAC TAACATTTAA AATTAAGGTT   
  
  
+ TGGCATATGA TGATGCCCAT TTCTGATGGC AGTTTGGTAT ATAATTTTTT TCAATTCAGT AAAAGCAAAT   
  
  
+ ACCCCATTTT ATGCTATTTC ACATGACCTA ACCAATTTTC TGAAATGATA TACTAAAATC TGACATTTAA   
  
  
+ TTTATTTGAA CAAAGTTTGT TATTATTTCC ATTCTTATTA TAATATAAAG TATTACACTT ATTACTATTA   
  
  
+ TTACTACTAC AATAATTATT ATTACTATAG TTAGTATTAT TACTATTATT AATACTACTA CCACTATTGT   
  
  
+ TAATTTGTTA TTATTACTAT TATTATTATT ATTATTATTA TTATTATTAT TATTTTTACT ACTCTTATTA   
  
  
+ CTATTATTAT ATTACTACTA CAATAATTAT TATTACTATA GTTAGTATTA TTCTTTTTAC TATTATTATT   
  
  
+ AATTATTAAT ATTATTAGTT TTAGTATTTT TTTAAAATTA TTATTAGAGT AACACTAGTA CTATAAAATT   
  
  
+ GACATTCTTT TCTTCCCCCC TCTCAATCTC TCATTCTCTC TTTCTCTCAG CCCTCTCCGC TGCTCTCACC   
  
  
+ CCTCACACTC TCCTTCGAGG CTTATTCAGG AAATCCCATA TCTCCTTTGA GGCTTATTCT TGGATTTGTT   
  
  
+ GTTGAACAGT TCTCTATATA TGTGTGTGTG TGTATATATA TATATATATA TATATATATA TATTTTTTTT   
  
  
+ TTTTTTTTTT TTTTTTTTTT GTGTTTAGAA ATTAGATATT CAGATATTGA AAAGTTTACG TCAAGATTTT   
  
  
+ TCTTTTCCAG AAAATTAAAT ATTTTACCTG GAATCTTTTG AGTTCCCATG TCTAGTTAAC AATTGTGTGT   
  
  
+ ATTTTATTTC CTGAACAATT TTCAGTTGTT AACATGAATT TTGAATGTTT CTTTTTTATT GTCTCTAGGG   
  
  
+ GCATATGCAT ATTAGCAGAG TTGTTAACTT GTTAACTAGA AGCACATGTT TGTCATATCT TCTTTGTGTT   
  
  
+ AGCTTTTTTA ATGGTTAATT GTCATTCTGC TGTGCTATAT TCTATATGTG CTTTGAATGC GTGTAGCTAA   
  
  
+ AATAATCCTA AAACTGAATT ATTTGAACTT CTAGACAAAT ACAACTCGAT GCCCAGGTAA CCTCCAAAGT   
  
  
+ TTTTTAAATA ACTGAACTAT CTTCAATATA ATACCATCAT GACAGTAAGG TTAGCTTGCT GAGTTTATTA   
  
  
+ AATATGTTAA CAGTTTCCCC AGTTGTCTTG TTTGTTTCTC ACTTGTGCCA TTAGGAGGCT GGGGTTTGGA   
  
  
+ ACGTTAGAGA AGAAAGAAAA AGGGAAAAAG AGGGAGAGAA AAAGAATAGA TATATTATAG TGATGACTGA   
  
  
+ TGAGTACTTG AGCACTTGAC TACCTGATTG TTTTTATTTT TTTCAAAATG TAAACTTCTA TTTATTTATT   
  
  
+ TAGGTAAAAC TTGATAATGG CATATTATTG AACCATGCTC TGGCACTTTG CACTTTGCAG ATCCTGTGCA   
  
  
+ TTTCAGTAGT CTGTATAATT CCTACTTGTT TTAAAAAA  

- ATAAAATCAA TCAAAATACC TTCTTATAAT TTTAGTAAAA AGTAAATGTA CAGTTAGATA TATTATACCA   
  
  
- ATTTATTAAC ATATTAAATA CGATAGAGTG TGCTTTTATG AATTGGACAA AATACTCTAC TATATCATTT   
  
  
- TAAACTTTAA GTTAAACAGT TTAAATCAAA AGTAACTCAA AAAAGCGACC TTTTTATATA TGGATTAAAC   
  
  
- TTCAGGGATA TTTAATTAGA CTATTCTAAT AAAATGGTTT TGACGAATTT AAAAGTATAA TTCAACAAAG   
  
  
- ACTGAAAGAC TGCAAAGACA CCATTCTTTA GAACAGATTA CGGAACAAAA TGTTAGTGTT ATCGGATACC   
  
  
- AATAGTTGAA AATATTATCA AACTTCATTT TGAAAAAAAA ACAAACAAAA ATCTTGAAAC TACTGGTCTA   
  
  
- CCAAACTGAA AAACAACGTA TAACTAATTT AAAGTGTACA TTTACGACTG ATTGTAAATT TTAATTCCAA   
  
  
- ACCGTATACT ACTACGGGTA AAGACTACCG TCAAACCATA TATTAAAAAA AGTTAAGTCA TTTTCGTTTA   
  
  
- TGGGGTAAAA TACGATAAAG TGTACTGGAT TGGTTAAAAG ACTTTACTAT ATGATTTTAG ACTGTAAATT   
  
  
- AAATAAACTT GTTTCAAACA ATAATAAAGG TAAGAATAAT ATTATATTTC ATAATGTGAA TAATGATAAT   
  
  
- AATGATGATG TTATTAATAA TAATGATATC AATCATAATA ATGATAATAA TTATGATGAT GGTGATAACA   
  
  
- ATTAAACAAT AATAATGATA ATAATAATAA TAATAATAAT AATAATAATA ATAAAAATGA TGAGAATAAT   
  
  
- GATAATAATA TAATGATGAT GTTATTAATA ATAATGATAT CAATCATAAT AAGAAAAATG ATAATAATAA   
  
  
- TTAATAATTA TAATAATCAA AATCATAAAA AAATTTTAAT AATAATCTCA TTGTGATCAT GATATTTTAA   
  
  
- CTGTAAGAAA AGAAGGGGGG AGAGTTAGAG AGTAAGAGAG AAAGAGAGTC GGGAGAGGCG ACGAGAGTGG   
  
  
- GGAGTGTGAG AGGAAGCTCC GAATAAGTCC TTTAGGGTAT AGAGGAAACT CCGAATAAGA ACCTAAACAA   
  
  
- CAACTTGTCA AGAGATATAT ACACACACAC ACATATATAT ATATATATAT ATATATATAT ATAAAAAAAA   
  
  
- AAAAAAAAAA AAAAAAAAAA CACAAATCTT TAATCTATAA GTCTATAACT TTTCAAATGC AGTTCTAAAA   
  
  
- AGAAAAGGTC TTTTAATTTA TAAAATGGAC CTTAGAAAAC TCAAGGGTAC AGATCAATTG TTAACACACA   
  
  
- TAAAATAAAG GACTTGTTAA AAGTCAACAA TTGTACTTAA AACTTACAAA GAAAAAATAA CAGAGATCCC   
  
  
- CGTATACGTA TAATCGTCTC AACAATTGAA CAATTGATCT TCGTGTACAA ACAGTATAGA AGAAACACAA   
  
  
- TCGAAAAAAT TACCAATTAA CAGTAAGACG ACACGATATA AGATATACAC GAAACTTACG CACATCGATT   
  
  
- TTATTAGGAT TTTGACTTAA TAAACTTGAA GATCTGTTTA TGTTGAGCTA CGGGTCCATT GGAGGTTTCA   
  
  
- AAAAATTTAT TGACTTGATA GAAGTTATAT TATGGTAGTA CTGTCATTCC AATCGAACGA CTCAAATAAT   
  
  
- TTATACAATT GTCAAAGGGG TCAACAGAAC AAACAAAGAG TGAACACGGT AATCCTCCGA CCCCAAACCT   
  
  
- TGCAATCTCT TCTTTCTTTT TCCCTTTTTC TCCCTCTCTT TTTCTTATCT ATATAATATC ACTACTGACT   
  
  
- ACTCATGAAC TCGTGAACTG ATGGACTAAC AAAAATAAAA AAAGTTTTAC ATTTGAAGAT AAATAAATAA   
  
  
- ATCCATTTTG AACTATTACC GTATAATAAC TTGGTACGAG ACCGTGAAAC GTGAAACGTC TAGGACACGT   
  
  
- AAAGTCATCA GACATATTAA GGATGAACAA AATTTTTT

+     Unnamed\_\_4

| Site Name | Organism | Position | Strand | Matrix score. | sequence | function |
| --- | --- | --- | --- | --- | --- | --- |
| Unnamed\_\_4 | Petroselinum hortense | 1035 | + | 4 | CTCC |  |
| Unnamed\_\_4 | Petroselinum hortense | 1734 | - | 4 | CTCC |  |
| Unnamed\_\_4 | Petroselinum hortense | 1092 | + | 4 | CTCC |  |
| Unnamed\_\_4 | Petroselinum hortense | 1783 | - | 4 | CTCC |  |
| Unnamed\_\_4 | Petroselinum hortense | 1060 | + | 4 | CTCC |  |
| Unnamed\_\_4 | Petroselinum hortense | 1602 | + | 4 | CTCC |  |

>PlantCARE\_20562   
+ TATTTTAGTT AGTTTTATGG AAGAATATTA AAATCATTTT TCATTTACAT GTCAATCTAT ATAATATGGT   
  
  
+ TAAATAATTG TATAATTTAT GCTATCTCAC ACGAAAATAC TTAACCTGTT TTATGAGATG ATATAGTAAA   
  
  
+ ATTTGAAATT CAATTTGTCA AATTTAGTTT TCATTGAGTT TTTTCGCTGG AAAAATATAT ACCTAATTTG   
  
  
+ AAGTCCCTAT AAATTAATCT GATAAGATTA TTTTACCAAA ACTGCTTAAA TTTTCATATT AAGTTGTTTC   
  
  
+ TGACTTTCTG ACGTTTCTGT GGTAAGAAAT CTTGTCTAAT GCCTTGTTTT ACAATCACAA TAGCCTATGG   
  
  
+ TTATCAACTT TTATAATAGT TTGAAGTAAA ACTTTTTTTT TGTTTGTTTT TAGAACTTTG ATGACCAGAT   
  
  
+ GGTTTGACTT TTTGTTGCAT ATTGATTAAA TTTCACATGT AAATGCTGAC TAACATTTAA AATTAAGGTT   
  
  
+ TGGCATATGA TGATGCCCAT TTCTGATGGC AGTTTGGTAT ATAATTTTTT TCAATTCAGT AAAAGCAAAT   
  
  
+ ACCCCATTTT ATGCTATTTC ACATGACCTA ACCAATTTTC TGAAATGATA TACTAAAATC TGACATTTAA   
  
  
+ TTTATTTGAA CAAAGTTTGT TATTATTTCC ATTCTTATTA TAATATAAAG TATTACACTT ATTACTATTA   
  
  
+ TTACTACTAC AATAATTATT ATTACTATAG TTAGTATTAT TACTATTATT AATACTACTA CCACTATTGT   
  
  
+ TAATTTGTTA TTATTACTAT TATTATTATT ATTATTATTA TTATTATTAT TATTTTTACT ACTCTTATTA   
  
  
+ CTATTATTAT ATTACTACTA CAATAATTAT TATTACTATA GTTAGTATTA TTCTTTTTAC TATTATTATT   
  
  
+ AATTATTAAT ATTATTAGTT TTAGTATTTT TTTAAAATTA TTATTAGAGT AACACTAGTA CTATAAAATT   
  
  
+ GACATTCTTT TCTTCCCCCC TCTCAATCTC TCATTCTCTC TTTCTCTCAG CCCTCTCCGC TGCTCTCACC   
  
  
+ CCTCACACTC TCCTTCGAGG CTTATTCAGG AAATCCCATA TCTCCTTTGA GGCTTATTCT TGGATTTGTT   
  
  
+ GTTGAACAGT TCTCTATATA TGTGTGTGTG TGTATATATA TATATATATA TATATATATA TATTTTTTTT   
  
  
+ TTTTTTTTTT TTTTTTTTTT GTGTTTAGAA ATTAGATATT CAGATATTGA AAAGTTTACG TCAAGATTTT   
  
  
+ TCTTTTCCAG AAAATTAAAT ATTTTACCTG GAATCTTTTG AGTTCCCATG TCTAGTTAAC AATTGTGTGT   
  
  
+ ATTTTATTTC CTGAACAATT TTCAGTTGTT AACATGAATT TTGAATGTTT CTTTTTTATT GTCTCTAGGG   
  
  
+ GCATATGCAT ATTAGCAGAG TTGTTAACTT GTTAACTAGA AGCACATGTT TGTCATATCT TCTTTGTGTT   
  
  
+ AGCTTTTTTA ATGGTTAATT GTCATTCTGC TGTGCTATAT TCTATATGTG CTTTGAATGC GTGTAGCTAA   
  
  
+ AATAATCCTA AAACTGAATT ATTTGAACTT CTAGACAAAT ACAACTCGAT GCCCAGGTAA CCTCCAAAGT   
  
  
+ TTTTTAAATA ACTGAACTAT CTTCAATATA ATACCATCAT GACAGTAAGG TTAGCTTGCT GAGTTTATTA   
  
  
+ AATATGTTAA CAGTTTCCCC AGTTGTCTTG TTTGTTTCTC ACTTGTGCCA TTAGGAGGCT GGGGTTTGGA   
  
  
+ ACGTTAGAGA AGAAAGAAAA AGGGAAAAAG AGGGAGAGAA AAAGAATAGA TATATTATAG TGATGACTGA   
  
  
+ TGAGTACTTG AGCACTTGAC TACCTGATTG TTTTTATTTT TTTCAAAATG TAAACTTCTA TTTATTTATT   
  
  
+ TAGGTAAAAC TTGATAATGG CATATTATTG AACCATGCTC TGGCACTTTG CACTTTGCAG ATCCTGTGCA   
  
  
+ TTTCAGTAGT CTGTATAATT CCTACTTGTT TTAAAAAA  

- ATAAAATCAA TCAAAATACC TTCTTATAAT TTTAGTAAAA AGTAAATGTA CAGTTAGATA TATTATACCA   
  
  
- ATTTATTAAC ATATTAAATA CGATAGAGTG TGCTTTTATG AATTGGACAA AATACTCTAC TATATCATTT   
  
  
- TAAACTTTAA GTTAAACAGT TTAAATCAAA AGTAACTCAA AAAAGCGACC TTTTTATATA TGGATTAAAC   
  
  
- TTCAGGGATA TTTAATTAGA CTATTCTAAT AAAATGGTTT TGACGAATTT AAAAGTATAA TTCAACAAAG   
  
  
- ACTGAAAGAC TGCAAAGACA CCATTCTTTA GAACAGATTA CGGAACAAAA TGTTAGTGTT ATCGGATACC   
  
  
- AATAGTTGAA AATATTATCA AACTTCATTT TGAAAAAAAA ACAAACAAAA ATCTTGAAAC TACTGGTCTA   
  
  
- CCAAACTGAA AAACAACGTA TAACTAATTT AAAGTGTACA TTTACGACTG ATTGTAAATT TTAATTCCAA   
  
  
- ACCGTATACT ACTACGGGTA AAGACTACCG TCAAACCATA TATTAAAAAA AGTTAAGTCA TTTTCGTTTA   
  
  
- TGGGGTAAAA TACGATAAAG TGTACTGGAT TGGTTAAAAG ACTTTACTAT ATGATTTTAG ACTGTAAATT   
  
  
- AAATAAACTT GTTTCAAACA ATAATAAAGG TAAGAATAAT ATTATATTTC ATAATGTGAA TAATGATAAT   
  
  
- AATGATGATG TTATTAATAA TAATGATATC AATCATAATA ATGATAATAA TTATGATGAT GGTGATAACA   
  
  
- ATTAAACAAT AATAATGATA ATAATAATAA TAATAATAAT AATAATAATA ATAAAAATGA TGAGAATAAT   
  
  
- GATAATAATA TAATGATGAT GTTATTAATA ATAATGATAT CAATCATAAT AAGAAAAATG ATAATAATAA   
  
  
- TTAATAATTA TAATAATCAA AATCATAAAA AAATTTTAAT AATAATCTCA TTGTGATCAT GATATTTTAA   
  
  
- CTGTAAGAAA AGAAGGGGGG AGAGTTAGAG AGTAAGAGAG AAAGAGAGTC GGGAGAGGCG ACGAGAGTGG   
  
  
- GGAGTGTGAG AGGAAGCTCC GAATAAGTCC TTTAGGGTAT AGAGGAAACT CCGAATAAGA ACCTAAACAA   
  
  
- CAACTTGTCA AGAGATATAT ACACACACAC ACATATATAT ATATATATAT ATATATATAT ATAAAAAAAA   
  
  
- AAAAAAAAAA AAAAAAAAAA CACAAATCTT TAATCTATAA GTCTATAACT TTTCAAATGC AGTTCTAAAA   
  
  
- AGAAAAGGTC TTTTAATTTA TAAAATGGAC CTTAGAAAAC TCAAGGGTAC AGATCAATTG TTAACACACA   
  
  
- TAAAATAAAG GACTTGTTAA AAGTCAACAA TTGTACTTAA AACTTACAAA GAAAAAATAA CAGAGATCCC   
  
  
- CGTATACGTA TAATCGTCTC AACAATTGAA CAATTGATCT TCGTGTACAA ACAGTATAGA AGAAACACAA   
  
  
- TCGAAAAAAT TACCAATTAA CAGTAAGACG ACACGATATA AGATATACAC GAAACTTACG CACATCGATT   
  
  
- TTATTAGGAT TTTGACTTAA TAAACTTGAA GATCTGTTTA TGTTGAGCTA CGGGTCCATT GGAGGTTTCA   
  
  
- AAAAATTTAT TGACTTGATA GAAGTTATAT TATGGTAGTA CTGTCATTCC AATCGAACGA CTCAAATAAT   
  
  
- TTATACAATT GTCAAAGGGG TCAACAGAAC AAACAAAGAG TGAACACGGT AATCCTCCGA CCCCAAACCT   
  
  
- TGCAATCTCT TCTTTCTTTT TCCCTTTTTC TCCCTCTCTT TTTCTTATCT ATATAATATC ACTACTGACT   
  
  
- ACTCATGAAC TCGTGAACTG ATGGACTAAC AAAAATAAAA AAAGTTTTAC ATTTGAAGAT AAATAAATAA   
  
  
- ATCCATTTTG AACTATTACC GTATAATAAC TTGGTACGAG ACCGTGAAAC GTGAAACGTC TAGGACACGT   
  
  
- AAAGTCATCA GACATATTAA GGATGAACAA AATTTTTT

+     as-1

| Site Name | Organism | Position | Strand | Matrix score. | sequence | function |
| --- | --- | --- | --- | --- | --- | --- |
| as-1 | Arabidopsis thaliana | 289 | + | 5 | TGACG |  |
| as-1 | Arabidopsis thaliana | 1249 | - | 5 | TGACG |  |

>PlantCARE\_20562   
+ TATTTTAGTT AGTTTTATGG AAGAATATTA AAATCATTTT TCATTTACAT GTCAATCTAT ATAATATGGT   
  
  
+ TAAATAATTG TATAATTTAT GCTATCTCAC ACGAAAATAC TTAACCTGTT TTATGAGATG ATATAGTAAA   
  
  
+ ATTTGAAATT CAATTTGTCA AATTTAGTTT TCATTGAGTT TTTTCGCTGG AAAAATATAT ACCTAATTTG   
  
  
+ AAGTCCCTAT AAATTAATCT GATAAGATTA TTTTACCAAA ACTGCTTAAA TTTTCATATT AAGTTGTTTC   
  
  
+ TGACTTTCTG ACGTTTCTGT GGTAAGAAAT CTTGTCTAAT GCCTTGTTTT ACAATCACAA TAGCCTATGG   
  
  
+ TTATCAACTT TTATAATAGT TTGAAGTAAA ACTTTTTTTT TGTTTGTTTT TAGAACTTTG ATGACCAGAT   
  
  
+ GGTTTGACTT TTTGTTGCAT ATTGATTAAA TTTCACATGT AAATGCTGAC TAACATTTAA AATTAAGGTT   
  
  
+ TGGCATATGA TGATGCCCAT TTCTGATGGC AGTTTGGTAT ATAATTTTTT TCAATTCAGT AAAAGCAAAT   
  
  
+ ACCCCATTTT ATGCTATTTC ACATGACCTA ACCAATTTTC TGAAATGATA TACTAAAATC TGACATTTAA   
  
  
+ TTTATTTGAA CAAAGTTTGT TATTATTTCC ATTCTTATTA TAATATAAAG TATTACACTT ATTACTATTA   
  
  
+ TTACTACTAC AATAATTATT ATTACTATAG TTAGTATTAT TACTATTATT AATACTACTA CCACTATTGT   
  
  
+ TAATTTGTTA TTATTACTAT TATTATTATT ATTATTATTA TTATTATTAT TATTTTTACT ACTCTTATTA   
  
  
+ CTATTATTAT ATTACTACTA CAATAATTAT TATTACTATA GTTAGTATTA TTCTTTTTAC TATTATTATT   
  
  
+ AATTATTAAT ATTATTAGTT TTAGTATTTT TTTAAAATTA TTATTAGAGT AACACTAGTA CTATAAAATT   
  
  
+ GACATTCTTT TCTTCCCCCC TCTCAATCTC TCATTCTCTC TTTCTCTCAG CCCTCTCCGC TGCTCTCACC   
  
  
+ CCTCACACTC TCCTTCGAGG CTTATTCAGG AAATCCCATA TCTCCTTTGA GGCTTATTCT TGGATTTGTT   
  
  
+ GTTGAACAGT TCTCTATATA TGTGTGTGTG TGTATATATA TATATATATA TATATATATA TATTTTTTTT   
  
  
+ TTTTTTTTTT TTTTTTTTTT GTGTTTAGAA ATTAGATATT CAGATATTGA AAAGTTTACG TCAAGATTTT   
  
  
+ TCTTTTCCAG AAAATTAAAT ATTTTACCTG GAATCTTTTG AGTTCCCATG TCTAGTTAAC AATTGTGTGT   
  
  
+ ATTTTATTTC CTGAACAATT TTCAGTTGTT AACATGAATT TTGAATGTTT CTTTTTTATT GTCTCTAGGG   
  
  
+ GCATATGCAT ATTAGCAGAG TTGTTAACTT GTTAACTAGA AGCACATGTT TGTCATATCT TCTTTGTGTT   
  
  
+ AGCTTTTTTA ATGGTTAATT GTCATTCTGC TGTGCTATAT TCTATATGTG CTTTGAATGC GTGTAGCTAA   
  
  
+ AATAATCCTA AAACTGAATT ATTTGAACTT CTAGACAAAT ACAACTCGAT GCCCAGGTAA CCTCCAAAGT   
  
  
+ TTTTTAAATA ACTGAACTAT CTTCAATATA ATACCATCAT GACAGTAAGG TTAGCTTGCT GAGTTTATTA   
  
  
+ AATATGTTAA CAGTTTCCCC AGTTGTCTTG TTTGTTTCTC ACTTGTGCCA TTAGGAGGCT GGGGTTTGGA   
  
  
+ ACGTTAGAGA AGAAAGAAAA AGGGAAAAAG AGGGAGAGAA AAAGAATAGA TATATTATAG TGATGACTGA   
  
  
+ TGAGTACTTG AGCACTTGAC TACCTGATTG TTTTTATTTT TTTCAAAATG TAAACTTCTA TTTATTTATT   
  
  
+ TAGGTAAAAC TTGATAATGG CATATTATTG AACCATGCTC TGGCACTTTG CACTTTGCAG ATCCTGTGCA   
  
  
+ TTTCAGTAGT CTGTATAATT CCTACTTGTT TTAAAAAA  

- ATAAAATCAA TCAAAATACC TTCTTATAAT TTTAGTAAAA AGTAAATGTA CAGTTAGATA TATTATACCA   
  
  
- ATTTATTAAC ATATTAAATA CGATAGAGTG TGCTTTTATG AATTGGACAA AATACTCTAC TATATCATTT   
  
  
- TAAACTTTAA GTTAAACAGT TTAAATCAAA AGTAACTCAA AAAAGCGACC TTTTTATATA TGGATTAAAC   
  
  
- TTCAGGGATA TTTAATTAGA CTATTCTAAT AAAATGGTTT TGACGAATTT AAAAGTATAA TTCAACAAAG   
  
  
- ACTGAAAGAC TGCAAAGACA CCATTCTTTA GAACAGATTA CGGAACAAAA TGTTAGTGTT ATCGGATACC   
  
  
- AATAGTTGAA AATATTATCA AACTTCATTT TGAAAAAAAA ACAAACAAAA ATCTTGAAAC TACTGGTCTA   
  
  
- CCAAACTGAA AAACAACGTA TAACTAATTT AAAGTGTACA TTTACGACTG ATTGTAAATT TTAATTCCAA   
  
  
- ACCGTATACT ACTACGGGTA AAGACTACCG TCAAACCATA TATTAAAAAA AGTTAAGTCA TTTTCGTTTA   
  
  
- TGGGGTAAAA TACGATAAAG TGTACTGGAT TGGTTAAAAG ACTTTACTAT ATGATTTTAG ACTGTAAATT   
  
  
- AAATAAACTT GTTTCAAACA ATAATAAAGG TAAGAATAAT ATTATATTTC ATAATGTGAA TAATGATAAT   
  
  
- AATGATGATG TTATTAATAA TAATGATATC AATCATAATA ATGATAATAA TTATGATGAT GGTGATAACA   
  
  
- ATTAAACAAT AATAATGATA ATAATAATAA TAATAATAAT AATAATAATA ATAAAAATGA TGAGAATAAT   
  
  
- GATAATAATA TAATGATGAT GTTATTAATA ATAATGATAT CAATCATAAT AAGAAAAATG ATAATAATAA   
  
  
- TTAATAATTA TAATAATCAA AATCATAAAA AAATTTTAAT AATAATCTCA TTGTGATCAT GATATTTTAA   
  
  
- CTGTAAGAAA AGAAGGGGGG AGAGTTAGAG AGTAAGAGAG AAAGAGAGTC GGGAGAGGCG ACGAGAGTGG   
  
  
- GGAGTGTGAG AGGAAGCTCC GAATAAGTCC TTTAGGGTAT AGAGGAAACT CCGAATAAGA ACCTAAACAA   
  
  
- CAACTTGTCA AGAGATATAT ACACACACAC ACATATATAT ATATATATAT ATATATATAT ATAAAAAAAA   
  
  
- AAAAAAAAAA AAAAAAAAAA CACAAATCTT TAATCTATAA GTCTATAACT TTTCAAATGC AGTTCTAAAA   
  
  
- AGAAAAGGTC TTTTAATTTA TAAAATGGAC CTTAGAAAAC TCAAGGGTAC AGATCAATTG TTAACACACA   
  
  
- TAAAATAAAG GACTTGTTAA AAGTCAACAA TTGTACTTAA AACTTACAAA GAAAAAATAA CAGAGATCCC   
  
  
- CGTATACGTA TAATCGTCTC AACAATTGAA CAATTGATCT TCGTGTACAA ACAGTATAGA AGAAACACAA   
  
  
- TCGAAAAAAT TACCAATTAA CAGTAAGACG ACACGATATA AGATATACAC GAAACTTACG CACATCGATT   
  
  
- TTATTAGGAT TTTGACTTAA TAAACTTGAA GATCTGTTTA TGTTGAGCTA CGGGTCCATT GGAGGTTTCA   
  
  
- AAAAATTTAT TGACTTGATA GAAGTTATAT TATGGTAGTA CTGTCATTCC AATCGAACGA CTCAAATAAT   
  
  
- TTATACAATT GTCAAAGGGG TCAACAGAAC AAACAAAGAG TGAACACGGT AATCCTCCGA CCCCAAACCT   
  
  
- TGCAATCTCT TCTTTCTTTT TCCCTTTTTC TCCCTCTCTT TTTCTTATCT ATATAATATC ACTACTGACT   
  
  
- ACTCATGAAC TCGTGAACTG ATGGACTAAC AAAAATAAAA AAAGTTTTAC ATTTGAAGAT AAATAAATAA   
  
  
- ATCCATTTTG AACTATTACC GTATAATAAC TTGGTACGAG ACCGTGAAAC GTGAAACGTC TAGGACACGT   
  
  
- AAAGTCATCA GACATATTAA GGATGAACAA AATTTTTT
